# Probing the Origin of Affinity in the GM1-Cholera Toxin Complex through Site-Selective Editing with Fluorine

**DOI:** 10.1021/acscentsci.4c00622

**Published:** 2024-07-12

**Authors:** Christina Jordan, Taiki Hayashi, Arnelle Löbbert, Jingran Fan, Charlotte S. Teschers, Kathrin Siebold, Marialuisa Aufiero, Felix Pape, Emma Campbell, Alexander Axer, Kathrin Bussmann, Klaus Bergander, Jesko Köhnke, Alvar D. Gossert, Ryan Gilmour

**Affiliations:** † Institute for Organic Chemistry, University of Münster, 48149 Münster, Germany; ‡ Department of Biology, ETH Zürich, 8093 Zürich, Switzerland; § Institut für Lebensmittelchemie, Leibniz Universität Hannover, 30167 Hannover, Germany

## Abstract

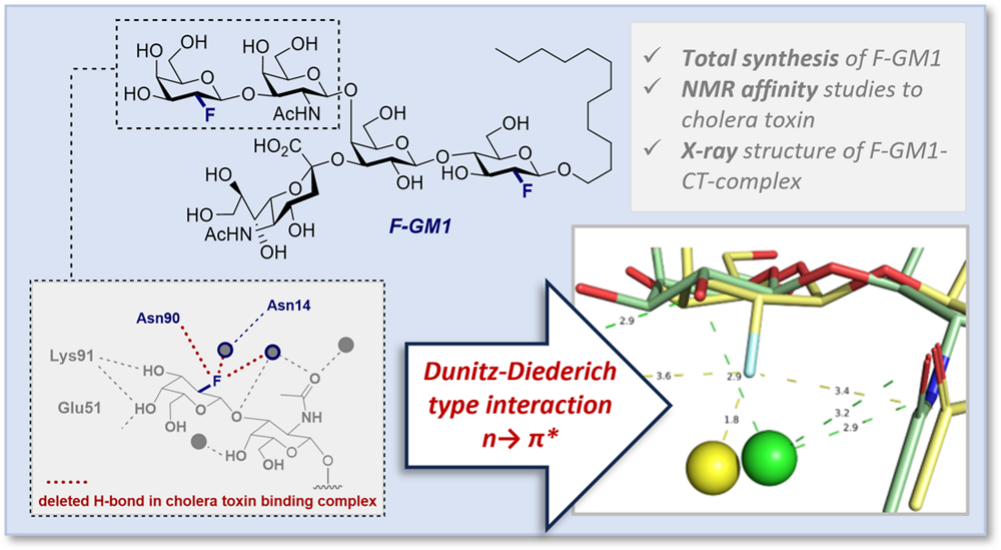

Carbohydrates regulate an inimitable spectrum of biological functions, yet successfully leveraging this therapeutic avenue continues to be frustrated by low affinities with glycan-specific proteins. A conspicuous exception is the interaction of monosialotetrahexosylganglioside (GM1) with the carbohydrate-recognition domain of cholera toxin from *Vibrio cholerae*: this is one of the strongest protein–carbohydrate interactions known. To establish the importance of a long-discussed key hydrogen bond between C2 of the terminal galactose of GM1 and the B subunit pentamer of cholera toxin (CTB_5_), the total synthesis of a selectively fluorinated GM1 epitope was conducted in 19 steps. This process of molecular editing (O^δ-^H → F^δ-^) strategically deletes the hydrogen bond donor while retaining the localized partial charge of the substituent. Comparison of the binding affinity of F-GM1/CTB_5_ with native GM1, the GM1 carbohydrate epitope, and *meta*-mononitrophenyl-α-galactoside (MNPG) revealed a trend that fully supports the importance of this key interaction. These NMR data suggest that F-GM1 binds in a closely similar conformation as native GM1. Crystallographic analyses of the complex also confirm that the OH → F bioisosteric exchange at C2 of the terminal galactose induces a ring conformation that eliminates key hydrogen bonds: these interactions are compensated for by inter- and intramolecular fluorine-specific interactions.

## Abstract 2

The stereocontrolled synthesis of F-GM1 has enabled the affinity of GM1 and cholera toxin (CTB_5_) to be interrogated by NMR and protein crystallography.

## Introduction

**Figure 1:**
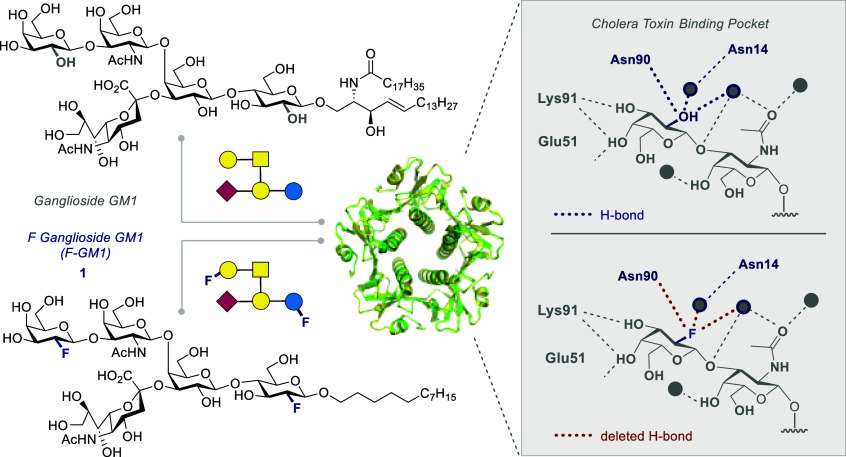
Naturally occurring ganglioside GM1 and the fluorinated analogue F-GM1 (**1**) (Asn = asparagine, Lys = lysine, Glu = glutamic acid) next to cholera toxin (CTB_5_). Key hydrogen bonds are shown in the insets (right).

Carbohydrates are biology′s *lingua franca*: the molecular basis of a post-translational communication system that spans the animal kingdoms.^1^ Understanding this language, and contributing to it to enable function, is a core objective in glycobiology and synthetic chemistry.^2^ The evolutionary success of glycans reflects their immense structural complexity and diversity;^3^ this, in turn, encodes for precise hydrogen bond networks and ensures remarkable fidelity for specific target proteins.^4^ Despite the ubiquity of glycan–protein interactions in cell behavior, these processes are characterized by binding affinities (mM) that are often too weak to be leveraged in a therapeutic context.^5^ This juxtaposition continues to frustrate translation of carbohydrate-specific recognition blueprints to challenges in contemporary drug discovery and vaccine development.^6^ A conspicuous exception is the interaction of the human ganglioside GM1 with the B subunit pentamer of cholera toxin (CTB_5_) from *Vibrio cholerae*,^7^ an interaction for which affinities in the range from *K*_D_ = 4.6 × 10^–10^ M^8a^ to *K*_D_ = 5.0 × 10^–8^ M^8b^ have been reported. The crystal structure of the CTB_5_-GM1 epitope complex was published in 1994 by Merritt et al.^9^ and reveals important H-bonds between the C2 C(sp^3^)–OH of the terminal galactose and Asn90, Asn14, and a water molecule (Figure 1, right). It is reported that essentially all of the binding energy is due to interactions with this galactose residue and the sialic acid motif. Therefore, to investigate the contribution of this pivotal hydrogen bond at the terminal galactose in enabling the remarkable affinity of the CTB_5_-GM1 complex, a chemical biology tool was conceived in which this OH was substituted by fluorine (Figure 1, left).^10^ To mitigate hydrolytic cleavage of the lipid mimetic, fluorination at C2 of the glucose unit, which is not directly involved in the interaction with CTB_5_ but carries the lipid anchor, was envisaged.^11^ Since site-selective deoxyfluorination of commercial GM1 is not synthetically feasible, success would be conditional on the development of a stereocontrolled, *de novo* synthesis of the fluorinated pentasaccharide.^12,13^ Strategically leveraging fluorine-directed glycosylation^14^ is appealing to introduce the key galactose (β1 → 3) and glucose units in a stereocontrolled manner.

In addition, bioisosteric editing with fluorine would not only preserve the localized electronic character^15^ but would serve to enhance metabolic stability^16^ and provide ^19^F NMR reporter nuclei for solution-phase affinity studies.^17^ This would allow for a qualitative ranking of F-GM1 to be established relative to the native ligand and known competitive binders.^18^ Furthermore, it was envisaged that crystallographic analysis of the CTB_5_–F-GM1 complex would provide additional structural insights into the origin of this remarkable affinity: this would have translational implications in understanding signal transduction and vaccine development.^19^

## Results and Discussion

**Figure 2:**
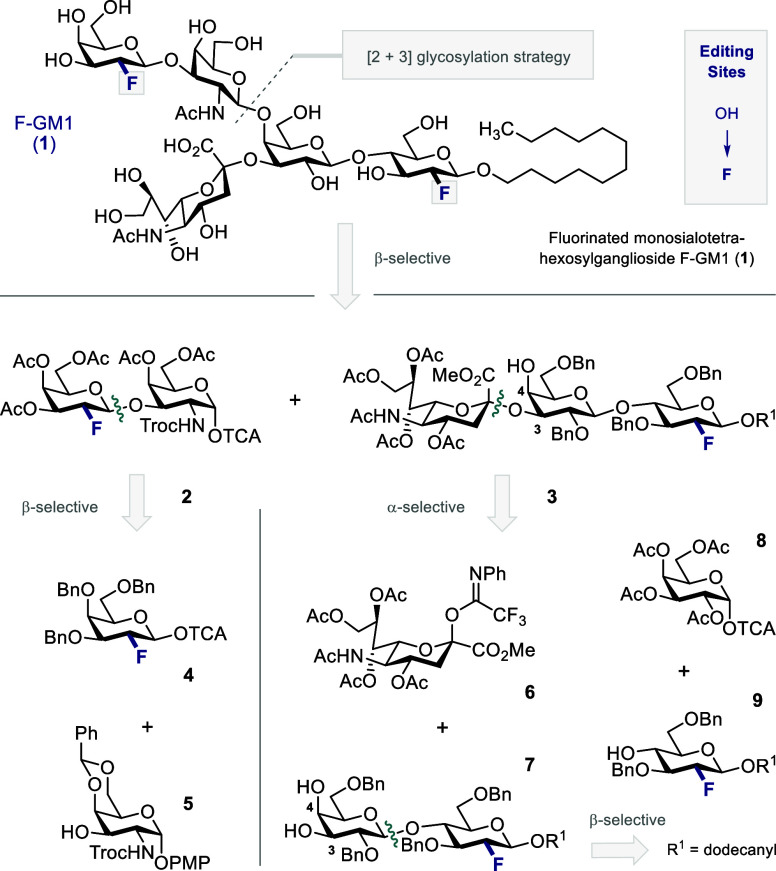
Retrosynthetic study of fluorinated GM1 analogue (F-GM1) **1**; R^1^ = 1-dodecyl, TCA = trichloroacetimidoyl, PMP = *p*-methoxylphenyl.

Retrosynthetically, the target molecule F-GM1 (**1**) was constructed via a [2 + 3] approach that would emulate the biosynthesis of the native epitope (Figure 2): the lower trisaccharide is the venerable GM3 epitope (**3**), and so, this strategy provides entry to a multitude of fluorinated gangliosides. This disconnection of the glycosidic β-GalNAc(1,4)Gal-linkage furnishes disaccharide donor **2** (northern branch) and trisaccharide acceptor **3** (F-GM3-motif). It was envisaged that F-disaccharide **2** could be generated by fluorine-directed glycosylation from the imidate donor **4**(14b) and the protected GalNAc acceptor **5**.^20^ The F-GM3 analogue **3** would be obtained through regio- and α-stereoselective union of sialic acid derivative **6**(21) with the fluorinated lactose **7** at C3. The precursor **7** would be assembled from fluorinated glucose acceptor **9** and per-acetylated galactose donor **8** exploiting β-selective neighboring group participation.^22^

**Scheme 1:**
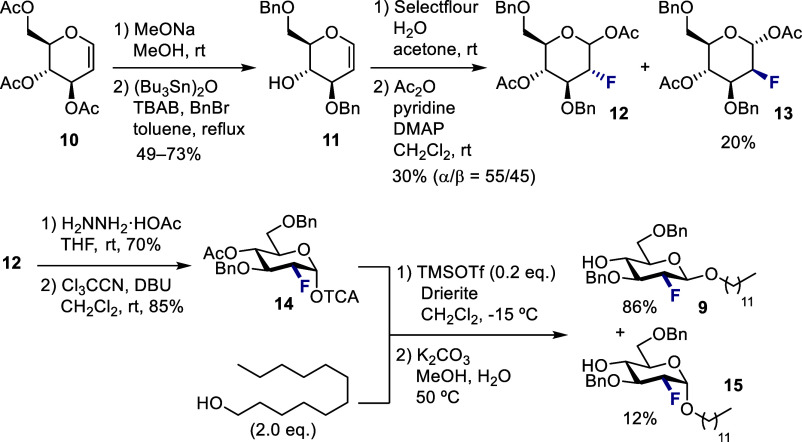
Preparation of Glycosyl Acceptor **9**

Synthesis of the target molecule commenced with the preparation of acceptor **9** (Scheme 1). Following deacetylation of commercially available glycal **10**, regioselective dibenzylation of the triol [(Bu_3_Sn)_2_O, tetrabutylammonium bromide (TBAB), BnBr in toluene, reflux]^23^ was achieved to access 4-OH glycal **11**. Hydroxyfluorination of **11** with Selectfluor followed by acetylation afforded fluorinated acetates **12** and **13** in 30% and 20% yield, respectively. Compound **12**, exhibiting the gluco-type configuration, was then processed further to the fluorinated ganglioside epitope. Selective deacetylation of the anomeric position (H_2_NNH_2_·HOAc in THF) and imidate donor formation (Cl_3_CCN, DBU in CH_2_Cl_2_) generated glycosyl donor **14**. Upon treatment of **14** and 1-dodecanol with catalytic amounts of trimethylsilyl trifluoromethanesulfonate (TMSOTf) (0.2 equiv) in CH_2_Cl_2_ at −15 °C, the glycosylation proceeded smoothly to favor the desired β-anomer (β/α = 88/12, determined by ^19^F NMR). This selectivity for the β-glycoside is grounded in a match of the C2 configuration and the weakly inductive nature of the benzyl protecting groups.^14a^ Removal of the *O*-4-Ac group enabled the separation of both anomers, thereby allowing the desired β-anomer **9** to be generated in 86% yield; the minor α-anomer **15** was also isolated (12% yield).

**Scheme 2:**
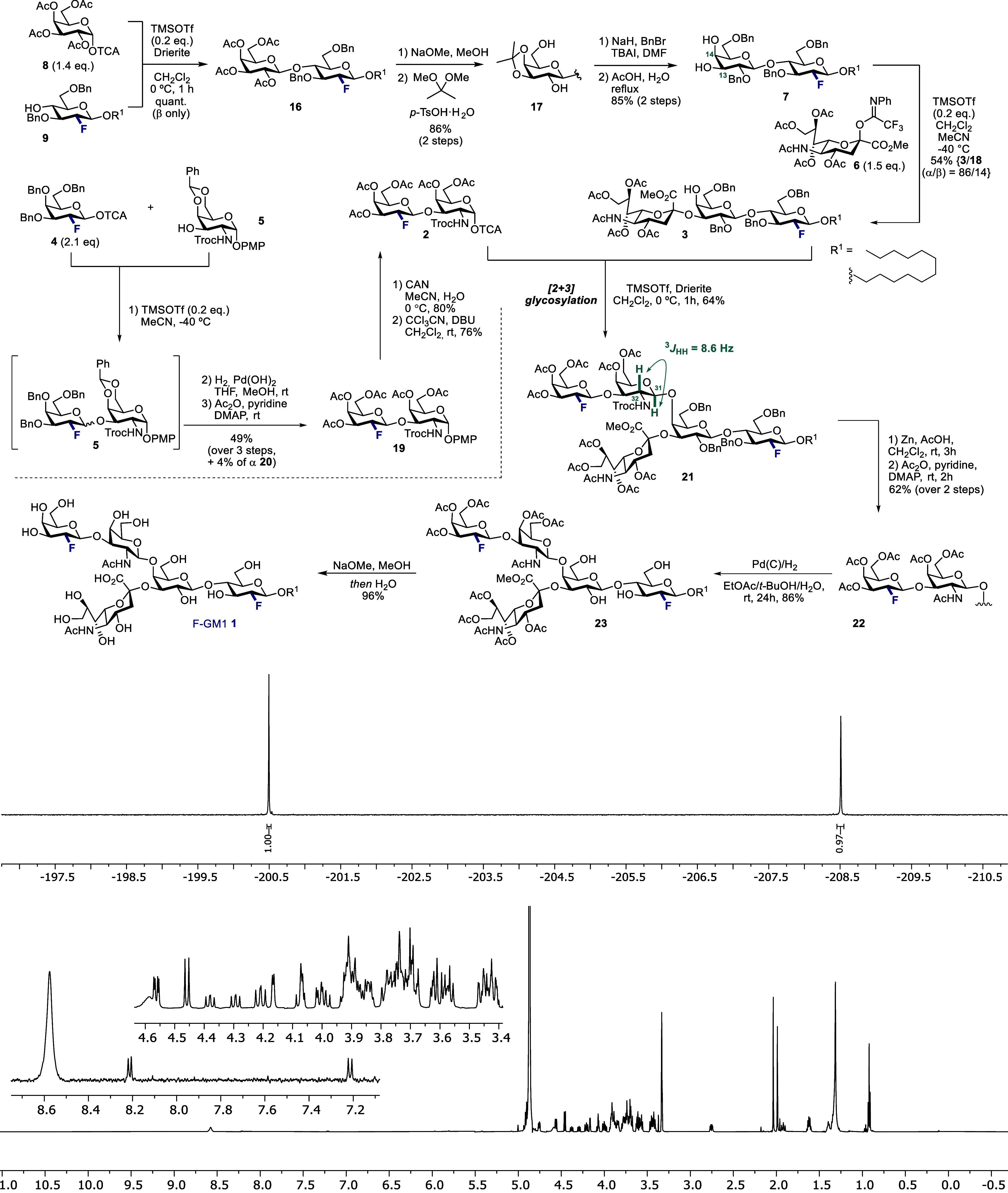
Overview of F-GM1 (**1**) Synthesis Where Top Row Discloses the Assembly of the F-GM3 (**3**) Structural Motif, While the Left Side Shows the Construction of the Northern Branch Epitope **2** Key step is the [2 + 3] glycosylation of donor **2** and acceptor **3** to furnish pentasaccharide **21** followed by global deprotection to afford final F-GM1 (**1**). Bottom: ^19^F {^1^H} and ^1^H NMR spectra of F-GM1 (**1**).

With the reducing-end fragment **9** in hand, the synthesis of trisaccharide **3** was initiated (Scheme 2). The glycosylation of galactose donor **8** with glucose acceptor **9** was catalyzed by TMSOTf, which enabled the disaccharide **16** to be obtained quantitatively with exclusive β-selectivity. Global deacetylation followed by acetonide formation (2,2-dimethoxypropane, *p*-TsOH·H_2_O)^24^ afforded diol **17** in 86% over 2 steps. Subsequent benzyl protection of the remaining hydroxyl groups and acetonide hydrolysis under acidic conditions forged the lactose acceptor **7** in 85% yield over two steps.

Next, glycosylation with sialyl donor **6** was conducted at −40 °C in a mixture of CH_2_Cl_2_ and the coordinating solvent MeCN. The reaction proceeded regioselectively at the *O*-13 position to afford the trisaccharides **3** and **18** (α- and β-anomer, respectively). Expectedly, the desired α-anomer **3** was formed as the major product under these chemical sialylation conditions with good levels of diastereoselectivity (α/β = 86/14, determined by ^19^F NMR). Gratifyingly, these two species could be separated by preparative HPLC to provide **3** as a single diastereomer. The northern disaccharide fragment was assembled from the fluorinated galacto-type donor **4**(14b) and the galactosamine acceptor **5**(20) (Scheme 2).

A sequence of fluorine-directed glycosylation, hydrogenolysis, and acetylation afforded the β-anomer **19** in 49% yield. The minor α-anomer **20** was isolated in 4% yield over three steps for completeness. Compound **19** was then processed to the disaccharide donor **2** by oxidative cleavage of the *p*-methoxylphenyl (PMP) group [ceric ammonium nitrate (CAN) in wet MeCN] and subsequent trichloroacetimidate formation (80% and 76% yield, respectively). To construct the protected pentassaccharide core, donor **2** and acceptor **3** were unified in a [2 + 3] glycosylation event promoted by catalytic amounts of TMSOTf (Scheme 2). Gratifyingly, this enabled the protected F-GM1 scaffold (**21**) to be forged in 64% isolated yield.

^19^F NMR spectroscopy revealed a mixture of three species in CDCl_3_ (ratio 7.3:2.4:0.3) of which the major species was identified as having the desired β-configuration, as is evident from inspection of the H31–H32 scalar coupling (^3^*J*_HH_ = 8.6 Hz, Scheme 2). Intriguingly, these three species proved to be discrete conformers of **21**, which was investigated by variable temperature NMR studies (*vide infra*).

**Figure 3:**
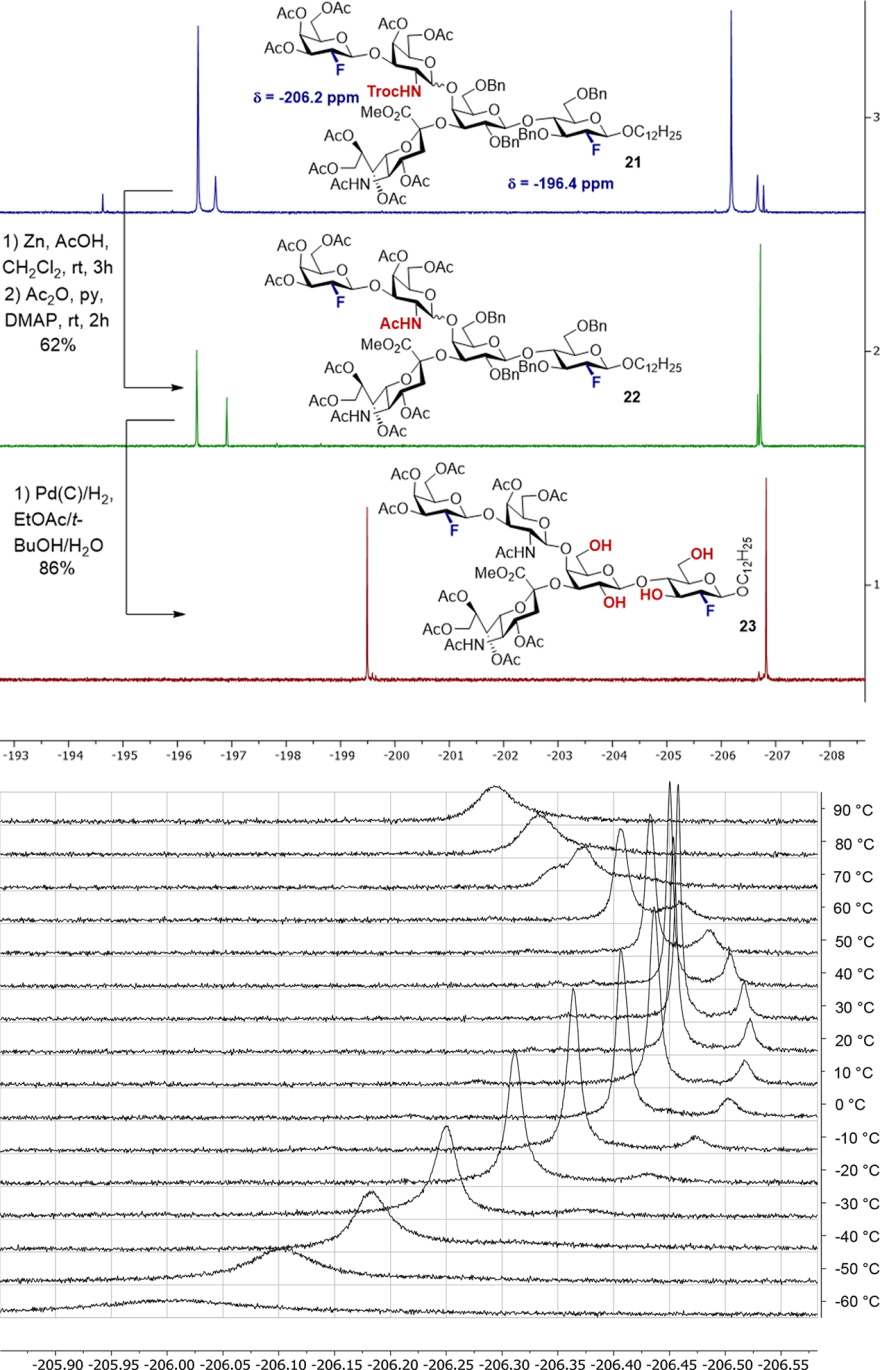
Top: Comparison of ^19^F NMR spectra of compounds **21** (top), **22** (middle), and **23** (bottom) after each deprotection sequence, which shows the increased conformational freedom and the presence of only β-configured products. Bottom: ^19^F NMR spectra of **22** in toluene-*d*_8_ show dynamic behavior of the two species.

For the end game of this synthesis campaign, reductive cleavage of the *N*-Troc group (Zn, AcOH in CH_2_Cl_2_) enabled the free amine to be acetyl-protected to afford **22** in 62% yield (conformer ratio 8:2, as determined by ^19^F NMR in CDCl_3_). Hydrogenolysis (H_2_, Pd/C) of all four Bn groups proceeded smoothly in a mixture of EtOAc/*t-*BuOH/H_2_O^25^ to afford the semiprotected F-GM1 epitope **23** in 86% isolated yield. Interestingly, the ^19^F NMR spectrum of the crude reaction mixture of the hydrogenation after workup (filtration, concentration) revealed a 95:5 mixture of two doubly fluorinated species. This increase in the major/minor ratio (7:3 ratio for **21** and 8:2 ratio for **22**, Figure 3) prompted us to further investigate the composition of the carbohydrate mixture.

Confronted with the possibilities of (a) epimerization occurring during the deprotection or (b) the presence of conformers, Occam’s razor was applied. Alteration of the diastereomeric ratio during the deprotection step was deemed unlikely, and it seemed more feasible that protecting group cleavage would be accompanied by steric decompression: this would conceivably lower the barrier for conformer interconversion.

To test this hypothesis, the 8:2 mixture of compound **22** was subjected to variable temperature (VT) NMR spectroscopy. Analysis of the VT-^19^F{^1^H} NMR spectra revealed dynamic behavior of the two species at temperatures above 40 °C, as evidenced by line broadening as a consequence of exchange (Figure 3, bottom; for further details and VT-^1^H NMR spectra, see the Supporting Information). Interestingly, these two species exchange slowly on the NMR time scale even at 90 °C. The dynamic behavior unambiguously proves that the two species are not diastereomers. Instead, this experiment substantiates the fact that the final glycosylation proceeds with excellent levels of diastereocontrol to yield a 7:3 mixture of two different conformers, both of which are β-configured.

Finally, the target F-GM1 (**1**) pentasaccharide was obtained in 96% yield after hydrolysis of the Ac protecting groups and the methyl ester using NaOMe in MeOH/H_2_O. The complexity of **1** is reflected by the ^1^H NMR spectrum (shown in Scheme 2), which is characterized by overlapping signals whose multiplicities could only be analyzed by means of 2D spectra (for complete assignment, see the Supporting Information). By contrast, the ^19^F NMR spectrum (Scheme 2) clearly showed the existence of a single diastereomer and two fluorine atoms: this underscores the utility of ^19^F NMR as a powerful technique to facilitate the analyses of such glycomimetics.

With the final F-GM1 product in hand, efforts were focused on characterizing the interaction of the ligand with CTB_5_ in detail and comparing it with that of natural GM1. Initially, the affinity of F-GM1 was determined to assess the impact of the changes in the hydrogen bonding network on the binding energy. The simple ^19^F NMR spectrum of F-GM1 lends itself to binding assays to study the interaction with complex biomolecules as it is devoid of any background signals of proteins or buffer components.^26,27^

**Figure 4:**
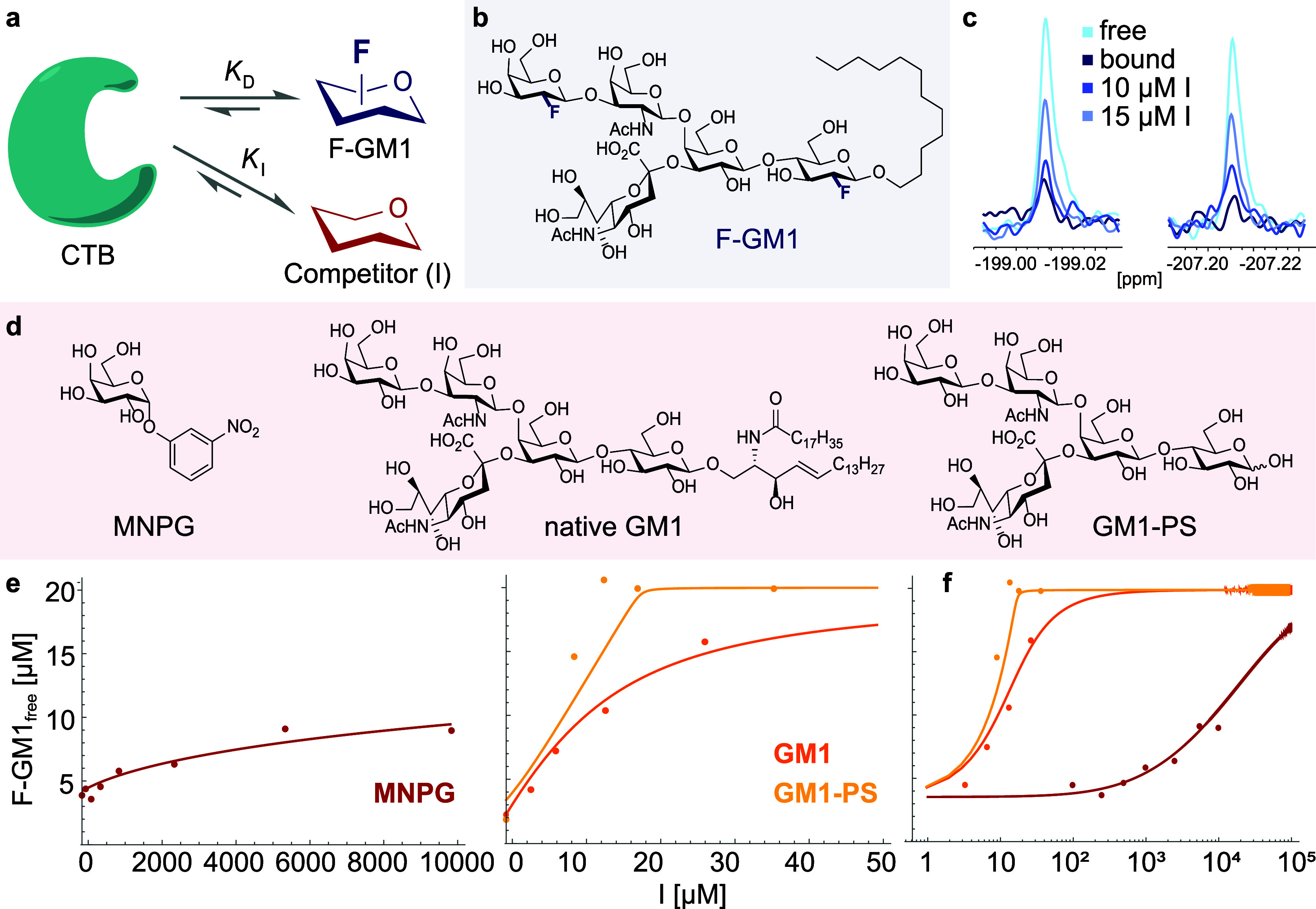
F-GM1 (**1**) and the competitive inhibitors for binding investigations to cholera toxin subunit B (CTB_5_, green). (a) Scheme of the competition binding assay using F-GM1 (blue, with dissociation constant *K*_D_) as reporter ligand and competitive inhibitors (red, with respective dissociation constants *K*_I_). Molecular structures are shown in panels (b) and (d); MNPG = *meta*-mononitrophenyl-α-galactoside. (c) ^19^F NMR signals of free F-GM1 (20 μM) and in the presence of CTB (17 μM monomer concentration) with increasing concentrations of GM1. (e) Fitted curves to this type of data for all inhibitors. Note the different concentration scale for the weak inhibitor MNPG and that the titration of GM1 was discontinued above 30 μM because of micelle-forming effects. (f) Overlay of displacement curves for all three inhibitors on a logarithmic scale using the consensus *K*_D_ = 150 nM for F-GM1 and *K*_I_ values of 50 nM, 0.3 nM, and 200 μM for GM1, GM1-PS, and MNPG, respectively.^30,32,34^

This approach was leveraged in a competition-based format in order to assess the affinity of F-GM1 compared with several previously characterized cholera toxin ligands (Figure 4).^27^ In this study, F-GM1 was employed as a so-called “reporter-ligand” as it showed a clear binding signature: because of strong *T*_2_-relaxation effects, its ^19^F signals were strongly broadened in the presence of equimolar amounts of CTB. Displacement of F-GM1 from CTB under increasing concentrations of different inhibitors was then monitored as a function of the signal of free F-GM1 that appeared. The IC_50_ values obtained in this assay allowed the relative affinities of the examined ligands to be established (Figure 4). Native GM1 and GM1 pentasaccharide (GM1-PS) have a 3-fold and >10-fold higher affinity than F-GM1, respectively. This confirms that the OH group at C2 of the terminal galactose is an important H-bond donor/acceptor in the CTB_5_–GM1 interaction and also indicates that the overall binding mode of F-GM1 is largely preserved.

In order to employ F-GM1 as a tool in chemical biology and for biophysical characterization of newly developed CTB_5_ inhibitors, it would be highly desirable to determine its affinity. Using literature *K*_D_ values of the tested inhibitors, the affinity of the reporter F-GM1 can be calculated according to published procedures.^17e,28^ (Note that the popular simplified Cheng-Prusoff treatment^29^ cannot be applied here because of the elevated protein concentration employed, such that the explicit equations were used). In case of GM1 and its derivatives, however, a confusing number of affinities deviating by orders of magnitude have been published.^30−34^ This is partly due to the complexity introduced by the 5-fold symmetry of CTB_5_, the heterogeneity of the assays employed, and the micelle-forming properties of GM1. Indeed, micelle formation of GM1 with its prominent lipid tail was also observed at concentrations above 30 μM under our experimental conditions.

**Figure 5:**
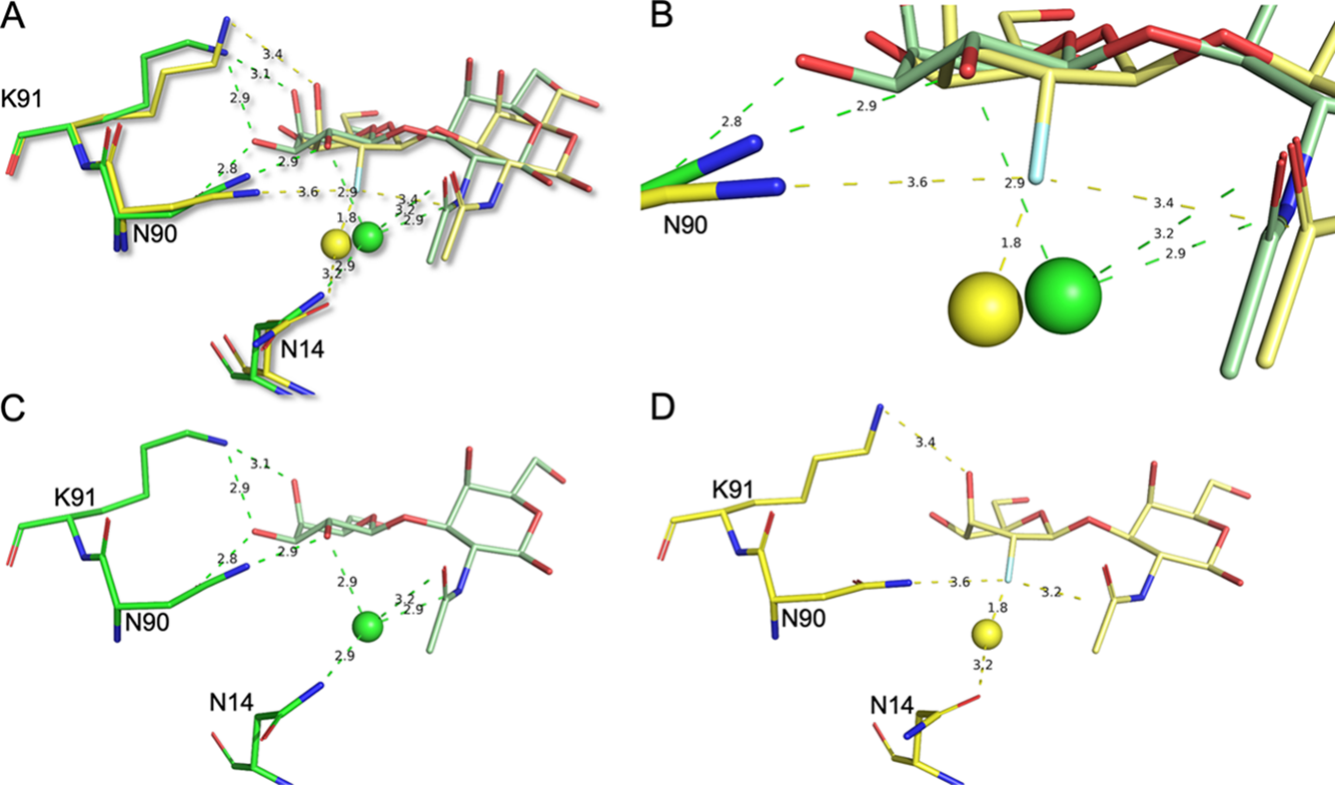
Comparison of GM1 and F-GM1 binding to CTB_5_. (A) Superposition of the published CTB_5_-GM1 (green; PDB ID: 2CHB) and the CTB_5_–F-GM1 (yellow; PDB ID: 9EWF) complex structures. Key CTB_5_ residues are shown as sticks, water molecules are shown as spheres (yellow CTB_5_-GM1 and green CTB_5_–F-GMGM), and the carbohydrate ligands are shown as sticks in colors corresponding to their protein. Hydrogen bonds are shown as dashed lines in corresponding color with distances given in Å. (B) Close-up of (A). (C) As (A), but only the CTB_5_-GM1 structure is shown to highlight the chair conformation of the terminal galactose. (D) As (A), but only the CTB_5_–F-GM1 structure is shown to highlight the conformation of the terminal fluorinated galactose.

To resolve the heterogeneity of literature affinities, a simultaneous fit of all measured inhibitors was performed, and only one set of published inhibitor affinities was in agreement with our experimental data, thereby yielding a consensus value of ∼150 nM for the *K*_D_ of F-GM1 (Figure 4). Thus, F-GM1 enabled the establishment of an NMR binding assay in a straightforward manner, which can now be used to characterize CTB_5_ antagonists and guide medicinal chemistry efforts. All solution-phase data obtained indicated that the general binding mode of GM1 was highly conserved in F-GM1 with the exception of the aforementioned three crucial hydrogen bonds (please see Figure 1). To acquire atomic resolution insights into the binding mode between CTB_5_ and F-GM1, we attempted to determine the high-resolution crystal structure of this complex. Unexpectedly, the addition of F-GM1, but not GM1, to CTB_5_ led to severe protein precipitation at protein concentrations required for crystallography. This problem was circumvented by crystallizing the protein with a disaccharide and then exchanging the ligands *in situ* by carefully timed soaking with high concentrations of F-GM1 (details can be found in the Supporting Information). This approach allowed us to collect a high-quality data set to 2.10 Å resolution (Table S2). The asymmetric unit contained two CTB_5_ pentamers whose overall structure was virtually unchanged compared with that of CTB_5_ in complex with GM1 (PDB ID: 2CHB; C_α_ RMSD of merely 0.4 Å over all C_α_ atoms of the pentamer, Figure S8). Crucially, F-GM1 was found to be bound in the same binding mode as GM1 with one crucial difference in the terminal fluorinated galactose unit. The OH → F substitution at C2 of the terminal galactose led to a change in conformation (Figure 5B,D).

Upon closer inspection of the crystal structure, key noncovalent interactions with the fluorine atom enable this change in the pyranose ring conformation to be placed on a structural foundation. The quasi-axial arrangement of the C(sp^3^)–F bond of the d-galactose enables a Dunitz–Diederich-type interaction^15a,35^ with the adjacent GalNAc C(sp^2^)=O (3.4 Å, Figure 5B). Moreover, this permits an interaction with the side chain residue N90 of the protein (3.6 Å, Figure 5B). These interactions abolish the formation of the important hydrogen bonds (CTB_5_, Asn14, and Asn90; Figure 5C versus Figure 5D) and confirm the working hypothesis.

## Conclusions

Motivated by the ubiquity of glycan-specific protein recognition in biology and medicine, the strongest interaction known, between GM1 and CTB_5_, has been interrogated through site-selective molecular editing of the ligand with fluorine. The importance of a key hydrogen bonding interaction between the terminal GM1 galactose, CTB_5_ (Asn90 and Asn14), and a water molecule has long been implicated as a major contributory factor in the remarkable affinity of this complex. However, to the best of our knowledge, direct editing approaches to investigate this supposition remain conspicuous in their absence. A multistep, stereocontrolled synthesis of F-GM1 (19 steps, longest linear sequence) has enabled the impact of H-bond deletion on binding affinity with CTB_5_ to be probed by NMR and protein crystallography. Competition studies have revealed that fluorination of the terminal galactose weakens binding to cholera toxin compared with the natural ligand GM1 but that the binding strength is of the same order of magnitude (about 3 times weaker): this suggests that all other important interactions are preserved. Indeed, the CTB_5_ − F-GM1 crystal structure reveals the binding mode of F-GM1 to be largely unchanged, except for the fluorinated, terminal galactose unit, and confirms that only the intended hydrogen bonds between the protein and ligand have been eliminated. These data confirm the bioisosteric nature of the introduced F and further underscore the utility of OH to F bioisosteric replacement in glycobiology. It is envisaged that this study will highlight the potential of fluorinated gangliosides in structural delineation and the obvious implications this has for probing glycan signal transduction, small molecule drug discovery, and vaccine development.

## Supporting Information Available

Supplementary tables and figures, full experimental procedures and analytical data (^1^H, ^13^C, and ^19^F spectral data) for new compounds, NMR experiments and crystallography data (PDF)
Full wwPDB X-ray structure validation report for 9EWF (PDF)

## Abbreviations

Asn: asparagine
CAN: ceric ammonium nitrate
CTB: cholera toxin subunit B
GM1/GM3: monosialotetrahexosylganglioside 1/3
GM1-PS: Monosialotetrahexosylganglioside 1 pentasaccharide
Glu: glutamic acid
Lys: lysine
MNPG: *meta*-mononitrophenyl-α-galactoside
NMR: nuclear magnetic resonance
Troc: 2,2,2-tricholoroethoxycarbonyl chloride
VT: variable temperature
