# Supplementary material for: Probing the Origin of Affinity in the GM1-Cholera Toxin Complex through Site-Selective Editing with Fluorine
Source: ACS Cent Sci. 2024 Jul 12;10(8):1481–9. doi: 10.1021/acscentsci.4c00622 (PMC11363330; doi:10.1021/acscentsci.4c00622)

Supporting Information

Probing the Origin of Affinity in the GM1-Cholera Toxin Complex through Site-selective Editing with Fluorine

Christina Jordan,[‡a] Taiki Hayashi,[‡a] Arnelle Löbbert,[b] Jingran Fan,[c] Charlotte S. Teschers,[a] Kathrin Siebold,[a] Marialuisa Aufiero,[a] Felix Pape,[a] Emma Campbell,[a] Alexander Axer,[a] Kathrin Bussmann,[a] Klaus Bergander,[a] Jesko Köhnke,[*c] Alvar D. Gossert,[*b] and Ryan Gilmour[*a]

[a] Institute for Organic Chemistry, University of Münster, 48149 Münster, Germany
[b] Department of Biology, ETH Zürich, 8093 Zürich, Switzerland
[c] Institut für Lebensmittelchemie, Leibniz Universität Hannover, 30167 Hannover, Germany

Table of Contents

General Methods	S2
Experimental Procedures and Characterization Data	S3
Variable Temperature (VT) Experiments	S20
NMR Experimental Data (Competition Experiments)	S24
Experimental Procedures Crystallization	S24
References	S28
NMR Spectra	S29

General Methods

All reactions were performed under an atmosphere of argon in dried glassware, except when using aqueous reagents. All chemicals were reagent grade and used as supplied unless stated otherwise. Anhydrous solvents were dried by a Grubbs purification system including columns packed with molecular sieves and aluminium oxide. Solvents for extractions and chromatography were technical grade and were distilled on a rotary evaporator prior to usage. Extracts were dried over technical grade Na_2SO_4 or $MgSO_4$. Analytical thin layer chromatography (TLC) was performed on pre-coated *Merck* silica gel 60 F_{254} plates (0.25 mm) and visualized by UV or CAM stain. Column chromatography was carried out on *Fluka* silica gel 60 (230-400 mesh). Concentration *in vacuo* was performed at ca. 10 mbar and 45 °C, drying at 10^{-2} mbar and room temperature. ^{1}H NMR, ^{13}C NMR and ^{19}F NMR spectra were recorded on a *Bruker AV 400 MHz*, *Agilent DD2 500* and an *Agilent DD2 600* spectrometer by the NMR service of the Organisch-Chemisches Institut, Universität Münster. Chemical shifts (δ) are reported in ppm relative to the solvent residual peak (7.26 and 77.16 ppm for $CDCl_3$, 5.32 and 54.00 ppm for CD_2Cl_2, 3.31 and 49.00 ppm for CD_3OD). The multiplicities are reported as: s = singlet, d = doublet, t = triplet, q = quartet, m = multiplet, br = broad. Melting points were measured in open capillaries on a *Büchi B540* melting point apparatus. IR spectra of the neat compounds were measured on a *Perkin-Elmer Spectrum 100 FTIR* spectrometer and are reported in wavenumbers (cm^{-1}). Optical rotations were obtained using a *JASCO P-2000* polarimeter. Mass spectra (ESI-MS and MALDI-TOF-MS) were recorded by the MS service at the Organisch-Chemisches Institut, Universität Münster. Preparative HPLC purification was carried out using a *Büchi Pure C-850 Flash/Prep* system on an *Agilent ZORBAX Eclipse Plus C18* preparative LC column (21.2 x 150 mm, 5 µm).

Experimental Procedures and Characterization Data

Compound **11**

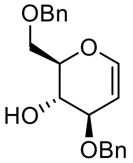

To a solution of tri-*O*-acetyl-D-glucal **10** (5.98 g, 22.0 mmol, 1.0 eq.) in MeOH (40 mL) was added NaOMe (0.44 M in MeOH, 2.5 mL, 1.1 mmol, 5 mol%) at room temperature. After stirring for 20 min, the mixture was concentrated *in vacuo.* The mixture of this crude material and $(Bu_3Sn)_2O$ (12.3 mL, 24.1 mmol, 1.1 eq.) in toluene (100 mL) was refluxed for 20 h with a Dean-Stark trap. After cooling the mixture below boiling temperature, BnBr (8.4 mL, 71 mmol, 3.2 eq.) and TBAB (14.2 g, 44.0 mmol, 2.0 eq.) were added. After further refluxing for 16 h, the mixture was allowed to cool to room temperature. The reaction was quenched by adding water. The mixture was extracted with EtOAc (×3). The combined organic layers were washed with brine, dried (Na_2SO_4), and concentrated *in vacuo*. The residue was purified by successive column chromatography (SiO_2, cHex:EtOAc = 9:1 to 85:15) and (SiO_2, CH_2Cl_2:Et_2O = 98:2 to 9:1) to afford pure fraction of **11** (1.29 g, 3.95 mmol, 18 %) as a colorless oil and a mixed fraction of **11** (containing 10% benzyl alcohol, 3.73 g, 55 %; total yield 73%).

11: $[\alpha]_D^{28}$ -36.4 (c 1.00, $CHCl_3$), lit. $[\alpha]_D^{26}$ -35 (c 1.2, $CHCl_3$)[1].; ^1H NMR (400 MHz, CD_2Cl_2) δ 7.40–7.24 (10H, overlapped, Bn), 6.38 (dd, *J* = 6.2, 1.6 Hz, 1H, C1), 4.86 (dd, *J* = 6.2, 2.3 Hz, 1H, C2), 4.68 (d, *J* = 11.7 Hz, 1H, O3-Bn), 4.59 (d, *J* = 11.9 Hz, 1H, O6-Bn), 4.58 (d, *J* = 11.7 Hz, 1H, O3-Bn), 4.54 (d, *J* = 11.9 Hz, 1H, O6-Bn), 4.06 (m, 1H, C3), 3.99–3.89 (2H, overlapped, C4+C5), 3.81 (dd, *J* = 10.9, 4.6 Hz, 1H, C6), 3.77 (dd, *J* = 10.9, 3.3Hz, 1H, C6), 2.56 (d, *J* = 3.4 Hz, 1H, 4-OH); ^{13}C NMR (101 MHz, CD_2Cl_2) δ 145.0 (C1), [139.2, 138.7, 128.9 (2C), 128.30, 128.26, 128.22, 128.15] (Bn), 100.6 (C2), 77.6 (C5), 76.8 (C3), 74.0 (O6-Bn), 71.2 (O3-Bn), 69.8 (C6), 69.4 (C4); ESI-MS found: 349.1427 calcd: 349.1410 for $C_{20}H_{22}NaO_4$ $[M+Na]^+$; IR: 3423, 3087, 3063, 3030, 2918, 2863, 1645, 1607, 1586, 1496, 1454, 1388, 1362, 1329, 1234, 1177, 1090, 1058, 1028, 911, 852, 818, 737, 698, 676.

Compounds **12** (Gluco-type) and **13** (Manno-type)

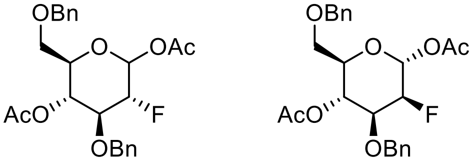

To a solution of glucal **11** (1.26 g, 3.86 mmol, 1.0 eq.) in acetone/water (5/1, 36 mL) was added Selectflour® (1.64 g, 4.63 mmol, 1.2 eq.) at room temperature. After stirring for 1 h, the reaction was quench by saturated aqueous $NaHCO_3$ at 0 °C. The mixture was extracted with CH_2Cl_2 (×3). The combined organic layers were dried (Na_2SO_4) and concentrated *in vacuo*. Chromatographic purification (SiO_2, cHex:EtOAc = 7:3 to 65:35) afforded the crude lactol as a mixture of diastereomers. To a solution of this crude material in CH_2Cl_2 (8 mL) was added successively Ac_2O (0.67 mL, 7.09 mmol, 3.0 eq.), pyridine (0.95 mL, 11.8 mmol, 5.0 eq.) and DMAP (14.7 mg, 0.120 mmol, 5 mol%) at room temperature. After stirring for 1 h, the reaction was quench by saturated aqueous $NaHCO_3$ at 0 °C. The mixture was extracted with EtOAc (×3). The combined organic layers were washed with aqueous 1 M HCl (×2), saturated aqueous $NaHCO_3$ and brine successively, dried (Na_2SO_4), and concentrated *in vacuo*. Chromatographic purification (SiO_2, cHex:EtOAc = 85:15 to 8:2) afforded **12** (521 mg, 1.17 mmol, 30 %, α/β = 55/45) as a colorless oil and **13** (353 mg, 0.791 mmol, 20 %, α-anomer) as a colorless oil.

12 (α/β = 55/45, determined by ^{19}F NMR): ^{1}H NMR (599 MHz, CD_2Cl_2, The signals for β-anomer are marked with an asterisk) δ 7.39–7.26 (10H+10H*, overlapped, Bn+Bn*), 6.34 (d, J = 3.9 Hz, 1H, C1), 5.74* (dd, J = 8.1 Hz and $^{3}J_{FH}$ = 3.5 Hz, 1H, C1), 5.11 (dd, J = 10.2, 9.4 Hz, 1H, C4), 5.07* (dd, J = 10.0, 9.3 Hz, 1H, C4), 4.82 (d, J = 11.6 Hz, 1H+1H*, O3-Bn+O3-Bn*), 4.68 (ddd, $^{2}J_{FH}$ = 48.6 Hz and J = 9.2, 3.9 Hz, 1H, C2), 4.63* (d, J = 11.6 Hz, 1H, O3-Bn), 4.62 (d, J = 11.6 Hz, 1H, O3-Bn), 4.487* (ddd, $^{2}J_{FH}$ = 51.1 Hz and J = 8.7, 8.1 Hz, 1H, C2), 4.486* (d, J = 11.7 Hz, 1H, O6-Bn), 4.48 (d, J = 11.6 Hz, 1H, O6-Bn), 4.452* (d, J = 11.7 Hz, 1H, O6-Bn), 4.450 (d, J = 11.6 Hz, 1H, O6-Bn), 4.02 (ddd, $^{3}J_{FH}$ = 12.2 Hz and J = 9.4, 9.2 Hz, 1H, C3), 3.94 (ddd, J = 10.2, 4.7, 3.0 Hz, 1H, C5), 3.80* (ddd, $^{3}J_{FH}$ = 14.6 Hz and J = 9.3, 8.7 Hz, 1H, C3), 3.71* (ddd, J = 10.0, 4.7, 2.8 Hz, 1H, C5), 3.56* (dd, J = 11.0, 2.8 Hz, 1H, C6), 3.53 (dd, J = 11.0, 3.0 Hz, 1H, C6), 3.48 (dd, J = 11.0, 4.7 Hz, 1H+1H*, C6+C6*), 2.17 (s, 3H, Ac), 2.16* (s, 3H, Ac), 1.93 (s, 3H, Ac), 1.92* (s, 3H, Ac); ^{13}C NMR (151 MHz, CD_2Cl_2, The discernible signals for β-anomer are marked with an asterisk) δ [169.82*, 169.77, 169.7*, 169.5] (Ac+Ac*), [138.7, 138.5, 138.44, 138.43, 128.92, 128.90, 128.86, 128.49, 128.47, 128.46, 128.4, 128.33, 128.26, 128.2] (Bn+Bn*, two signals are missing, possibly due to overlapping), 92.4* (d, $^{1}J_{FC}$ = 188.0 Hz, C2), 91.9* (d, $^{2}J_{FC}$ = 24.8 Hz, C1), 90.3 (d, $^{1}J_{FC}$ = 191.4 Hz, C2), 89.4 (d, $^{2}J_{FC}$ = 22.7 Hz, C1), 80.6* (d, $^{2}J_{FC}$ = 17.6 Hz, C3), 78.0 (d, $^{2}J_{FC}$ = 17.7 Hz, C3), 75.0 (d, $^{4}J_{FC}$ = 2.4 Hz, O3-Bn), 74.9* (d, $^{4}J_{FC}$ = 2.4 Hz, O3-Bn), 74.8* (d, $^{4}J_{FC}$ = 1.1 Hz, C5), 74.1 (O6-Bn), 74.0* (O6-Bn), 71.7 (d, $^{4}J_{FC}$ = 1.2 Hz, C5), 69.6* (d, $^{3}J_{FC}$ = 9.5 Hz, C4), 69.5 (d, $^{3}J_{FC}$ = 9.7 Hz, C4), 69.2 (d, $^{5}J_{FC}$ = 0.7 Hz, C6), 69.0* (d, $^{5}J_{FC}$ = 0.6 Hz, C6), [21.28, 21.25*, 21.2, 21.1*] (Ac+Ac*); ^{19}F NMR (564 MHz, CD_2Cl_2, The signal for β-anomer is marked with an asterisk) δ -198.5* (ddd, $^{2}J_{FH}$ = 51.1 Hz and $^{3}J_{FH}$ = 14.6, 3.5 Hz), -200.6 (dd, $^{2}J_{FH}$ = 48.6 Hz and $^{3}J_{FH}$ = 12.2 Hz); ESI-MS found: 469.1646 calcd: 469.1633 for $C_{24}H_{27}FNaO_7$ $[M+Na]^+$; IR: 3087, 3065, 3031, 2873, 2320, 2104, 1754, 1497, 1455, 1433, 1367, 1214, 1137, 1080, 1061, 1033, 932, 857, 821, 738, 699.

13: $[\alpha]_D^{26}$ +53.5 (c 1.00, MeCN); ^{1}H NMR (599 MHz, CD_2Cl_2) δ 7.39–7.26 (10H, overlapped, Bn), 6.22 (dd, $^{3}J_{FH}$ = 6.6 Hz and J = 2.3 Hz, 1H, C1), 5.32 (ddd, J = 10.1, 9.8 Hz and $^{4}J_{FH}$ = 0.7 Hz, 1H, C4), 4.73 (ddd, $^{2}J_{FH}$ = 48.5 Hz and J = 2.5, 2.3 Hz, 1H, C2), 4.71 (d, J = 11.9 Hz, 1H, O3-Bn), 4.59 (d, J = 11.9 Hz, 1H, O3-Bn), 4.51 (d, J = 11.8 Hz, 1H, O6-Bn), 4.48 (d, J = 11.8 Hz, 1H, O6-Bn), 3.92 (ddd, J = 10.1, 4.4, 3.6 Hz, 1H, C5), 3.84 (ddd, $^{2}J_{FH}$ = 28.5 Hz and J = 9.8, 2.5 Hz, 1H, C3), 3.540 (d, J = 3.6 Hz, 1H, C6), 3.539 (d, J = 4.4 Hz, 1H, C6), 2.10 (s, 3H, Ac), 1.96 (s, 3H, Ac); ^{13}C NMR (151 MHz, CD_2Cl_2) δ [169.93, 168.88] (Ac), [138.60, 138.22, 129.01, 128.85, 128.51, 128.43, 128.28, 128.18] (Bn), 91.0 (d, $^{2}J_{FC}$ = 31.2 Hz, C1), 86.2 (d, $^{1}J_{FC}$ = 180.2 Hz, C2), 75.7 (d, $^{2}J_{FC}$ = 17.3 Hz, C3), 74.1 (O6-Bn), 72.9 (C5), 72.6 (O3-Bn), 69.5 (C6), 67.8 (d, $^{3}J_{FC}$ = 1.7 Hz, C4), [21.22, 21.20] (Ac); ^{19}F NMR (564 MHz, CD_2Cl_2) δ -204.6 (ddd, $^{2}J_{FH}$ = 48.5 Hz, $^{3}J_{FH}$ = 28.5, 6.6 Hz and $^{4}J_{FH}$ = 0.7 Hz); ESI-MS found: 469.1636 calcd: 469.1633 for $C_{24}H_{27}FNaO_7$ $[M+Na]^+$; IR: 3065, 3031, 2868, 2325, 2108, 1748, 1497, 1455, 1433, 1372, 1314, 1300, 1218, 1150, 1106, 1057, 1046, 1020, 1001, 972, 894, 848, 793, 739, 699, 686.

Compound **S1**

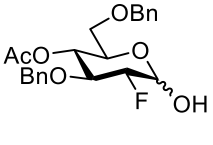

To a solution of **12** (490 mg, 1.10 mmol, 1.0 eq.) in THF (3.7 mL) was added $H_2NNH_2H{\cdot}OAc$ (13 3mg, 1.44 mmol, 1. eq.) at room temperature. After stirring for 18.5 h, water was added to the mixture at 0 °C. The mixture was extracted with EtOAc (×3). The combined organic layers were washed with water, saturated aqueous $NaHCO_3$ and brine, dried (Na_2SO_4), and concentrated *in vacuo*. Chromatographic purification (SiO_2, cHex:EtOAc = 8:2 to 75:25) afforded **S1** (313 mg, 0.774 mmol, 70 %, α/β = 75/25) as a colorless oil.

S1(anomeric mixture α/β = 75/25 in $CDCl_3$ determined by ^{19}F NMR): $[\alpha]_D^{24}$ +30.0 (c 1.00, $CHCl_3$); ^{1}H NMR (599 MHz, $CDCl_3$, The signals for β-anomer are marked with an asterisk) δ 7.36–7.25

(10H+10H*, overlapped, Bn+Bn*), 5.40 (d, J = 3.7 Hz, 1H, C1), 4.98* (dd, J = 9.6, 9.0 Hz, 1H, C4), 4.95 (dd, J = 10.4, 9.8 Hz, 1H, C4), 4.844 (d, J = 11.8 Hz, 1H, O3-Bn), 4.839* (d, J = 11.8 Hz, 1H, O3-Bn), 4.75* (dd, J = 7.6 Hz and $^{3}J_{FH}$ = 3.1 Hz, 1H, C1), 4.60 (d, J = 11.8 Hz, 1H+1H*, O3-Bn+O3-Bn*), 4.53 (ddd, $^{2}J_{FH}$ = 49.4 Hz and J = 9.2, 3.7 Hz, 1H, C2), 4.52 (d, J = 12.1 Hz, 1H+1H*, O6-Bn+O6-Bn*), 4.50 (d, J = 12.1 Hz, 1H+1H*, O6-Bn+O6-Bn*), 4.32* (ddd, $^{2}J_{FH}$ = 50.9 Hz and J = 8.7, 7.6 Hz, 1H, C2), 4.14 (ddd, J = 10.4, 5.6, 3.6 Hz, 1H, C5), 4.04 (ddd, $^{3}J_{FH}$ = 12.2 Hz and J = 9.8, 9.2 Hz, 1H, C3), 3.71 (br, 1H+1H*, 1-OH+1-OH*), 3.67* (ddd, $^{3}J_{FH}$ = 14.5 Hz and J = 9.0, 8.7 Hz, 1H, C3), 3.58* (m, 1H, C5), 3.52–3.43 (2H+2H*, overlapped, C6+C6*), 1.884* (s, 3H, Ac), 1.879 (s, 3H, Ac); ^{13}C NMR (151 MHz, $CDCl_3$, The signals for β-anomer are marked with an asterisk) δ 169.8 (Ac), 169.7* (Ac), [138.3, 137.9*, 137.53, 137.46*, 128.6*, 128.53, 128.50, 128.22, 128.21*, 128.02*, 127.99*, 127.97, 127.9, 127.8] (Bn+Bn*, two signals are missing, possibly due to overlapping), 94.7* (d, $^{2}J_{FC}$ = 23.7 Hz, C1), 94.1* (d, $^{1}J_{FC}$ = 186.9 Hz, C2), 91.3 (d, $^{1}J_{FC}$ = 190.5 Hz, C2), 90.5 (d, $^{2}J_{FC}$ = 21.3 Hz, C1), 79.8* (d, $^{2}J_{FC}$ = 18.0 Hz, C3), 77.1 (d, $^{2}J_{FC}$ = 17.7 Hz, C3), 74.6 (d, $^{4}J_{FC}$ = 2.6 Hz, O3-Bn), 74.3* (d, $^{4}J_{FC}$ = 2.6 Hz, O3-Bn), 73.8* (O6-Bn), 73.7 (O6-Bn), 73.6* (d, $^{4}J_{FC}$ = 0.7 Hz, C5), 69.93 (d, $^{3}J_{FC}$ = 9.1 Hz, C4), 69.89* (d, $^{3}J_{FC}$ = 9.2 Hz, C4), 69.14* (C6), 69.08 (C6), 68.7 (d, $^{4}J_{FC}$ = 0.8 Hz, C5), 20.87 (Ac), 20.86* (Ac); ^{19}F NMR (564 MHz, $CDCl_3$, The signal for β-anomer is marked with an asterisk) δ -196.2* (ddd, $^{2}J_{FH}$ = 50.9 Hz and $^{3}J_{FH}$ = 14.5, 3.1 Hz), -198.2 (dd, $^{2}J_{FH}$ = 49.4 Hz and $^{3}J_{FH}$ = 12.2 Hz); ESI-MS found: 427.1539 calcd: 427.1527 for $C_{22}H_{25}FNaO_6$ $[M+Na]^+$; IR: 3409, 3064, 3030, 2913, 2869, 1743, 1497, 1454, 1368, 1331, 1229, 1155, 1131, 1052, 1027, 984, 937, 912, 855, 823, 742, 699, 667.

Compounds **14** (α-anomer) and **S2** (β-anomer)

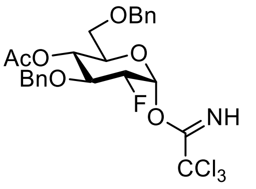

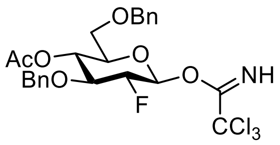

To a solution of **S1** (291 mg, 0.720 mg, 1.0 eq.) in CH_2Cl_2 (3 mL) was added successively Cl_3CCN (0.73 mL, 7.2 mmol, 10 eq.) and DBU (3 drops) at room temperature. After stirring for 2 h, the mixture was concentrated *in vacuo*. Chromatographic purification (SiO_2, cHex:EtOAc = 9:1 to 85:15) afforded **14** (336 mg, 0.612 mmol, 85 %) as a colorless oil and **S2** (28.6 mg, 0.0521 mmol, 7 %) as a colorless oil.

14: $[\alpha]_D^{24}$ +78.6 (c 1.00, MeCN); ^{1}H NMR (599 MHz, CD_2Cl_2) δ 8.80 (s, 1H, NH), 7.37–7.26 (10H, overlapped, Bn), 6.56 (d, J = 3.8 Hz, 1H, C1), 5.16 (ddd, J = 10.3, 9.4 Hz and $^{4}J_{FH}$ = 0.9 Hz, 1H, C4), 4.83 (d, J = 11.6 Hz, 1H, O3-Bn), 4.75 (ddd, $^{2}J_{FH}$ = 48.3 Hz and J = 9.3, 3.8 Hz, 1H, C2), 4.64 (d, J = 11.6 Hz, 1H, O3-Bn), 4.48 (d, J = 11.7 Hz, 1H, O6-Bn), 4.45 (d, J = 11.7 Hz, 1H, O6-Bn), 4.10 (ddd, $^{3}J_{FH}$ = 11.8 Hz and J = 9.4, 9.3 Hz, 1H, C3), 4.06 (ddd, J = 10.3, 4.5, 2.8 Hz, 1H, C5), 3.55 (dd, J = 11.2, 2.8 Hz, 1H, C6), 3.49 (dd, J = 11.2, 4.5 Hz, 1H, C6), 1.94 (s, 3H, Ac); ^{13}C NMR (151 MHz, CD_2Cl_2) δ 169.8 (Ac), 161.2 (C=NH), [138.51, 138.50, 128.9, 128.8, 128.6, 128.41, 128.39, 128.2] (Bn), 93.7 (d, $^{2}J_{FC}$ = 22.8 Hz, C1), 91.4 (CCl_3), 90.6 (d, $^{1}J_{FC}$ = 192.5 Hz, C2), 77.6 (d, $^{2}J_{FC}$ = 17.7 Hz, C3), 75.1 (d, $^{4}J_{FC}$ = 2.6 Hz, O3-Bn), 74.0 (O6-Bn), 72.2 (d, $^{4}J_{FC}$ = 1.0 Hz, C5), 69.2 (d, $^{3}J_{FC}$ = 9.4 Hz, C4), 68.9 (d, J_{FC} = 0.7 Hz, C6), 21.2 (Ac); ^{19}F NMR (564 MHz, CD_2Cl_2) δ -200.8 (ddd, $^{2}J_{FH}$ = 48.3 Hz and $^{3}J_{FH}$ = 11.8 Hz and $^{4}J_{FH}$ = 0.9 Hz); ESI-MS found: 570.0629 calcd: 570.0624 for $C_{24}H_{25}Cl_3FNNaO_6$ $[M+Na]^+$; IR: 3339, 3089, 3064, 3030, 2915, 2867, 2322, 2097, 1745, 1675, 1608, 1497, 1454, 1364, 1328, 1284, 1225, 1143, 1117, 1082, 1073, 1030, 967, 919, 856, 833, 795, 737, 699, 681.

S2: $[\alpha]_D^{24}$ +3.0 (c 1.00, MeCN); ^{1}H NMR (599 MHz, CD_2Cl_2) δ 8.83 (s, 1H, NH), 7.39–7.26 (10H, overlapped, Bn), 5.96 (dd, *J* = 7.8 Hz and $^3J_{FH}$ = 3.7 Hz, 1H, C1), 5.10 (dd, *J* = 10.0, 9.5 Hz, 1H, C4), 4.84 (d, *J* = 11.7 Hz, 1H, O3-Bn), 4.650 (ddd, $^2J_{FH}$ = 51.0 Hz and *J* = 8.8, 7.8 Hz, 1H, C2), 4.647 (d, *J* = 11.7 Hz, 1H, O3-Bn), 4.51 (d, *J* = 11.8 Hz, 1H, O6-Bn), 4.48 (d, *J* = 11.8 Hz, 1H, O6-Bn), 3.86 (ddd, $^3J_{FH}$ = 14.8 Hz and *J* = 9.5, 8.8 Hz, 1H, C3), 3.77 (ddd, *J* = 10.0, 5.3, 2.9 Hz, 1H, C5), 3.59 (dd, *J* = 11.2, 2.9 Hz, 1H, C6), 3.53 (dd, *J* = 11.2, 5.3 Hz, 1H, C6), 1.94 (s, 3H, Ac); ^{13}C NMR (151 MHz, CD_2Cl_2) δ 169.8 (Ac), 161.5 (C=NH), [138.5, 138.4, 128.93, 128.86, 128.51, 128.48, 128.4, 128.2] (Bn), 95.9 (d, $^2J_{FC}$ = 25.2 Hz, C1), 92.4 (d, $^1J_{FC}$ = 188.4 Hz, C2), 91.1 (CCl_3), 80.3 (d, $^2J_{FC}$ = 17.9 Hz, C3), 75.1 (d, $^4J_{FC}$ = 0.7 Hz, C5), 74.8 (d, $^4J_{FC}$ = 2.3 Hz, O3-Bn), 74.0 (O6-Bn), 69.6 (d, $^3J_{FC}$ = 9.0 Hz, C4), 69.1 (C6), 21.2 (Ac); ^{19}F NMR (564 MHz, CD_2Cl_2) δ -197.8 (ddd, $^2J_{FH}$ = 51.0 Hz and $^3J_{FH}$ = 14.8, 3.7 Hz); ESI-MS found: 570.0633 calcd: 570.0624 for $C_{24}H_{25}Cl_3FNNaO_6$ $[M+Na]^+$; IR: 3331, 3064, 3030, 2875, 1751, 1677, 1497, 1454, 1366, 1290, 1224, 1061, 1028, 990, 955, 911, 893, 836, 797, 737, 698, 680.

Compounds **9** (β-anomer) and **15** (α-anomer)

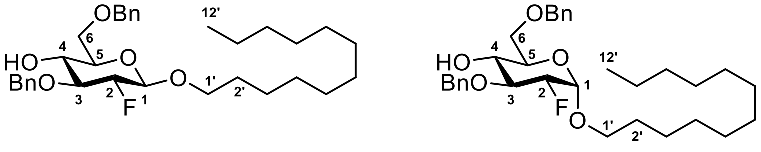

To a mixture of TCA donor **14** (1.36 g, 2.48 mmol, 1.0 eq.), 1-dodecanol (923 mg, 4.95 mmol, 2.0 eq.) and Drierite™ (500 mg) in dry CH_2Cl_2 (10 mL) was added TMSOTf (90 μL, 0.50 mmol, 0.2 eq.) at -15 °C. After stirring for 1 h, the reaction was quenched by adding NEt_3 (900 μL). The mixture was filtered through a Celite® pad, washed with EtOAc and concentrated *in vacuo*. Chromatographic purification (SiO_2, cHex:EtOAc = 95:5 to 9:1) afforded crude mixture of diastereomers. To a suspension of this crude material in MeOH (41 mL) was added K_2CO_3 (461 mg, 3.34 mmol, 1.5 eq.) at room temperature. The mixture was heated at 50 °C and stirred for 1 h. After cooling to 0 °C, the reaction was quenched by adding saturated aqueous NH_4Cl. The mixture was extracted with EtOAc (×3). The combined organic layers were washed with brine, dried (Na_2SO_4), and concentrated *in vacuo*. Chromatographic purification (SiO_2, cHex:EtOAc = 9:1 to 8:2) afforded **9** (1.02 g, 1.92 mmol, 86 %) as a colorless oil and **15** (141 mg, 0.266 mmol, 12 %) as a colorless oil.

9: $[\alpha]_D^{24}$ -35.3 (c 1.00, $CHCl_3$); ^{1}H NMR (599 MHz, $CDCl_3$) δ 7.41–7.27 (10H, overlapped, Bn), 4.93 (d, *J* = 11.3 Hz, 1H, O3-Bn), 4.71 (d, *J* = 11.3 Hz, 1H, O3-Bn), 4.61 (d, *J* = 12.1 Hz, 1H, O6-Bn), 4.57 (d, *J* = 12.1 Hz, 1H, O6-Bn), 4.48 (dd, *J* = 7.7 Hz and $^3J_{FH}$ = 2.5 Hz, 1H, C1), 4.32 (ddd, $^2J_{FH}$ = 51.2 Hz and *J* = 8.1, 7.7 Hz, 1H, C2), 3.90 (dt, *J* = 9.4, 6.7 Hz, 1H, C1'), 3.77 (dd, *J* = 10.5, 3.8 Hz, 1H, C6), 3.71 (dd, *J* = 10.5, 5.3 Hz, 1H, C6), 3.63 (dd, *J* = 9.2, 8.6 Hz, 1H, C4), 3.58 (ddd, $^3J_{FH}$ = 14.5 Hz and *J* = 8.6, 8.1 Hz, 1H, C3), 3.55 (dt, *J* = 9.4, 6.8 Hz, 1H, C1'), 3.48 (ddd, *J* = 9.2, 5.3, 3.8 Hz, 1H, C5), 2.63 (brs, 1H, 4-OH), 1.69–1.59 (2H, overlapped, C2'), 1.43–1.20 (18H, overlapped, dodecyl), 0.89 (t, *J* = 7.0 Hz, 3H, C12'); ^{13}C NMR (151 MHz, $CDCl_3$) δ [138.2, 138.0, 128.7, 128.6, 128.2, 128.1, 127.9, 127.8] (Bn), 100.6 (d, $^2J_{FC}$ = 23.1 Hz, C1), 93.1 (d, $^1J_{FC}$ = 186.3 Hz, C2), 82.7 (d, $^2J_{FC}$ = 16.7 Hz, C3), 74.6 (d, $^4J_{FC}$ = 2.8 Hz, O3-Bn), 74.3 (C5), 73.8 (O6-Bn), 71.1 (d, $^3J_{FC}$ = 8.7 Hz, C4), 70.4 (C1'), 70.1(C6), [32.1, 29.81, 29.78, 29.74, 29.72, 29.71, 29.54, 29.49, 26.0, 22.8] (dodecyl) 14.3 (C12'); ^{19}F NMR (564 MHz, $CDCl_3$) δ -196.6 (ddd, $^2J_{FH}$ = 51.2 Hz and $^3J_{FH}$ = 14.5, 2.5 Hz); ESI-MS found: 553.3301 calcd: 553.3300 for $C_{32}H_{47}FNaO_5$ $[M+Na]^+$; IR: 3452, 3063, 3031, 2923, 2853, 1497, 1455, 1365, 1311, 1270, 1207, 1173, 1125, 1077, 1058, 1029, 1008, 910, 818, 735, 697.

15: $[\alpha]_D^{26}$ +44.4 (c 1.00, $CHCl_3$); ^{1}H NMR (599 MHz, $CDCl_3$) δ 7.41–7.27 (10H, overlapped, Bn), 5.01 (d, *J* = 3.8 Hz, 1H, C1), 4.94 (d, *J* = 11.4 Hz, 1H, O3-Bn), 4.69 (d, *J* = 11.4 Hz, 1H, O3-Bn), 4.61 (d, *J* = 12.1 Hz, 1H, O6-Bn), 4.56 (d, *J* = 12.1 Hz, 1H, O6-Bn), 4.48 (ddd, $^2J_{FH}$ = 49.8 Hz and *J* = 9.3, 3.8 Hz, 1H, C2), 3.92 (ddd, $^3J_{FH}$ = 11.7 Hz and *J* = 9.3, 9.0 Hz, 1H, C3), 3.80 (ddd, *J* = 9.8, 3.9, 3.5 Hz, 1H, C5), 3.75–3.69 (2H, overlapped, C6+C1'), 3.69 (dd, *J* = 10.6, 3.5 Hz, 1H, C6), 3.66 (dd, *J* = 9.8, 9.0 Hz, 1H, C4), 3.50 (dt, *J* = 9.7, 6.6 Hz, 1H, C1'), 2.5 (brs, 1H, 4-OH), 1.70–1.60 (2H, overlapped, C2'), 1.43–1.20 (18H, overlapped, dodecyl), 0.89 (t, *J* = 7.0 Hz, 3H, C12'); ^{13}C NMR (151 MHz, $CDCl_3$) δ [138.5, 138.1, 128.7, 128.5, 128.1, 128.0, 127.8, 127.7] (Bn), 96.5 (d, $^2J_{FC}$ = 20.9 Hz, C1), 91.1 (d, $^1J_{FC}$ = 191.0 Hz, C2), 80.2 (d, $^2J_{FC}$ = 16.3 Hz, C3), 74.9 (d, $^4J_{FC}$ = 2.9 Hz, O3-Bn), 73.7 (O6-Bn), 70.4 (d, $^3J_{FC}$ = 8.2 Hz, C4), 70.0 (d, $^4J_{FC}$ = 1.2 Hz, C5), 69.5 (C6), 68.7 (C1'), [32.1, 29.81, 29.78, 29.76, 29.7, 29.55, 29.54, 29.49, 26.2, 22.8] (dodecyl) 14.3 (C12'); ^{19}F NMR (564 MHz, $CDCl_3$) δ -199.4 (dd, $^2J_{FH}$ = 49.8 Hz and $^3J_{FH}$ = 11.7 Hz); ESI-MS found: 553.3294 calcd: 553.3300 for $C_{32}H_{47}FNaO_5$ $[M+Na]^+$; IR: 3464, 3086, 3065, 3030, 2923, 2854, 1497, 1465, 1455, 1409, 1361, 1330, 1209, 1159, 1124, 1053, 1029, 911, 858, 819, 735, 697, 669.

Compound **16**

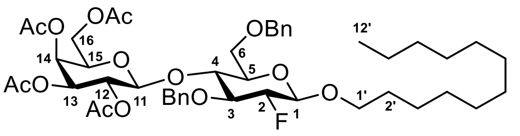

To a mixture of alcohol **9** (1.07 g, 2.02 mmol, 1.0 eq.), 2,3,4,6,-tetra-*O*-acetyl-α-D-galactopyranosyl trichloroacetimidate (**8**)[2] (1.43 g, 2.90 mmol, 1.4 eq.) and Drierite™ (651 mg) in dry CH_2Cl_2 (13 mL) was added a solution of TMSOTf (0.10 M in dry CH_2Cl_2, 3.9 mL, 0.39 mmol, 0.2 eq.) at 0 °C. After stirring for 1 h, the reaction was quenched by adding NEt_3 (0.4 mL). The mixture was filtered through a Celite® pad, washed with EtOAc and concentrated *in vacuo*. Chromatographic purification (SiO_2, cHex:EtOAc = 85:15 to 8:2) afforded lactoside **16** (1.73 g, 2.01 mmol, quant., β-only) as a colorless oil.

16: $[\alpha]_D^{26}$ -10.2 (c 1.00, $CHCl_3$); ^{1}H NMR (599 MHz, $CDCl_3$) δ 7.42–7.25 (10H, overlapped, Bn), 5.26 (dd, *J* = 3.5, 1.0 Hz, 1H, C14), 5.12 (dd, *J* = 10.4, 8.0 Hz, 1H, C12), 4.84 (s, 2H, O3-Bn), 4.83 (dd, *J* = 10.4, 3.5 Hz, 1H, C13), 4.73 (d, *J* = 12.2 Hz, 1H, O6-Bn), 4.64 (d, *J* = 8.0 Hz, 1H, C11), 4.49 (d, *J* = 12.2 Hz, 1H, O6-Bn), 4.43 (dd, *J* = 7.8 Hz and $^3J_{FH}$ = 2.9 Hz, 1H, C1), 4.30 (ddd, $^2J_{FH}$ = 50.6 Hz and *J* = 8.2, 7.8 Hz, 1H, C2), 4.04 (dd, *J* = 11.1, 7.8 Hz, 1H, C16), 3.93 (dd, *J* = 9.5, 8.9 Hz, 1H, C4), 3.91–3.85 (2H, overlapped, C16+C1'), 3.706 (d, *J* = 2.7 Hz, 2H, C6), 3.705 (ddd, $^3J_{FH}$ = 15.9 Hz and *J* = 8.9, 8.2 Hz, 1H, C3), 3.56–3.51 (2H, overlapped, C15+C1'), 3.39 (dt, *J* = 9.5, 2.7 Hz, 1H, C5), 2.11 (s, 3H, Ac), 1.98 (s, 3H, Ac), 1.97 (s, 3H, Ac), 1.95 (s, 3H, Ac), 1.67–1.60 (2H, overlapped, C2'), 1.39–1.21 (18H, overlapped, dodecyl), 0.88 (t, *J* = 7.0 Hz, 3H, C12'); ^{13}C NMR (151 MHz, $CDCl_3$) δ [170.32, 170.28, 170.2, 169.4] (Ac), [138.6, 138.0, 128.7, 128.4, 128.10, 128.08, 127.7, 127.6] (Bn), 100.7 (d, $^2J_{FC}$ = 23.1 Hz, C1), 100.4 (C11), 92.8 (d, $^1J_{FC}$ = 187.2 Hz, C2), 81.4 (d, $^2J_{FC}$ = 17.7 Hz, C3), 76.3 (d, $^3J_{FC}$ = 8.6 Hz, C4), 74.9 (C5), 74.4 (d, $^4J_{FC}$ = 2.3 Hz, O3-Bn), 73.8 (O6-Bn), 71.1 (C13), 70.6 (C15), 70.3 (C1'), 69.7 (C12), 67.7 (C6), 66.9 (C14), 60.9 (C16), [32.1, 29.80, 29.77, 29.74, 29.71, 29.70, 29.54, 29.49, 26.0, 22.8] (dodecyl), [20.9, 20.77, 20.75, 20.7] (Ac), 14.3 (C12'); ^{19}F NMR (564 MHz, $CDCl_3$) δ -195.5 (ddd, $^2J_{FH}$ = 50.6 Hz and $^3J_{FH}$ = 15.9, 2.9 Hz); ESI-MS found: 883.4228 calcd: 883.4251 for $C_{46}H_{65}FNaO_{14}$ $[M+Na]^+$; IR: 3062, 3030, 2924, 2854, 1750, 1497, 1455, 1431, 1368, 1311, 1217, 1164, 1132, 1052, 956, 909, 753, 740, 699, 677, 667.

Compound **17**

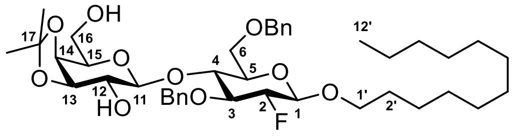

To a solution of **16** (525 mg, 0.610 mmol, 1.0 eq.) in MeOH (4.3 mL) was added a solution of NaOMe (0.44 M in MeOH, 0.28 mL, 0.12 mmol, 0.2 eq.) at room temperature. After stirring for 15 min, the mixture was neutralized with Amberlyst® [H^+] (213 mg) at 0 °C. The mixture was filtered through a Celite® pad, washed with MeOH and concentrated *in vacuo*. To a mixture of this crude material in 2,2-dimethoxypropane (6.5 mL) was added *p*TsOH·H_2O (23.3 mg, 0.122 mmol, 0.2 eq.) at room temperature. After stirring for 1.5 h, the reaction was quenched by adding NEt_3 (0.55 mL) at 0 °C and concentrated *in vacuo*. The mixture was coevaporated with toluene (×3) to remove residual NEt_3 and dissolved in EtOH/H_2O (9/1, 9 mL).[3] After refluxing for 1 h, the mixture was allowed to cool to room temperature. After dilution with CH_2Cl_2 at 0 °C, saturated aqueous $NaHCO_3$ was added to the reaction mixture. The mixture was extracted with CH_2Cl_2 (×3). The combined organic layers were dried (Na_2SO_4) and concentrated *in vacuo*. Chromatographic purification (SiO_2, cHex:EtOAc = 65:35) afforded **17** (386 mg, 0.527 mmol, 86 %) as a white solid.

17: mp 101-102 °C; $[\alpha]_D^{25}$ +7.3 (c 1.00, MeCN); ^{1}H NMR (599 MHz, CD_2Cl_2) δ 7.44–7.26 (10H, overlapped, Bn), 4.84 (d, *J* = 11.2 Hz, 1H, O3-Bn), 4.81 (d, *J* = 11.2 Hz, 1H, O3-Bn), 4.65 (d, *J* = 12.0 Hz, 1H, O6-Bn), 4.54 (d, *J* = 12.0 Hz, 1H, O6-Bn), 4.47 (dd, *J* = 7.7 Hz and $^3J_{FH}$ = 3.0 Hz, 1H, C1), 4.32 (d, *J* = 8.3 Hz, 1H, C11), 4.25 (ddd, $^2J_{FH}$ = 50.8 Hz and *J* = 8.5, 7.7 Hz, 1H, C2), 4.02 (dd, *J* = 5.5, 2.2 Hz, 1H, C14), 3.91 (dd, *J* = 9.6, 8.8 Hz, 1H, C4), 3.90 (dd, *J* = 7.3, 5.5 Hz, 1H, C13), 3.89–3.84 (2H, overlapped, C6+C1'), 3.79 (dd, *J* = 11.3, 2.1 Hz, 1H, C6), 3.72 (ddd, $^3J_{FH}$ = 15.4 Hz and *J* = 8.8, 8.5 Hz, 1H, C3), 3.67 (m, 1H, C16), 3.62–3.51 (3H, overlapped, C15+C16+C1'), 3.49 (ddd, *J* = 9.6, 3.6, 2.1 Hz, 1H, C5), 3.44 (ddd, *J* = 8.3, 7.3, 2.8 Hz, 1H, C12), 2.92 (d, *J* = 2.8 Hz, 1H, C12-OH), 2.17 (d, *J* = 7.9 Hz, 1H, C16-OH), 1.66–1.59 (2H, overlapped, C2'), 1.49 (s, 3H, Me), 1.40–1.23 (18H, overlapped, dodecyl), 1.31 (s, 3H, Me), 0.88 (t, *J* = 7.0 Hz, 3H, C12'); ^{13}C NMR (151 MHz, CD_2Cl_2) δ [139.0, 138.5, 129.0, 128.8, 128.6, 128.44, 128.37, 128.2] (Bn), 110.7 (C17), 102.4 (C11), 101.1 (d, $^2J_{FC}$ = 23.1 Hz, C1), 93.6 (d, $^1J_{FC}$ = 186.8 Hz, C2), 81.9 (d, $^2J_{FC}$ = 17.3 Hz, C3), 79.9 (C13), 76.6 (d, $^3J_{FC}$ = 8.4 Hz, C4), 75.1 (d, $^4J_{FC}$ = 2.3 Hz, O3-Bn), 75.1 (d, $^4J_{FC}$ = 1.1 Hz, C5), 74.8 (C12), 74.5 (C14), 74.3 (C15), 74.1 (O6-Bn), 70.6 (C1'), 69.0 (C6), 62.6 (C16), [32.5, 30.3, 30.22, 30.20 (2C), 30.18, 30.0, 29.9] (dodecyl), 28.5 (Me), 26.6 (Me), [26.5, 23.3] (dodecyl), 14.5 (C12'); ^{19}F NMR (564 MHz, CD_2Cl_2) δ -196.2 (ddd, $^2J_{FH}$ = 50.8 Hz and $^3J_{FH}$ = 15.4, 3.0 Hz); ESI-MS found: 755.4133 calcd: 755.4141 for $C_{41}H_{61}FNaO_{10}$ $[M+Na]^+$; IR: 3377, 3067, 3032, 2926, 2855, 1497, 1455, 1370, 1329, 1294, 1242, 1219, 1156, 1140, 1044, 1029, 964, 918, 904, 893, 872, 810, 741, 696, 658.

Compound **7**

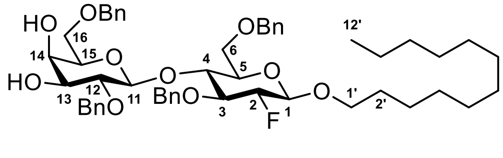

To a solution of **17** (386 mg, 0.527 mmol, 1.0 eq.) in DMF (10.5 mL) was added NaH (63.2 mg, 60% dispersion in mineral oil, 1.58 mmol, 3.0 eq.) at 0 °C. After stirring for 15 min, BnBr (0.19 mL, 1.58 mmol, 3.0 eq.) and TBAI (19.4 mg, 52.7 µmol, 0.1 eq.) were added successively. After stirring for 1.5 h at room temperature, the reaction was heated to 60 °C for 14 h. After cooling to room temperature, the reaction was diluted with aqueous LiCl (5 mL, 5%wt) at 0 °C. The mixture was extracted with Et_2O (×3). The

combined organic layers were washed with brine, dried (Na_2SO_4), and concentrated *in vacuo*. Chromatographic purification (SiO_2, cHex:EtOAc = 95:5 to 9:1) afforded the benzylated disaccharide containing traces on benzyl bromide. The mixture of this crude material in AcOH/water (4/1, 4.0 mL) was heated to 95 °C and stirred for 2 h. After cooling to 0 °C, the reaction was neutralized with saturated aqueous $NaHCO_3$. The mixture was extracted with EtOAc (×3). The combined organic layers were washed with saturated aqueous $NaHCO_3$ and brine, dried (Na_2SO_4) and concentrated *in vacuo*. Chromatographic purification (SiO_2, cHex:EtOAc = 7:3 to 65:35) afforded **7** (392 mg, 0.449 mmol, 85 %) as a colorless oil.

7: $[\alpha]_D^{25}$ +8.7 (c 1.00, $CHCl_3$); ^{1}H NMR (599 MHz, $CDCl_3$) δ 7.44–7.21 (20H, overlapped, Bn), 4.90 (d, *J* = 11.5 Hz, 1H, O3-Bn), 4.82 (d, *J* = 11.5 Hz, 1H, O3-Bn), 4.80 (d, *J* = 11.6 Hz, 1H, O12-Bn), 4.67 (d, *J* = 11.6 Hz, 1H, O12-Bn), 4.58 (d, *J* = 12.1 Hz, 1H, O6-Bn), 4.47 (d, *J* = 11.9 Hz, 1H, O16-Bn), 4.448 (dd, *J* = 7.7 Hz and $^3J_{FH}$ = 2.5 Hz, 1H, C1), 4.446 (d, *J* = 12.1 Hz, 1H, O6-Bn), 4.44 (d, *J* = 7.6 Hz, 1H, C11), 4.42 (d, *J* = 11.9 Hz, 1H, O16-Bn), 4.31 (ddd, $^2J_{FH}$ = 50.7 Hz and *J* = 8.2, 7.7 Hz, 1H, C2), 3.99 (dd, *J* = 9.4, 8.6 Hz, 1H, C4), 3.95 (brs, 1H, C14), 3.89 (dt, *J* = 9.5, 6.8 Hz, 1H, C1'), 3.80 (dd, *J* = 11.0, 4.2 Hz, 1H, C6), 3.75 (dd, *J* = 11.0, 1.9 Hz, 1H, C6), 3.70 (ddd, $^3J_{FH}$ = 15.9 Hz and *J* = 8.6, 8.2 Hz, 1H, C3), 3.66 (dd, *J* = 10.1, 6.3 Hz, 1H, C16), 3.55 (dd, *J* = 10.1, 5.0 Hz, 1H, C16), 3.53 (dt, *J* = 9.5, 6.9 Hz, 1H, C1'), 3.48–3.41 (2H, overlapped, C12+C13), 3.43 (ddd, *J* = 9.4, 4.2, 1.9 Hz, 1H, C5), 3.35 (dd, *J* = 6.3, 5.0 Hz, 1H, C15), 2.33 (br, 2H, C13-OH+C14-OH), 1.69–1.60 (2H, overlapped, C2'), 1.41–1.21 (18H, overlapped, dodecyl), 0.89 (t, *J* = 7.0 Hz, 3H, C12'); ^{13}C NMR (151 MHz, $CDCl_3$) δ [138.8, 138.5, 138.3, 138.0, 128.7, 128.6, 128.5, 128.3, 128.02, 128.00, 127.9, 127.77, 127.76, 127.7, 127.5] (Bn, one signal is missing, possibly due to overlapping), 102.6 (C11), 100.7 (d, $^2J_{FC}$ = 23.1 Hz, C1), 92.7 (d, $^1J_{FC}$ = 186.7 Hz, C2), 81.3 (d, $^2J_{FC}$ = 17.5 Hz, C3), 80.2 (C12), 76.2 (d, $^3J_{FC}$ = 8.2 Hz, C4), 75.3 (C5), 75.1 (O12-Bn), 74.5 (d, $^4J_{FC}$ = 1.7 Hz, O3-Bn), 73.7 (O16-Bn), 73.7 (C13), 73.4 (O6-Bn), 73.1 (C15), 70.2 (C1'), 69.1 (C14+C16), 68.2 (C6), [32.1, 29.81, 29.78, 29.76, 29.72, 29.71, 29.6, 29.5, 26.1, 22.8] (dodecyl), 14.3 (C12'); ^{19}F NMR (564 MHz, $CDCl_3$) δ -195.8 (dd, $^2J_{FH}$ = 50.7 Hz and $^3J_{FH}$ = 15.9, 2.5 Hz); ESI-MS found: 895.4777 calcd: 895.4767 for $C_{52}H_{69}FNaO_{10}$ $[M+Na]^+$; IR: 3437, 3063, 3031, 2923, 2854, 1497, 1454, 1366, 1309, 1210, 1091, 1057, 1029, 911, 736, 697.

Compound **6**[4]

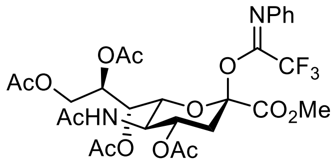

To a mixture of 4,7,8,9-tetra-*O*-acetyl-*N*-acetylneuraminic acid methyl ester (502 mg, 1.02 mmol, 1.0 eq.) and 2,2,2-trifluoro-*N*-phenylacetimidoyl chloride (426 mg, 2.05 mmol, 2.0 eq.) in dry CH_2Cl_2 (5 mL) were successively added EtN^iPr_2 (0.53 mL, 3.03 mmol, 3.0 eq.) and DMAP (25.0 mg, 0.205 mmol, 0.2 eq.) at room temperature. After stirring for 1 h, the mixture was concentrated *in vacuo*. Chromatographic purification (SiO_2, cHex:EtOAc = 4:6 to 3:7) afforded **6** (401 mg, 0.605 mmol, 59 %, β-anomer) as a white solid.

6: mp 132-136 °C (decomp.); $[\alpha]_D^{26}$ -34.3 (c 1.00, MeCN); ^{1}H NMR (599 MHz, CD_2Cl_2) δ 7.29 (m, 2H, *m*-NPh), 7.11 (m, 1H, *p*-NPh), 6.76 (m, 2H, *o*-NPh), 5.48 (d, *J* = 9.7 Hz, 1H, NH), 5.40 (dd, *J* = 5.2, 2.2 Hz, 1H, C7), 5.26 (ddd, *J* = 11.6, 10.0, 4.9 Hz, 1H, C4), 5.15 (ddd, *J* = 7.1, 5.2, 2.6 Hz, 1H, C8), 4.46 (dd, *J* = 12.3, 2.6 Hz, 1H, C9), 4.25 (dd, *J* = 10.7, 2.2 Hz, 1H, C6), 4.20 (ddd, *J* = 10.7, 10.0, 9.7 Hz, 1H, C5), 4.08 (dd, *J* = 12.3, 7.1 Hz, 1H, C9), 3.81 (s, 3H, Me), 2.83 (dd, *J* = 13.6, 4.9 Hz, 1H, C3), 2.17 (dd, *J* = 13.6, 11.6 Hz, 1H, C3), 2.12 (s, 3H, Ac), 2.05 (s, 3H, Ac), 2.04 (s, 3H, Ac), 1.87 (s, 3H, Ac), 1.80 (s, 3H, Ac); ^{13}C NMR (151 MHz, CD_2Cl_2) δ [171.2, 170.8, 170.7, 170.53, 170.51] (Ac), 165.9 (C1), 143.4

(*ipso*-NPh), 141.8 (q, $^2J_{FC}$ = 36.2 Hz, C=N), 129.4 (*m*-NPh), 125.2 (*p*-NPh), 119.7 (q, J_{FC} = 1.5 Hz, *o*-NPh), 116.2 (q, $^1J_{FC}$ = 287.3 Hz, CF_3) 100.6 (C2), 74.1 (C6), 71.5 (C8), 68.7 (C4), 68.6 (C7), 62.8 (C9), 53.6 (Me), 49.4 (C5), 36.0 (C3), [23.5, 21.21, 21.16, 21.1, 20.8] (Ac); ^{19}F NMR (564 MHz, CD_2Cl_2) δ -65.7; ESI-MS found: 685.1845 calcd: 685.1827 for $C_{28}H_{33}F_3N_2NaO_{13}$ $[M+Na]^+$; IR: 3338, 2960, 1741, 1695, 1661, 1598, 1548, 1490, 1439, 1370, 1332, 1315, 1207, 1163, 1130, 1109, 1072, 1033, 991, 945, 911, 873, 847, 823, 779, 763, 750, 696, 665.

$^{13}C\{^1H_{sel}\}$ (δ^1H = 3.81 ppm, Me) NMR (151 MHz, $CDCl_3$)

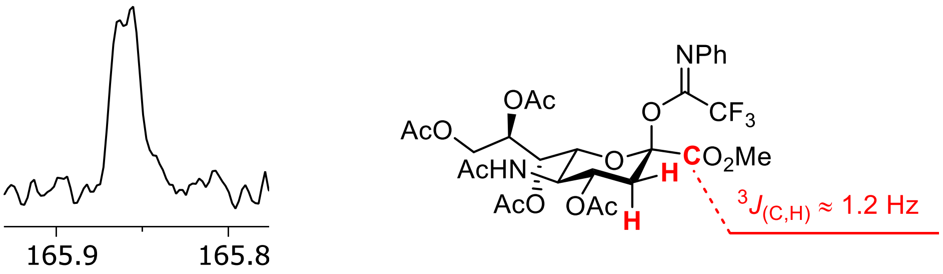

Compounds **3** (α-anomer) and **18** (β-anomer)

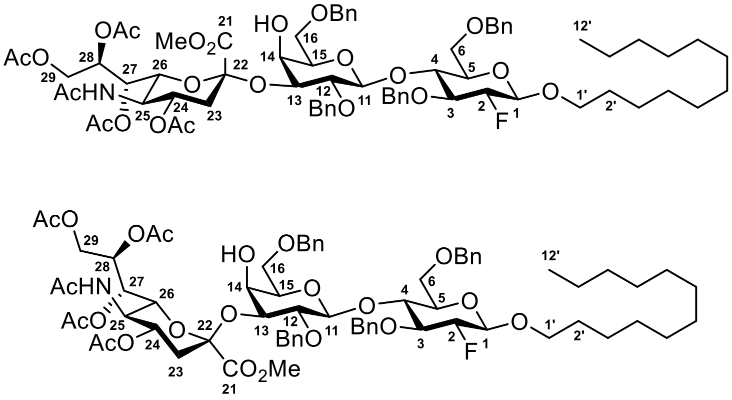

To a mixture of acceptor **7** (251 mg, 0.287 mmol, 1.0 eq.) and donor **6** (288 mg, 0.435 mmol, 1.5 eq.) in dry CH_2Cl_2/MeCN (1/1, 5.6 mL) was added dropwise a solution of TMSOTf (0.10 M in MeCN, 0.57 mL, 0.057 mmol, 0.2 eq.) at -40 °C. After stirring for 1 h at this temperature, the reaction was quenched by adding NEt_3 (0.3 mL). The mixture was concentrated *in vacuo*. Chromatographic purification (SiO_2, cHex:EtOAc = 11:4 to 3:7) afforded an anomeric mixture of **3** and **18** (207 mg, 0.154 mmol, 54% as total yield, **3**/**18** (α/β) = 86/14) as a colorless oil. Separation of this anomeric mixture was performed by Prep-HPLC (detail in below) to afford **3** and **18**, respectively.

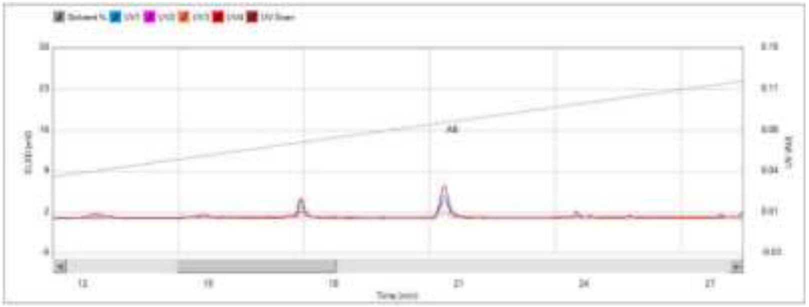

System: Büchi Pure C-850 Flash/Prep
Column: Agilent ZORBAX Eclipse Plus C18 preparative LC column (21.2 x 150 mm, 5 µm)
UV-Detection: 210nm
Flow: 15 mL/min
Solvents: MeCN/H_2O
Gradient: 5%→90% MeCN in 30 min

3: $[\alpha]_D^{28}$ -3.0 (c 1.00, $CHCl_3$); ^{1}H NMR (599 MHz, $CDCl_3$) δ 7.46–7.17 (20H, overlapped, Bn), 5.41 (ddd, *J* = 8.1, 6.1, 2.7 Hz, 1H, C28), 5.31 (dd, *J* = 8.1, 2.2 Hz, 1H, C27), 5.15 (m, 1H, NH), 4.90 (d, *J* = 11.3 Hz, 1H, O3-Bn), 4.86 (ddd, *J* = 12.2, 10.2, 4.6 Hz, 1H, C24), 4.80 (d, *J* = 11.7 Hz, 1H, O12-Bn), 4.77 (d, *J* = 11.3 Hz, 1H, O3-Bn), 4.65 (d, *J* = 11.7 Hz, 1H, O12-Bn), 4.58 (d, *J* = 7.7 Hz, 1H, C11), 4.47 (d, *J* = 12.6 Hz, 1H, O6-Bn), 4.450 (d, *J* = 12.6 Hz, 1H, O6-Bn), 4.445 (d, *J* = 11.9 Hz, 1H, O16-Bn), 4.41 (dd, *J* = 7.8 Hz and $^3J_{FH}$ = 2.9 Hz, 1H, C1), 4.37 (d, *J* = 11.9 Hz, 1H, O16-Bn), 4.30 (dd, *J* = 12.5, 2.7 Hz, 1H, C29), 4.27 (ddd, $^2J_{FH}$ = 50.9 Hz, *J* = 8.7, 7.8 Hz, 1H, C2), 4.079 (dt, *J* = 10.8, 10.2 Hz, 1H, C25), 4.078 (dd, *J* = 9.5, 3.4 Hz, 1H, C13), 4.01 (dd, *J* = 10.8, 2.2 Hz, 1H, C26), 3.95 (dd, *J* = 12.5, 6.1 Hz, 1H, C29), 3.94 (dd, *J* = 9.6, 8.6 Hz, 1H, C4), 3.86 (dt, *J* = 9.5, 6.8 Hz, 1H, C1'), 3.82 (d, *J* = 3.4 Hz, 1H, C14), 3.76 (s, 3H, Me), 3.74–3.63 (4H, overlapped, C3+2×C6+C16), 3.54 (dd, *J* = 9.5, 7.7 Hz, 1H, C12), 3.54–3.48 (3H, overlapped, C15+C16+C1'), 3.39 (ddd, *J* = 9.6, 4.9, 2.0 Hz, 1H, C5), 2.75 (brs, 1H, C14-OH), 2.52 (dd, *J* = 13.0, 4.6 Hz, 1H, C23), 2.09 (s, 3H, Ac), 2.04 (dd, *J* = 13.0, 12.2 Hz, 1H, C23), 2.01 (s, 3H, Ac), 1.98 (s, 3H, Ac), 1.88 (s, 3H, Ac), 1.87 (s, 3H, Ac), 1.65–1.59 (2H, overlapped, C2'), 1.38–1.21 (18H, overlapped, dodecyl), 0.88 (t, *J* = 7.0 Hz, 3H, C12'); ^{13}C NMR (151 MHz, $CDCl_3$) δ [171.0, 170.7, 170.4, 170.14, 170.06] (Ac), 168.5 (C21), [139.1, 138.8, 138.5, 138.4, 128.42, 128.35, 128.3, 128.2, 128.0, 127.70, 127.66, 127.63, 127.60, 127.5, 127.4] (Bn, one signal is missing, possibly due to overlapping), 102.5 (C11), 100.6 (d, $^2J_{FC}$ = 23.0 Hz, C1), 98.5 (C22), 92.7 (d, $^1J_{FC}$ = 186.8 Hz, C2), 81.6 (d, $^2J_{FC}$ = 17.4 Hz, C3), 78.6 (C12), 76.4 (C13), 76.2 (d, $^3J_{FC}$ = 8.1 Hz, C4), 75.3 (C5), 75.1 (O12-Bn), 74.6 (d, $^4J_{FC}$ = 1.4 Hz, O3-Bn), 73.5 (O16-Bn), 73.2 (O6-Bn), 72.9 (C26), 72.6 (C15), 70.1 (C1'), 69.2 (C24), 68.9 (C28), 68.8 (C16), 68.5 (C6), 68.1 (C14), 67.3 (C27), 62.4 (C29), 53.2 (Me), 49.4 (C25), 36.7 (C23), [32.0, 29.82, 29.79, 29.76, 29.73, 29.70, 29.54, 29.47, 26.0] (dodecyl), 23.3 (Ac), 22.8 (dodecyl), [21.3, 21.0, 20.8, 20.6] (Ac), 14.3 (C12'); ^{19}F NMR (564 MHz, $CDCl_3$) δ -195.9 (ddd, $^2J_{FH}$ = 50.9 Hz and $^3J_{FH}$ = 16.0, 2.9 Hz); MALDI-TOF MS {matrix = DHB (EtOAc)} found: 1368.63 calcd: 1368.63 for $C_{72}H_{96}FNNaO_{22}$ $[M+Na]^+$; IR: 3511, 3356, 3329, 3066, 3029, 3009, 2925, 2855, 1743, 1669, 1606, 1586, 1539, 1497, 1455, 1368, 1304, 1215, 1155, 1117, 1048, 1002, 948, 858, 824, 797, 751, 698, 667.

$^{13}C\{^{1}H_{sel}\}$ (δ ^{1}H = 3.76 ppm, Me) NMR (151 MHz, $CDCl_3$)

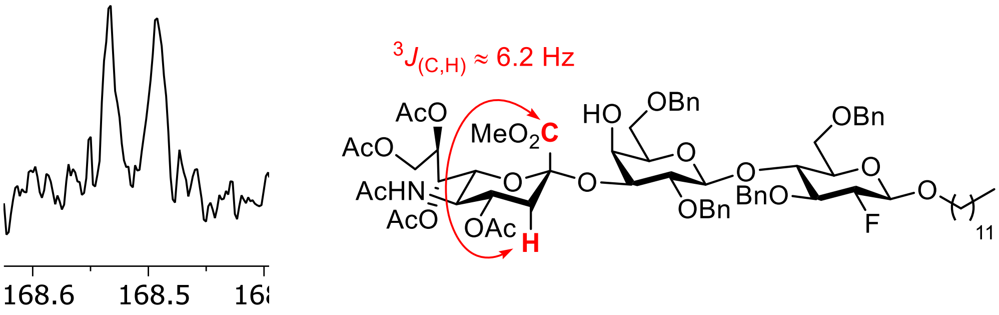

18: $[\alpha]_D^{27}$ -0.6 (c 1.00, $CHCl_3$); ^{1}H NMR (599 MHz, $CDCl_3$) δ 7.44–7.21 (20H, overlapped, Bn), 5.25 (dd, J = 4.3, 2.7 Hz, 1H, C27), 5.22 (ddd, J = 7.6, 4.3, 2.4 Hz, 1H, C28), 5.17 (ddd, J = 12.1, 10.3, 4.5 Hz, 1H, C24), 4.89 (d, J = 11.4 Hz, 1H, O3-Bn), 4.87 (d, J = 11.3 Hz, 1H, O12-Bn), 4.81 (d, J = 11.4 Hz, 1H, O3-Bn), 4.76 (dd, J = 12.4, 2.4 Hz, 1H, C29), 4.66 (d, J = 11.3 Hz, 1H, O12-Bn), 4.57–4.52 (1H, overlapped, NH), 4.57 (d, J = 7. Hz, 1H, C11), 4.55 (d, J = 12.2 Hz, 1H, O6-Bn), 4.52 (d, J = 12.2 Hz, 1H, O6-Bn), 4.47 (d, J = 12.1 Hz, 1H, O16-Bn), 4.42 (d, J = 12.1 Hz, 1H, O16-Bn), 4.41 (dd, J = 7.5 Hz, $^{3}J_{FH}$ = 3.2 Hz, 1H, C1), 4.33 (dd, J = 10.6, 2.7 Hz, 1H, C26), 4.29 (ddd, $^{2}J_{FH}$ = 50.8 Hz and J = 8.2, 7.5 Hz, 1H, C2), 4.09 (dt, J = 10.6, 10.4 Hz, 1H, C25), 4.02 (dd, J = 12.4, 7.6 Hz, 1H, C29), 4.01 (dd, J = 9.8, 8.9 Hz, 1H, C4), 3.90 (d, J = 3.1 Hz, 1H, C14), 3.86 (dt, J = 9.5, 6.8 Hz, 1H, C1’), 3.82–3.76 (3H, overlapped, 2×C6+C13), 3.73–3.63 (3H, overlapped, C3+C12+C16), 3.65 (s, 3H, Me), 3.58 (dd, J = 10.0, 5.1 Hz, 1H, C16), 3.50 (dt, J = 9.5, 6.8 Hz, 1H, C1’), 3.43 (m, 1H, C5), 3.40 (t, J = 5.2 Hz, 1H, C15), 2.53 (dd, J = 13.7, 4.5 Hz, 1H, C23), 2.12 (s, 3H, Ac), 2.09 (s, 3H, Ac), 2.00 (dd, J = 13.7, 12.1 Hz, 1H, C23), 1.984 (s, 3H, Ac), 1.983 (s, 3H, Ac), 1.73 (s, 3H, Ac), 1.66–1.59 (2H, overlapped, C2’), 1.39–1.21 (18H, overlapped, dodecyl), 0.88 (t, J = 7.0 Hz, 3H, C12’); ^{13}C NMR (151 MHz, $CDCl_3$) δ [170.8, 170.7, 170.4, 170.30, 170.28] (Ac), 167.9 (C21), [138.72, 138.71, 138.5, 138.1, 128.6, 128.5, 128.4, 128.3, 128.2, 128.14, 128.09, 127.8, 127.7, 127.6, 127.54, 127.53] (Bn), 102.4 (C11), 100.7 (d, $^{2}J_{FC}$ = 23.1 Hz, C1), 99.5 (C22), 92.7 (d, $^{1}J_{FC}$ = 186.7 Hz, C2), 81.1 (d, $^{2}J_{FC}$ = 17.7 Hz, C3), 78.5 (C12), 78.1 (C13), 75.9 (d, $^{3}J_{FC}$ = 8.1 Hz, C4), 75.4 (O12-Bn), 75.3 (C5), 74.4 (d, $^{4}J_{FC}$ = 1.7 Hz, O3-Bn), 73.7 (O16-Bn), 73.2 (O6-Bn), 72.7 (C15), 72.1 (C26), 71.6 (C28), 70.2 (C1’), 69.4 (C16), 68.8 (C24), 68.6 (C27), 68.4 (C6), 68.2 (C14), 62.8 (C29), 53.2 (Me), 48.8 (C25), 35.6 (C23), [32.1, 29.80, 29.77, 29.74, 29.71, 29.70, 29.54, 29.48, 26.0] (dodecyl), 23.3 (Ac), 22.8 (dodecyl), [21.3, 21.0, 20.9 (2C)] (Ac), 14.3 (C12’); ^{19}F NMR (564 MHz, $CDCl_3$) δ -195.6 (ddd, $^{2}J_{FH}$ = 50.8 Hz and $^{3}J_{FH}$ = 15.9, 3.2 Hz); MALDI-TOF MS {matrix = DHB (EtOAc)} found: 1368.65 calcd: 1368.63 for $C_{72}H_{96}FNNaO_{22}$ $[M+Na]^+$; IR: 3361, 3065, 3031, 2925, 2855, 1743, 1688, 1662, 1542, 1497, 1455, 1368, 1313, 1224, 1161, 1124, 1040, 1029, 987, 943, 912, 874, 831, 790, 749, 698, 666.

$^{13}C\{^{1}H_{sel}\}$ (δ ^{1}H = 3.65 ppm, Me) NMR (151 MHz, $CDCl_3$)

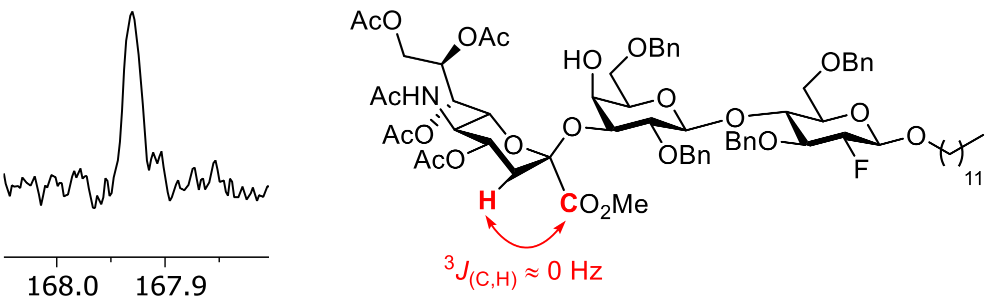

Compounds **19** (β-anomer) and **20** (α-anomer).

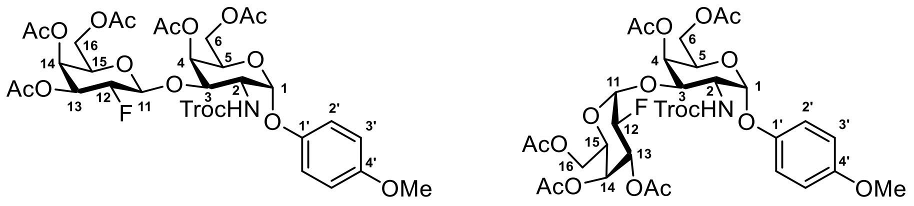

To a mixture of **5**[5] (142 mg, 0.259 mmol, 1.0 eq.) and **4**[6] (323 mg, 0.541 mmol, 2.1 eq.) in dry MeCN (2.5 mL) was added a solution of TMSOTf (0.10 M in dry MeCN, 0.52 mL, 0.052 mmol, 0.2 eq.) at -40 °C. After stirring for 1 h at the same temperature, the reaction was quenched by adding NEt_3 (0.15 mL). The mixture was concentrated *in vacuo*. Chromatographic purification (SiO_2, cHex:EtOAc = 9:1 to 75:25) followed by chromatographic purification (SiO_2, CH_2Cl_2:EtOAc = 95:5) afforded crude material. A flask, thoroughly purged with argon, was charged with $Pd(OH)_2$ (20 wt% on activated carbon, 28.7 mg), to which was added a solution of this crude material in THF/MeOH (1/1, 3.2 mL). The atmosphere was changed from argon to H_2 (1 atm), and the mixture was stirred for 15 h at room temperature. After changing the atmosphere from H_2 to argon, the mixture was filtered through a Celite® pad, washed with MeOH and was concentrated *in vacuo*. To a mixture of this crude material in Ac_2O (1.5 mL) and pyridine (1.5 mL) was added DMAP (3.9 mg, 0.032 mmol, 16 mol%) at room temperature. After stirring for 6 h, the reaction was quenched by adding saturated aqueous $NaHCO_3$ at 0 °C. The mixture was extracted with CH_2Cl_2 (×3). The combined organic layers were washed with 1 M aqueous HCl (×3) and saturated aqueous $NaHCO_3$, dried (Na_2SO_4), and concentrated *in vacuo*. Chromatographic purification (SiO_2, cHex:EtOAc = 6:4) followed by chromatographic purification (SiO_2, CH_2Cl_2:EtOAc = 9:1 to 8:2) afforded **19** (107 mg, 0.128 mmol, 49 %) as white amorphous solid and **20** (8.8 mg, 0.011 mmol, 4 %) as white amorphous solid.

19: $[\alpha]_D^{27}$ +111.9 (c 1.00, $CHCl_3$); The solution of **19** in $CDCl_3$ showed a mixture of compounds (85:15 based on ^{1}H NMR) tentatively assigned as rotamers. ^{1}H NMR (599 MHz, $CDCl_3$, The discernible signals for the minor rotamer are marked with an asterisk) δ 7.01 (m, 2H, C2'), 6.83 (m, 2H, C3'), 5.52 (d, 1H, *J* = 2.9 Hz, C4), 5.51 (d, *J* = 3.8 Hz, 1H, C1), 5.38 (m, 1H, C14), 5.33 (d, *J* = 9.8 Hz, 1H, NH), 5.07 (ddd, $^3J_{FH}$ = 13.3 Hz and *J* = 9.9, 3.6 Hz, 1H, C13), 4.85* (d, *J* = 11.8 Hz, 1H, Troc), 4.76 (d, *J* = 12.0 Hz, 1H, Troc), 4.71 (dd, *J* = 7.6 Hz and $^3J_{FH}$ = 4.1 Hz, 1H, C11), 4.68 (d, *J* = 12.0 Hz, 1H, Troc), 4.59* (d, *J* = 11.8 Hz, 1H, Troc), 4.491 (ddd, *J* = 10.4, 9.8, 3.8 Hz, 1H, C2), 4.485 (ddd, $^2J_{FH}$ = 51.5 Hz, *J* = 9.9, 7.6 Hz, 1H, C12), 4.29 (dd, *J* = 8.1, 4.4 Hz, 1H, C5), 4.21 (dd, *J* = 11.7, 4.4 Hz, 1H, C6), 4.144 (dd, *J* = 11.3, 6.8 Hz, 1H, C16), 4.136 (dd, *J* = 10.4, 2.9 Hz, 1H, C3), 4.05 (dd, *J* = 11.3, 7.0 Hz, 1H, C16), 3.96 (dd, *J* = 11.7, 8.1 Hz, 1H, C6), 3.93 (td, *J* = 6.9, 1.3 Hz, 1H, C15), 3.77 (s, 3H, OMe), 2.16 (s, 3H, Ac), 2.13 (s, 3H, Ac), 2.05 (s, 3H, Ac), 2.04 (s, 3H, Ac), 1.94 (s, 3H, Ac); ^{13}C NMR (151 MHz, $CDCl_3$) δ [170.6, 170.5, 170.2, 170.1, 170.0] (Ac), 155.8 (C4'), 154.5 (Troc), 150.1 (1'), 118.5 (2'), 114.9 (3'), 101.6 (d, $^2J_{FC}$ = 22.9 Hz, C11), 97.8 (C1), 95.4 (Troc), 88.1 (d, $^1J_{FC}$ = 187.1 Hz, C12), 76.4 (C3), 74.9 (Troc), 70.98 (C15), 70.95 (d, $^2J_{FC}$ = 18.8 Hz, C13), 68.9 (C4), 68.4 (C5), 67.5 (d, $^3J_{FC}$ = 8.2 Hz, C14), 62.8 (C6), 61.0 (C16), 55.8 (OMe), 51.0 (C2), [20.9, 20.80, 20.78, 20.71, 20.66] (Ac); ^{19}F NMR (564 MHz, $CDCl_3$) δ -206.6 (dddd, $^2J_{FH}$ = 51.5 Hz and $^3J_{FH}$ = 13.3, 4.1 Hz and $^4J_{FH}$ = 3.7 Hz); ESI-MS found: 856.1162 calcd: 856.1160 for $C_{32}H_{39}Cl_3FNNaO_{17}$ $[M+Na]^+$; IR: 3339, 2958, 2841, 1740, 1530, 1506, 1442, 1368, 1210, 1179, 1035, 950, 914, 896, 866, 820, 791, 767, 732, 718, 657.

20: $[\alpha]_D^{27}$ +146.5 (c 1.00, $CHCl_3$); The solution of **20** in $CDCl_3$ showed a mixture of compounds (82:18 based on ^{19}F NMR) tentatively assigned as rotamers. ^{1}H NMR (599 MHz, $CDCl_3$, The discernible signals for the minor rotamer are marked with an asterisk) δ 7.07* (m, 2H, C2'), 7.03 (m, 2H, C2'), 6.84 (m, 2H, C3'), 5.65 (d, *J* = 10.1 Hz, 1H, NH), 5.59 (brd, *J* = 3.4 Hz, 1H, C4), 5.48 (d, *J* = 3.6 Hz, 1H, C1), 5.43 (dd, *J* = 3.4 Hz and $^4J_{FH}$ = 3.1 Hz, 1H, C14), 5.33 (d, *J* = 4.0 Hz, 1H, C11), 5.25* (m, 1H, C13), 5.19

(ddd, $^3J_{FH}$ = 11.0 Hz and J = 10.2, 3.4 Hz, 1H, C13), 4.95 (d, J = 12.1 Hz, 1H, Troc), 4.86* (d, J = 11.8 Hz, 1H, Troc), 4.83* (d, J = 11.8 Hz, 1H, Troc), 4.73 (ddd, $^2J_{FH}$ = 49.2 Hz and J = 10.2, 4.0 Hz, 1H, C12), 4.72 (d, J = 12.1 Hz, 1H, Troc), 4.51–4.43 (2H, overlapped, C2+C15), 4.34 (m, 1H, C5), 4.25 (dd, J = 11.0, 3.4 Hz, 1H, C3), 4.19 (dd, J = 11.4, 7.4 Hz, 1H, C16), 4.18 (dd, J = 11.3, 5.6 Hz, 1H, C6), 4.07 (dd, J = 11.3, 7.3 Hz, 1H, C6), 4.01 (dd, J = 11.4, 5.5 Hz, 1H, C16), 3.78 (s, 3H, OMe), 2.15 (s, 6H, 2×Ac), 2.12 (s, 3H, Ac), 2.01 (s, 3H, Ac), 1.98 (s, 3H, Ac); ^{13}C NMR (151 MHz, $CDCl_3$) δ [171.2, 170.64, 170.57, 170.1, 169.8] (Ac), 155.9 (C4'), 154.4 (Troc), 150.3 (C1'), 118.5 (C2'), 114.9 (C3'), 98.2 (C1), 95.5 (Troc), 92.6 (d, $^2J_{FC}$ = 20.3 Hz, C11), 84.4 (d, $^1J_{FC}$ = 193.5 Hz, C12), 75.0 (Troc), 70.5 (C3), 68.5 (d, $^3J_{FC}$ = 7.6 Hz, C14), 68.4 (d, $^2J_{FC}$ = 19.2 Hz, C13), 67.6 (C5), 66.5 (C15), 64.7 (C4), 62.8 (C16), 62.3 (C6), 55.8 (OMe), 50.7 (C2), [21.1, 20.8, 20.73, 20.71 (2C)] (Ac); ^{19}F NMR (564 MHz, $CDCl_3$, The signals for the minor rotamer are marked with an asterisk) δ -209.5 (ddd, $^2J_{FH}$ = 49.2 Hz and $^3J_{FH}$ = 11.0 Hz and $^4J_{FH}$ = 3.1 Hz), -209.8* (dd, $^2J_{FH}$ = 49.5 Hz and $^3J_{FH}$ = 10.8 Hz); ESI-MS found: 856.1159 calcd: 856.1160 for $C_{32}H_{39}Cl_3FNNaO_{17}$ [M+Na]$^+$; IR: 3361, 3016, 2996, 2957, 2839, 1740, 1506, 1464, 1455, 1442, 1371, 1300, 1209, 1150, 1135, 1074, 1035, 977, 957, 944, 910, 871, 819, 799, 752, 719, 667.

Compound **S3**

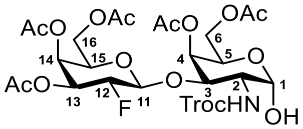

To a solution of **19** (100 mg, 0.120 mmol, 1.0 eq.) in MeCN/water (3/1, 4 mL) was added cerium ammonium nitrate (99.2 mg, 0.181 mmol, 1.5 eq.) at 0 °C. After stirring for 1. h, additional cerium ammonium nitrate (120 mg, 0.219 mmol, 1.8 eq.) was added to the mixture at 0 °C. After further stirring for 5 h, the reaction was quenched by adding saturated aqueous $NaHCO_3$ and saturated aqueous $Na_2S_2O_3$ at 0 °C. The mixture was extracted with CH_2Cl_2 (×3). The combined organic layers were dried (Na_2SO_4), and concentrated *in vacuo*. Chromatographic purification (SiO_2, cHex:EtOAc = 6:4 to 45:55) afforded **S3** (69.8 mg, 0.0958 mmol, 80 %) as a pale yellow oil.

S3: $[\alpha]_D^{27}$ +58.3 (c 1.00, $CHCl_3$); The solution of **S3** in $CDCl_3$ showed a mixture of compounds (78:15:7 based on ^{1}H NMR) tentatively assigned as isomers. The signals for the minor isomers are omitted. ^{1}H NMR (500 MHz, $CDCl_3$) δ 5.48 (brd, J = 3.3 Hz, 1H, C4), 5.38 (br, 1H, C1), 5.36 (m, 1H, C14), 5.31 (d, J = 9.8 Hz, 1H, NH), 5.04 (ddd, $^3J_{FH}$ = 13.3 Hz and J = 9.8, 3.5 Hz, 1H, C13), 4.76 (d, J = 12.0 Hz, 1H, Troc), 4.66 (dd, J = 7.5 Hz and $^3J_{FH}$ = 4.0 Hz, 1H, C11), 4.65 (d, J = 12.0 Hz, 1H, Troc), 4.45 (ddd, $^2J_{FH}$ = 51.4 Hz and J = 9.8, 7.5 Hz, 1H, C12), 4.35 (m, 1H, C5), 4.33 (ddd, J = 10.3, 9.8, 3.4 Hz, 1H, C2), 4.20 (dd, J = 11.6, 4.9 Hz, 1H, C6), 4.14 (dd, J = 11.3, 6.2 Hz, 1H, C16), 4.05 (dd, J = 11.3, 6.9 Hz, 1H, C16), 4.03 (dd, J = 10.3, 3.3 Hz, 1H, C3), 3.97 (dd, J = 11.6, 7.2 Hz, 1H, C6), 3.91 (m, 1H, C15), 3.46 (brs, 1H, OH), 2.14 (s, 3H, Ac), 2.12 (s, 3H, Ac), 2.07 (s, 3H, Ac), 2.05 (s, 3H, Ac), 2.03 (s, 3H, Ac); ^{13}C NMR (126 MHz, $CDCl_3$) δ [170.8, 170.6, 170.24, 170.16, 170.1] (Ac), 154.6 (Troc), 101.6 (d, $^2J_{FC}$ = 22.5 Hz, C11), 95.5 (Troc), 92.5 (C1), 88.0 (d, $^1J_{FC}$ = 186.4 Hz, C12), 75.9 (C3), 74.9 (Troc), 71.0 (d, $^2J_{FC}$ = 19.2 Hz, C13), 70.9 (C15), 69.3 (C4), 67.7 (C5), 67.5 (d, $^3J_{FC}$ = 8.2 Hz, C14), 63.0 (C6), 61.0 (C16), 51.1 (C2), [20.96, 20.95, 20.8, 20.71, 20.67] (Ac); ^{19}F NMR (470 MHz, $CDCl_3$) δ -206.7 (dddd, $^2J_{FH}$ = 51.4 Hz and $^3J_{FH}$ = 13.3, 4.0 Hz and $^4J_{FH}$ = 3.2 Hz); ESI-MS found: 750.0735 calcd: 750.0741 for $C_{25}H_{33}Cl_3FNNaO_{16}$ [M+Na]$^+$; IR: 3354, 3023, 2960, 1738, 1528, 1432, 1369, 1218, 1176, 1040, 951, 914, 852, 818, 751, 667.

Compound **2**

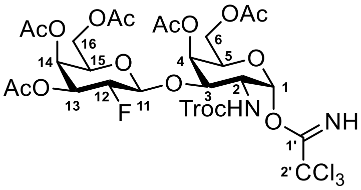

To a solution of **S3** (106 mg, 0.145 mg, 1.0 eq.) in dry CH_2Cl_2 (1.5 mL) was added successively Cl_3CCN (0.15 mL, 1.48 mmol, 10 eq.) and a solution of DBU (0.10 M in CH_2Cl_2, 0.29 mL, 0.029 mmol, 0.2 eq.) at room temperature. After stirring for 2.5 h, the mixture was concentrated *in vacuo*. Chromatographic purification (SiO_2, cHex:EtOAc = 7:3 to 65:35) afforded **2** (96.3 mg, 0.110 mmol, 76 %) as white solid.

2: $[\alpha]_D^{28}$ +80.7 (c 1.00, MeCN); The solution of **2** in CD_2Cl_2 showed a mixture of compounds (79:14:7 based on ^{19}F NMR) tentatively assigned as isomers. The signals for the minor isomers are omitted. ^{1}H NMR (500 MHz, CD_2Cl_2) δ 8.85 (s, 1H, C=NH), 6.45 (d, J = 3.7 Hz, 1H, C1), 5.56 (d, J = 3.4 Hz, 1H, C4), 5.38 (m, 1H, C14), 5.17 (d, J = 9.1 Hz, 1H, C2-NH), 5.09 (ddd, $^3J_{FH}$ = 13.3 Hz and J = 9.8, 3.6 Hz, 1H, C13), 4.78 (d, J = 12.1 Hz, 1H, Troc), 4.77 (dd, J = 7.6 Hz and $^3J_{FH}$ = 3.9 Hz, 1H, C11), 4.67 (d, J = 12.1 Hz, 1H, Troc), 4.51 (ddd, J = 11.0, 9.1, 3.7 Hz, 1H, C2), 4.50 (ddd, $^2J_{FH}$ = 51.0 Hz and J = 9.8, 7.6 Hz, 1H, C12), 4.31 (dd, J = 7.5, 4.4 Hz, 1H, C5), 4.24 (dd, J = 11.8, 4.4 Hz, 1H, C6), 4.14 (dd, J = 11.0, 6.1 Hz, 1H, C16), 4.10 (dd, J = 11.0, 3.4 Hz, 1H, C3), 4.02 (dd, J = 11.0, 6.8 Hz, 1H, C16), 3.98 (m, 1H, C15), 3.92 (dd, J = 11.8, 7.5 Hz, 1H, C6), 2.16 (s, 3H, Ac), 2.11 (s, 3H, Ac), 2.03 (s, 3H, Ac), 2.01 (s, 3H, Ac), 1.99 (s, 3H, Ac); ^{13}C NMR (126 MHz, CD_2Cl_2) δ [170.9, 170.8, 170.5, 170.4, 170.3] (Ac), 160.9 (C1'), 154.8 (Troc), 101.6 (d, $^2J_{FC}$ = 23.1 Hz, C11), 95.9 (2C, C1+Troc), 91.4 (C2'), 88.6 (d, $^1J_{FC}$ = 186.8 Hz, C12), 76.1 (C3), 75.2 (Troc), 71.6 (C15), 71.3 (d, $^2J_{FC}$ = 18.7 Hz, C13), 70.7 (C5), 69.1 (C4), 67.9 (d, $^3J_{FC}$ = 8.3 Hz, C14), 63.1 (C6), 61.4 (C16), 51.0 (C2), [21.1, 21.0 (2C), 20.93, 20.88] (Ac); ^{19}F NMR (470 MHz, CD_2Cl_2) δ -206.9 (ddd, $^2J_{FH}$ = 51.0 Hz, $^3J_{FH}$ = 13.3, 3.9 Hz); ESI-MS found: 892.9833 calcd: 892.9837 for $C_{27}H_{33}Cl_6FN_2NaO_{16}$ $[M+Na]^+$; IR: 3338, 3320, 2966, 1741, 1677, 1521, 1435, 1369, 1219, 1174, 1152, 1045, 1017, 964, 950, 880, 856, 837, 817, 796, 772, 731, 720, 662.

Compound **21**

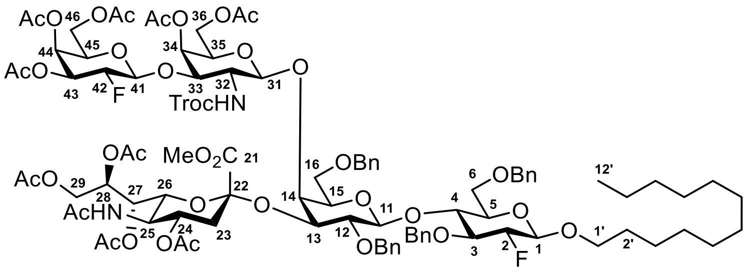

To a mixture of acceptor **3** (139 mg, 0.103 mmol, 1.0 eq.), donor **2** (135 mg, 0.155 mmol, 1.5 eq.) and Drierite™ (57 mg) in dry CH_2Cl_2 (3 mL) was added TMSOTf (5.6 µL, 0.031 µmol, 0.30 eq.) at 0 °C. After stirring for 1 h, the reaction was quenched by adding NEt_3 (0.1 mL). The mixture was filtered through cotton wool, washed with EtOAc and concentrated *in vacuo*. Chromatographic purification (SiO_2, cHex:EtOAc = 1:4) afforded **21** (136 mg, 66.3 µmol, 64%, β-anomer only) as a white solid.

21: $[\alpha]_D^{24}$ = +7.1 (c 0.25, $CHCl_3$); The solution of **21** in $CDCl_3$ showed a mixture of conformers (7.2:2.6:0.2 based on ^{19}F NMR). ^{1}H NMR (599 MHz, $CDCl_3$, the signals for the minor rotamers are

omitted) δ 7.52 (d, J = 7.7 Hz, 3H, Bn), 7.38 (t, J = 7.3 Hz, 2H, Bn), 7.32 – 7.16 (m, 14H, Bn), 6.00 (d, J = 8.9 Hz, 1H, NHTroc), 5.48 (br s, 1H, C34), 5.35 (br s, 1H, C44), 5.24 (dd, J = 9.8, 1.6 Hz, 1H, C27), 5.23 (d, J = 9.8 Hz, 1H, NHAc), 5.19 (dd, J = 9.4, 3.8 Hz, 1H, C28), 5.14 (d, J = 6.4 Hz, 1H, C24), 5.05 (t, $^3J_{FH}$ = 13.5 Hz and J =10.0 Hz, 1H, C43), 4.92 (d, J = 8.6 Hz, 1H, C31), 4.89 (d, J = 10.1 Hz, 1H, O3-Bn), 4.87 (d, J = 11.9 Hz, 1H, Troc), 4.76 – 4.71 (m, 2H, overlapped, O3-Bn+O12-Bn), 4.66 – 4.62 (m, 1H, C41), 4.58 (d, J = 11.6 Hz, 2H, overlapped, O6-Bn+O12-Bn), 4.55 (d, J = 12.0 Hz, 1H, Troc), 4.44 (d, J = 7.9 Hz, 1H, C1), 4.45 (dt, $^2J_{FH}$ = 49.8 Hz and J = 8.3 Hz, 1H, H42), 4.43 (d, J = 11.2 Hz, 1H, O6-Bn), 4.32 (d, J = 12.7 Hz, 1H, O16-Bn), 4.25 (dt, $^2J_{FH}$ = 51.0 Hz and J = 8.2 Hz, 1H, C2), 4.28 – 4.23 (m, 2H, overlapped, C36+O16-Bn), 4.23 – 4.15 (m, 3H, overlapped, C32+C35+C46), 4.09 – 4.01 (m, 3H, overlapped, C29+C46), 4.01 – 3.92 (m, 4H, overlapped, C14+C25+C33+C36), 3.93 (t, J = 9.3 Hz, 1H, C4), 3.92 – 3.83 (m, 4H, overlapped, C1'+C13+C26+C45), 3.88 (s, 3H, Me), 3.78 – 3.69 (m, 4H, C3+C6+C16), 3.55 – 3.48 (m, 2H, overlapped, C12+C1'), 3.45 – 3.42 (m, 1H, C16), 3.405 (dd, J = 9.7, 3.3 Hz, 1H, C5), 3.400 (dd, J = 10.0, 3.1 Hz, 1H, C15), 2.18 (s, 3H, Ac), 2.22 – 2.16 (m, 2H, overlapped, C23), 2.12 (s, 6H, 2Ac), 2.04 (s, 3H, Ac), 2.03 (s, 3H, Ac), 2.00 (s, 3H, Ac), 1.97 (s, 3H, Ac), 1.97 (s, 3H, Ac), 1.91 (s, 3H, Ac), 1.89 (s, 3H, Ac), 1.65 – 1.60 (m, 2H, C2'), 1.30 – 1.23 (m, 18H, dodecyl), 0.87 (t, J = 7.0 Hz, 3H, C12'); ^{13}C NMR (151 MHz, $CDCl_3$, the signals for the minor rotamers are omitted) δ [170.8, 170.53 (2C), 170.47, 170.3, 170.2, 170.1, 169.8, 169.7] (Ac), 168.8 (C21), 154.6 (Troc), [138.6 (2C), 138.5, 138.4, 129.0, 128.4, 128.3, 128.2, 127.8, 127.7, 127.62, 127.56, 127.2] (Bn, 3C missing due to overlapping), 102.3 (C11), 102.0 (C31), 101.6 (d, $^2J_{FC}$ = 23.3 Hz, C41), 100.7 (d, $^2J_{FC}$ = 23.0 Hz, C1), 99.6 (C22), 96.0 (Troc), 92.5 (d, $^1J_{FC}$ = 186.7 Hz, C2), 88.3 (d, $^1J_{FC}$ = 186.7 Hz, C42), 81.3 (d, $^2J_{FC}$ = 17.4 Hz, C3), 80.2 (C33), 78.9 (C12), 78.2 (C14), 76.3 (C13), 76.1 (d, $^3J_{FC}$ = 7.3 Hz, C4), 75.35 (C5), 75.35 (O12-Bn), 75.26 (O3-Bn), 74.7 (Troc), 73.5 (O16-Bn), 73.42 (C15), 73.42 (O6-Bn), 72.2 (C26), 71.1 (d, $^2J_{FC}$ = 18.8 Hz, C43), 70.8 (C35), 70.7 (C45), 70.1 (C1'), 69.2 (C24), 69.1 (C16), 68.4 (C34), 68.3 (C6), 68.1 (C28), 67.3 (C44), 66.5 (C27), 62.5 (C36), 61.9 (C29), 60.7 (C46), 53.9 (C32), 53.5 (Me), 49.5 (C25), 35.1(23), [32.0, 29.79, 29.76, 29.74, 29.71, 29.69, 29.54, 29.47, 26.0] (dodecyl), 23.3 (Ac), 22.8 (dodecyl), [21.4, 20.9, 20.8, 20.8, 20.7, 20.7, 20.7, 20.6] (Ac, 1 C missing due to overlapping), 14.2 (C12'); ^{19}F NMR (564 MHz, $CDCl_3$, the signals for the minor rotamers are marked with an asterisk) δ -194.63** (dd, $^2J_{FH}$ = 50.9 Hz and $^3J_{FH}$ = 16.0 Hz, C2), -196.38 (dd, $^2J_{FH}$ = 50.9 Hz and $^3J_{FH}$ = 16.0 Hz, C2), -196.70* (dd, $^2J_{FH}$ = 50.8 Hz and $^3J_{FH}$ = 16.0 Hz, C2), -206.18 (dd, $^2J_{FH}$ = 51.4 Hz and $^3J_{FH}$ = 13.5 Hz, C42), -206.67* (dd, $^2J_{FH}$ = 51.3 Hz and $^3J_{FH}$ = 10.7 Hz, C42), -206.74 – -206.86** (m, C42); MALDI-TOF MS {matrix = DHB (EtOAc)} found: 2077.83 calcd: 2077.70 for $C_{97}H_{127}Cl_3F_2N_2NaO_{37}$ $[M+Na]^+$; IR (neat) 2925, 2853, 1742, 1691, 1531, 1453, 1367, 1301, 1216, 1166, 1044, 946, 912, 820, 735, 698.

Compound **22**

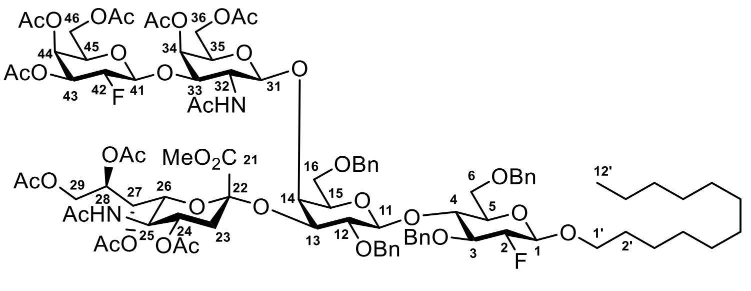

To a mixture of pentasaccharide **21** (131 mg, 64.0 µmol, 1.00 eq.) and freshly activated Zn powder[7] (1.50 g) in CH_2Cl_2 (5 mL) was added AcOH (5 mL) at room temperature. After stirring for 3 h, the mixture was filtered through a glass filter and the filtrate was concentrated *in vacuo*. The crude material was redissolved in Ac_2O/pyridine (2 mL/4 mL), followed by addition of DMAP (2.00 mg, 16.4 µmol, 0.25 eq).

After stirring at room temperature for 2 h, the reaction was quenched by adding saturated aqueous $NaHCO_3$ at 0 °C. The mixture was extracted with CH_2Cl_2 (×3) and the combined organic layers were washed with 1 M aqueous HCl (×3) and saturated aqueous $NaHCO_3$, dried (Na_2SO_4), and concentrated *in vacuo*. Chromatographic purification (SiO_2, 100% EtOAc) afforded **22** (78 mg, 40 µmol, 62 %) as a colorless oil.

22: The solution of **22** in $CDCl_3$ showed a mixture of conformers (8:2 based on ^{19}F NMR) as proven by the dynamic nature of the species in variable temperature NMR experiments (*vide infra*). ^{1}H NMR (599 MHz, $CDCl_3$, the signals of the minor conformer are omitted) δ 7.53 (d, J = 7.6 Hz, 2H, Bn), 7.37 (t, J = 7.6 Hz, 2H, Bn), 7.31 – 7.15 (m, 16H, overlapped, Bn), 6.24 (d, J = 8.1 Hz, 1H, C32-NHAc), 5.46 (d, J = 2.6 Hz, 1H, C34), 5.35 (t, J = 3.1 Hz, 1H, C44), 5.28 (dd, J = 5.0, 2.6 Hz, 1H, C28), 5.25 (dd, J = 9.7, 1.7 Hz, 1H, C27), 5.20 (d, J = 9.6 Hz, 1H, C25-NHAc), 5.13 – 5.04 (m, 2H, overlapped, C24+C43), 4.95 (d, J = 8.1 Hz, 1H, C31), 4.92 (d, J = 10.4 Hz, 1H, O3-Bn), 4.76 (d, J = 10.2 Hz, 1H, O3-Bn), 4.72 (d, J = 11.5 Hz, 1H, O12-Bn), 4.65 – 4.61 (m, 2H, overlapped, O12-Bn+C41), 4.53 (d, J = 7.8 Hz, 1H, C11), 4.51 – 4.40 (m, 3H, overlapped, C1+O6-Bn), 4.48 (dt, $^{2}J_{FH}$ = 51.9 Hz and J = 8.7 Hz, 1H, C42), 4.28 (d, J = 12.1 Hz, 1H, O16-Bn), 4.24 (dt, $^{2}J_{FH}$ = 51.6 Hz and J = 8.1 Hz, 1H, C2), 4.24 – 4.14 (m, 5H, overlapped, O16-Bn+C32+C33+C36+C46), 4.12 – 4.09 (m, 1H, C29), 4.08 – 4.00 (m, 3H, overlapped, C29+C35+C46), 4.00 – 3.88 (m, 6H, overlapped, C4+C13+C25+C26+C36+C45), 3.88 – 3.83 (m, 2H, overlapped, C14+C1'), 3.85 (s, 3H, Me), 3.73 – 3.66 (m, 3H, overlapped, C3+C6+C16), 3.62 (dd, J = 11.1, 5.4 Hz, 1H, C6), 3.55 – 3.51 (m, 1H, C1'), 3.49 (t, J = 9.6 Hz, 1H, C12), 3.45 – 3.40 (m, 2H, overlapped, C5+C15), 3.39 – 3.36 (m, 1H, C16), 2.39 (dd, J = 13.9, 4.9 Hz, 1H, C23), 2.17 (s, 3H, Ac), 2.15 – 2.10 (m, 1H, C23), 2.12 (s, 3H, Ac), 2.12 (s, 3H, Ac), 2.04 (s, 3H, Ac), 2.01 (s, 3H, Ac), 1.99 (s, 3H, Ac), 1.95 (s, 3H, Ac), 1.91 (s, 6H, 2xAc), 1.90 (s, 6H, 2xAc), 1.82 (s, 3H, Ac), 1.28 – 1.24 (m, 18H, dodecyl), 0.88 (t, J = 7.1 Hz, 3H, C12'); ^{13}C NMR (151 MHz, $CDCl_3$, the signals of the minor conformer are omitted) δ [171.3, 170.9, 170.57, 170.56, 170.5, 170.30, 170.28, 170.1, 169.9, 169.8] (Ac), 168.8 (C21), [138.8, 138.7, 138.63, 138.57, 129.0, 128.37, 128.35, 128.3, 128.2, 128.1, 127.8, 127.64, 127.60, 127.56, 127.5, 127.1] (Bn), 102.5 (C11), 101.9 (d, $^{2}J_{FC}$ = 23.1 Hz, C41), 101.7 (C31), 100.7 (d, $^{2}J_{FC}$ = 23.1 Hz, C1), 99.3 (C22), 92.6 (d, $^{1}J_{FC}$ = 186.6 Hz, C2), 88.2 (d, $^{1}J_{FC}$ = 186.0 Hz, C42), 81.6 (d, $^{2}J_{FC}$ = 17.6 Hz, C3), 79.3 (C33), 79.1 (C12), 77.3 (C14), 76.34 (d, $^{3}J_{FC}$ = 8.4 Hz, C4), 76.29 (C13), 75.4 (C5), 75.3 (O3-Bn), 75.2 (O12-Bn), 73.4 (O16-Bn), 73.3 (O6-Bn), 72.2 (C26), 71.2 (d, $^{2}J_{FC}$ = 18.7 Hz, C43), 70.8 (C35), 70.6 (C45), 70.2 (C1'), 69.14 (C24), 69.11 (C16), 68.7 (C34), 68.6 (C6), 68.1 (C28), 67.4 (d, $^{3}J_{FC}$ = 8.5 Hz, C44), 66.7 (C27), 62.6 (C36), 62.0 (C29), 60.8 (C46), 53.2 (Me), 52.6 (C32), 49.6 (C25), 35.7 (C23), [32.1, 29.84, 29.81, 29.78, 29.76, 29.7, 29.6, 29.5, 26.1, 22.8] (dodecyl), [23.4, 21.4, 21.2, 21.1, 21.0, 20.81, 20.79, 20.76, 20.74, 20.70, 20.6] (Ac), 14.3 (C12'); ^{19}F NMR (564 MHz, $CDCl_3$, the signals of the minor conformer are marked with an asterisk) δ -196.36 (dd, $^{2}J_{FH}$ = 51.0 Hz and $^{3}J_{FH}$ = 16.0 Hz, C2), -196.91* (dd, $^{2}J_{FH}$ = 50.4 Hz and $^{3}J_{FH}$ = 16.2 Hz, C2), -206.60 – -206.74* (m, C42), -206.72 (ddt, $^{2}J_{FH}$ = 51.5 Hz and $^{3}J_{FH}$ = 13.3, 3.5 Hz and $^{4}J_{FH}$ = 3.5 Hz, C42); MALDI-TOF MS {matrix = DHB (EtOAc)} found: 1945.76 calcd: 1945.81 for $C_{96}H_{128}F_2N_2NaO_{36}$ $[M+Na]^+$.

Compound **23**

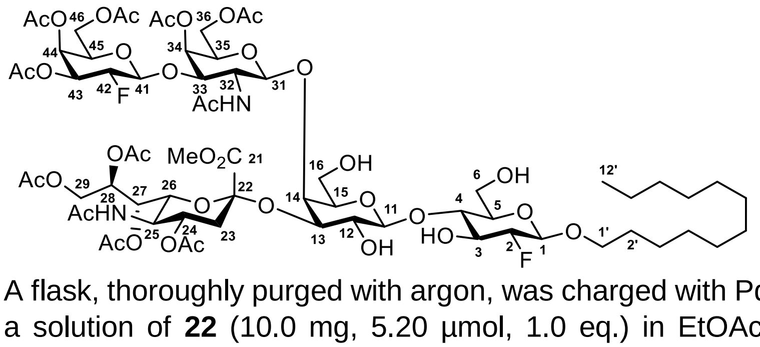

A flask, thoroughly purged with argon, was charged with Pd/C (10.0 mg, 100 wt%), to which was added a solution of **22** (10.0 mg, 5.20 µmol, 1.0 eq.) in EtOAc/H_2O/t-BuOH (2/1/1). The atmosphere was changed from argon to H_2 (1 atm), and the mixture was stirred at room temperature for 24 h. The reaction

mixture was filtered over Celite® and thoroughly washed with EtOAc and H_2O. The product was purified by column chromatography (SiO_2, cHex:EtOAc:MeOH = 3:3:0.5) and obtained as a colorless foam (7.00 mg, 4.50 µmol, 86 %).

23: $[\alpha]_D^{24}$ = +16.3 (c 0.53, MeCN); The solution of **23** in $CDCl_3$ showed a mixture of conformers (95:5 based on ^{19}F NMR). The signals of the minor conformer are omitted. ^1H NMR (599 MHz, $CDCl_3$) δ 5.88 (d, *J* = 7.3 Hz, 1H, C32-NHAc), 5.43 – 5.39 (m, 1H, C28), 5.39 (d, *J* = 3.6 Hz, 1H, C34), 5.35 (mdd, *J* = 3.6, 1.2 Hz, 1H, C44), 5.26 (dd, *J* = 9.2, 1.8 Hz, 1H, C27), 5.24 (d, *J* = 9.7 Hz, 1H, C25-NHAc), 5.21 (d, *J* = 8.4 Hz, 1H, C31), 5.04 (ddd, $^3J_{FH}$ = 13.5 Hz and *J* = 9.9, 3.6 Hz, 1H, C43), 5.00 (td, *J* = 10.5, 4.9 Hz, 1H, C24), 4.84 (dd, *J* = 10.6, 3.6 Hz, 1H, H33), 4.60 (dd, *J* = 7.9 Hz and $^3J_{FH}$ = 3.9 Hz, 1H, C41), 4.59 (d, *J* = 8.5 Hz, 1H, C11), 4.49 (dd, *J* = 7.9 Hz and $^3J_{FH}$ = 2.7 Hz, 1H, C1), 4.43 (ddd, $^2J_{FH}$ = 51.5 Hz and *J* = 9.9, 7.6 Hz, 1H, C42), 4.27 (dd, *J* = 12.4, 2.9 Hz, 1H, C29), 4.25 (dd, *J* = 11.5, 3.3 Hz, 1H, C36), 4.20 (dd, $^2J_{FH}$ = 51.2 Hz and *J* = 8.9, 7.7 Hz, 1H, C2), 4.14 (dd, *J* = 10.7, 1.8 Hz, 1H, C26), 4.12 (dd, *J* = 11.3, 6.2 Hz, 1H, C46), 4.09 – 4.00 (m, 3H, overlapped, C5+C25+C46), 3.98 (dd, *J* = 12.4, 7.0 Hz, 1H, C29), 3.97 – 3.85 (m, 7H, overlapped, C1'+2xC6+C14+C35+C36+C45), 3.84 (s, 3H, Me), 3.83 – 3.75 (m, 2H, overlapped, C3+C16), 3.70 (t, *J* = 9.2 Hz, 1H, C12), 3.68 (dd, *J* = 10.0, 7.8 Hz, 1H, C4), 3.67 – 3.62 (m, 2H, overlapped, C15+C16), 3.57 – 3.51 (m, 1H, C1'), 3.49 – 3.45 (m, 1H, C32), 3.45-3.41 (m, 1H, C13), 2.81 (dd, *J* = 13.5, 4.6 Hz, 1H, C23), 2.16 (s, 3H, Ac), 2.14 (s, 3H, Ac), 2.14 (s, 3H, Ac), 2.13 (s, 3H, Ac), 2.05 (s, 3H, Ac), 2.05 (s, 3H, Ac), 2.04 (s, 3H, Ac), 2.04 (s, 3H, Ac), 2.03 (s, 3H, Ac), 1.94 (s, 3H, Ac), 1.96 – 1.92 (m, 1H, C23), 1.91 (s, 3H, Ac), 1.66 – 1.59 (m, 2H, C2'), 1.27 – 1.24 (m, 18H, dodecyl), 0.88 (t, *J* = 7.1 Hz, 3H, C12'); ^{13}C NMR (151 MHz, $CDCl_3$) δ [171.6, 171.2, 171.0, 170.51 (2C), 170.49, 170.4, 170.2, 170.1, 170.01, 169.98] (Ac), 168.5 (C21), 104.1 (C11), 101.8 (d, $^2J_{FC}$ = 22.9 Hz, C41), 100.8 (d, $^2J_{FC}$ = 22.7 Hz, C1), 100.3 (C31), 98.2 (C22), 92.0 (d, $^1J_{FC}$ = 187.8 Hz, C2), 88.0 (d, $^1J_{FC}$ = 186.7 Hz, C42), 81.9 (C12), 76.1 (C5), 75.5 (C33), 74.7 (C13), 73.9 (C15), 73.8 (d, $^2J_{FC}$ = 17.7 Hz, C3), 73.25 (C14), 73.20 (C26), 71.7 (C35), 71.0 (d, $^2J_{FC}$ = 18.8 Hz, C43), 70.8 (C45), 70.5 (C1'), 69.2 (C4), 69.0 (C34), 68.2 (C24), 68.0 (C28), 67.5 (d, $^3J_{FC}$ = 8.2 Hz, C44), 67.0 (C27), 63.3 (C36), 62.8 (C29), 62.1 (C6), 60.9 (C46), 59.2 (C16), 54.6 (C32), 53.1 (Me), 49.8 (C25), 37.7 (C23), [32.1, 29.84, 29.80, 29.77, 29.74, 29.70, 29.55, 29.49, 26.0] (dodecyl), 23.8 (Ac), 23.3 (Ac), 22.8 (dodecyl), [21.4, 21.0, 20.90, 20.87, 20.81, 20.78, 20.71, 20.70 (2C)] (Ac), 14.3 (C12'); ^{19}F NMR (564 MHz, $CDCl_3$, the signals of the minor conformer are marked with an asterisk) δ -199.51 (ddd, $^2J_{FH}$ = 51.4 Hz and $^3J_{FH}$ = 15.5, 2.7 Hz, C2), -199.53 - -199.56* (m, C2), -206.73* (dd, $^2J_{FH}$ = 51.6 Hz and $^3J_{FH}$ =12.7 Hz, C42), -206.86 (ddt, $^2J_{FH}$ = 51.2 Hz and $^3J_{FH}$ = 13.5, 3.3 Hz, C42); MALDI-TOF MS {matrix = DHB (EtOAc)} found: 1585.87 calcd: 1585.62 for $C_{68}H_{104}F_2N_2NaO_{36}$ $[M+Na]^+$; IR (neat) 3887, 3853, 3814, 3743, 3729, 3628, 3590, 3455, 2959, 2920, 2851, 1751, 1735, 1701, 1654, 1633, 1559, 1537, 1504, 1438, 1368, 1257, 1217, 1027.

Compound 1

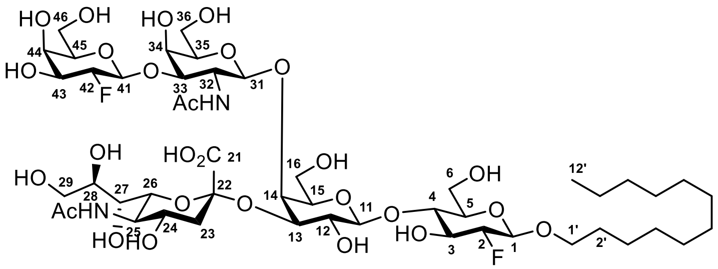

Compound **23** (24.7 mg, 15.8 µmol mmol, 1.00 eq.) was dissolved MeOH (2 mL) and sodium methoxide (42.0 mg, 7.9 µmol, 0.50 eq.) was added at room temperature. After stirring for 2 h, H_2O (0.1 mL) was added and the reaction was stirred for 30 min. The mixture was quenched by Amberlyst® (3 mg) and filtered through cotton wool. The crude material was purified by column chromatography (SiO_2, $CHCl_3$/MeOH/H_2O, 7/2.6/0.4) and obtained as a colorless foam (17.8 mg, 15.2 µmol, 96 %).

1: $[\alpha]_D^{24}$ = +7.4 (c 0.20, $CHCl_3$); ^{1}H NMR (599 MHz, CD_3OD) δ 8.55 (s, 1H, C21, COOH), 8.19 (d, *J* = 8.3 Hz, 1H, NH), 7.19 (d, *J* = 9.9 Hz, 1H, NH), 4.89 (m, C31), 4.73 (dd, *J* = 7.6Hz, 1H, C41), 4.54 (dd, *J* = 7.7 Hz, 1H, C1), 4.44 (d, *J* = 7.9 Hz, 1H, C11), 4.31 (dd, $^2J_{FH}$ = 51.8 Hz and *J* = 9.3 Hz, 1H, C42), 4.19 (m, 1H, C32), 4.14 (m, 1H, C14), 4.05 (m, 1H, C34), 4.01 (m, 1H, C2), 3.94 – 3.81 (m, 9H, overlapped, C13+C44+C1'dodecyl+C6+C29+C16+C24 +C35), 3.80 – 3.65 (m, 11H, overlapped, C36+C3+C28+C43+C46+C36+C15+C16+C33+C25), 3.64 – 3.53 (m, 4H, overlapped, C45+C4+C1'dodecyl+C29), 3.48 – 3.38 (m, 5H, overlapped, C26+C5+C12+C27), 2.73 (dd, *J* = 12.7, 4.89 Hz, 1H, C23), 1.90 (m, 1H, C23), 1.42-1.22 (m, 18H, dodecyl), 1.60 (m, 2H, C2' dodecyl), 0.90 (m, 2H, C12' dodecyl); ^{13}C NMR (151 MHz, CD_3OD) δ 175.7 (Ac), 174.0 (Ac), 175.3 (C21), 104.8 (C11), 104.3 (C31), 104.2 (d,$^2J_{FC}$ = 23.5 Hz, C41), 103.4 (C22), 101.6 (d,$^2J_{FC}$= 22.8 Hz, C1), 93.2 (d,$^1J_{FC}$= 186.6 Hz, C2), 92.8 (d,$^1J_{FC}$ = 182.3 Hz, C42), 83.6 (C33), 80.5 (d,$^3J_{FC}$ = 7.9 Hz, C4), 78.9 (C14), 76.5 (C45), 76.4 (C5), 76.3 (C13), 75.8 (C35), 75.6 (C15), 75.1 (C26), 74.8 (d,$^2J_{FC}$ = 17.4 Hz, C3), 73.38 (C28), 73.35 (d,$^2J_{FC}$ = 17.2 Hz, C43) 71.0 (C12), 70.88 (d,$^3J_{FC}$ = 11.2 Hz, C44), 70.86 (C1' dodecyl), 70.5 (C27), 69.66 (C34), 69.66 (C24), 65.3 (C29), 63.0 (C36), 62.2 (C46), 61.8 (C16), 61.6 (C6), 53.8 (C25), 52.1 (C32), 38.6 (C23), [33.1, 30.75, 30.73, 30.69 (C2'), 30.68, 30.49, 30.44, 27.03, 23.7] (dodecyl), 14.4 (C12' dodecyl); ^{19}F NMR (564 MHz, CD_3OD) δ -200.52 (ddd, $^2J_{FC}$ = 51.4 Hz and $^3J_{FC}$ = 15.9 Hz C2), -208.52 (dd, $^2J_{FC}$ = 51.9 Hz and $^3J_{FC}$ = 14.7 Hz); MALDI-TOF MS {matrix = DHB (EtOAc)} found: 1193.56 calcd: 1193.51 for $C_{49}H_{84}F_2N_2NaO_{27}$ $[M+Na]^+$; IR (neat) 697, 756, 803, 892, 1026, 1112, 1262, 1315, 1351, 1376, 1434, 1596, 1736, 2853, 2923, 3276.

Variable Temperature (VT) NMR Experiments

To investigate the relationship (diastereomers vs. conformers) between the two species (7:3 mixture) observed in the ^{19}F NMR spectrum of compound **22** in $CDCl_3$, variable temperature NMR studies were performed on an *Agilent DD2 500* NMR spectrometer. $^{19}F\{^1H\}$ NMR spectra in $CDCl_3$ were recorded in 5 °C intervals in the range of -20 °C to 60 °C (**Figure S1**).

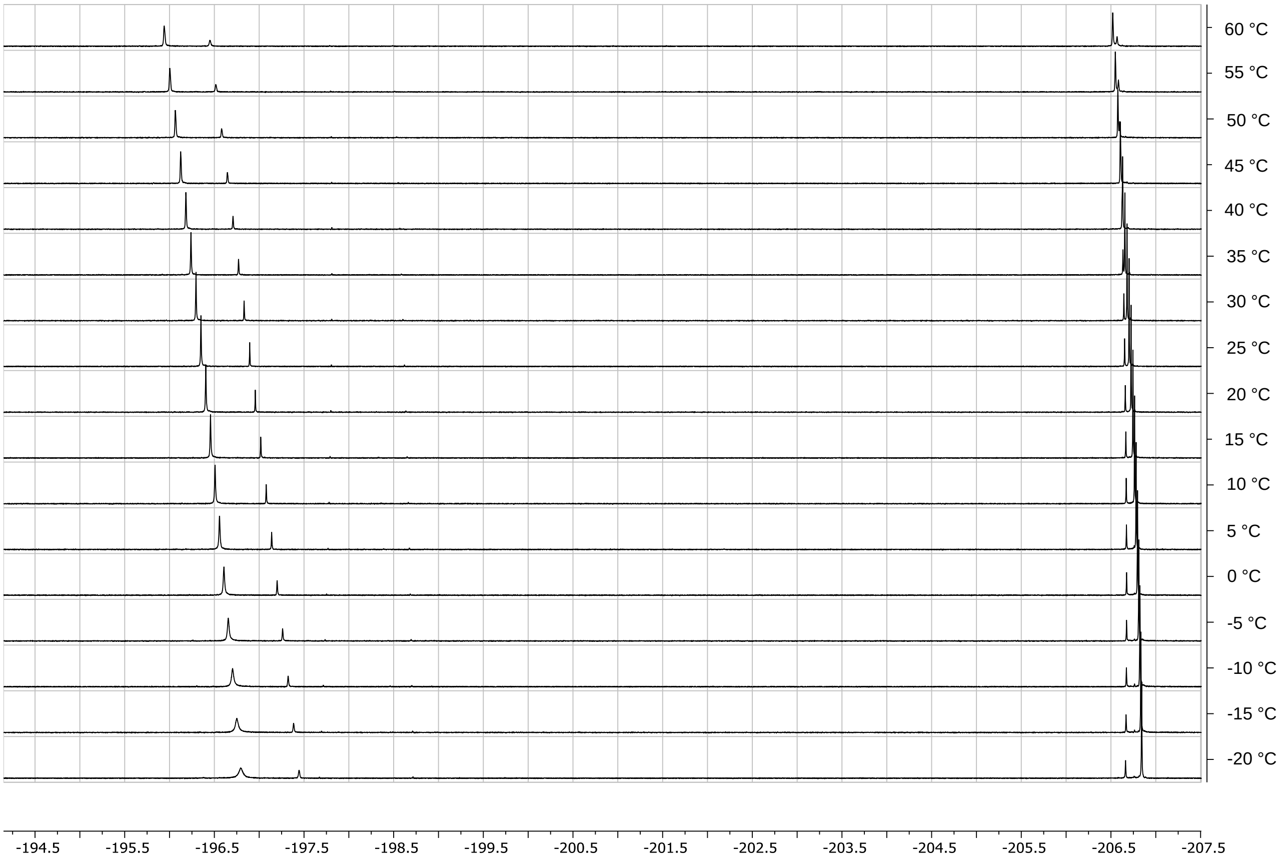

Figure S1. VT-$^{19}F\{^1H\}$ NMR spectra of pentasaccharide **22** in $CDCl_3$ in the temperature range of -20 °C to 60 °C.

For the signal of the fluorinated galactose moiety (δ ca. -206 ppm, see **Figure S2**), two distinct effects can be observed in the NMR spectrum.

(1) The chemical shifts of the major and the minor species are temperature dependent. The downfield shift is much more pronounced for the major species, leading to a crossing of both resonances at a temperature of 45 °C. At higher temperatures, the energy difference (shift difference in Hz) between both conformers in $CDCl_3$ solution increases again.

(2) The lineshape is temperature dependent, with a broadening of the resonances at temperatures above 45 °C. This dynamic behavior is also observed for the resonance of the fluorinated glucose moiety (**Figure S3**) and indicates that the two observed species are conformers, exchanging faster at higher temperature, rather than diastereomers.

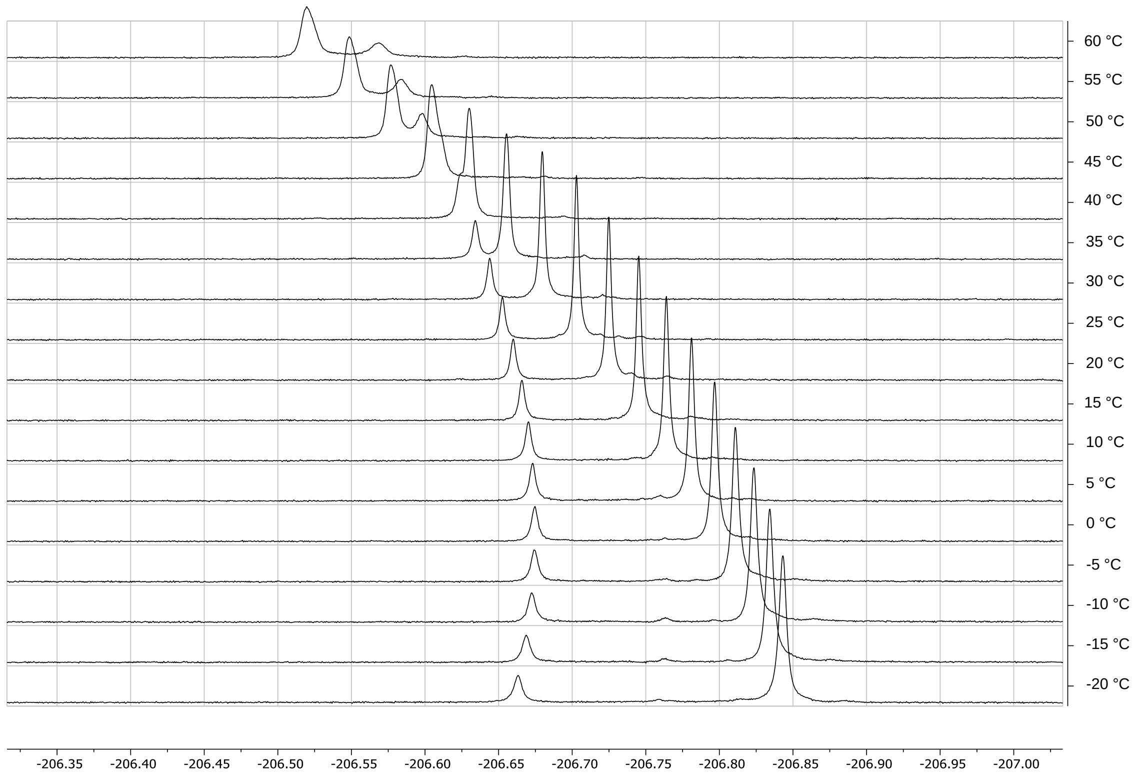

Figure S2. VT-$^{19}F\{^1H\}$ NMR spectra of the fluorinated galactose moiety of compound **22** in $CDCl_3$ in the temperature range of -20 °C to 60 °C.

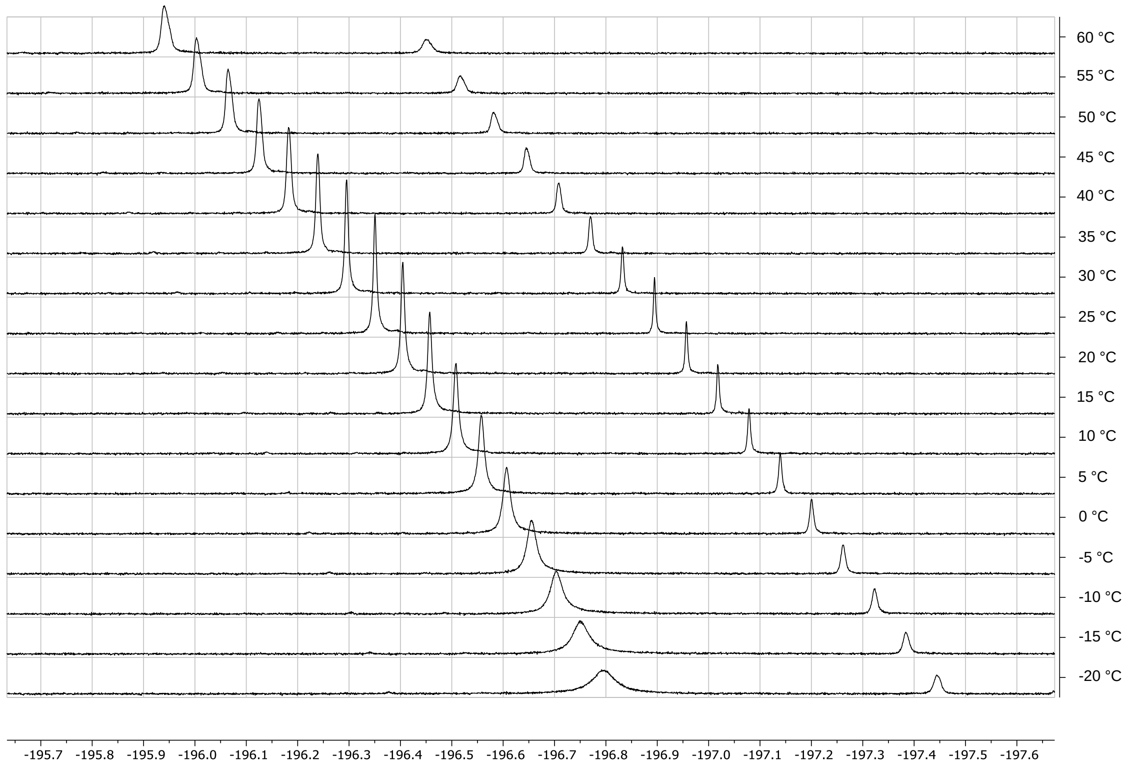

Figure S3. VT-$^{19}F\{^1H\}$ NMR spectra of the fluorinated glucose moiety of compound **22** in $CDCl_3$ in the temperature range of -20 °C to 60 °C.

To investigate this effect further, pentasaccharide **22** was dissolved in toluene-d8 and subjected to VT-NMR spectroscopy in the range of -60 °C to 90 °C in 10 °C intervals (**Figure S4**, **Figure S5** and **Figure S6**). Between -60 °C and 30 °C, the resonances get expectedly sharper at higher temperature, and two distinct species can be identified in a 8:2 ratio at 30 °C. At temperatures above 40 °C, the resonances get broader again (**Figure S5**, **Figure S6**) reflecting the dynamic behavior of the system already observed in $CDCl_3$. At 90 °C, the resonances of the minor conformer can hardly be identified anymore (**Figure S5**). However, within the temperature range investigated herein, both conformers do not exchange rapidly on the NMR timescale, as the signals do not get sharper again at higher temperature.

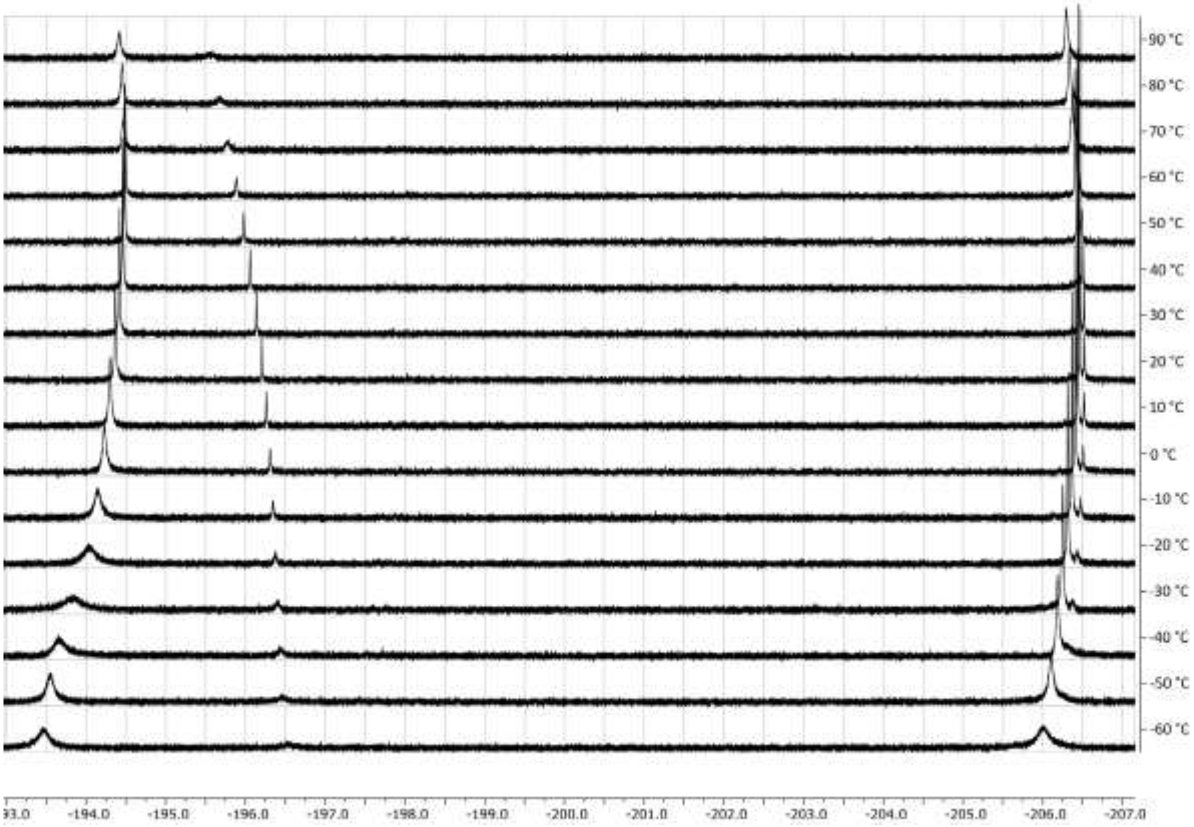

Figure S4. VT-$^{19}F\{^1H\}$ NMR spectra of pentasaccharide **22** in toluene-d8 in the temperature range of -60 °C to 90 °C.

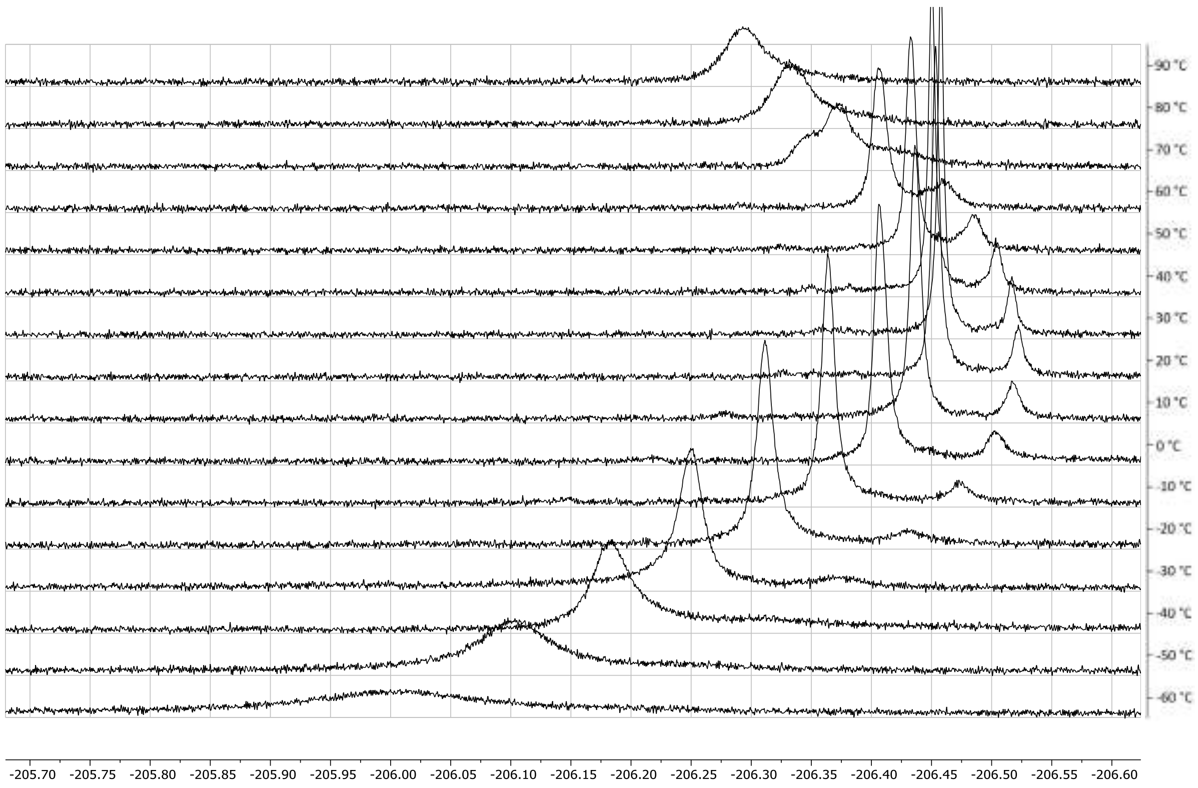

Figure S5. VT-^{19}F{^{1}H} NMR spectra of the fluorinated galactose moiety of compound **22** in toluene-d8 in the temperature range of -60 °C to 90 °C.

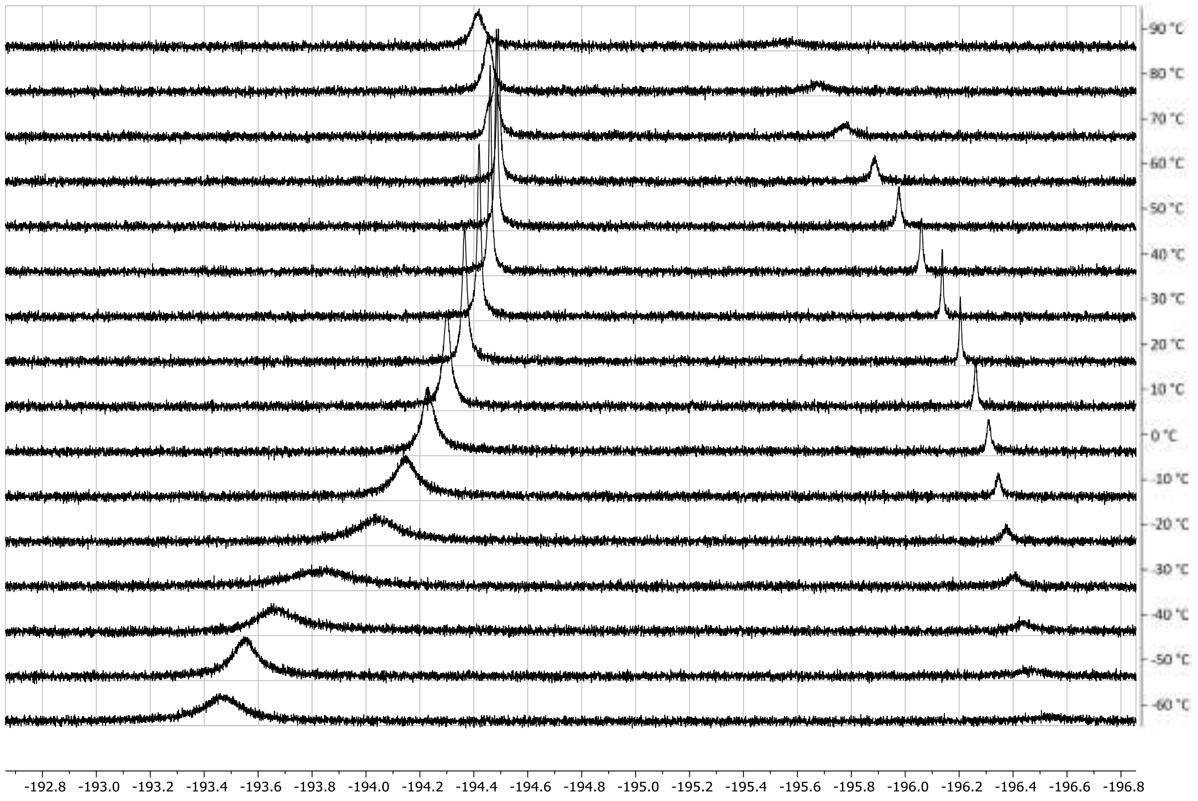

Figure S6. VT-^{19}F{^{1}H} NMR spectra of the fluorinated glucose moiety of compound **22** in toluene-d8 in the temperature range of -60 °C to 90 °C.

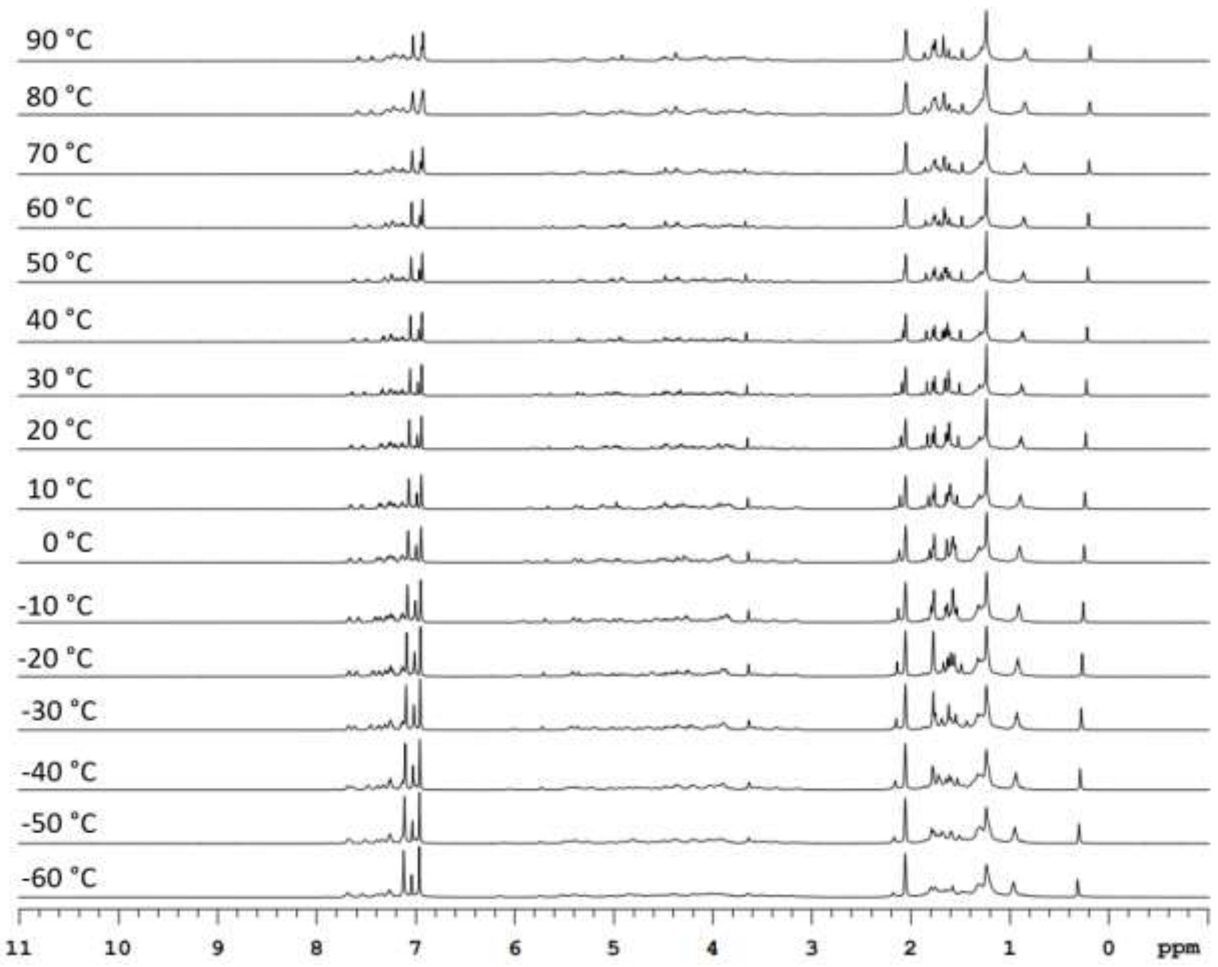

Figure S7. Overlay of ^{1}H NMR spectra in toluene-d_8 of compound **22** measured at different temperatures (-60 to +90 °C) do not provide the same level of information about the dynamic behaviour as the VT-^{19}F{^{1}H} NMR.

NMR Experimental Data (Competition Experiments)

NMR samples contained F-GM1 and CTB in a buffer consisting of 10 mM HEPES pH 7.4, 150 mM NaCl, 10% D_2O, 50 μM TFE and 100 μM DSS. Spectra were acquired at 298 K on a Bruker AVIIIHD 600 MHz spectrometer equipped with a QCI-F CryoProbe™ using a pulse-acquire experiment with ^{1}H decoupling. 512 scans were acquired in 22 min for each spectrum and the resulting FIDs were processed with exponential apodisation with 2 Hz line broadening, and using the apbk command from the Topspin software (Bruker). Data were analyzed as described by Dalvit *et. al*.[8]

Experimental Procedures Crystallization

Cloning, Expression and Purification of CTB

ctb (Uniprot ID P01556; classical biotype) was amplified from the plasmid pET21b_CTB (Genscript) by PCR (primers from Eurofins Genomics and are given in Table 1) and cloned into pET26b (Novagen) using Gibson Assembly. The sequence of this construct, pET26b CTB, was verified and the plasmid transformed into *E. coli* Lemo21 (DE3) cells using a standard heat shock protocol.

A single colony was transferred into LB medium supplemented with 50 µg mL^{-1} kanamycin and 34 µg mL^{-1} Chloramphenicol and incubated over night at 37 °C shaking at 180 rpm. This culture was then used to inoculate (1 to 100) fresh LB medium supplemented with the same antibiotics and grown at 37 °C until the optical density (OD600) reached 0.5. Protein expression was induced by the addition of 0.5 mM IPTG at a shaker temperature of 25 °C and the cultures were grown overnight at 200 rpm.
The culture medium was cleared by centrifugation (7,500 x rpm, 37 °C, 90 min) and the supernatant subsequently cleared by filtration using a 0.45 µm filter. The flow-through was applied to a 5 mL His-Trap FF column (GE Healthcare) preequilibrated in IMAC Buffer A (20 mM Tris pH 8, 500 mM NaCl) at 3 mL min^{-1}. After extensive washing (20 column volumes), CTB was eluted using IMAC Buffer B (20 mM Tris pH 8, 500 mM NaCl, 150 mM Imidazole). Peak fractions were collected and analyzed by SDS-PAGE. All fraction containing CTB were pooled and concentrated before injection onto a Superdex 200 16/600 size exclusion column (GE Healthcare) pre-equilibrated in SEC buffer (20 mM Tris pH 7.5, 200 mM NaCl) at a flow rate of 1 mL min^{-1}. The purity of CTB was confirmed by SDS-PAGE. The final concentration of CTB was adjusted to 430 µM by using a 10k molar mass cutoff filter (ThermoFisher), and the protein concentration determined using photometric analysis (Nanodrop 2000, Thermo Scientific). The protein was aliquoted into 40 µL aliquots, flash frozen in liquid nitrogen and stored at – 80 °C.

Crystallization, Data collection and Structure determination of CTB complexes

Protein crystallization required a concentration of 430 µM CTB, but addition of F-GM1, not GM1, led to severe protein aggregation at CTB concentrations above 100 µM, irrespective of the method used (e.g. starting at CTB concentrations well below 100 µM and concentrating the CTB-F-GM1 complex carefully via dialysis or filtration, or adding F-GM1 to concentrated CTB). To still be able to obtain structural information, CTB (430 µM) was incubated with 4.3 mM F-GM4 at room temperature for 4 h and the complex subsequently crystallized. The much smaller F-GM4 ganglioside is a disaccharide that was prepared according to a published protocol.[9]

CTB-F-GM4 crystals were observed after 3 days in 0.1 M MES/Imidazole pH 7.5, 0.03 M $MgCl_2$, 0.03 M $CaCl_2$, 16% PEG 1,000, 12% PEG 3,350 and 10% MPD (crystallization buffer). These crystals were then harvested and washed several times in fresh drops of crystallization buffer to remove all free F-GM4. Crystals were then transferred into drops comprised of crystallization buffer supplemented with 1 mM F-GM1 to replace solvent-accessible F-GM4 in the crystals. Crucially, there is no part of the F-GM4 disaccharide overlapping with the fluorinated D-galactose when bound to CTB, which makes the interpretation of the electron density map in this essential area unambiguous. Collecting hundreds of datasets finally identified soaking for 20 min at room temperature, followed by cryoprotection in crystallization buffer supplemented with 1 mM F-GM1 and 25% MPD as the best compromise between ligand replacement and intact diffraction patterns. Datasets were collected at the Deutsches Elektronen Synchrotron (DESY), beamline P11 and the highest-occupancy (F-GM1) dataset manually selected following molecular replacement and refinement of 37 structures.

Diffraction data were processed using AIMLESS[10] in CCP4[11] (Program suite V8.0.016) and molecular replacement carried was out with PHASER[12] using PDB ID: 1PZJ as a search model. The solution was manually rebuilt with COOT[13] (Verison 0.9.8.92 under XQuartz 11 version 2.8.5) and refined using PHENIX[13] (Version 1.21-5190). The structure was validated with MolProbity[15], and all structure images were created with PyMOL (The PyMOL Molecular Graphics System, Version 2.5.7, Schrödinger, LLC).

Table 1. List of primers used in this study.

Primer name	Primer sequence 5'-3'	Application

CTB_F	ACTCCGCAGAACATCAC	Cloning of CTB
CTB_R	ATTAGCCATGCTGATGGC	Cloning of CTB
CTB_26b_R	GTGATGTTCTGCGGAGTGGCCATCGCCGGCTG	Amplification of pET_26b
CTB_26b_F	GCCATCAGCATGGCTAATTGAGTTGGCTGCTGCCAC	Amplification of pET_26b

Table 2. Crystallographic data of CTB_F-GM1 complex.

	CTB_F-GM1
**PDB ID**	9EWF
**Data collection**	
Space group	$P42_12$
Cell dimensions	
a, b, c (Å)	132.5, 132.5, 113.9
α, β, γ (°)	90.0, 90.0, 90.0
Wavelength (Å)	1.033210
Resolution (Å)	2.10 (2.16-2.10)
R_{sym} or R_{merge}	18.8 (134.4)
$CC_{1/2}$	0.998 (0.667)
$I / \sigma I$	10.6 (1.9)
Completeness (%)	100 (100)
Redundancy	12.8 (12.2)
**Refinement**	
Resolution (Å)	46.85-2.10
No. reflections	59,555
R_{work} / R_{free}	0.183 / 0.221
No. atoms	9,253
Protein	8,140
Ligand/ion	324
Water	789

B-factors	34.72
Protein	33.76
Ligand/ion	47.34
Water	39.52
R.m.s. deviations	
Bond lengths (Å)	0.015
Bond angles (°)	0.74

*1 crystal per structure. *Values in parentheses are for highest-resolution shell.

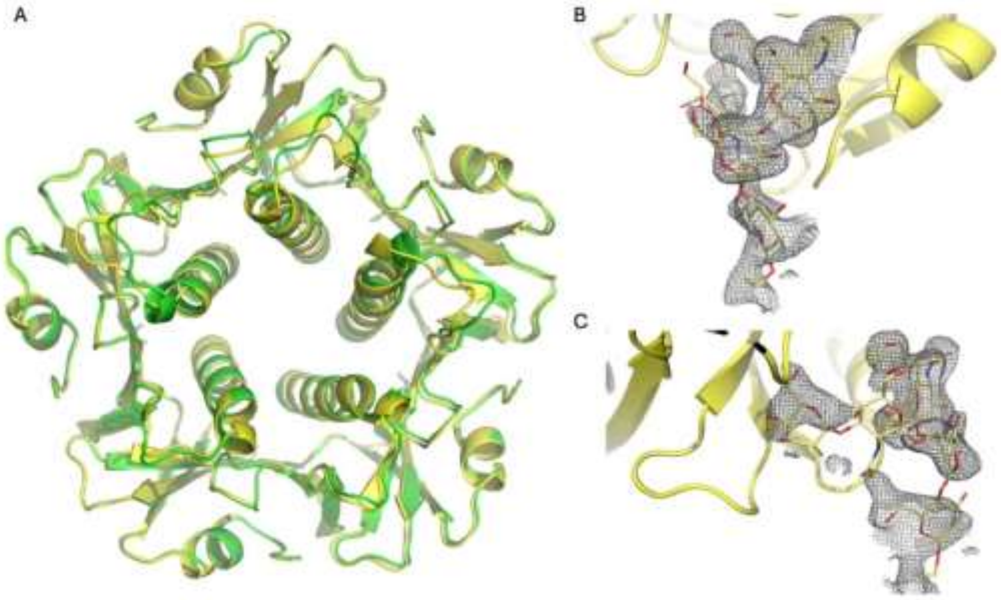

Figure S8. Structural analysis of the CTB_F-GM1 complex. **A** Superposition of the GM1-bound CTB pentamer (PDB ID 2chb, green) with the F-GM1-bound CTB pentamer whose structure was determined in this study (yellow). **B** and **C** POLDER map calculated for the F-GM1 ligand (grey isomesh) contoured at 2.0 σ. The relation of **B** and **C** is a 90 ° counter-clockwise rotation around a vertical axis in the plane of the paper.

References

[1] Ogawa, S.; Senba, S. Preparation of Building Blocks for Carba-Oligosaccharides: Some Protected 5a′-Carba-D-hexopyranosyl-1,5-anhydro-2-deoxy-D-*arabino*-hex-1-enitols, and 5a,5a′-Dicarba Congeners Thereof. *J. Carbohydr. Chem*. **2006**, *25*, 69-93.
[2] Yu, H.; Chen, X. Aldolase-Catalyzed Synthesis of β-d-Gal*p*-(1→9)-d-KDN: A Novel Acceptor for Sialyltransferases. *Org. Lett*. **2006**, *8*, 2393-2396.
[3] Bengtsson, M.; Broddefalk, J.; Dahmén, J.; Henriksson, K.; Kihlberg, J.; Lönn, H.; Srinivasa, B. R.; Stenvall, K. Convergent synthesis of neoglycopeptides by coupling of 2-bromoethyl glycosides to cysteine and homocysteine residues in T cell stimulating peptides. *Glycoconj. J.* **1998**, 15, 223-231.
[4] Cai, S.; Yu, B. Efficient Sialylation with Phenyltrifluoroacetimidates as Leaving Groups. *Org Lett*. **2003**, *5*, 3827-3830.
[5] Adamo, R.; Romano, M. R.; Berti, F.; Leuzzi, R.; Tontini, M.; Danieli, E.; Cappelletti, E.; Cakici, O. S.; Swennen, E.; Pinto, V.; Brogioni, B.; Proietti, D.; Galeotti, C. L.; Lay, L.; Monteiro, M. A.; Scarselli, M.; Costantino, P. Phosphorylation of the synthetic hexasaccharide repeating unit is essential for the induction of antibodies to Clostridium difficile PSII cell wall polysaccharide. *ACS Chem. Biol.* **2012**, *7*, 1420-1428.
[6] Durantie, E.; Bucher, C.; Gilmour, R. Fluorine-Directed β-Galactosylation: Chemical Glycosylation Development by Molecular Editing. *Chem. Eur. J.* **2012**, *18*, 8208-8215.
[7] Frank, R. L. & Smith, P. V. 4-Ethylpyridine. *Org. Synth*. **1955**, *Coll. Vol. 3*, 410.
[8] Dalvit, C.; Fagerness, P. E.; Hadden, D. T. A.; Sarver, R. W; Stockman, B. J. Fluorine-NMR experiments for high-throughput screening: theoretical aspects, practical considerations, and range of applicability. *J. Am. Chem. Soc.* **2003**, *125*, 7696–7703.[9] Kieser, T. J.; Santschi, N.; Nowack, L.; Kehr, G.; Kuhlmann, T.; Albrecht, S.; Gilmour, R. Single Site Fluorination of the GM4 Ganglioside Epitope Upregulates Oligodendrocyte Differentiation *ACS Chem. Neurosci.* **2018**, *9*, 1159-1165.
[10]Evans, P. R. & Murshudov, G. N. How good are my data and what is the resolution? *Acta Crystallogr. D Biol. Crystallogr.* **2013**, *69* (7), 1204–1214.
[11] Collaborative Computational Project, Number 4. The CCP4 Suite: Programs for Protein Crystallography. *Acta Crystallogr. D Biol. Crystallogr.* **1994**, *50* (Pt 5), 760–763.
[12] McCoy, A. J.; Grosse-Kunstleve, R. W.; Adams, P. D.; Winn, M. D.; Storoni, L. C.; Read, R. J. *Phaser* crystallographic software. *J. Appl. Crystallogr.* **2007**, *40* (4), 658–674.
[13] Emsley, P.; Cowtan, K. Coot: model-building tools for molecular graphics. *Acta Crystallogr. D Biol. Crystallogr.* **2004**, *60* (12), 2126–2132.
[14] Liebschner, D.; Afonine, P. V.; Baker, M. L.; Bunkóczi, G.; Chen, V. B.; Croll, T. I.; Hintze, B.; Hung, L.-W.; Jain, S.; McCoy, A. J.; Moriarty, N. W.; Oeffner, R. D.; Poon, B. K.; Prisant, M. G.; Read, R. J.; Richardson, J. S.; Richardson, D. C.; Sammito, M. D.; Sobolev, O. V.; Stockwell, D. H.; Terwilliger, T. C.; Urzhumtsev, A. G.; Videau, L. L.; Williams, C. J.; Adams, P. D. Macromolecular structure determination using X-rays, neutrons and electrons: recent developments in *Phenix. Acta Crystallogr. Sect. Struct. Biol*. **2019**, *75* (10), 861–877.
[15] Chen, V. B.; Arendall, W. B.; Headd, J. J.; Keedy, D. A.; Immormino, R. M.; Kapral, G. J.; Murray, L. W.; Richardson, J. S.; Richardson, D. C. MolProbity: all-atom structure validation for macromolecular crystallography. *Acta Crystallogr. D Biol. Crystallogr.* **2010**, *66* (1), 12–21.

Compound **11** (^{1}H NMR, 400 MHz, CD_2Cl_2)

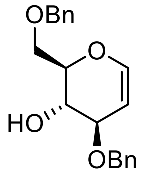

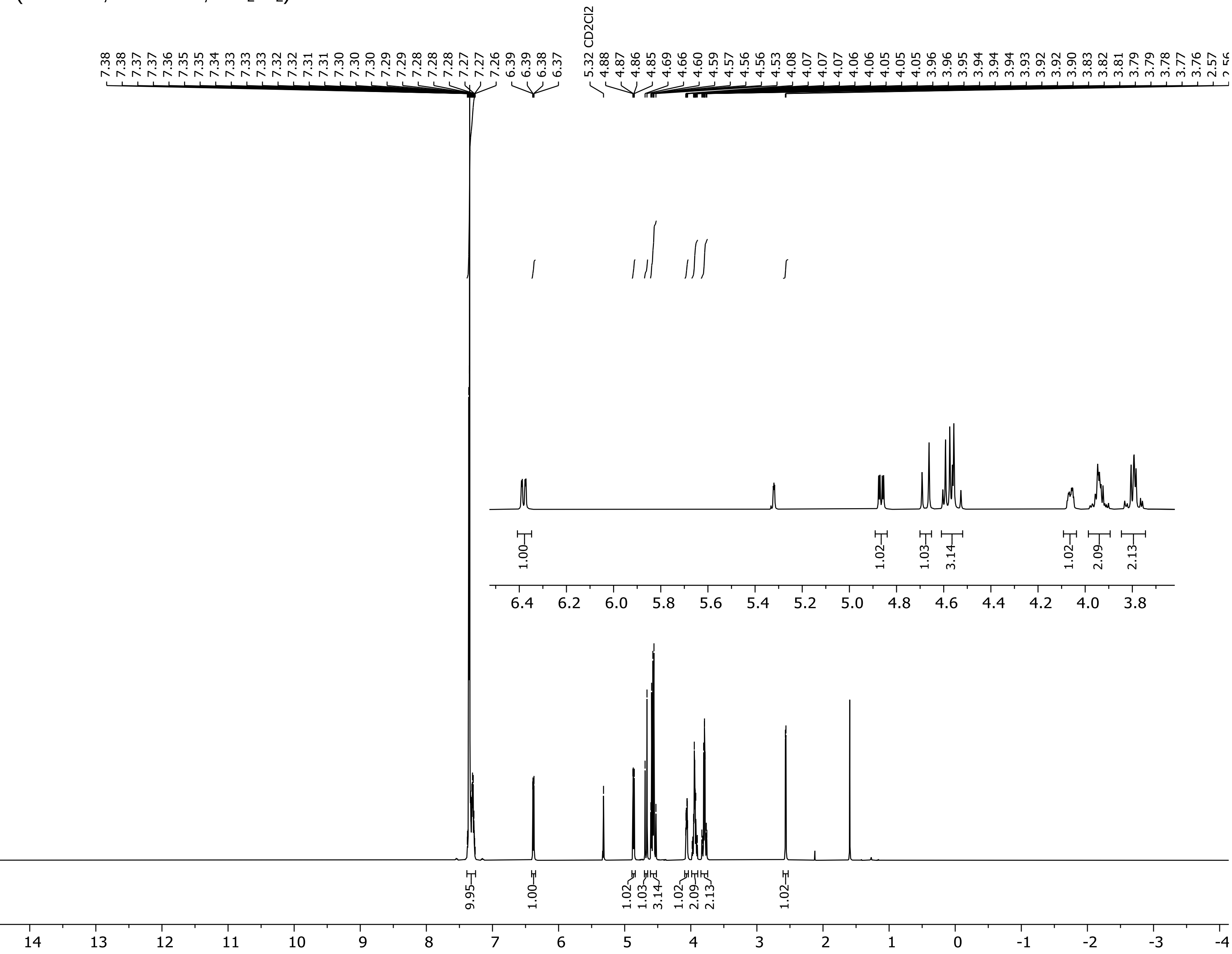

Compound **11** (^{13}C NMR, 101 MHz, CD_2Cl_2)

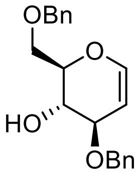

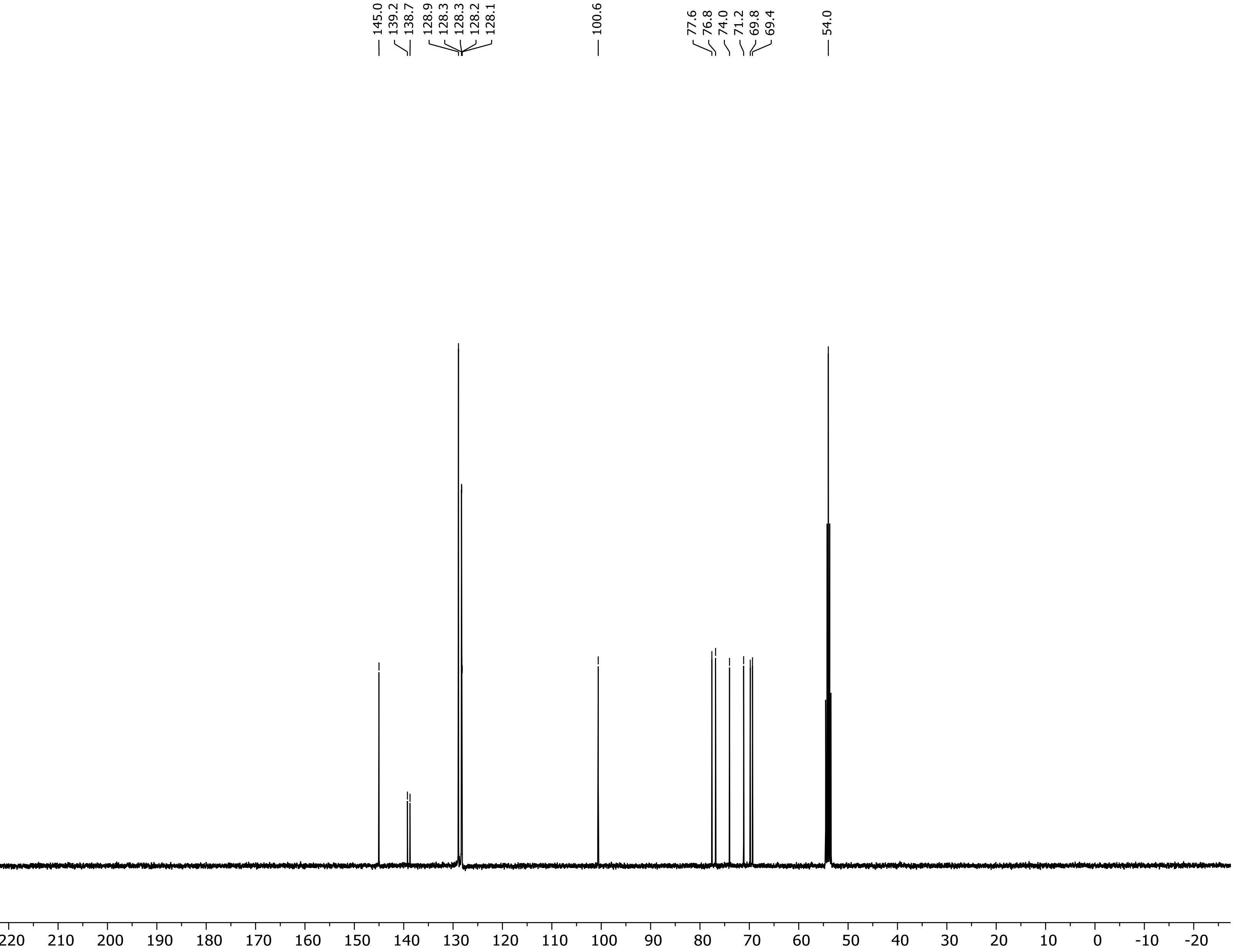

Compound **12** (^{1}H NMR, 599 MHz, CD_2Cl_2, α/β = 57/43)

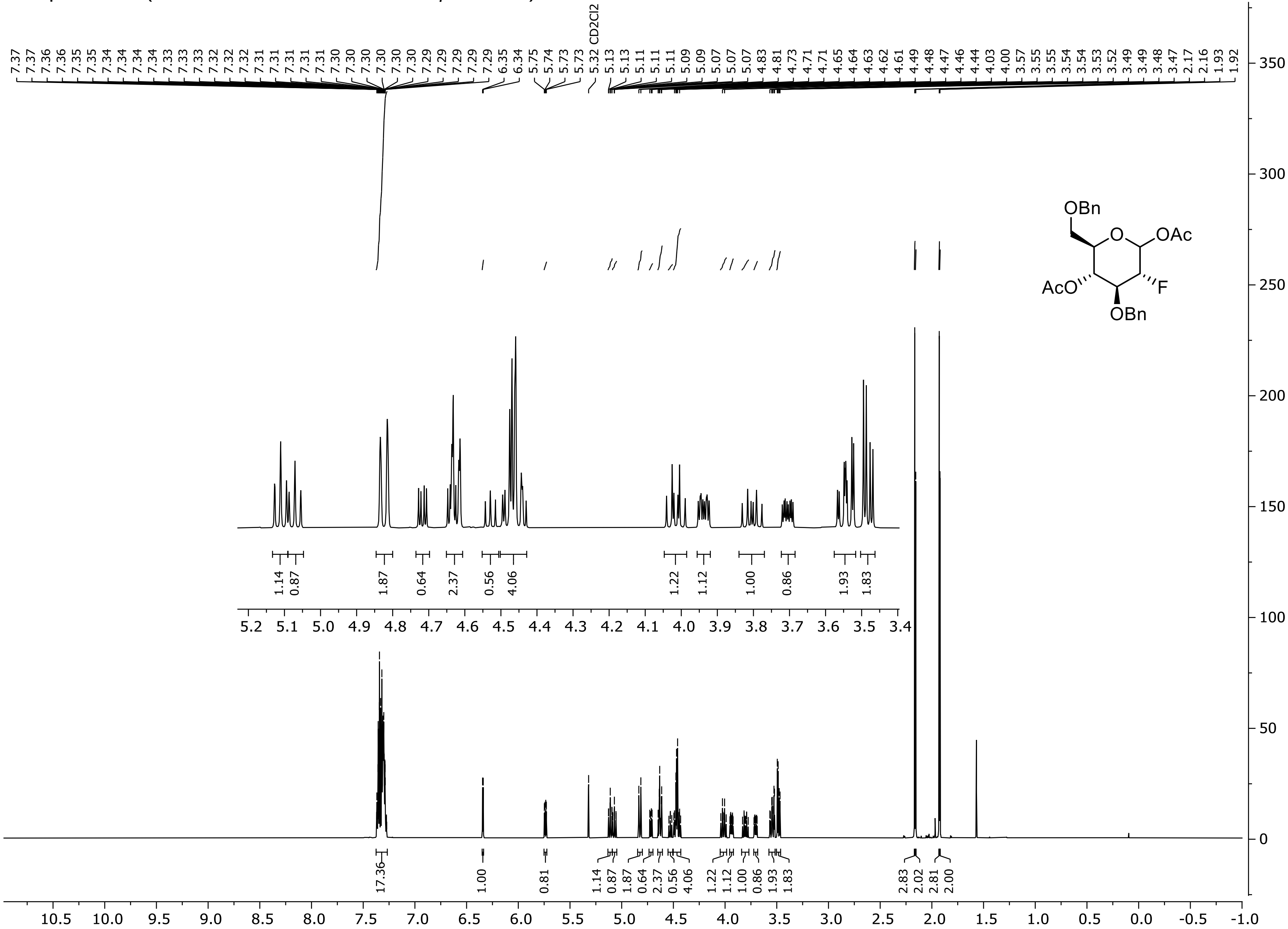

Compound **12** (^{13}C NMR, 151 MHz, CD_2Cl_2, α/β = 57/43)

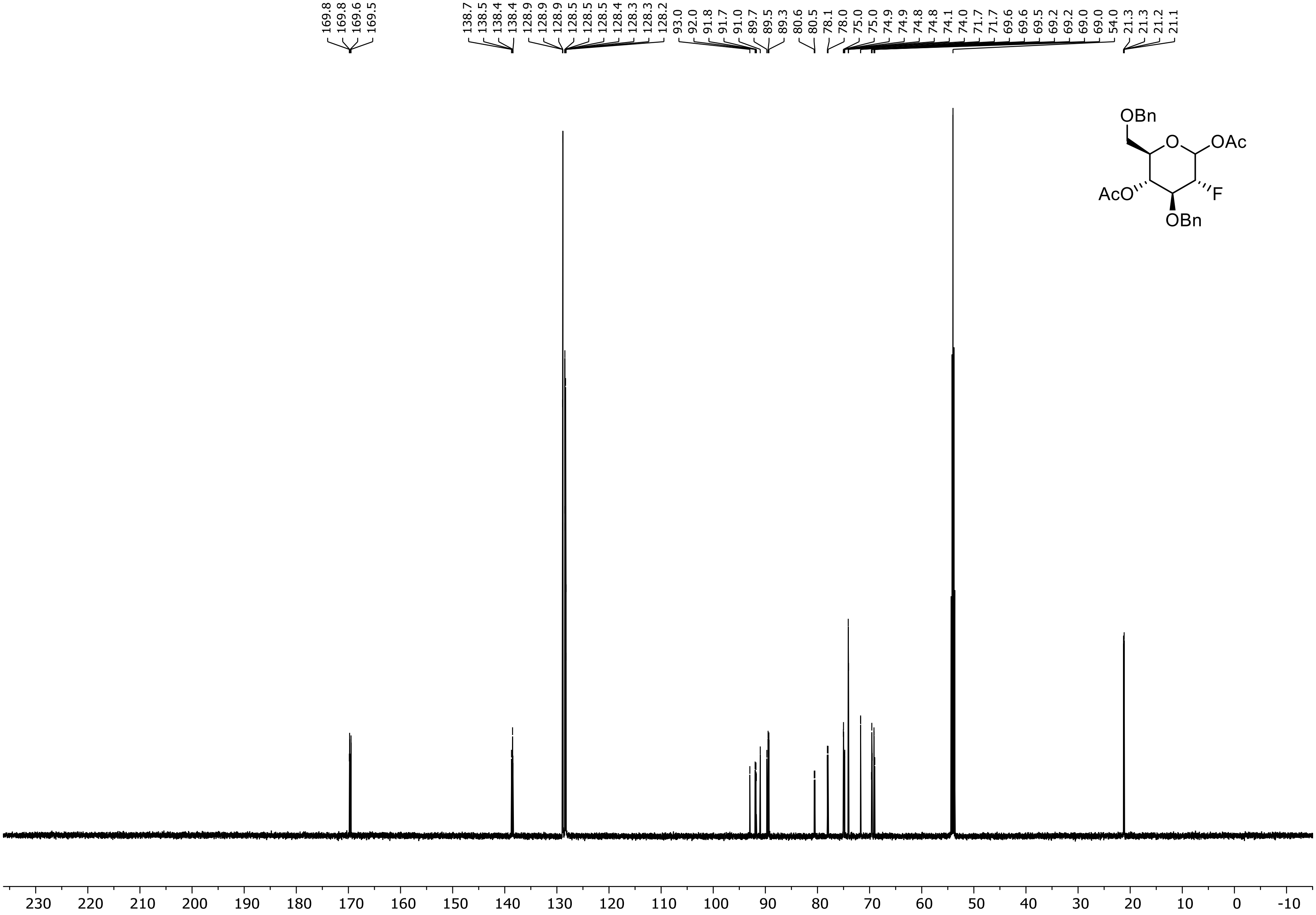

Compound **12** (^{19}F NMR, 564 MHz, CD_2Cl_2, α/β = 57/43) and (1) $^{19}F\{^1H\}$ and (2) ^{19}F NMR (564 MHz)

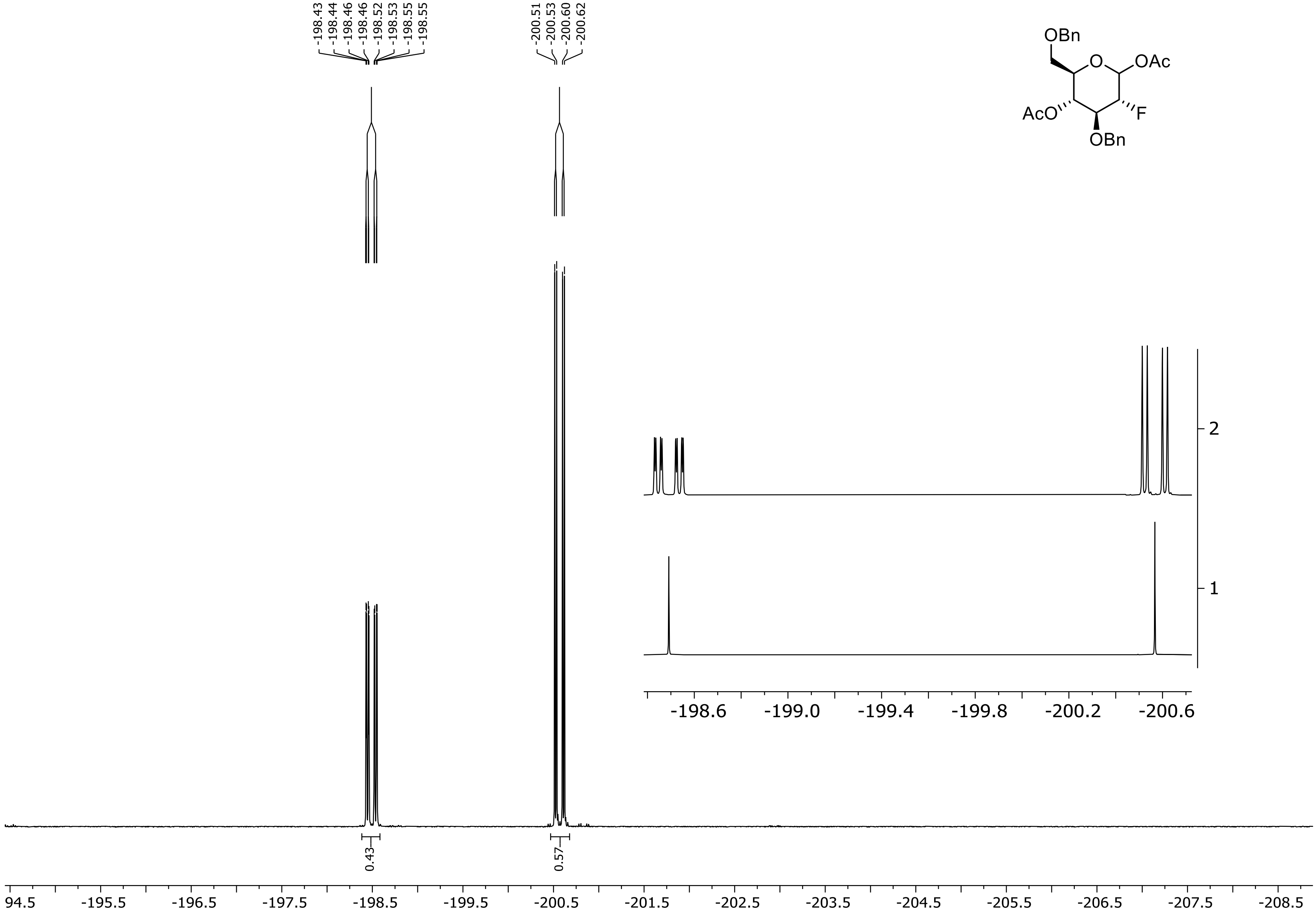

Compound **13** (^{1}H NMR, 599 MHz, CD_2Cl_2)

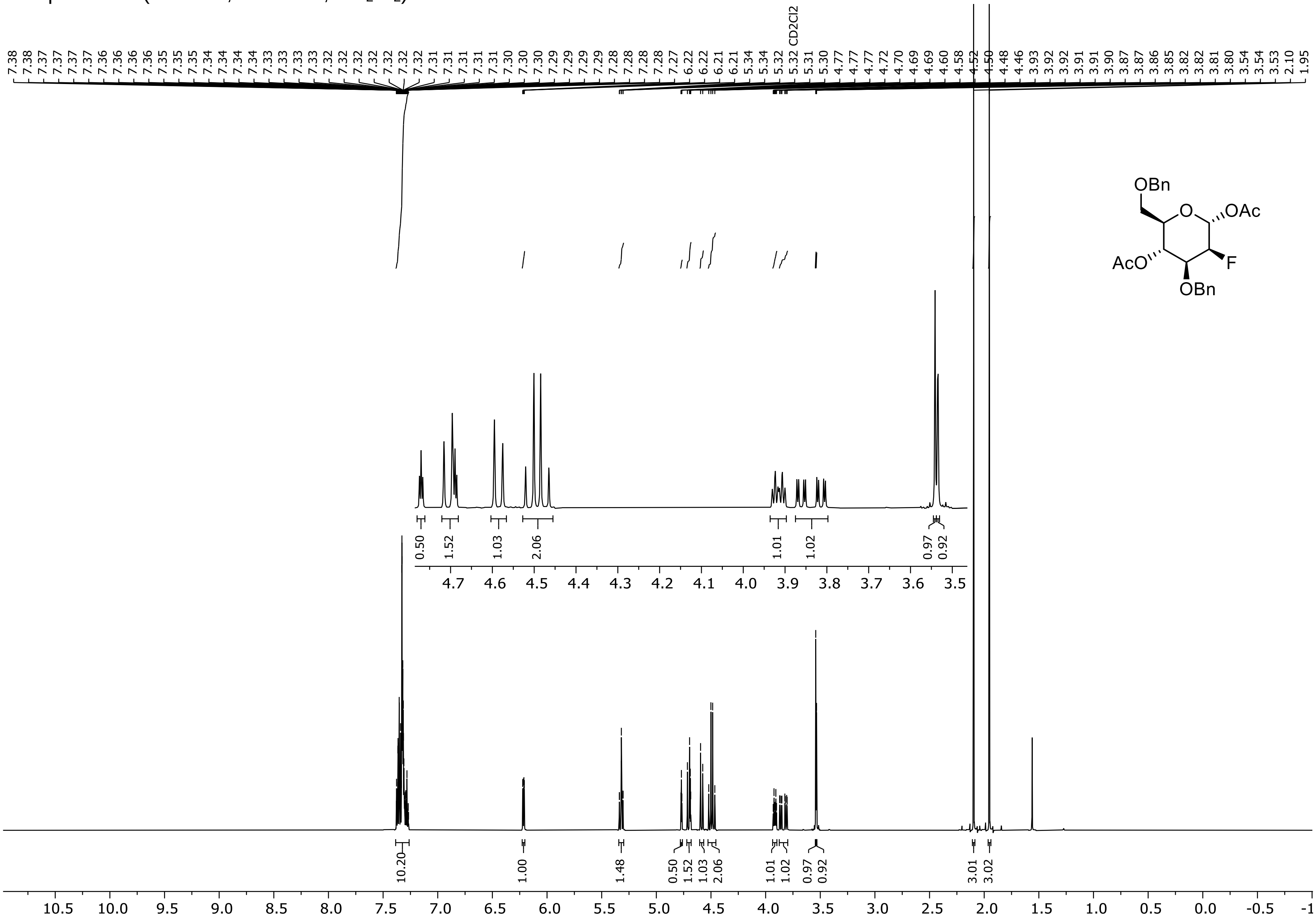

Compound **13** (^{13}C NMR, 151 MHz, CD_2Cl_2)

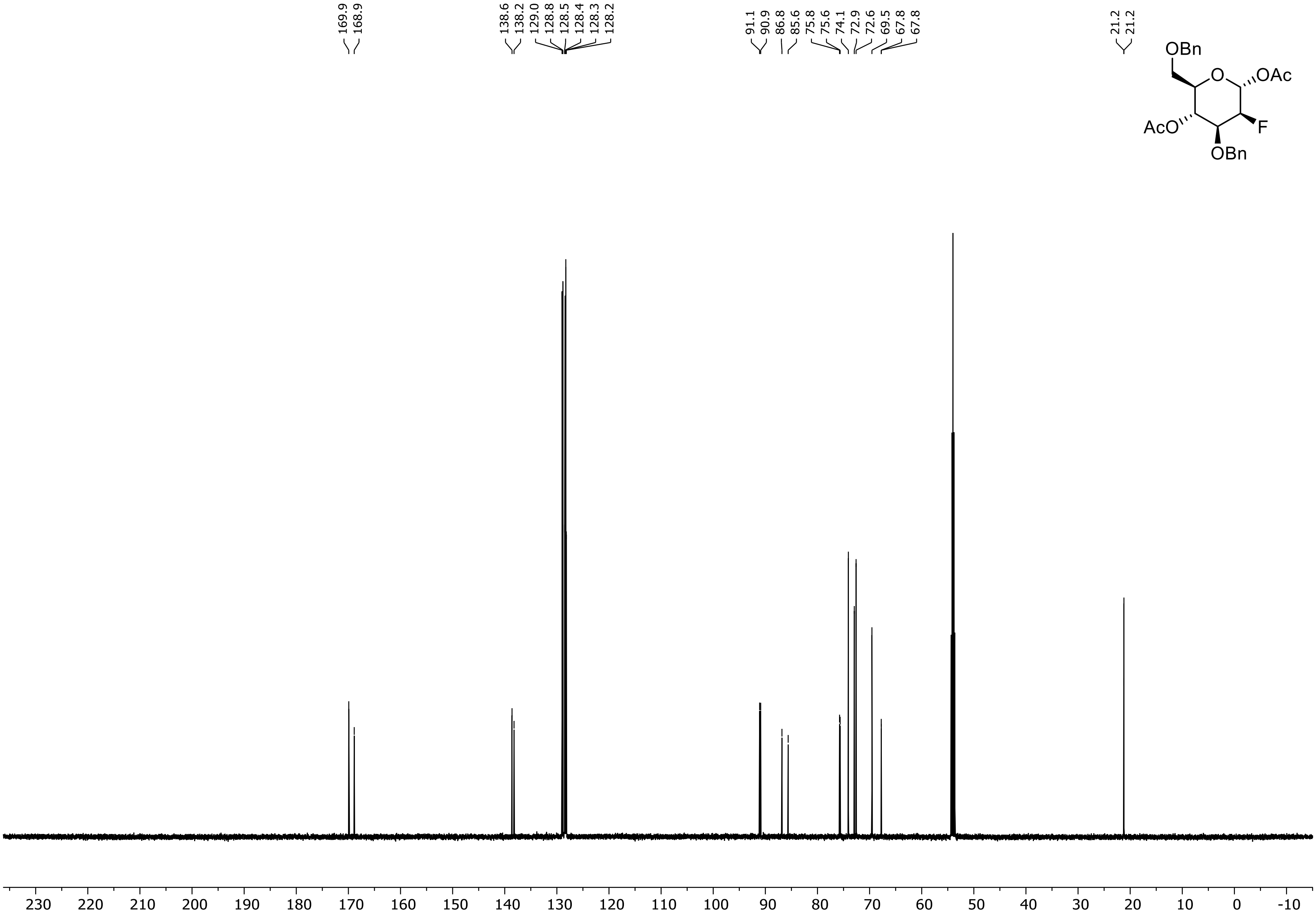

Compound **13** (^{19}F NMR, 564 MHz, CD_2Cl_2) and (1) $^{19}F\{^1H\}$ and (2) ^{19}F NMR (564 MHz)

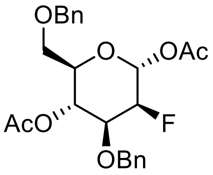

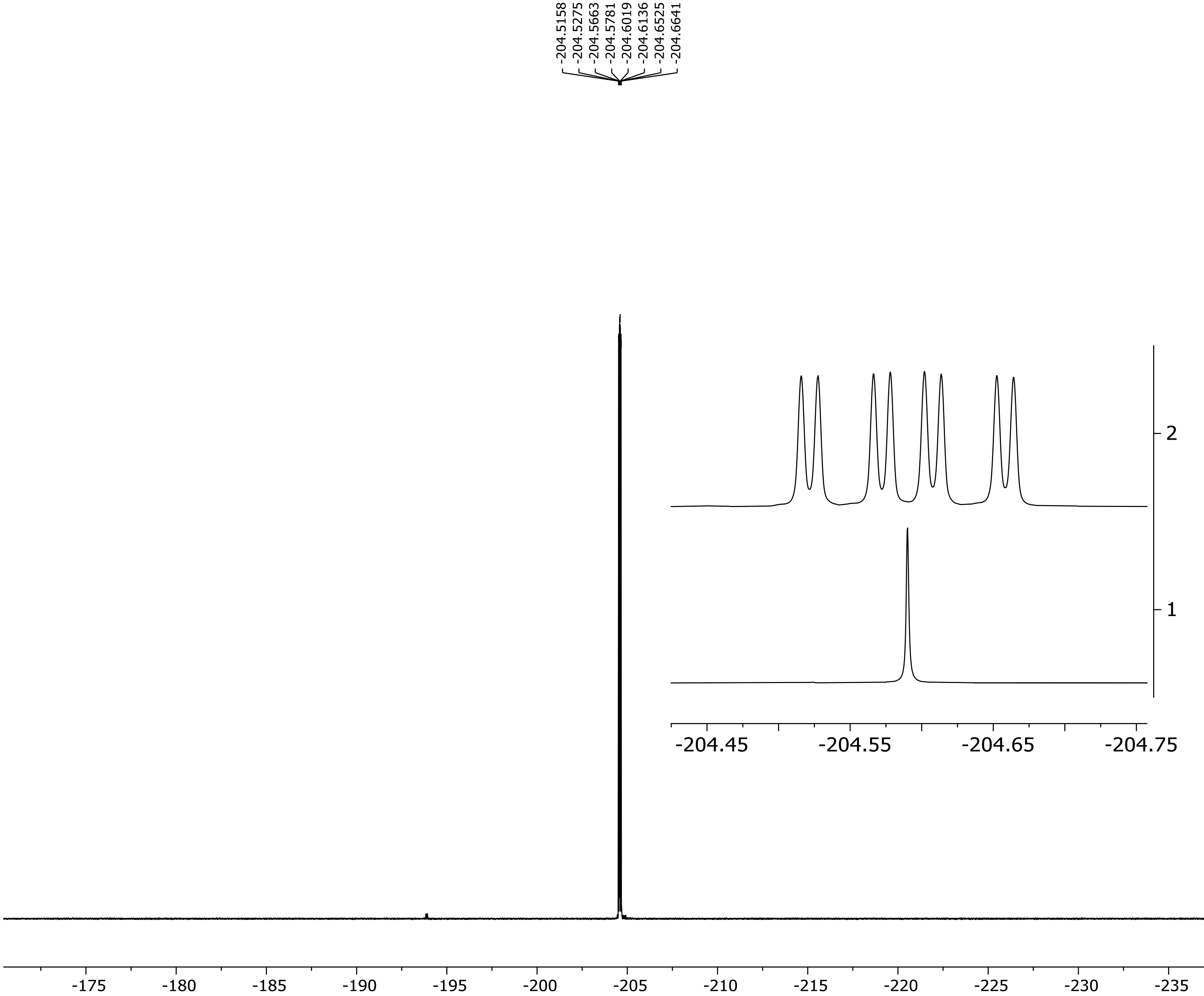

Compound **S1** (^{1}H NMR, 599 MHz, $CDCl_3$, α/β = 75/25)

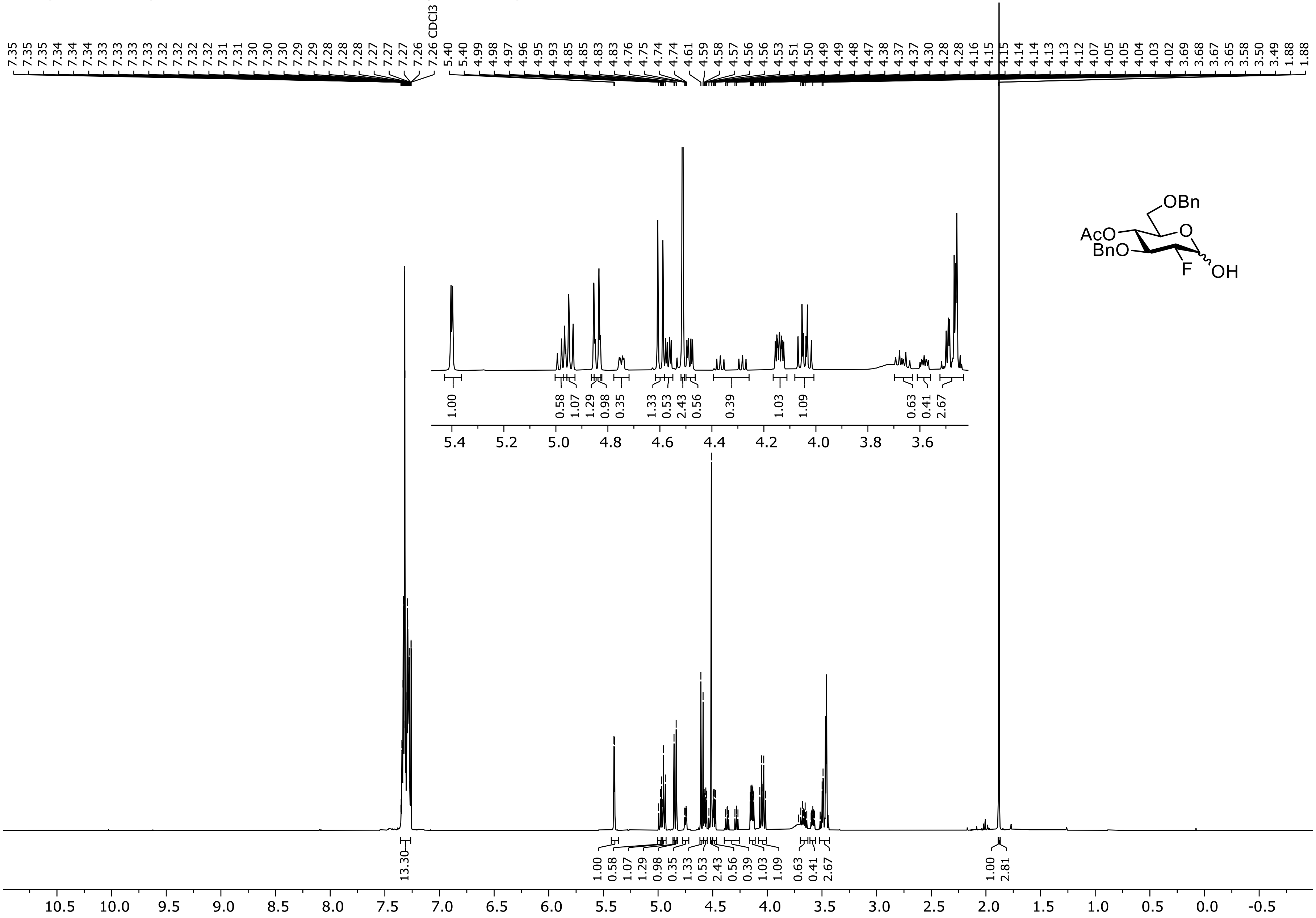

Compound **S1** (^{13}C NMR, 151 MHz, $CDCl_3$, α/β = 75/25)

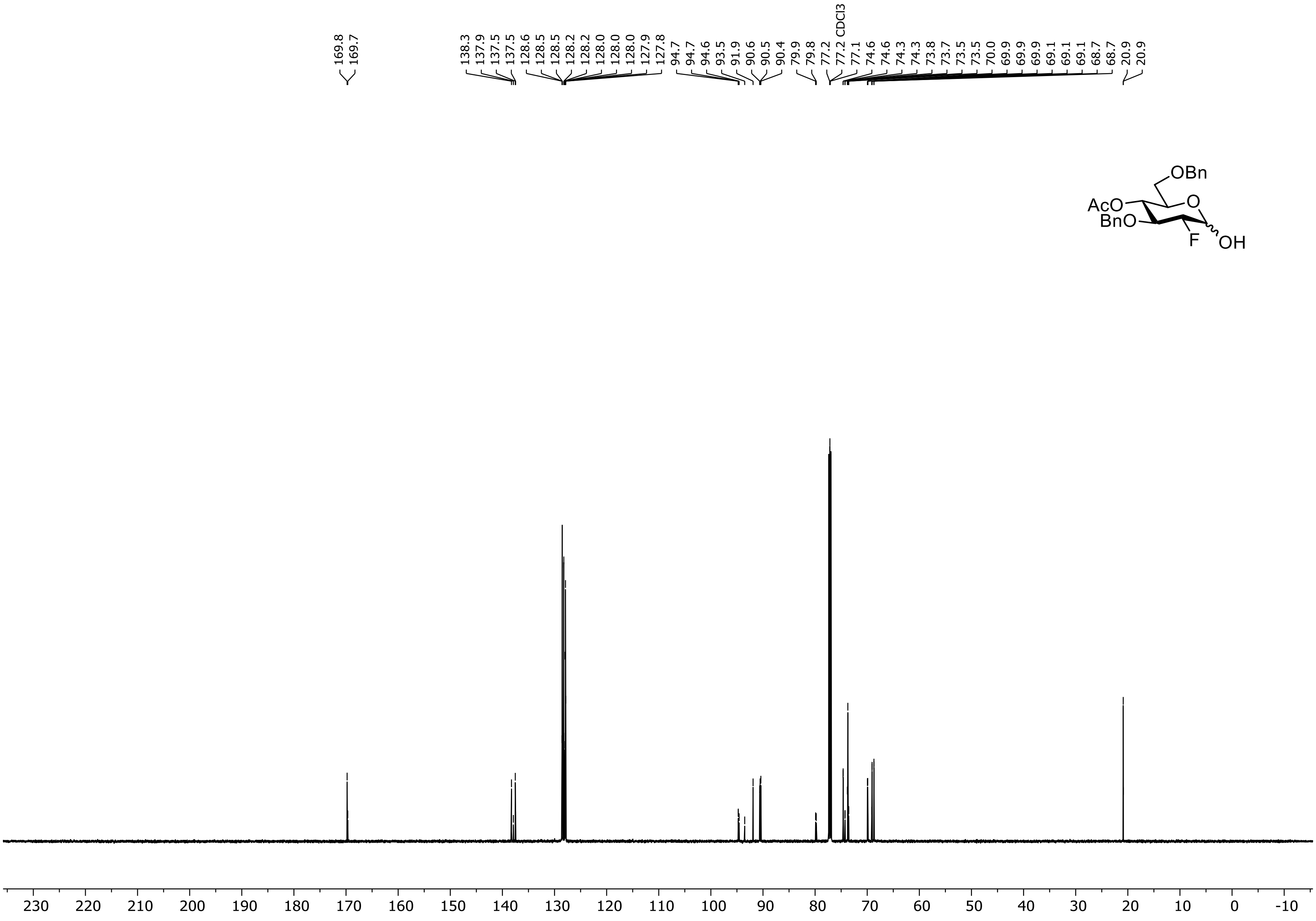

Compound **S1** (^{19}F NMR, 564 MHz, $CDCl_3$, α/β = 75/25) and (1) ^{19}F{^{1}H} and (2) ^{19}F NMR (564 MHz)

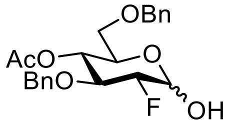

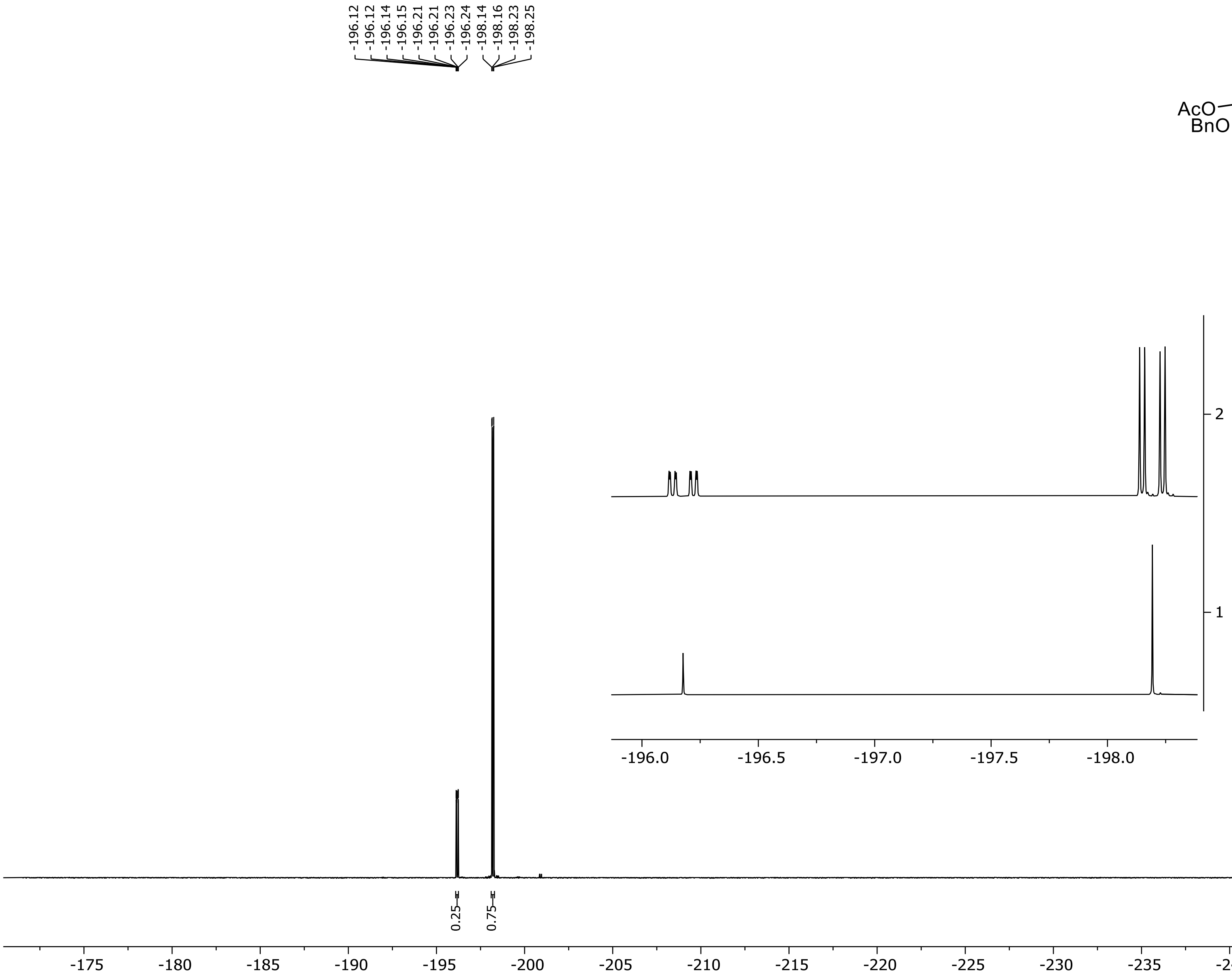

Compound **14** (^{1}H NMR, 599 MHz, CD_2Cl_2)

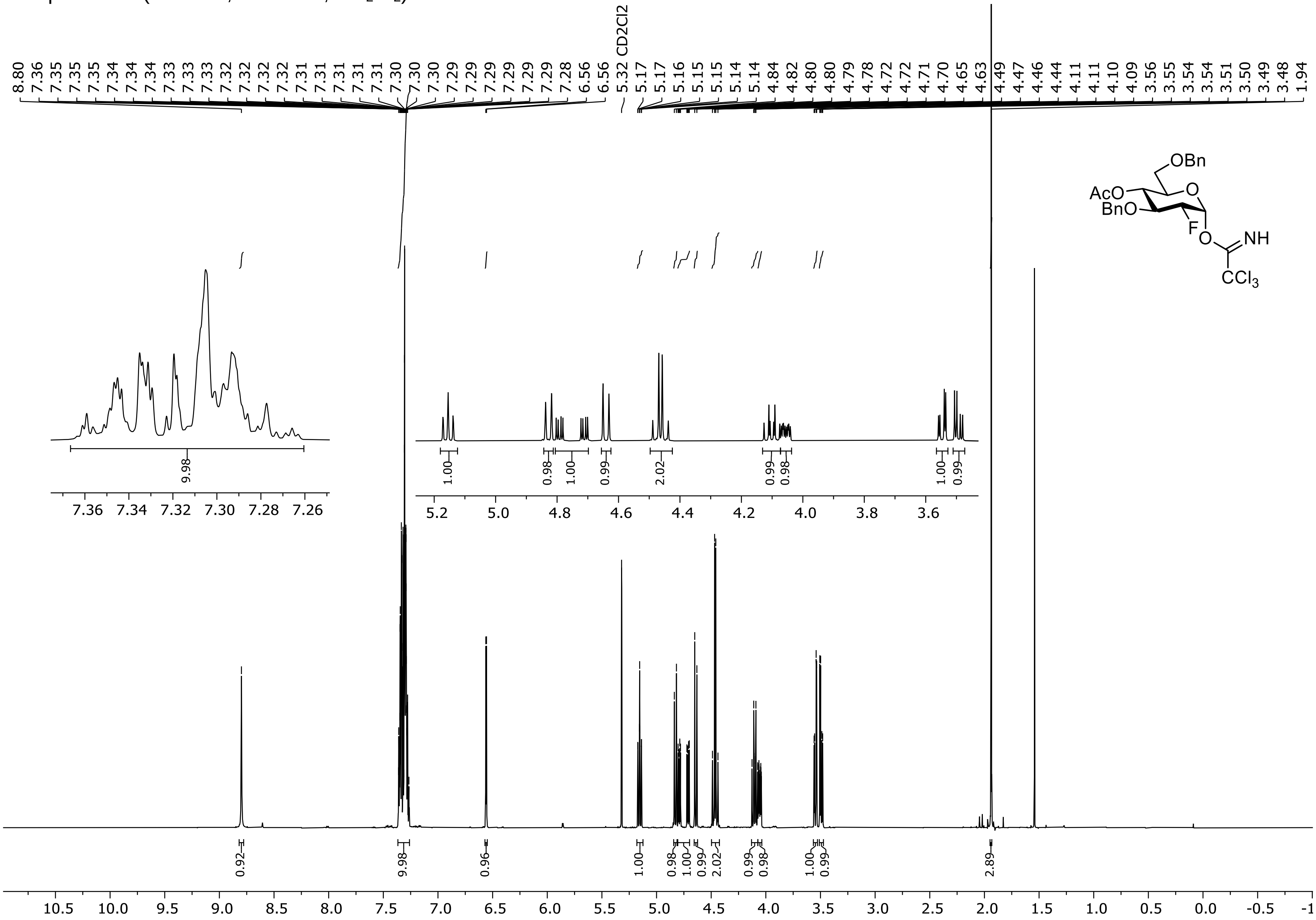

Compound **14** (^{13}C NMR, 151 MHz, CD_2Cl_2)

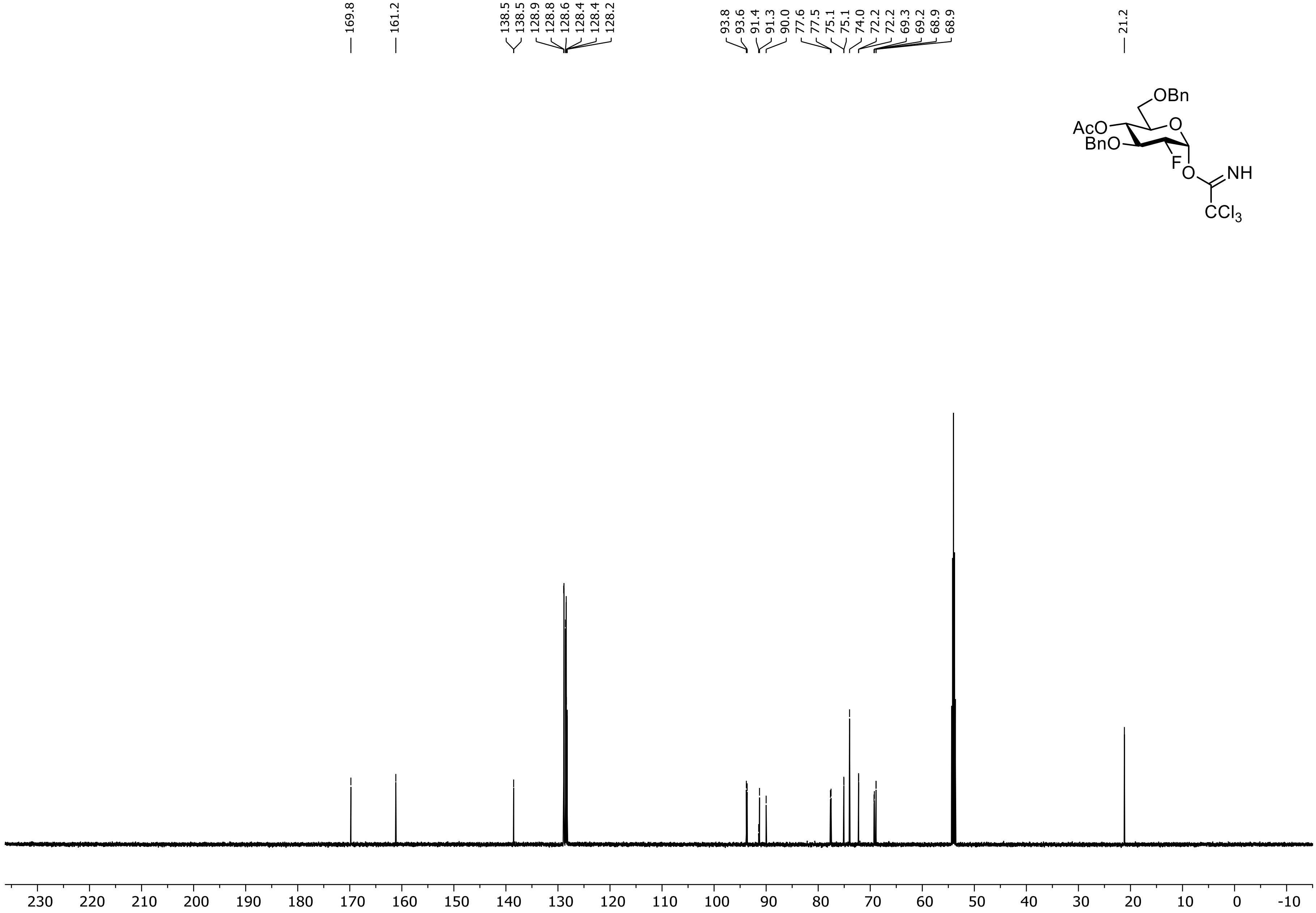

Compound **14** (^{19}F NMR, 564 MHz, CD_2Cl_2) (1) $^{19}F\{^1H\}$ and (2) ^{19}F NMR (564 MHz)

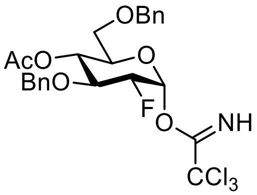

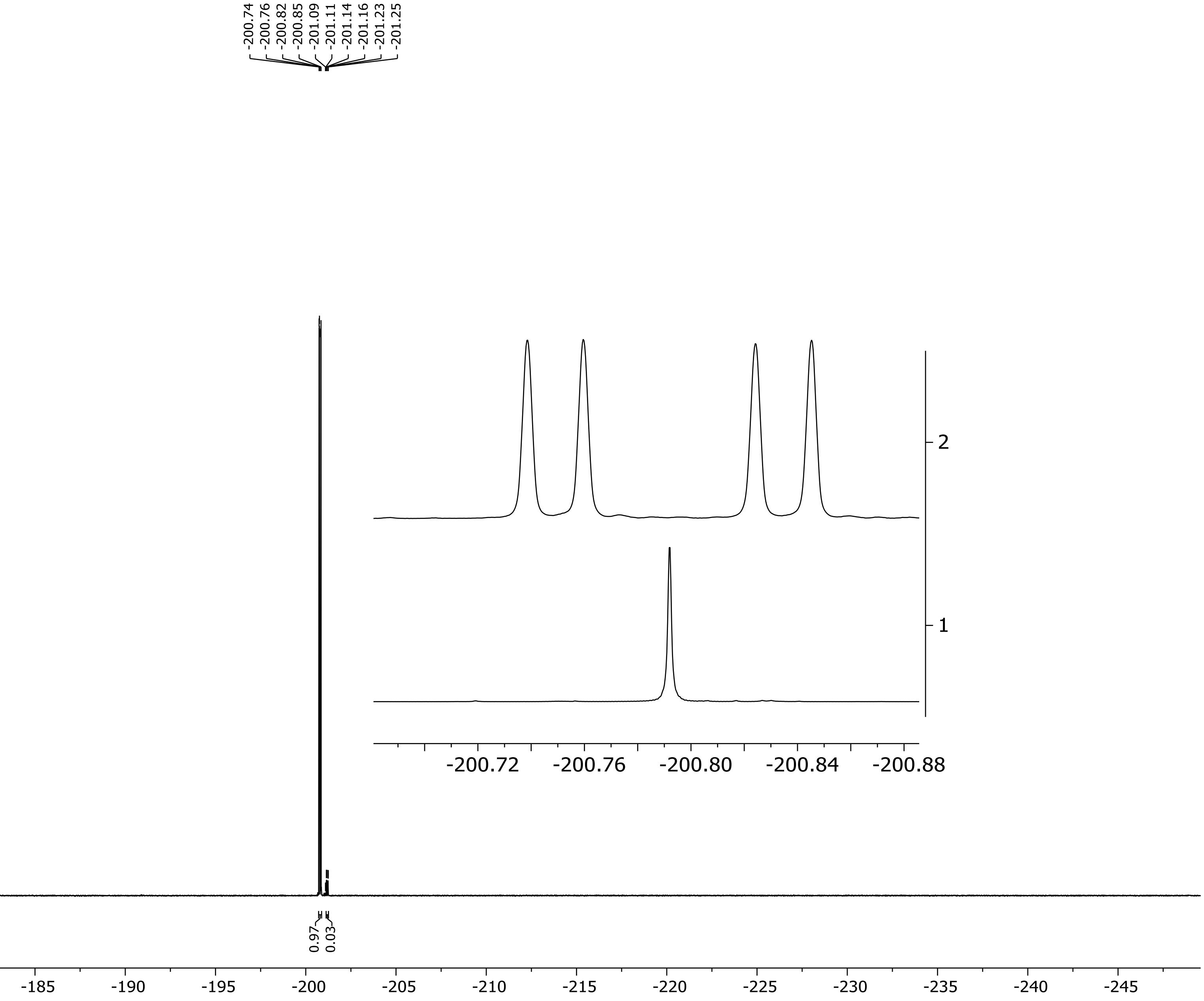

Compound **S2** (^{1}H NMR, 599 MHz, CD_2Cl_2)

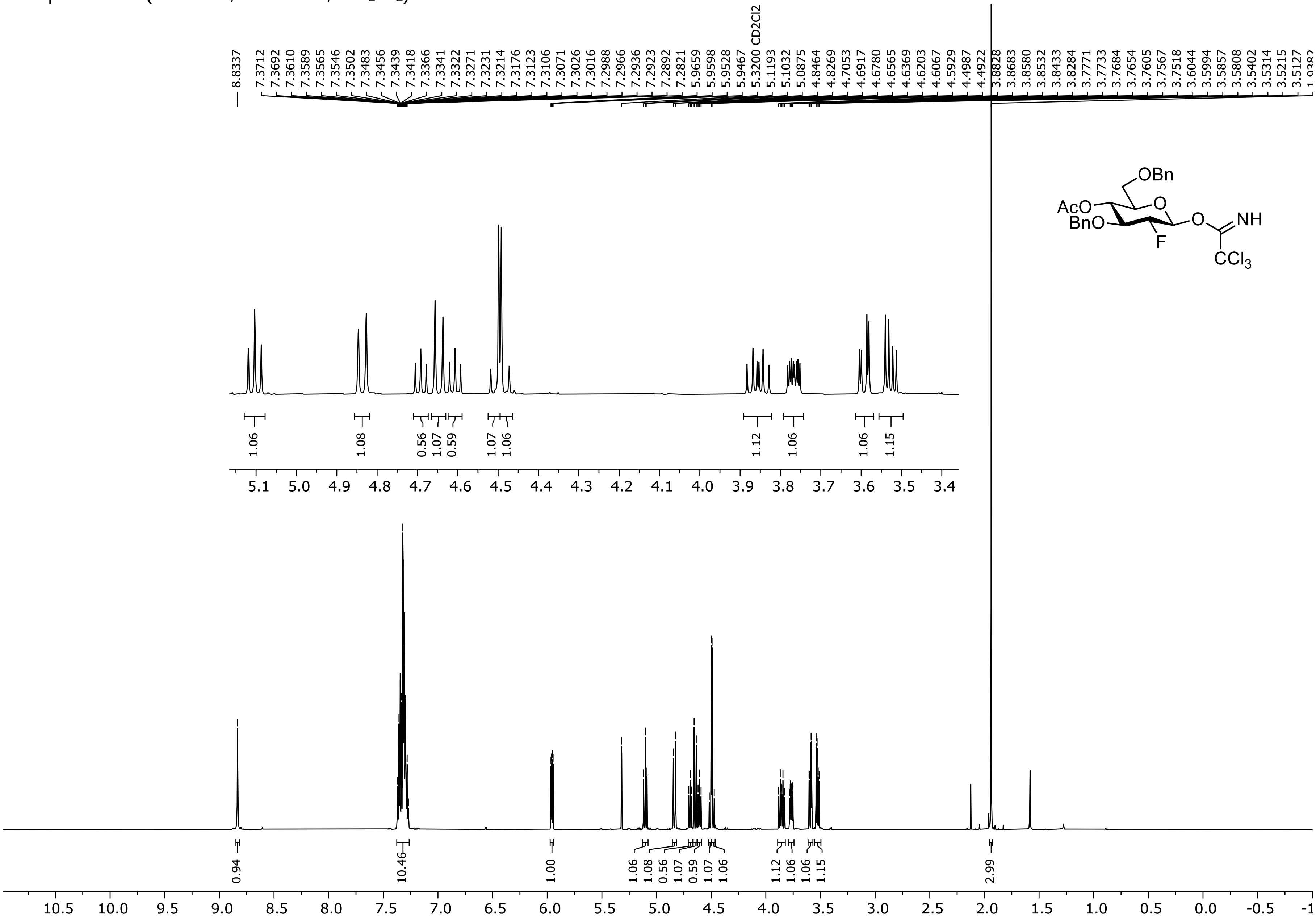

Compound **S2** (^{13}C NMR, 151 MHz, CD_2Cl_2)

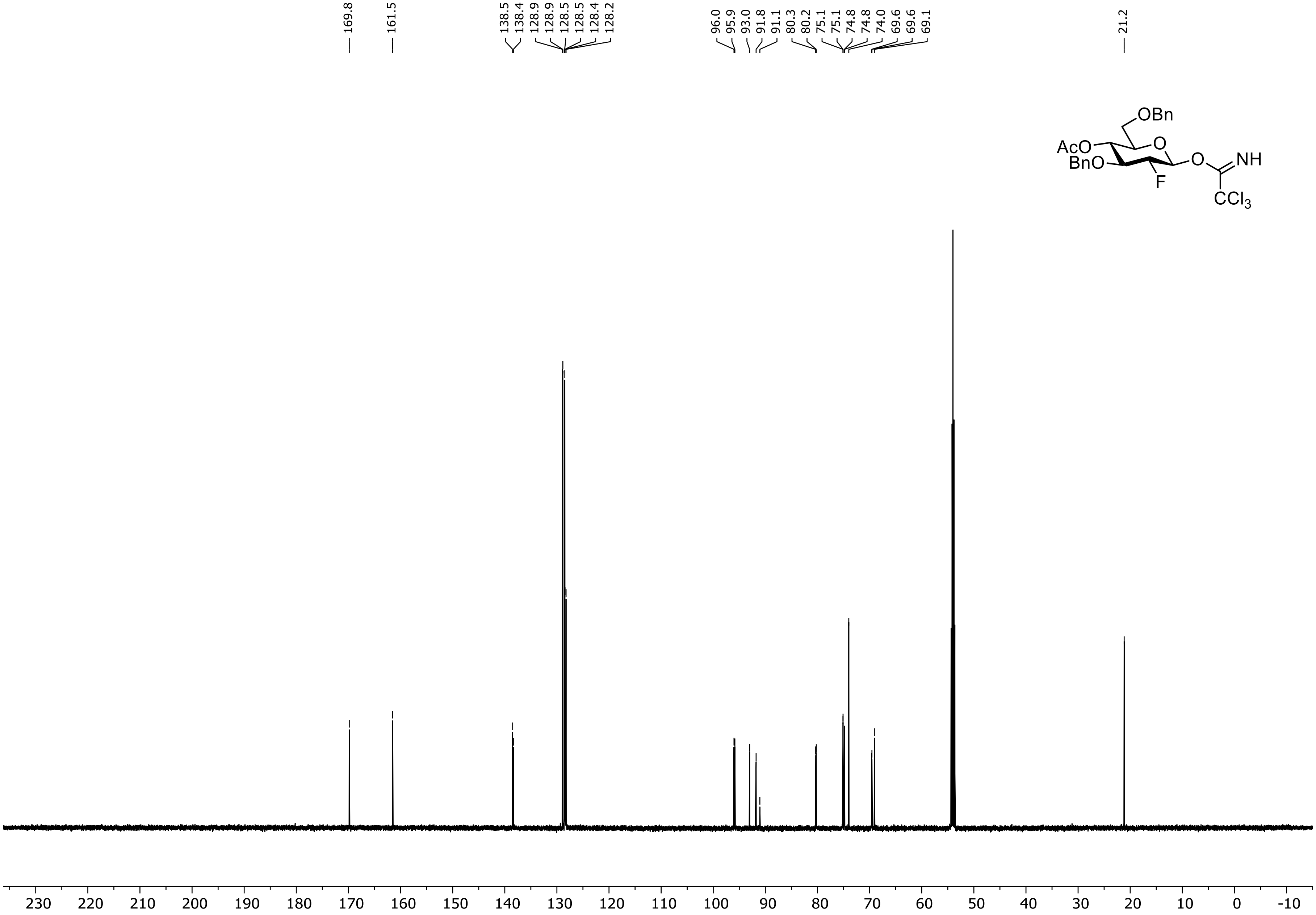

Compound **S2** (^{19}F NMR, 564 MHz, CD_2Cl_2) and (1) $^{19}F\{^1H\}$ and (2) ^{19}F NMR (564 MHz)

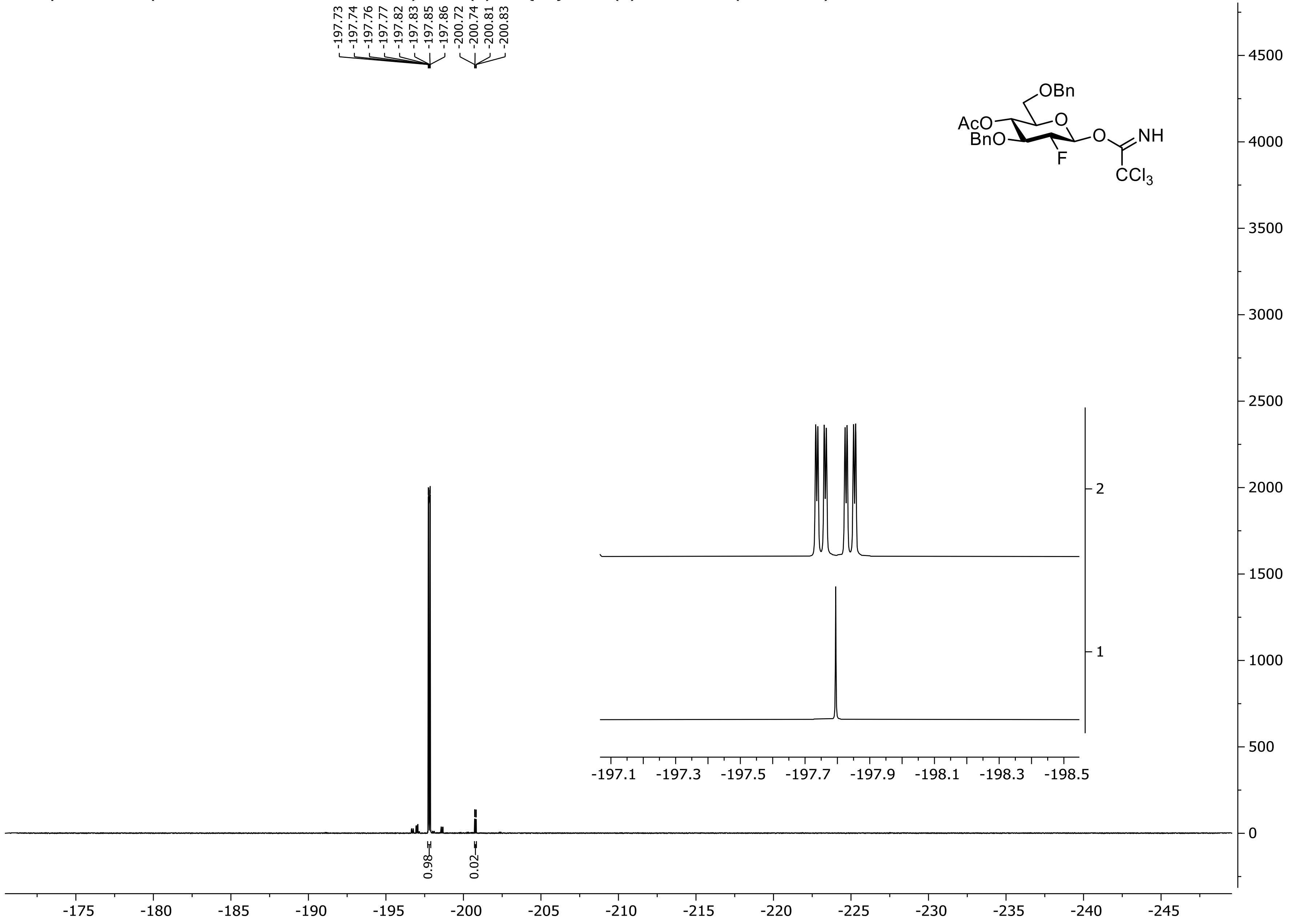

Compound **9** (^{1}H NMR, 599 MHz, $CDCl_3$)

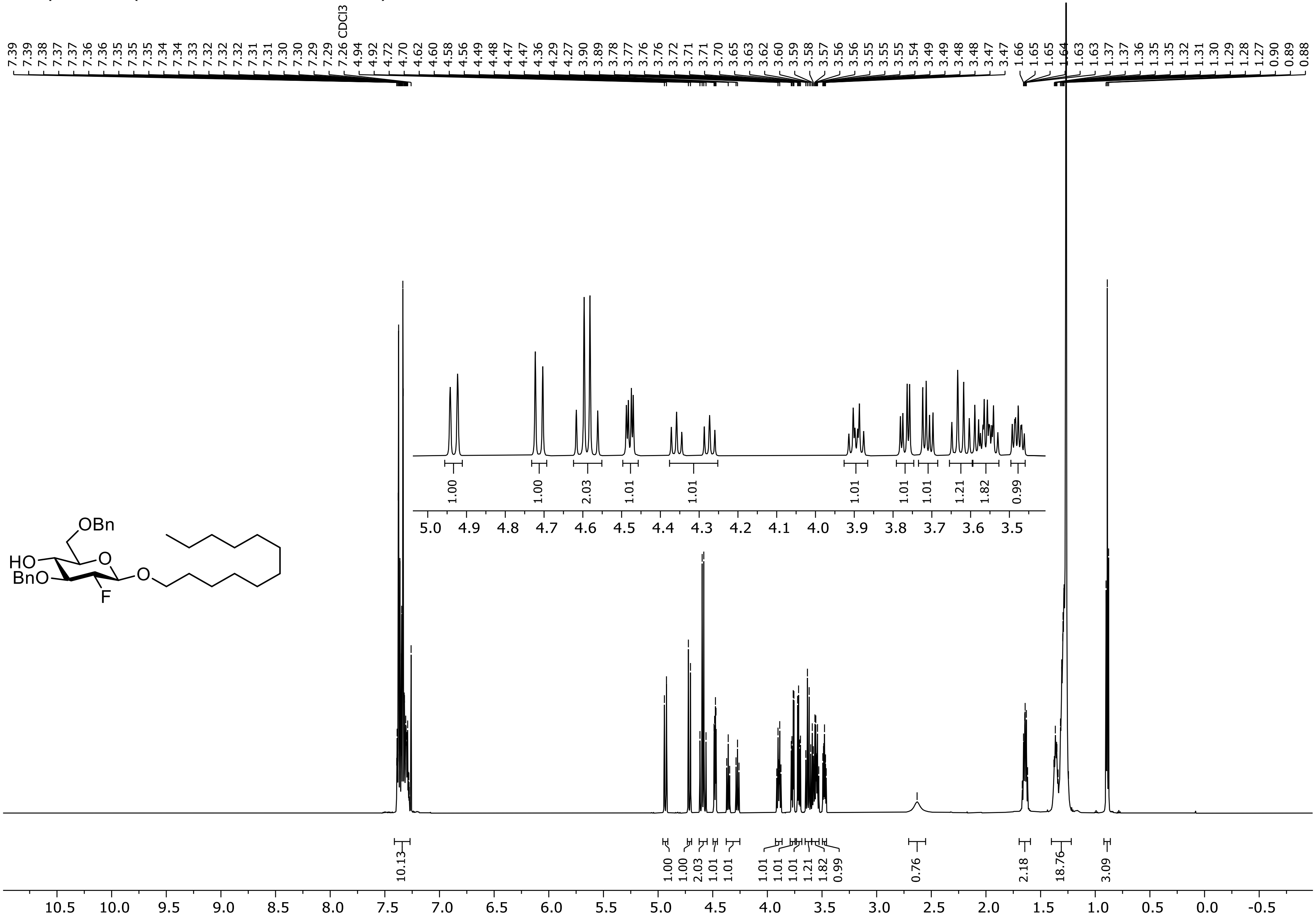

Compound **9** (^{13}C NMR, 151 MHz, $CDCl_3$)

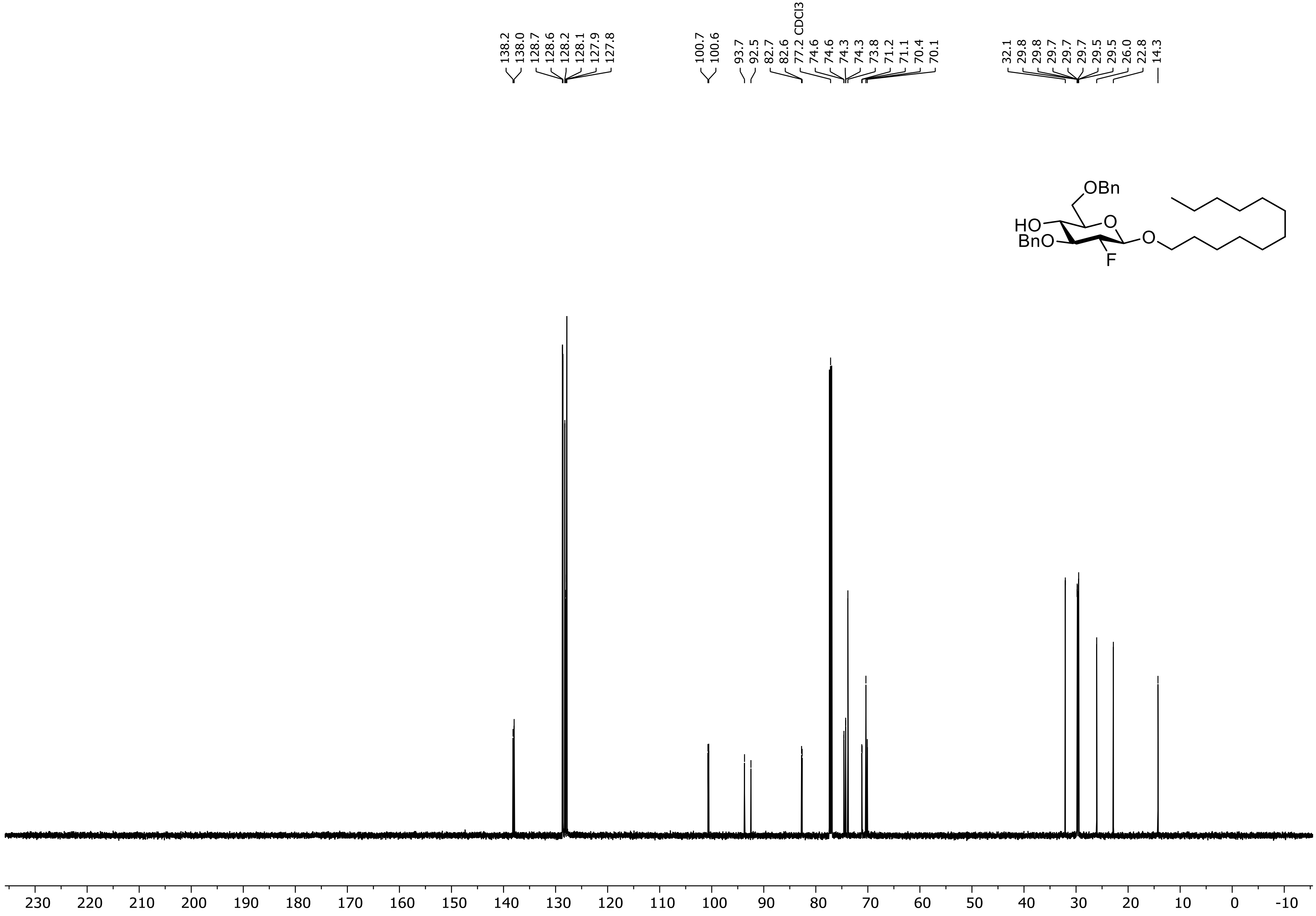

Compound **9** (^{19}F NMR, 564 MHz, $CDCl_3$) and (1) $^{19}F\{^1H\}$ and (2) ^{19}F NMR (564 MHz)

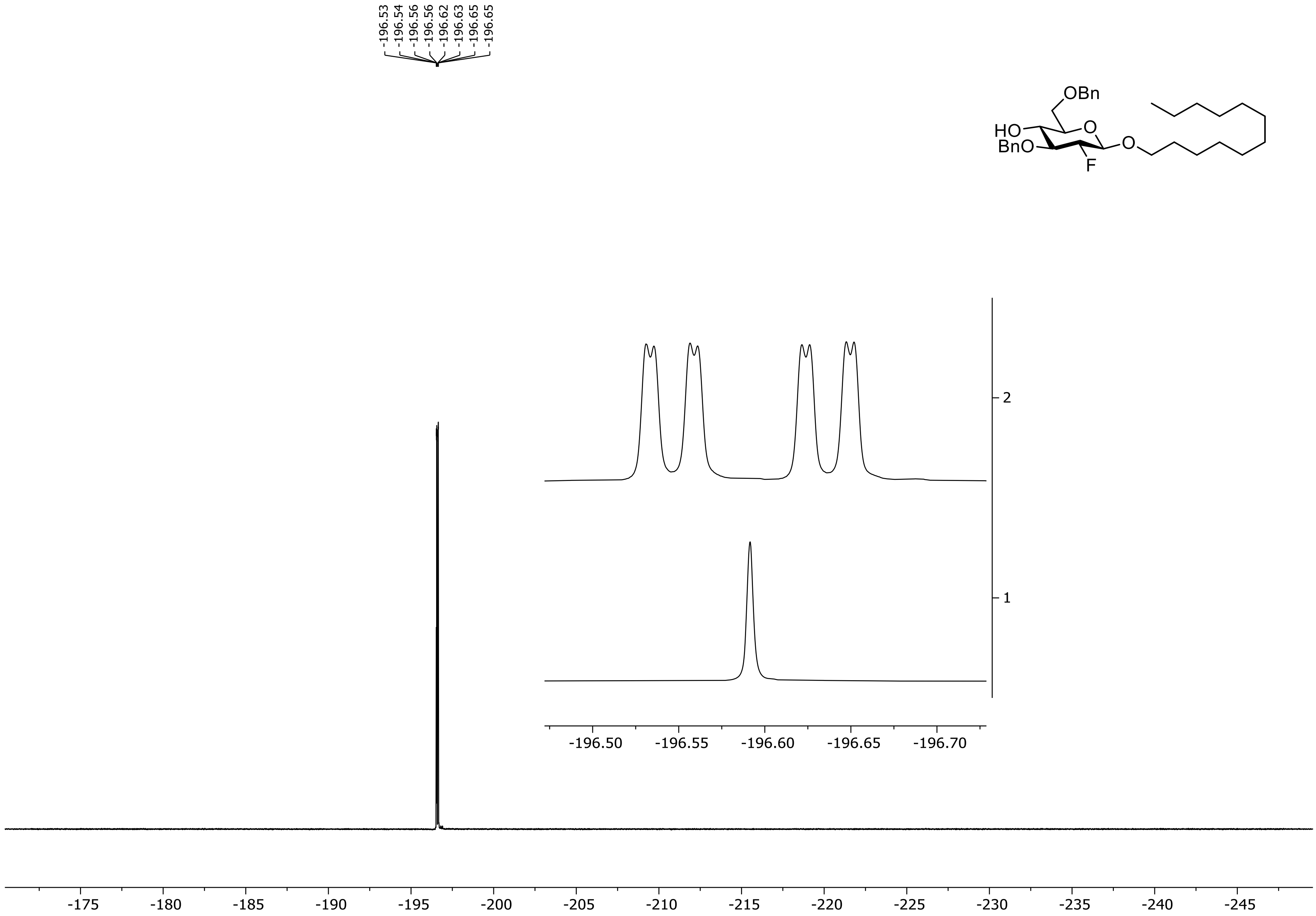

Compound **15** (^{1}H NMR, 599 MHz, $CDCl_3$)

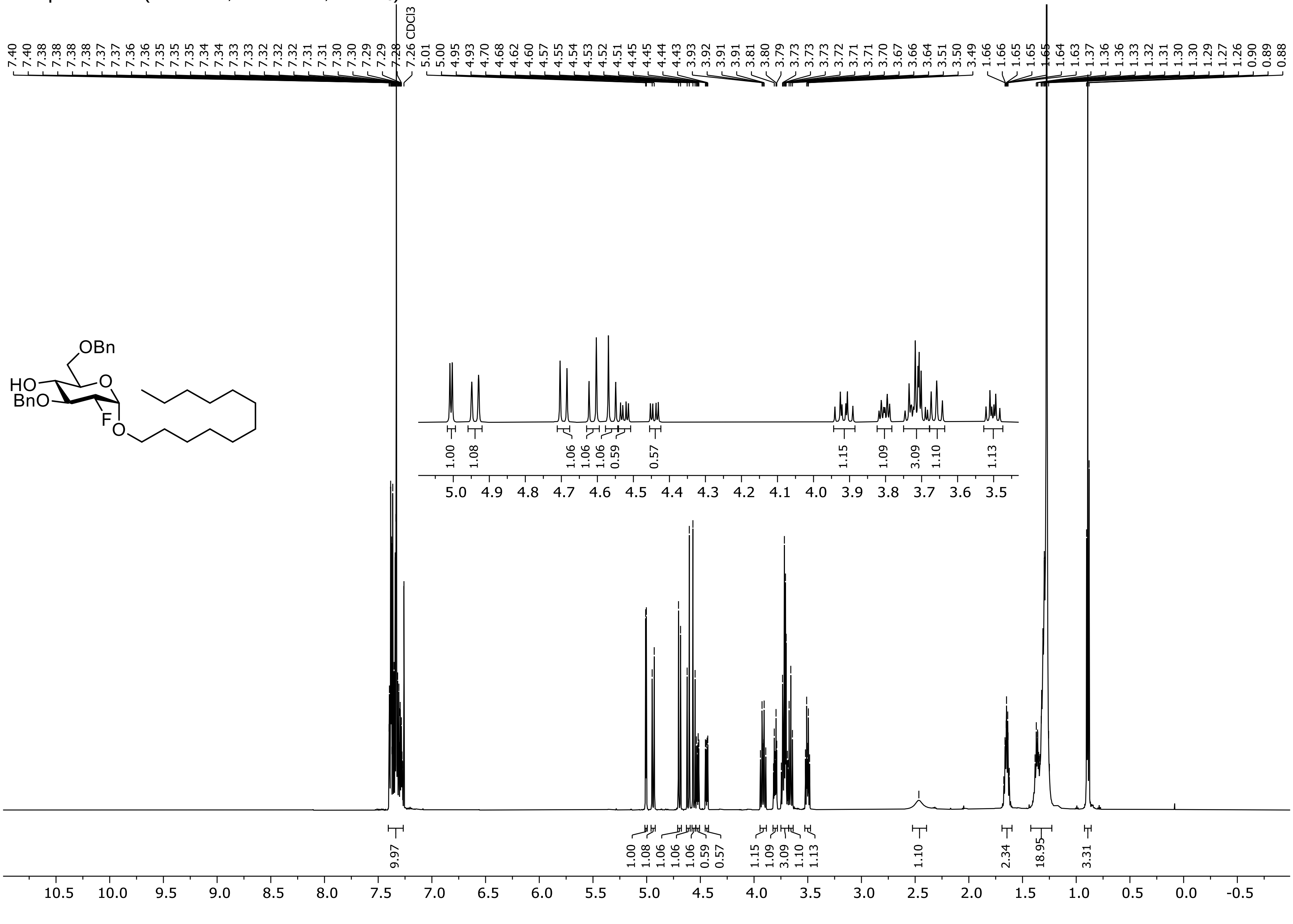

Compound **15** (^{13}C NMR, 151 MHz, $CDCl_3$)

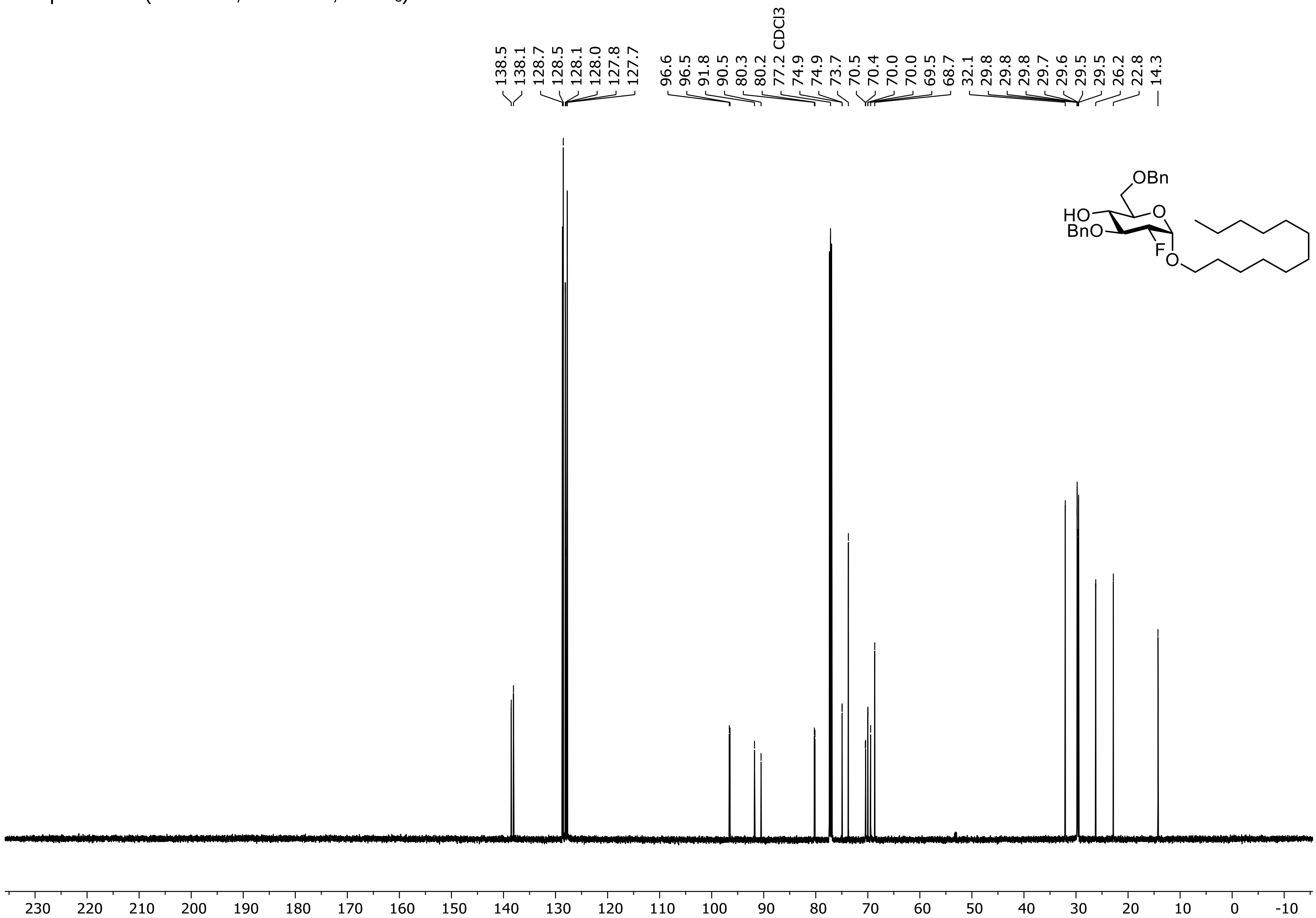

Compound **15** (^{19}F NMR, 564 MHz, $CDCl_3$) and (1) $^{19}F\{^1H\}$ and (2) ^{19}F NMR (564 MHz)

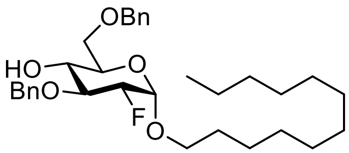

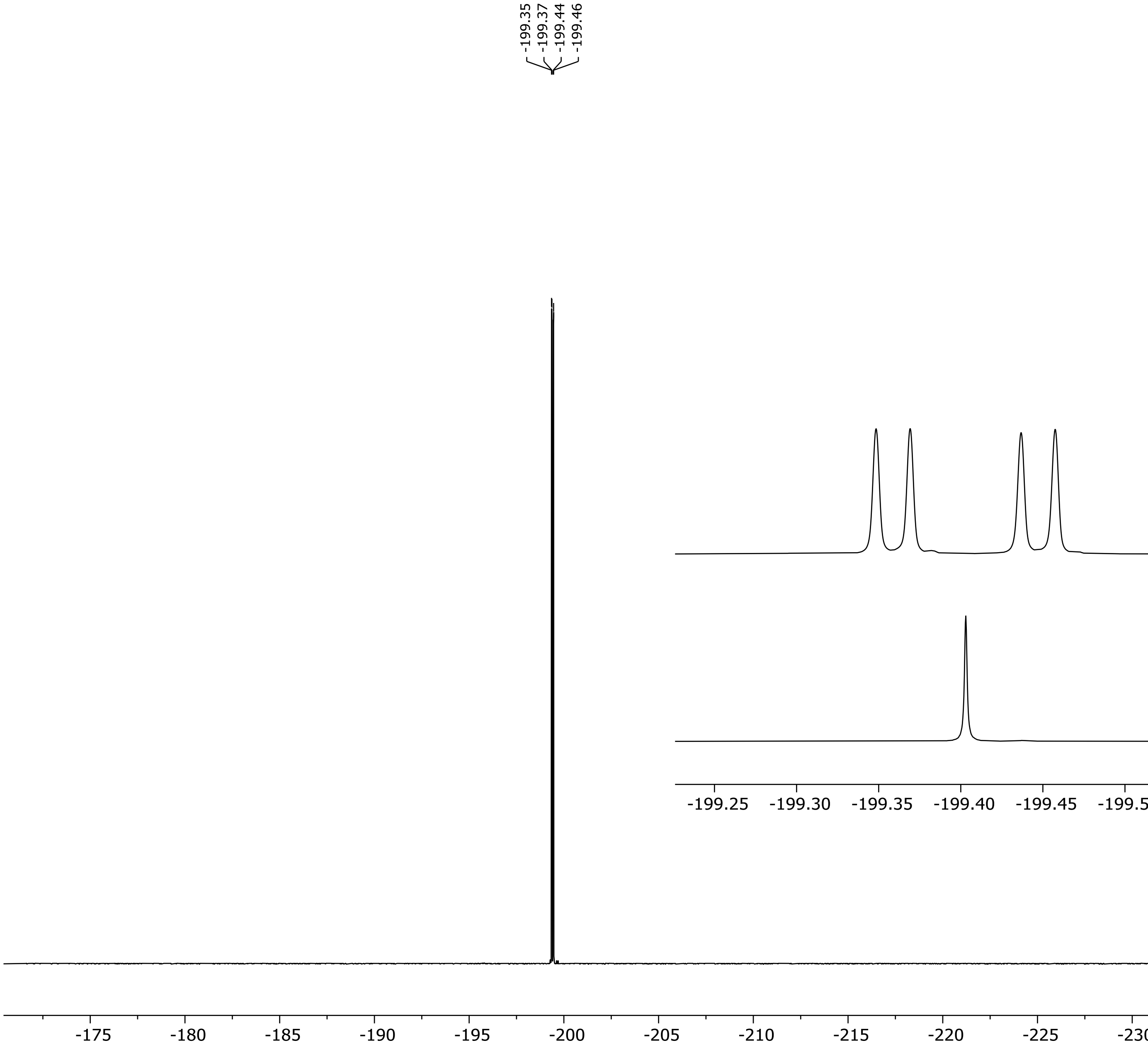

Compound **16** (^{1}H NMR, 599 MHz, $CDCl_3$)

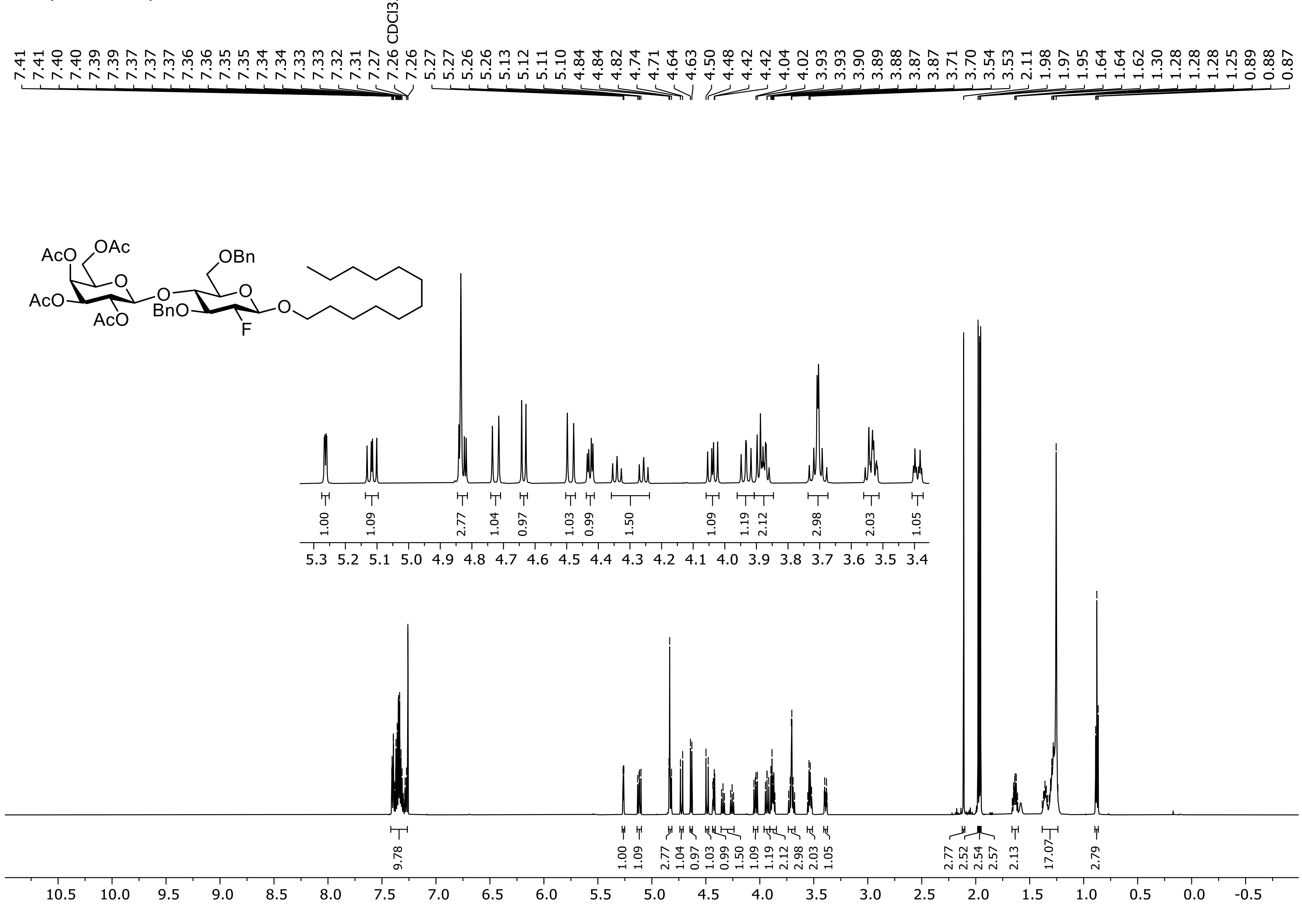

Compound **16** (^{13}C NMR, 151 MHz, $CDCl_3$)

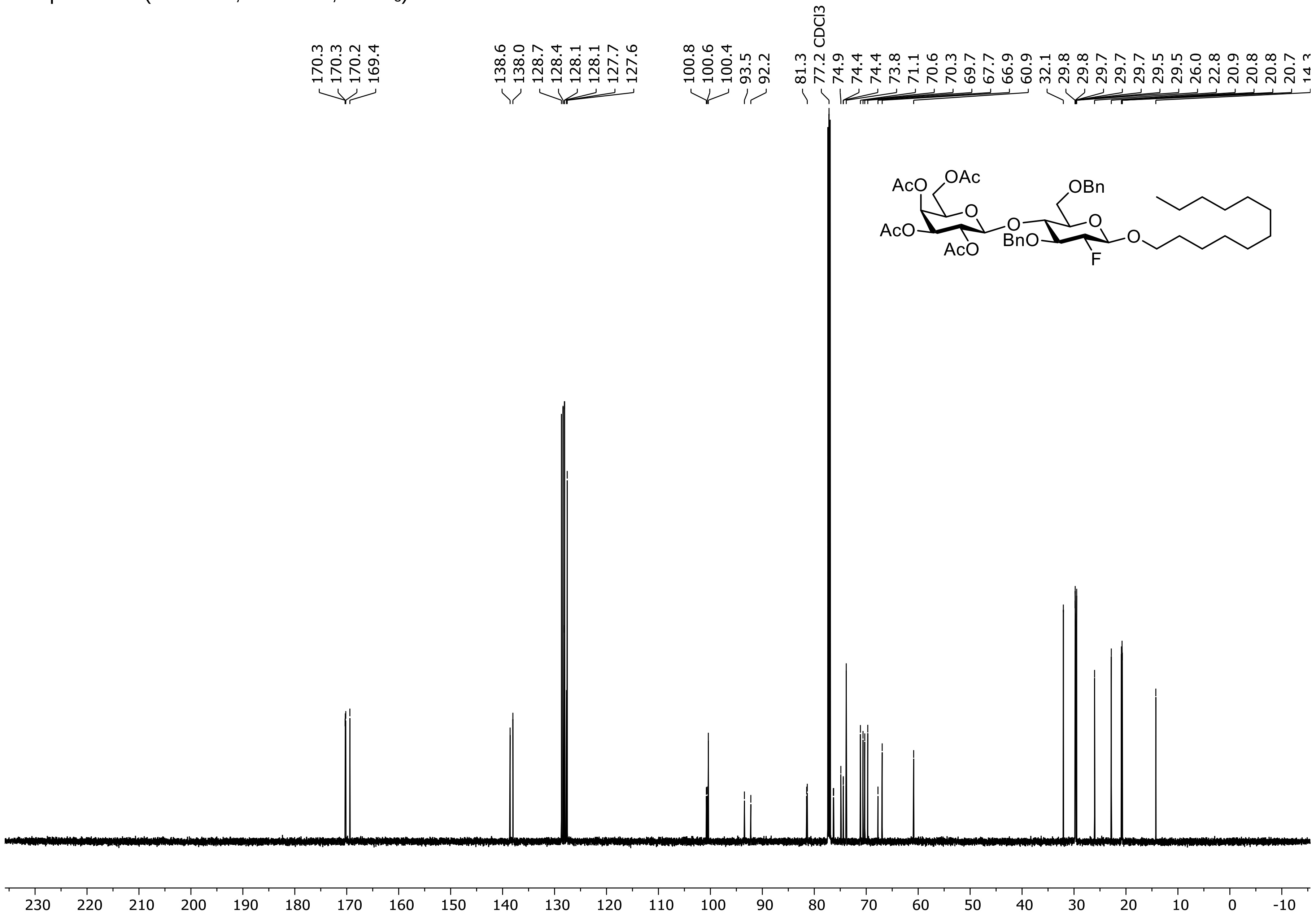

Compound **16** (^{19}F NMR, 564 MHz, $CDCl_3$) and (1) ^{19}F{^{1}H} and (2) ^{19}F NMR (564 MHz)

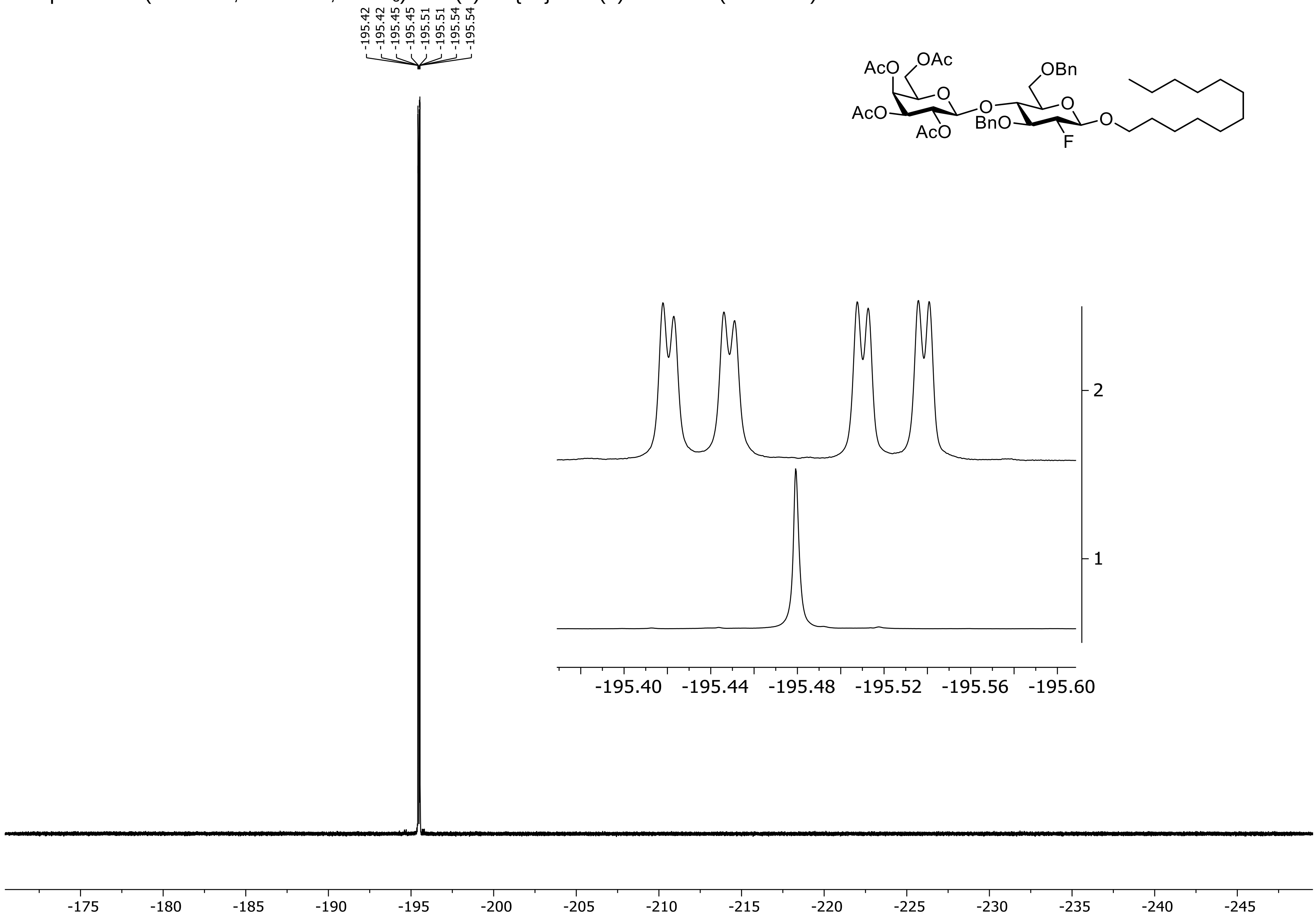

Compound **16** (HMBC, $CDCl_3$)

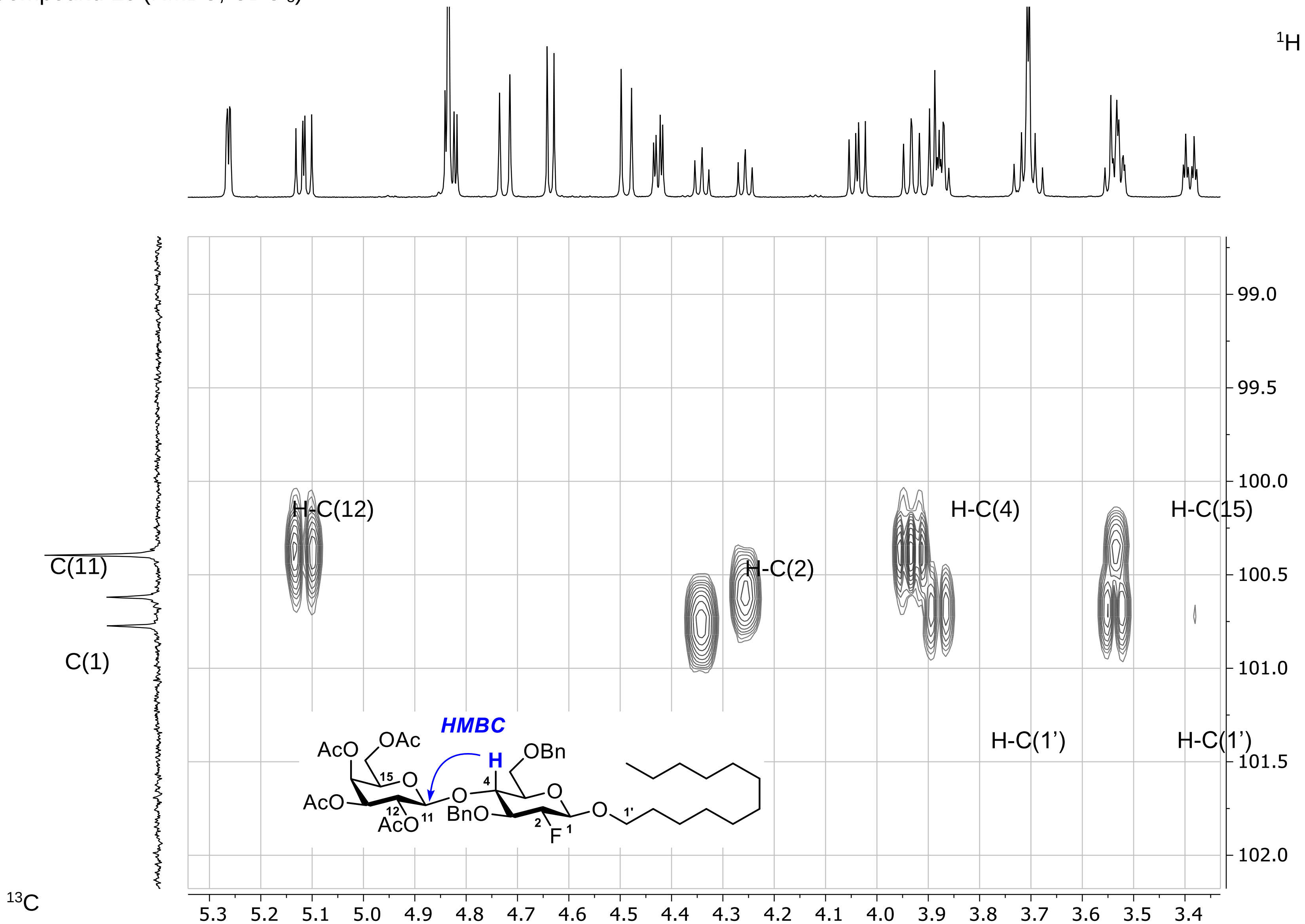

Compound **17** (^{1}H NMR, 599 MHz, CD_2Cl_2)

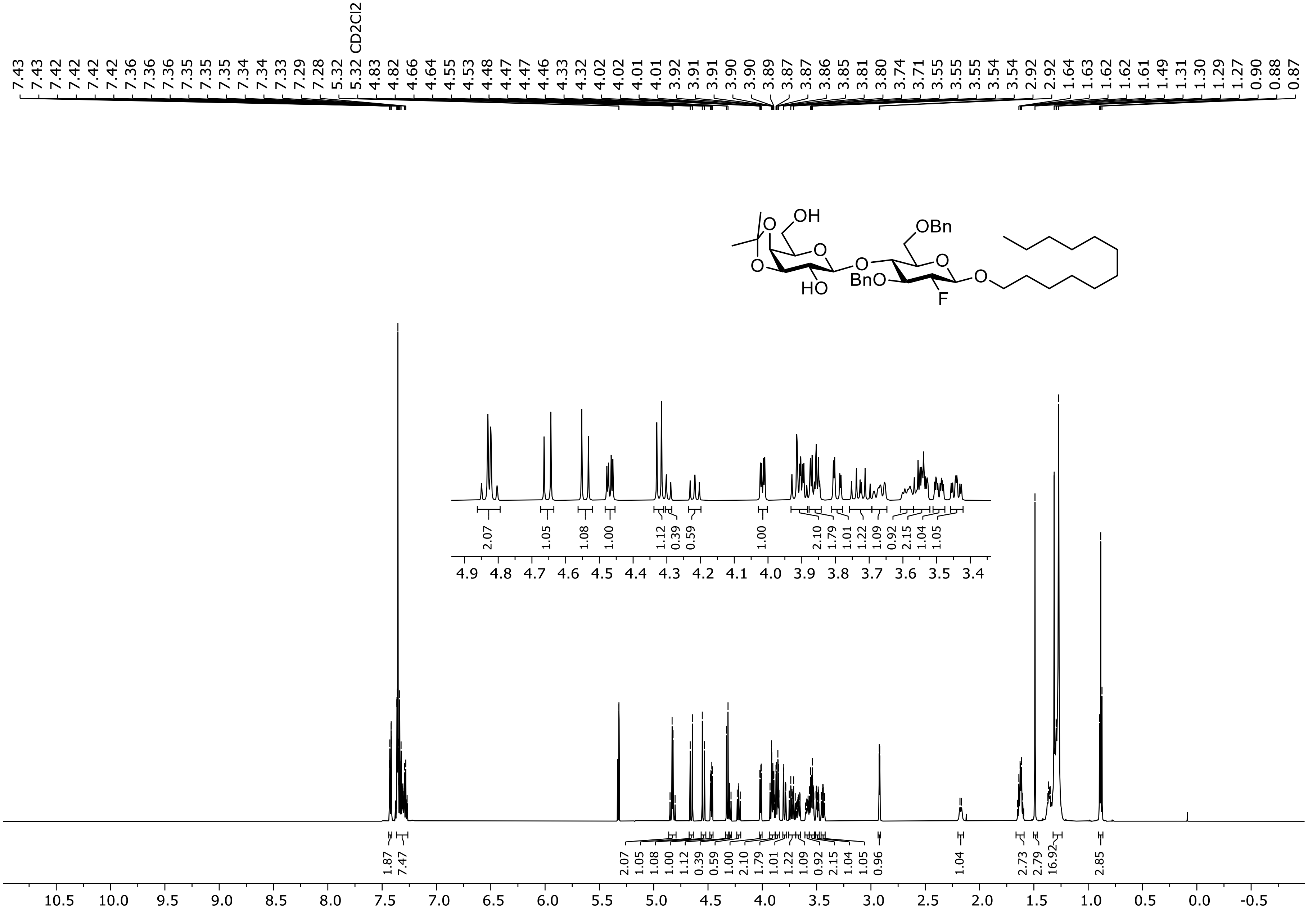

Compound **17** (^{13}C NMR, 151 MHz, CD_2Cl_2)

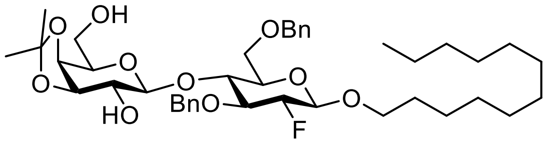

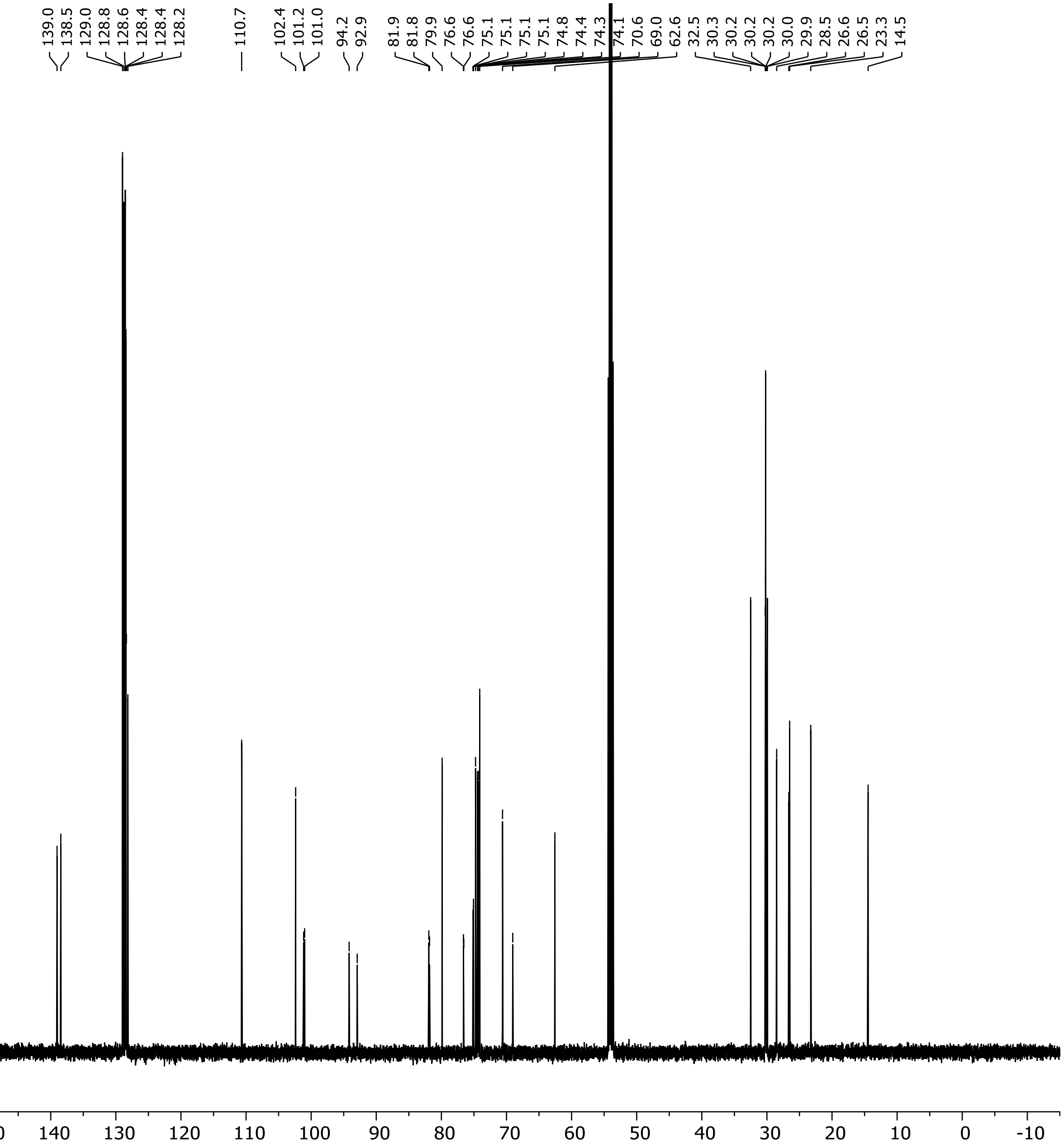

Compound **17** (^{19}F NMR, 564 MHz, CD_2Cl_2) and (1) $^{19}F\{^1H\}$ and (2) ^{19}F NMR (564 MHz)

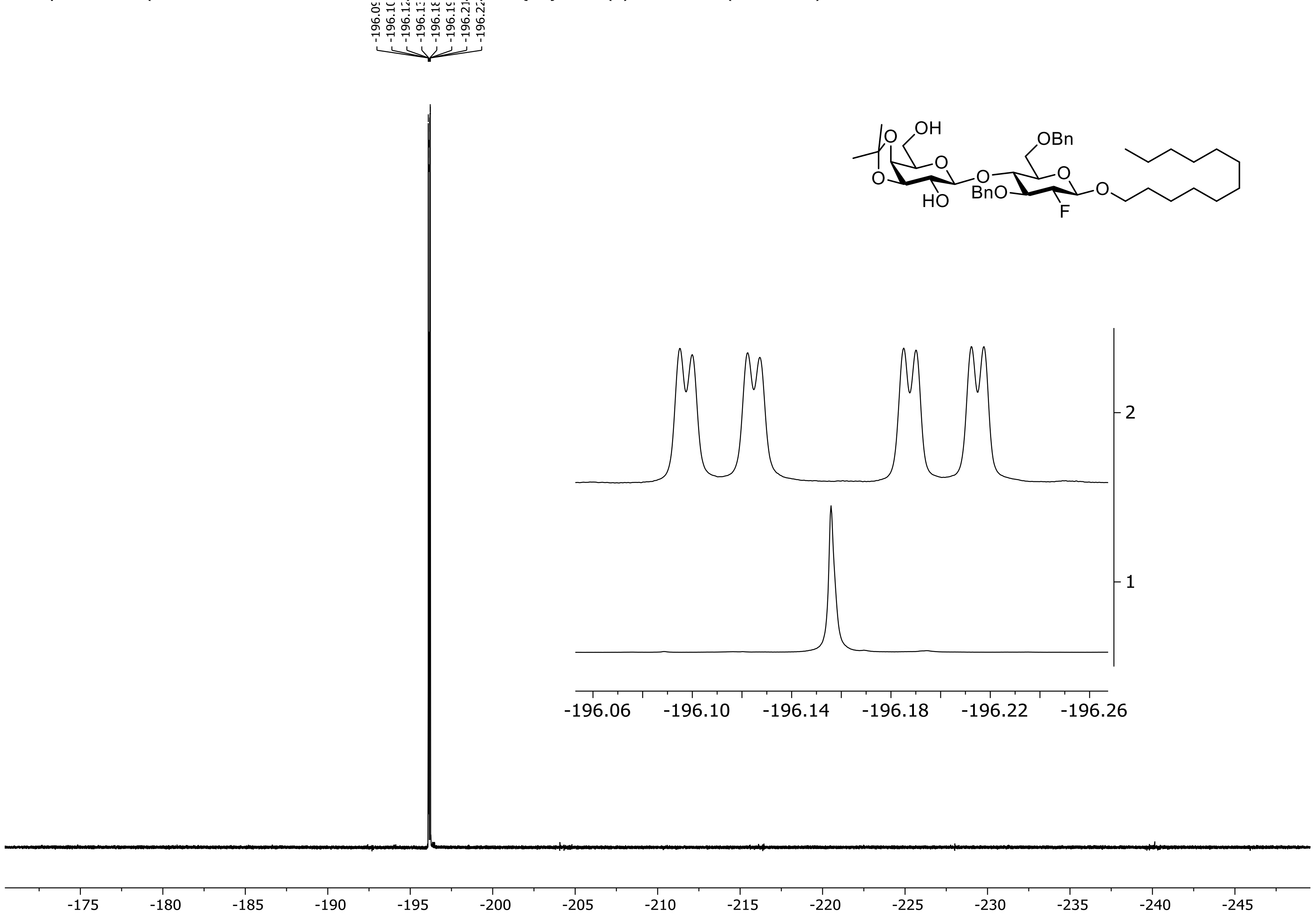

Compound **7** (^{1}H NMR, 599 MHz, $CDCl_3$)

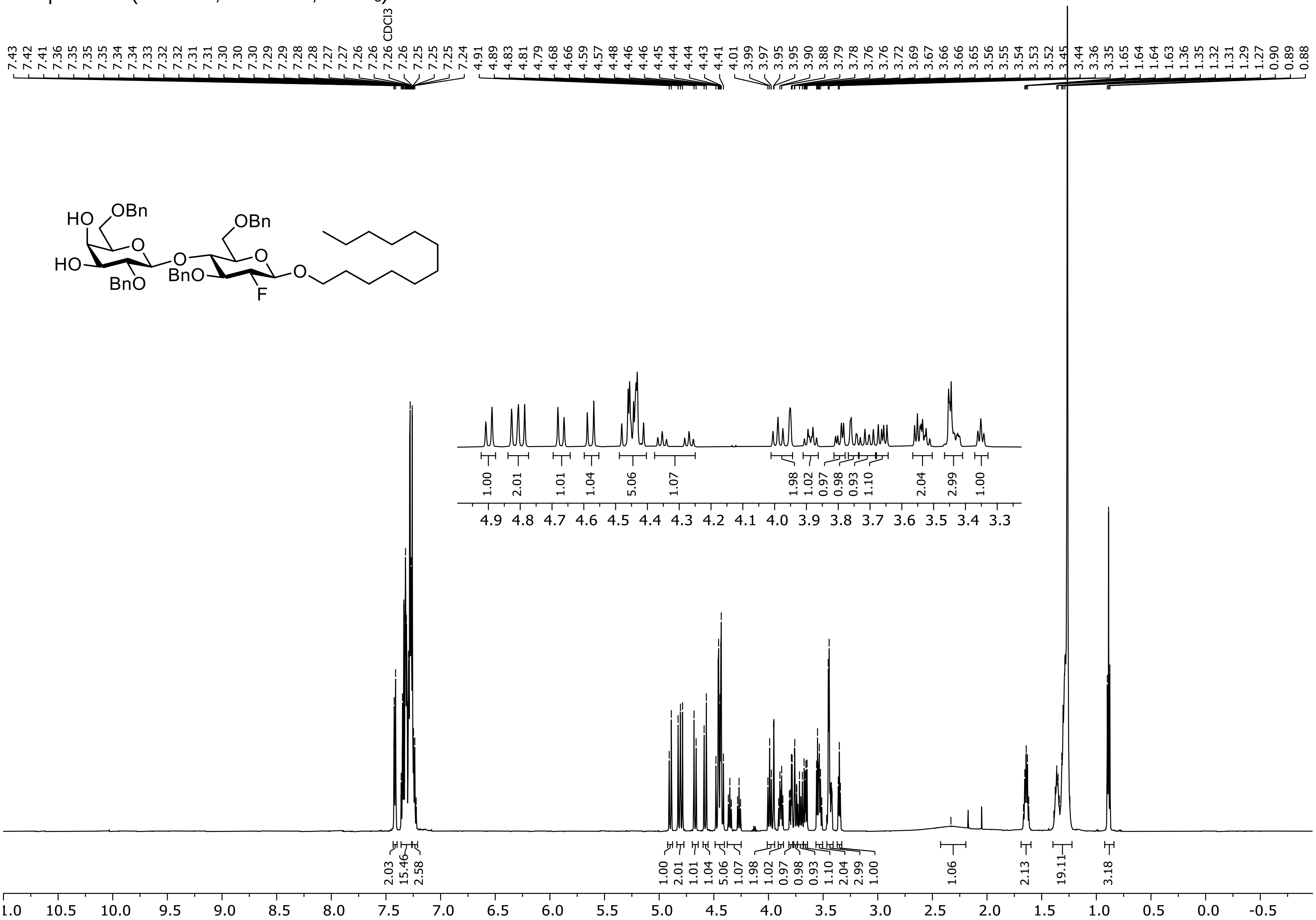

Compound **7** (^{13}C NMR, 151 MHz, $CDCl_3$)

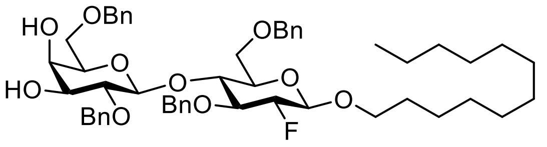

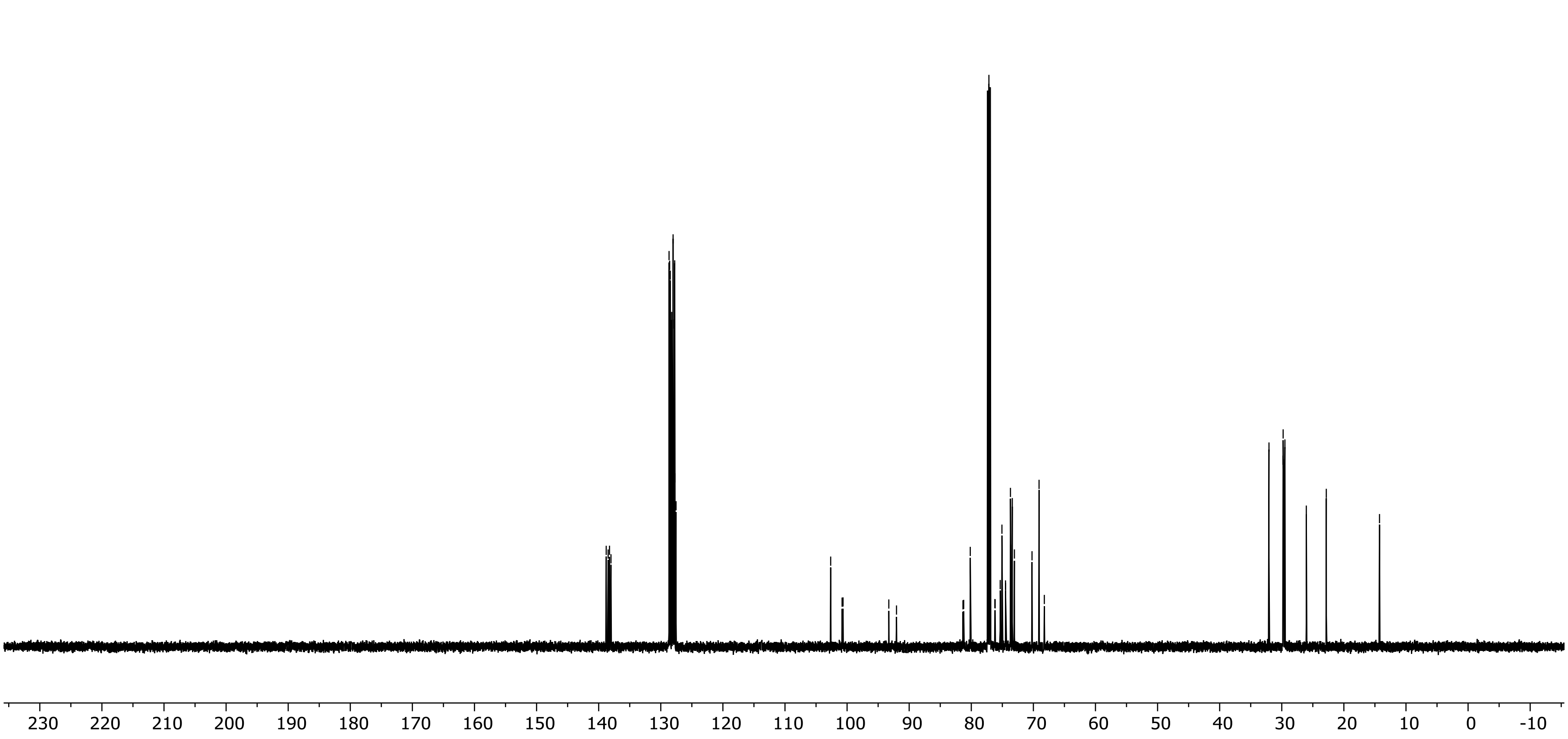

Compound **7** (^{19}F NMR, 564 MHz, $CDCl_3$) and (1) ^{19}F{^{1}H} and (2) ^{19}F NMR (564 MHz)

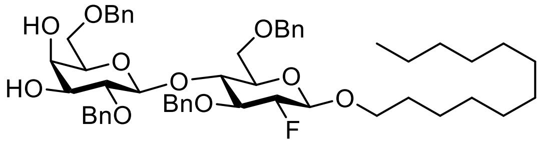

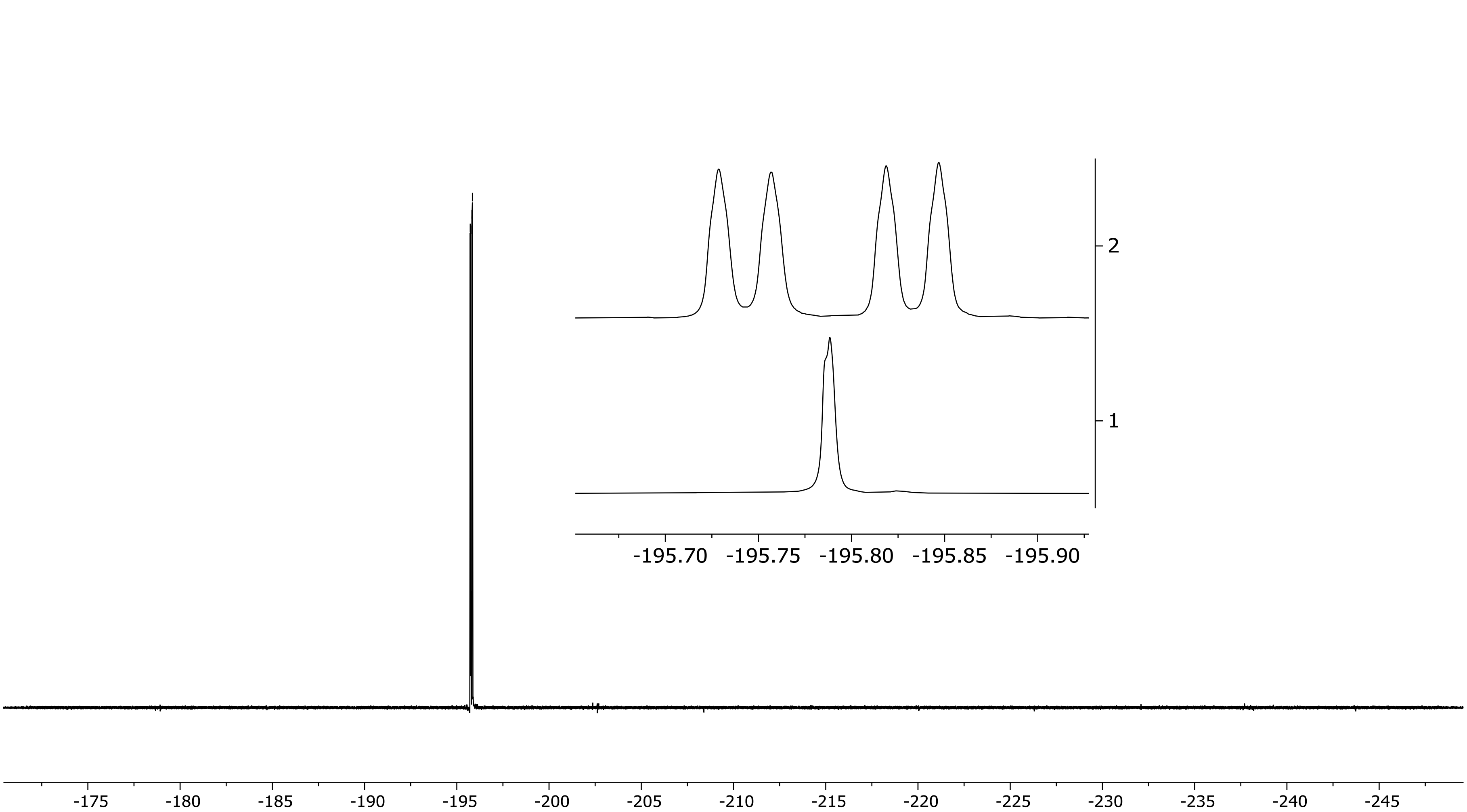

Compound **6** (^1H NMR, 599 MHz, CD_2Cl_2)

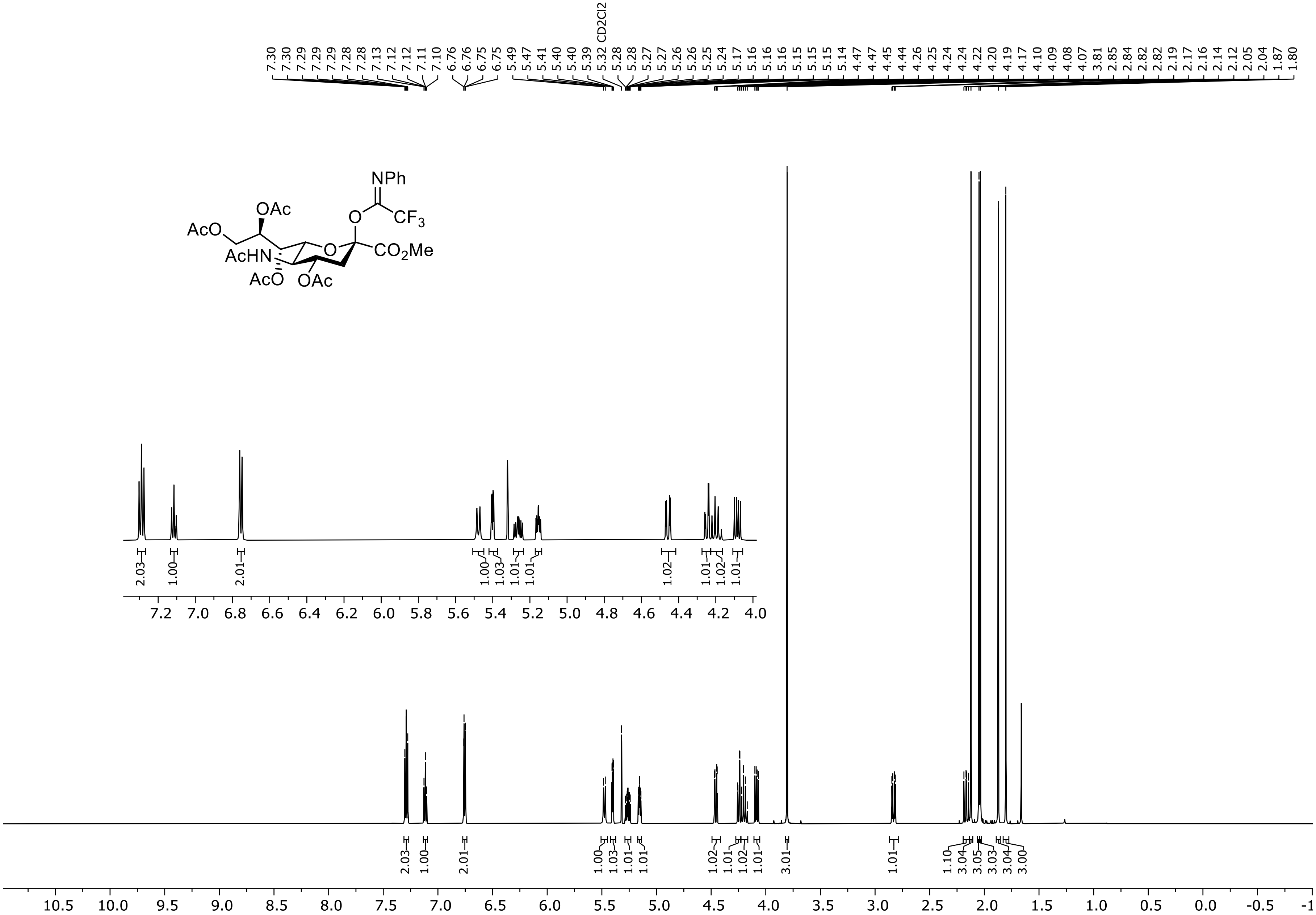

Compound **6** (^{13}C NMR, 151 MHz, CD_2Cl_2)

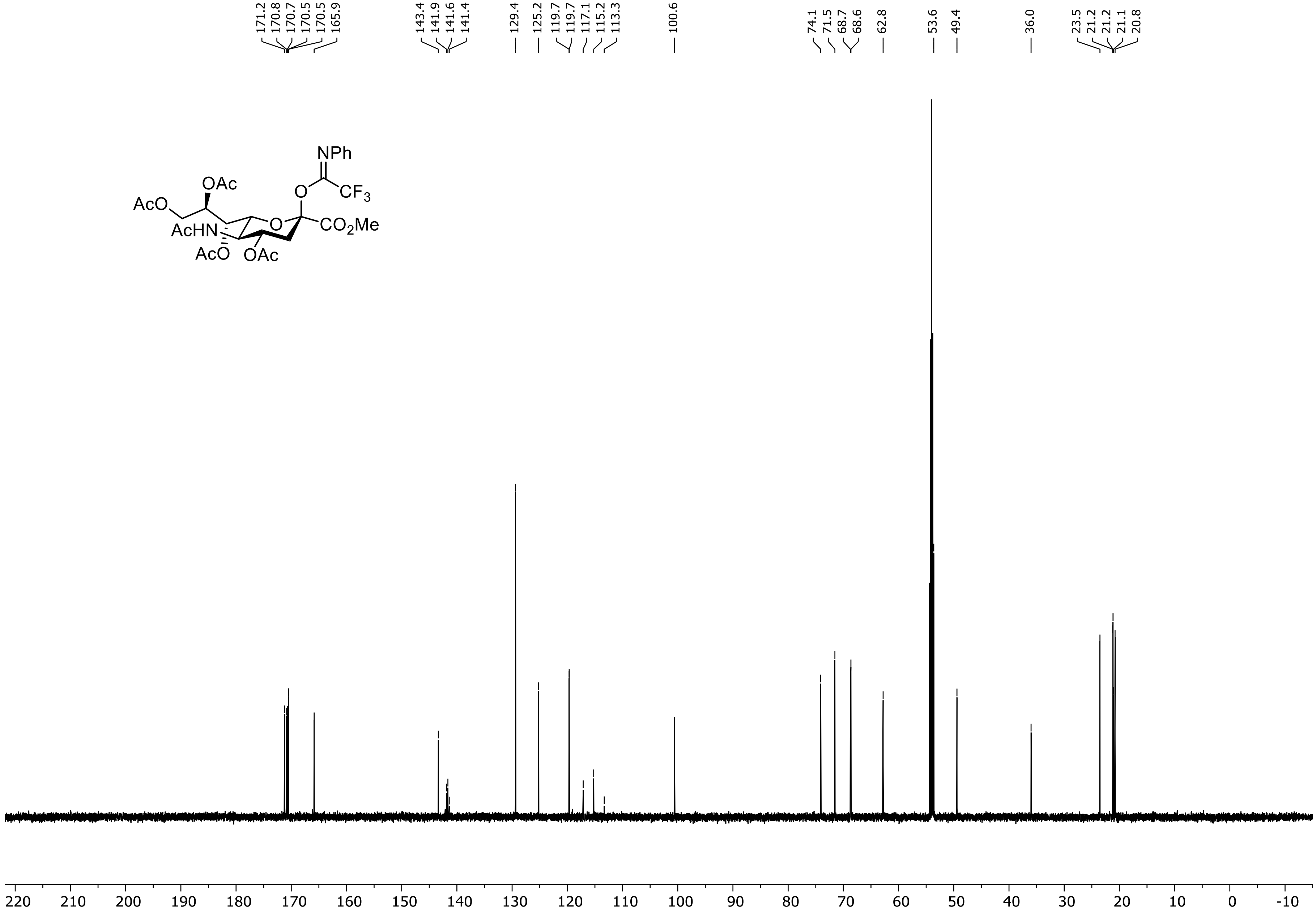

Compound **6** (^{19}F NMR, 564 MHz, CD_2Cl_2)

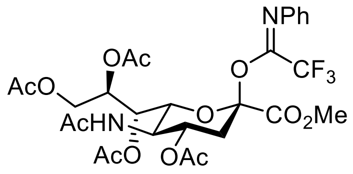

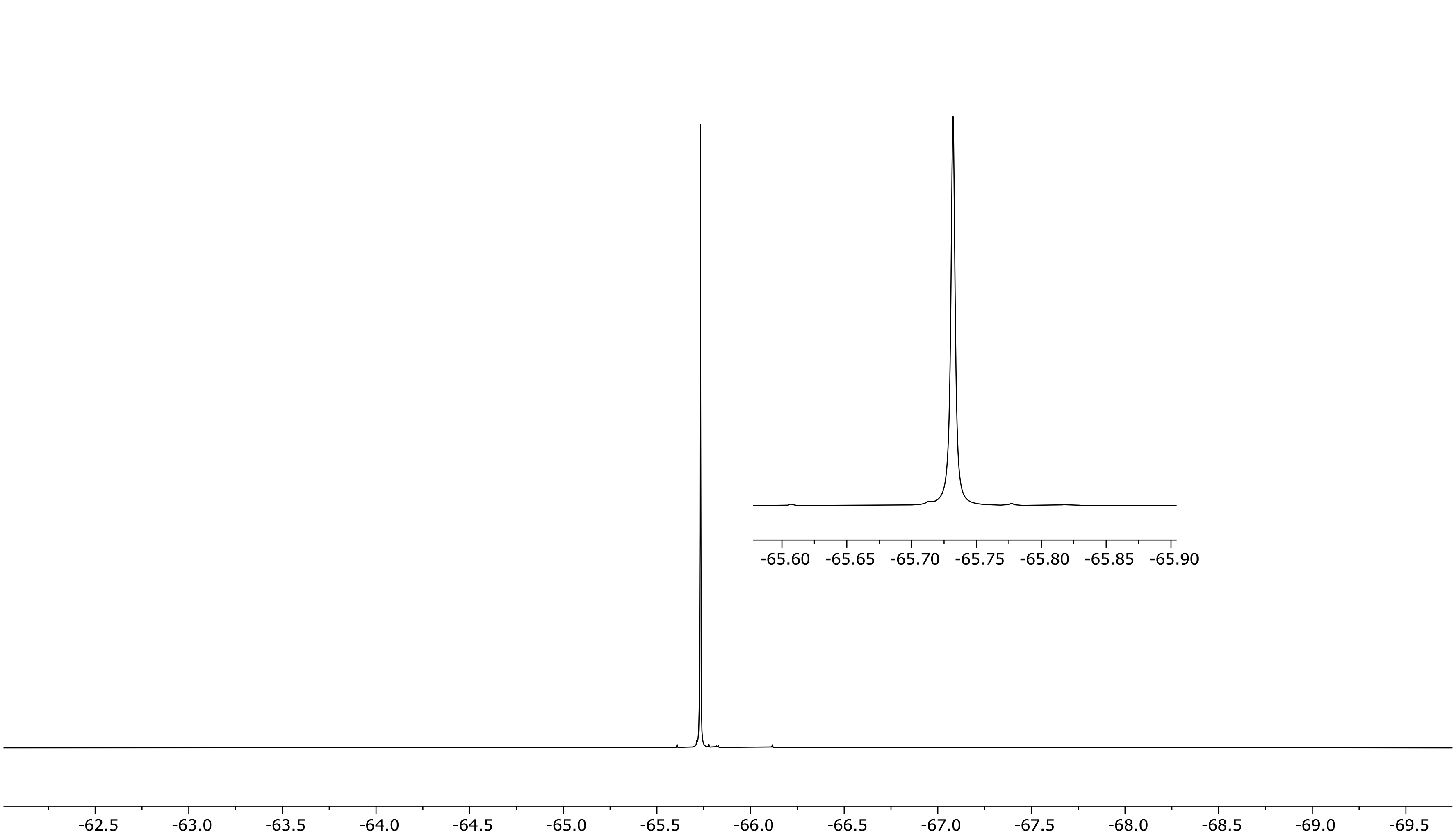

Compound **3** (^{1}H NMR, 599 MHz, $CDCl_3$)

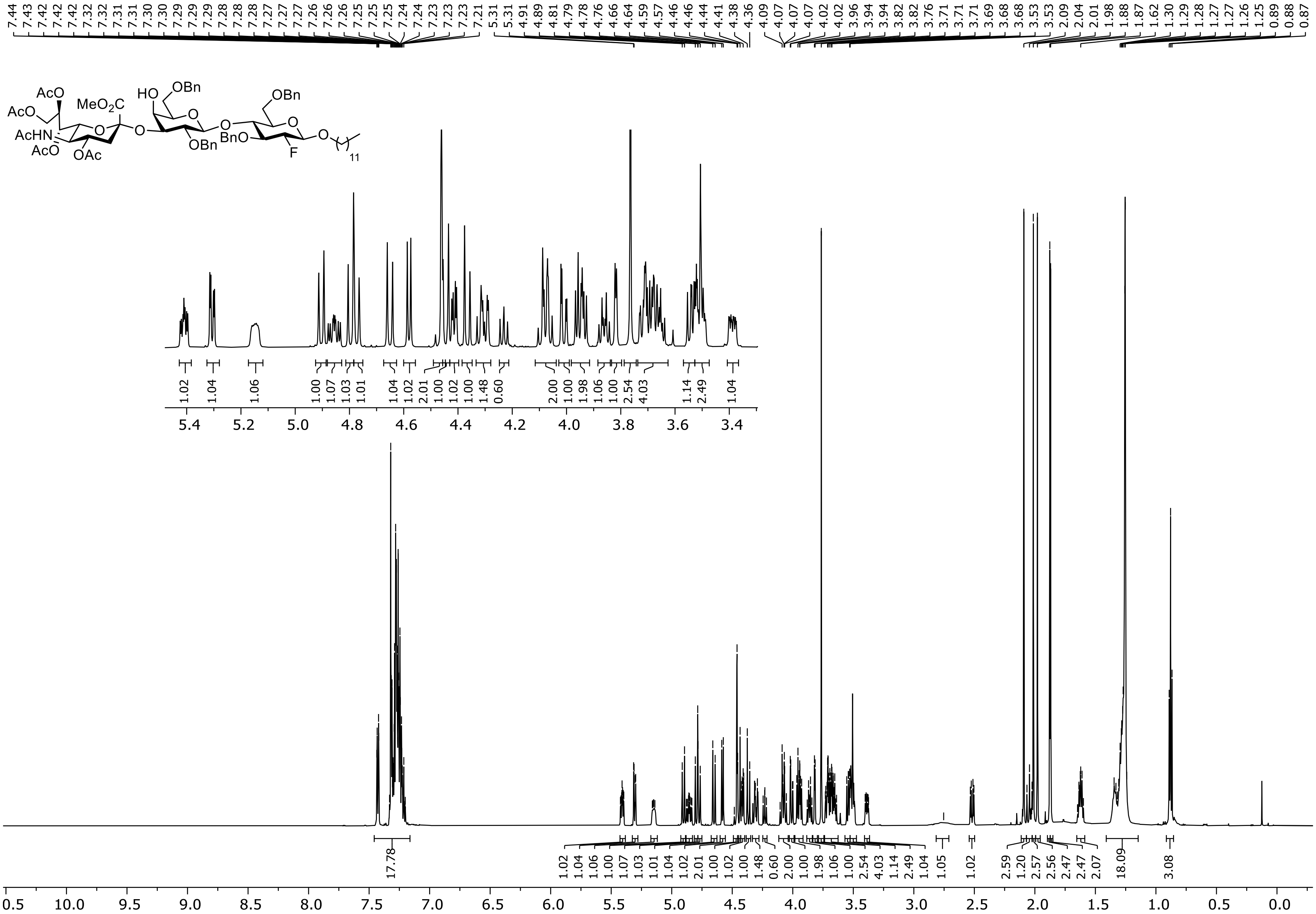

Compound **3** (^{13}C NMR, 151 MHz, $CDCl_3$)

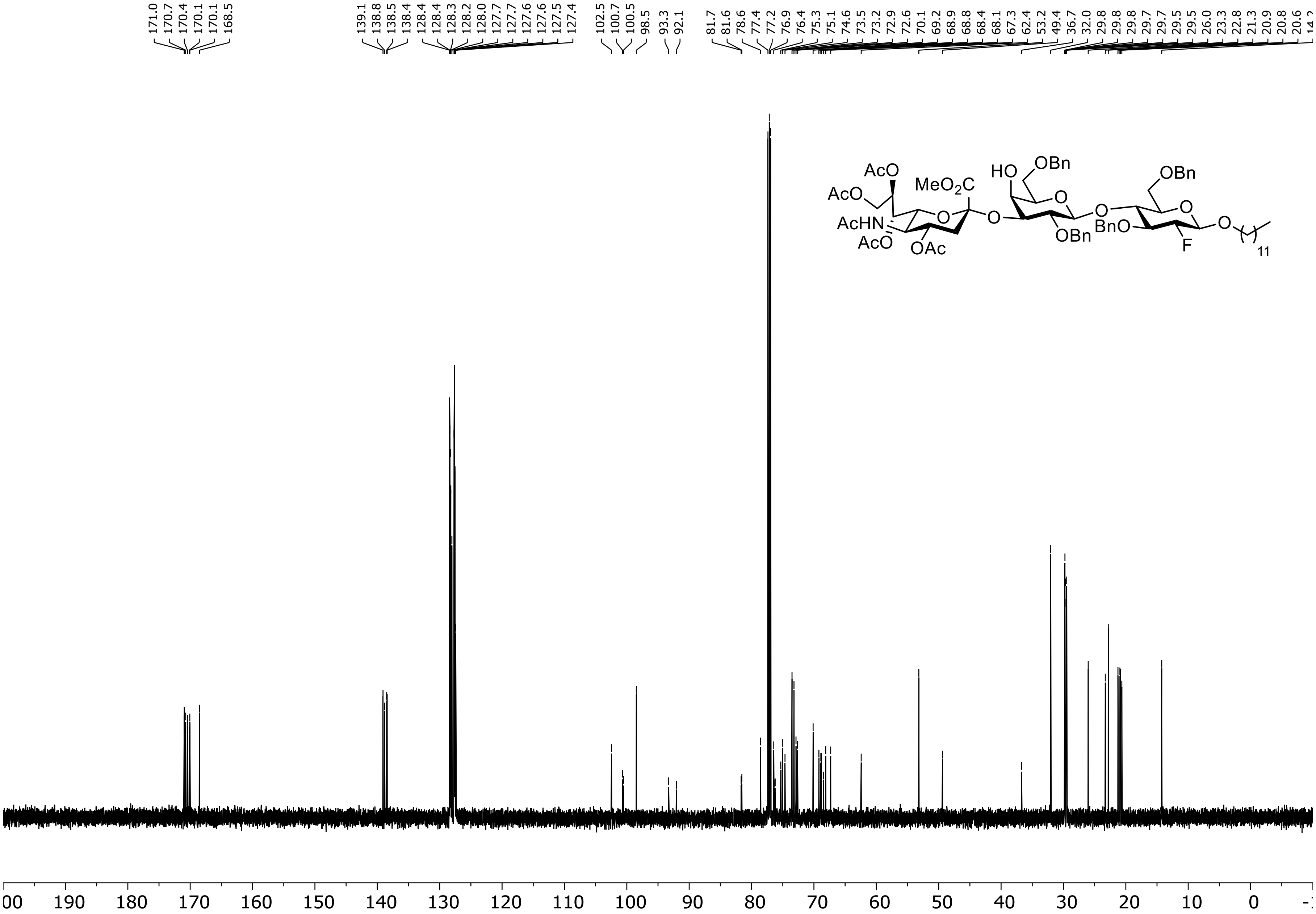

Compound **3** (^{19}F NMR, 564 MHz, $CDCl_3$) and (1) $^{19}F\{^1H\}$ and (2) ^{19}F NMR (564 MHz)

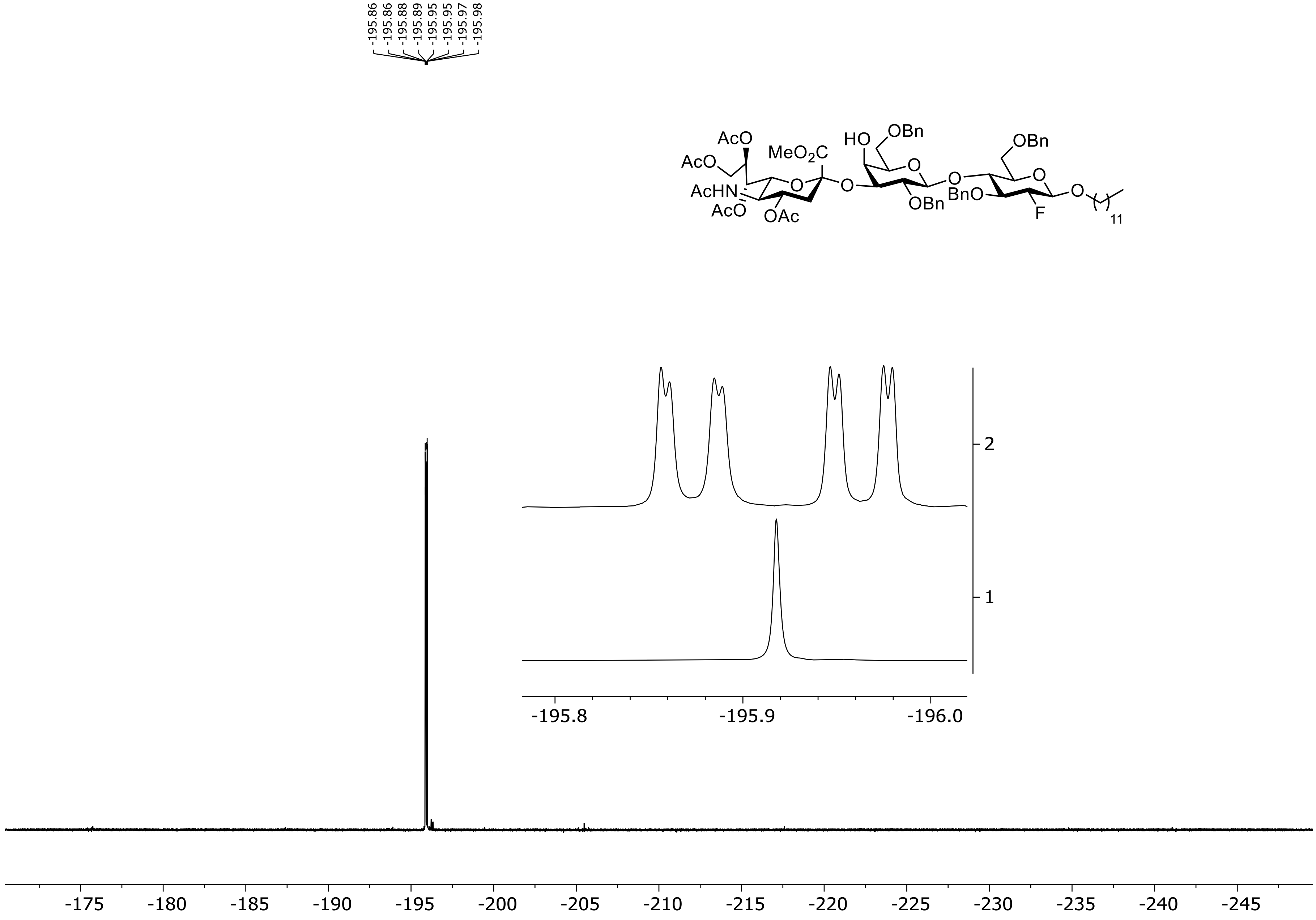

Compound **3** (HMBC, $CDCl_3$)

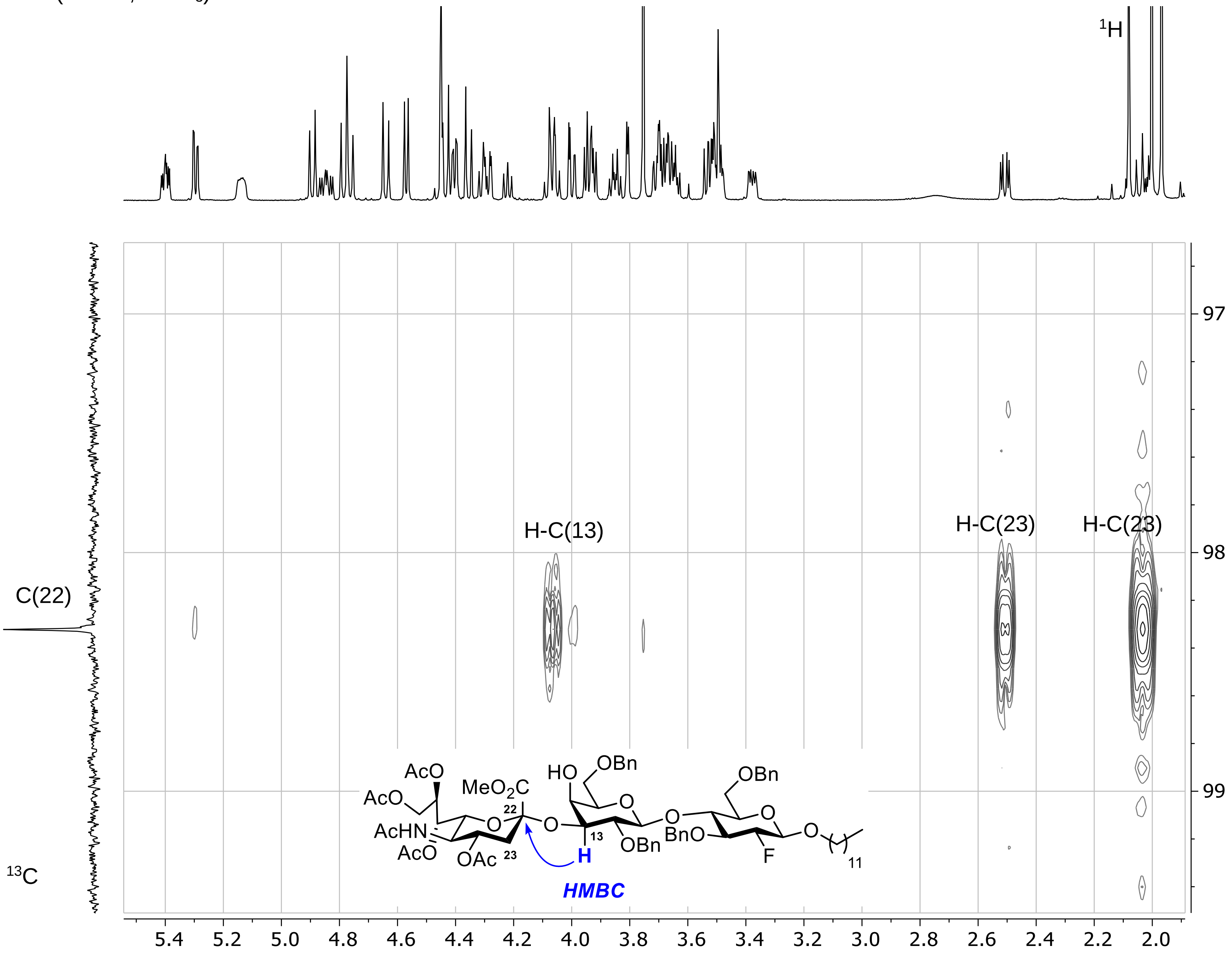

Compound **18** (^{1}H NMR, 599 MHz, $CDCl_3$)

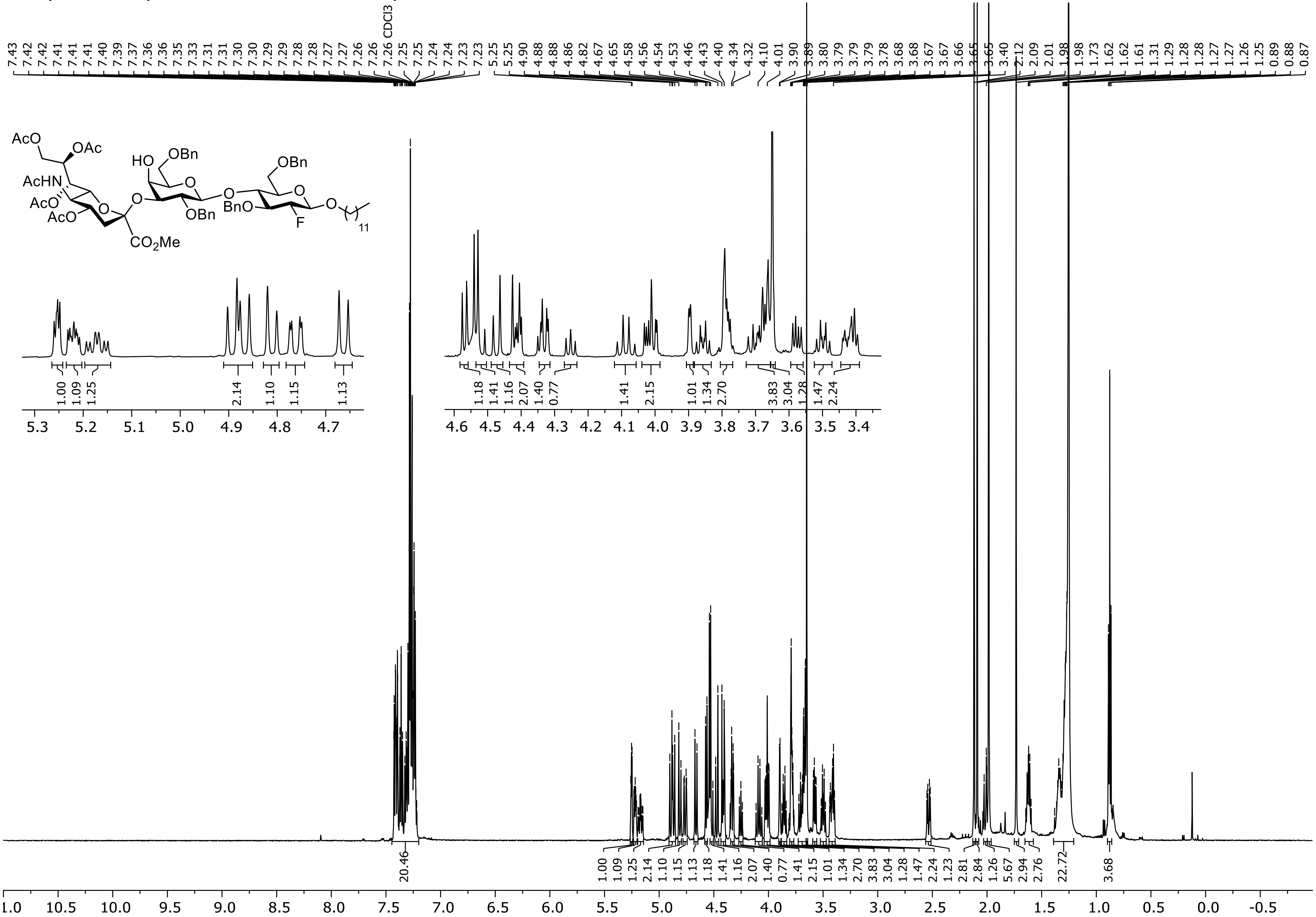

Compound **18** (^{13}C NMR, 151 MHz, $CDCl_3$)

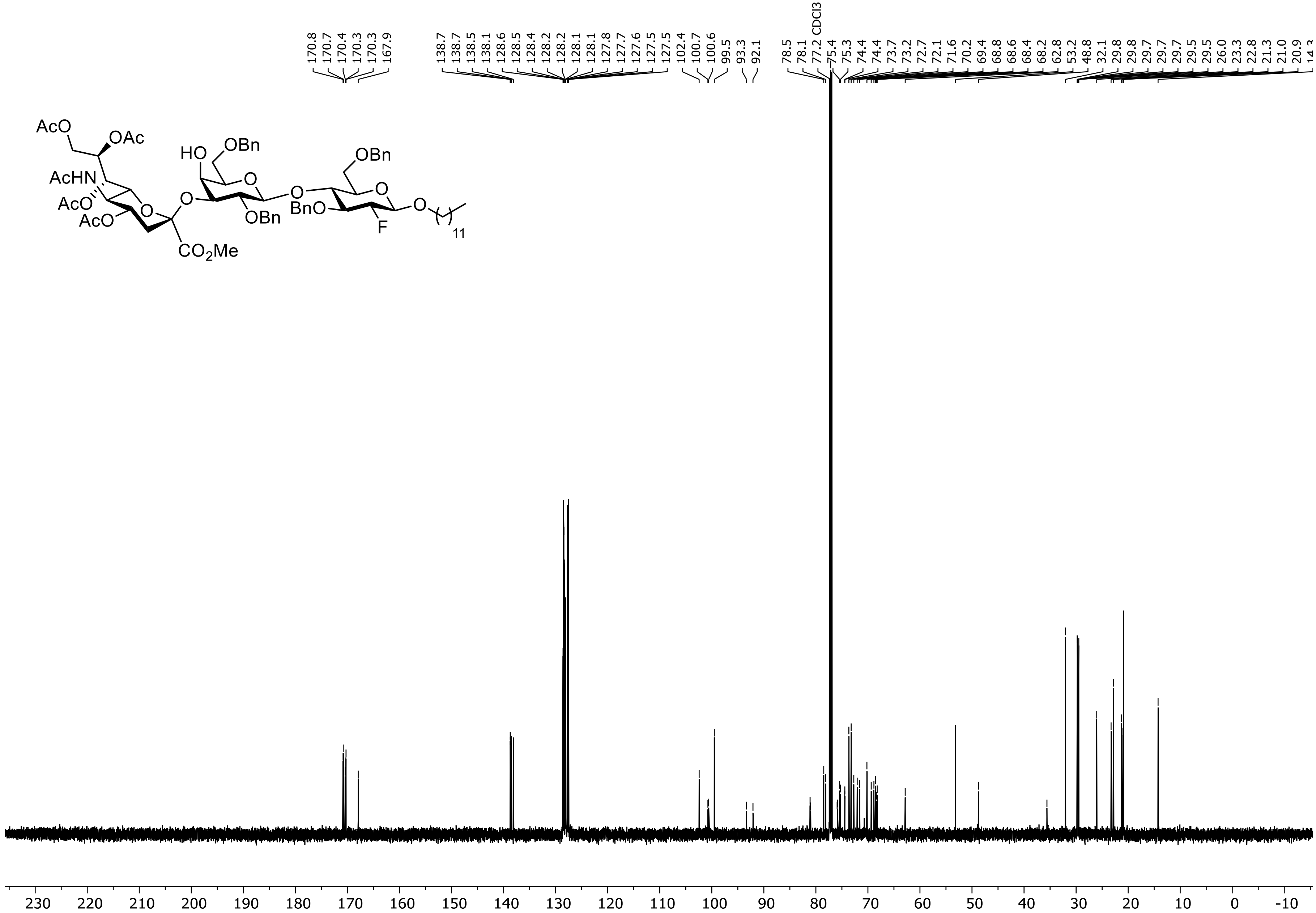

Compound **18** (^{19}F NMR, 564 MHz, $CDCl_3$) and (1) ^{19}F{^{1}H} and (2) ^{19}F NMR (564 MHz)

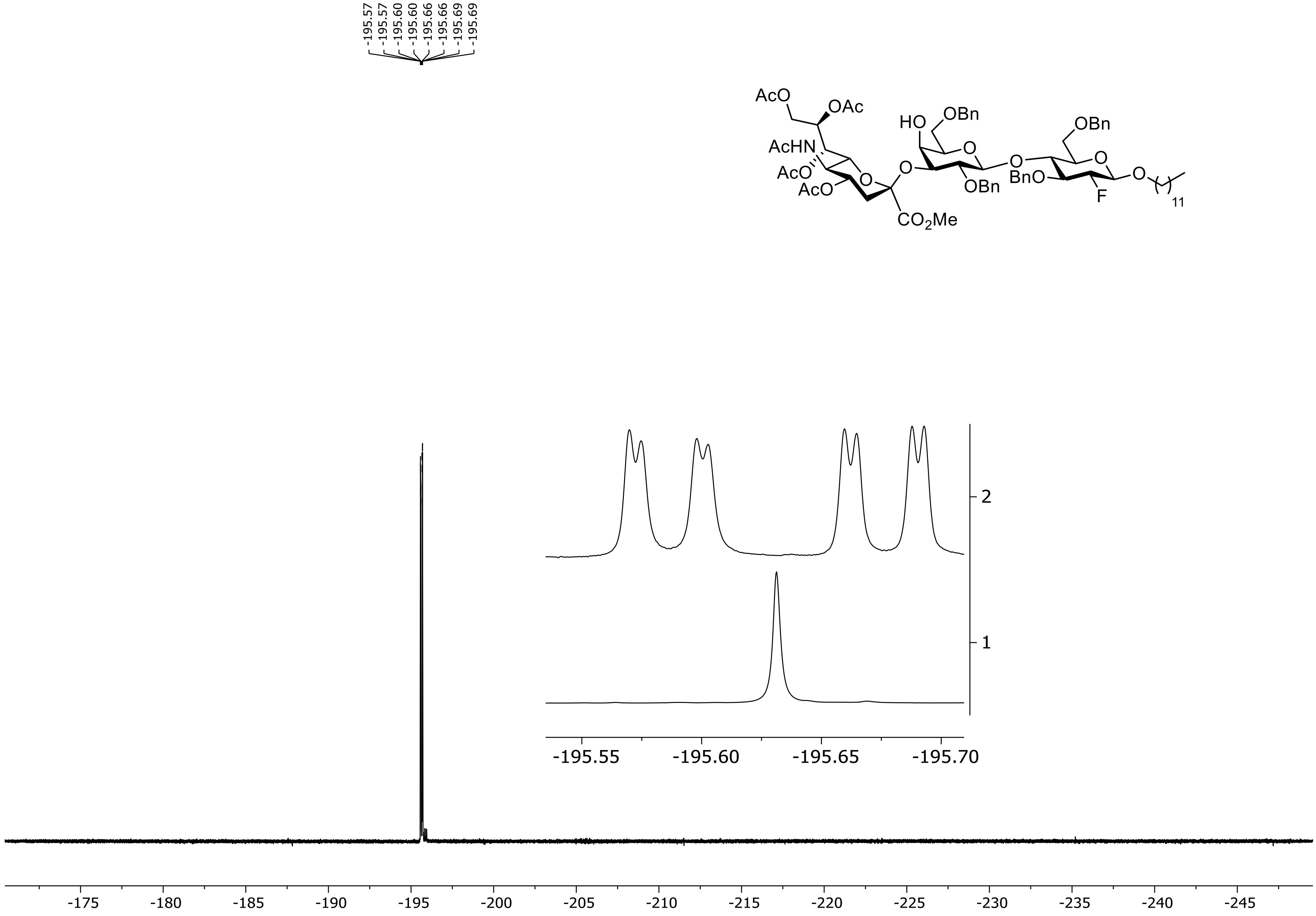

Compound **18** (HMBC, $CDCl_3$)

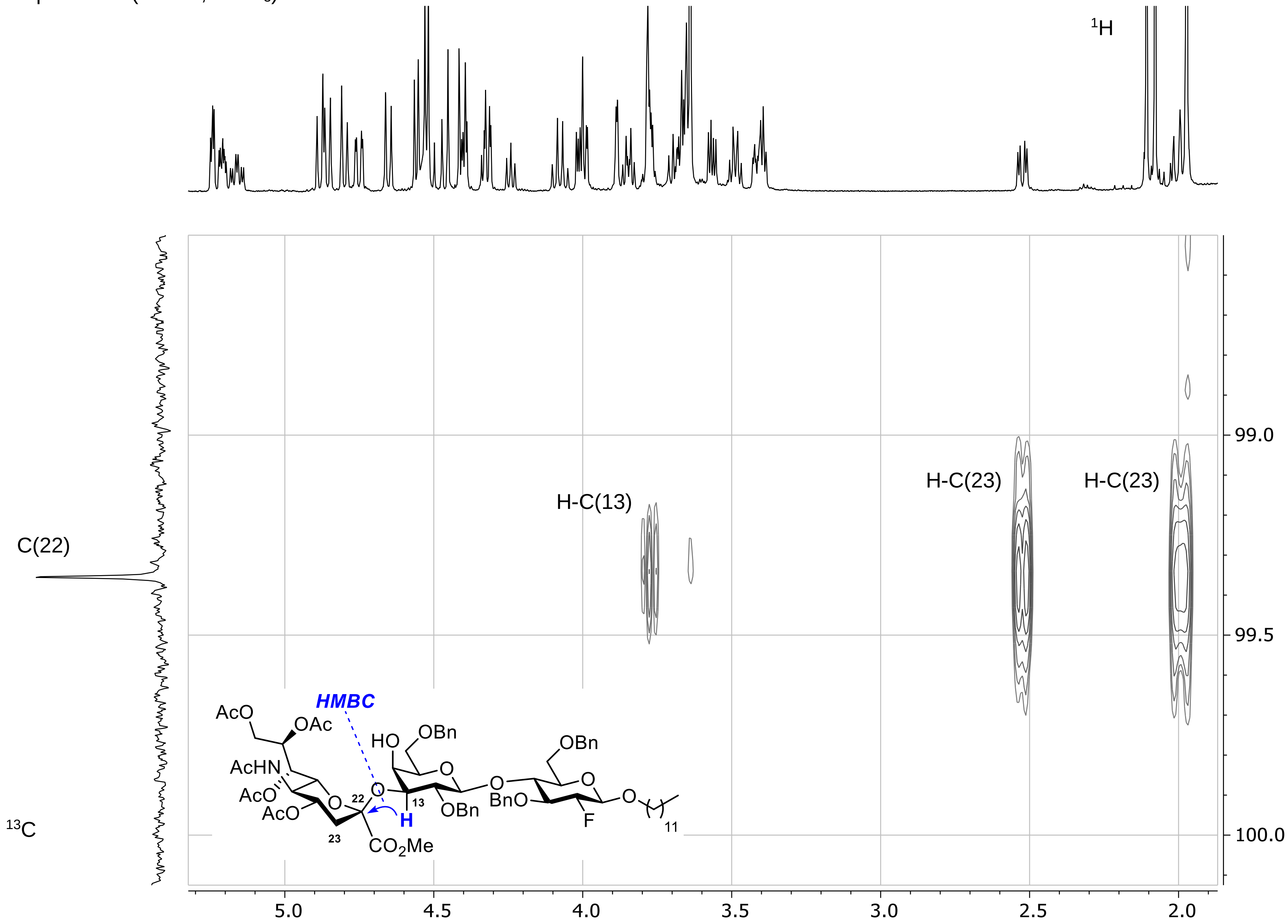

Compound **19** (^{1}H NMR, 599 MHz, $CDCl_3$)

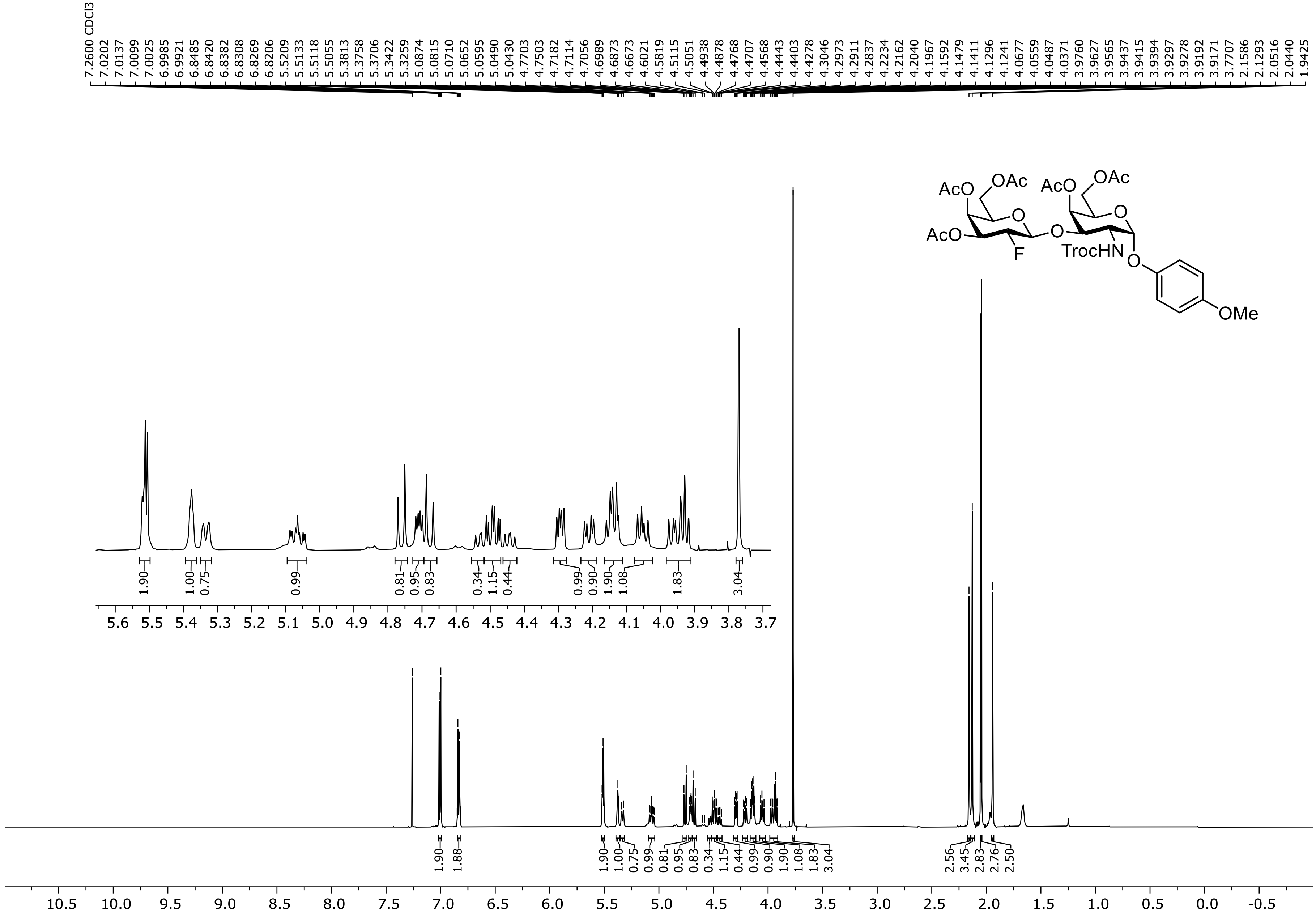

Compound **19** (^{13}C NMR, 151 MHz, $CDCl_3$)

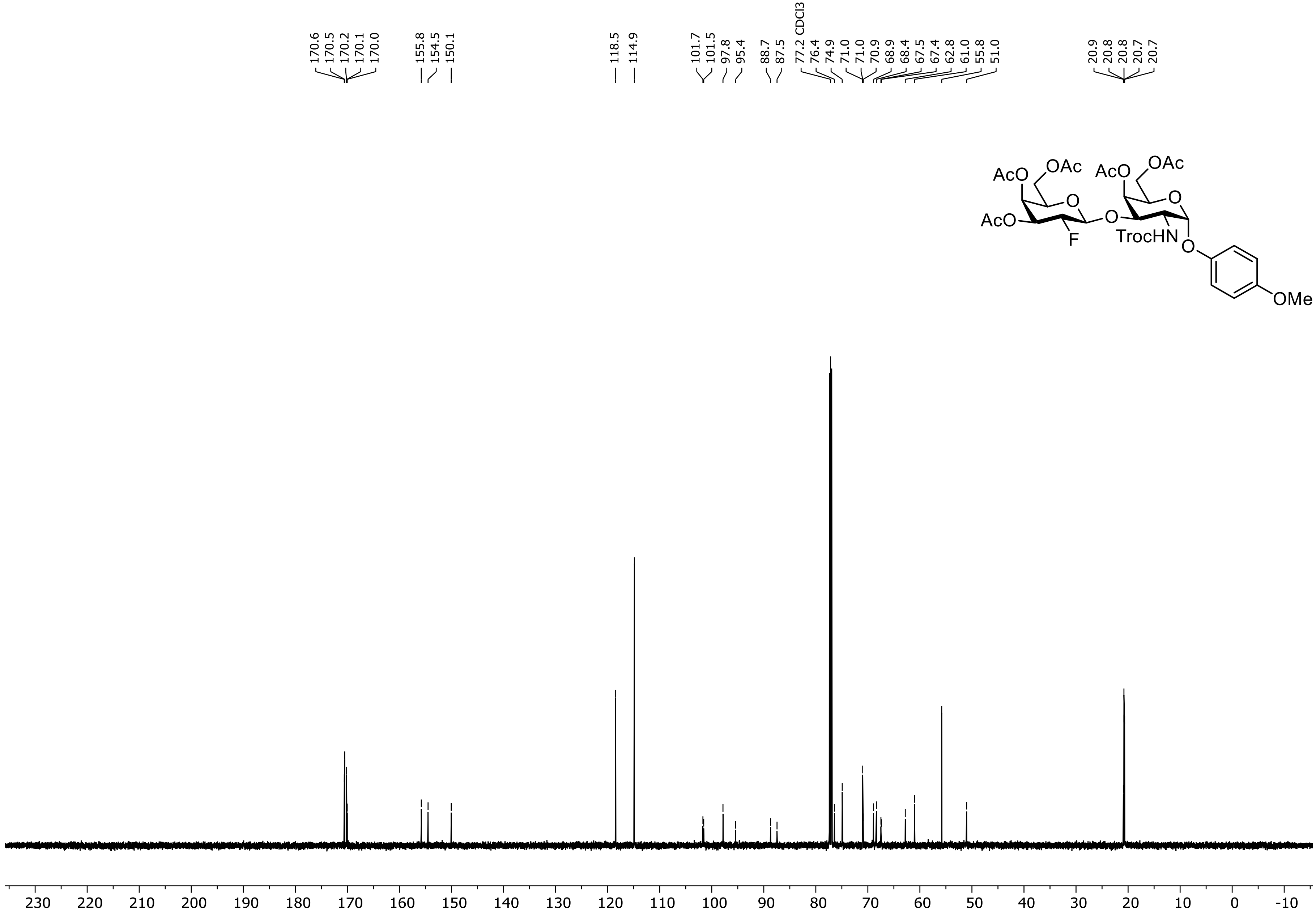

Compound **19** (^{19}F NMR, 564 MHz, $CDCl_3$) and (1) $^{19}F\{^1H\}$ and (2) ^{19}F NMR (564 MHz)

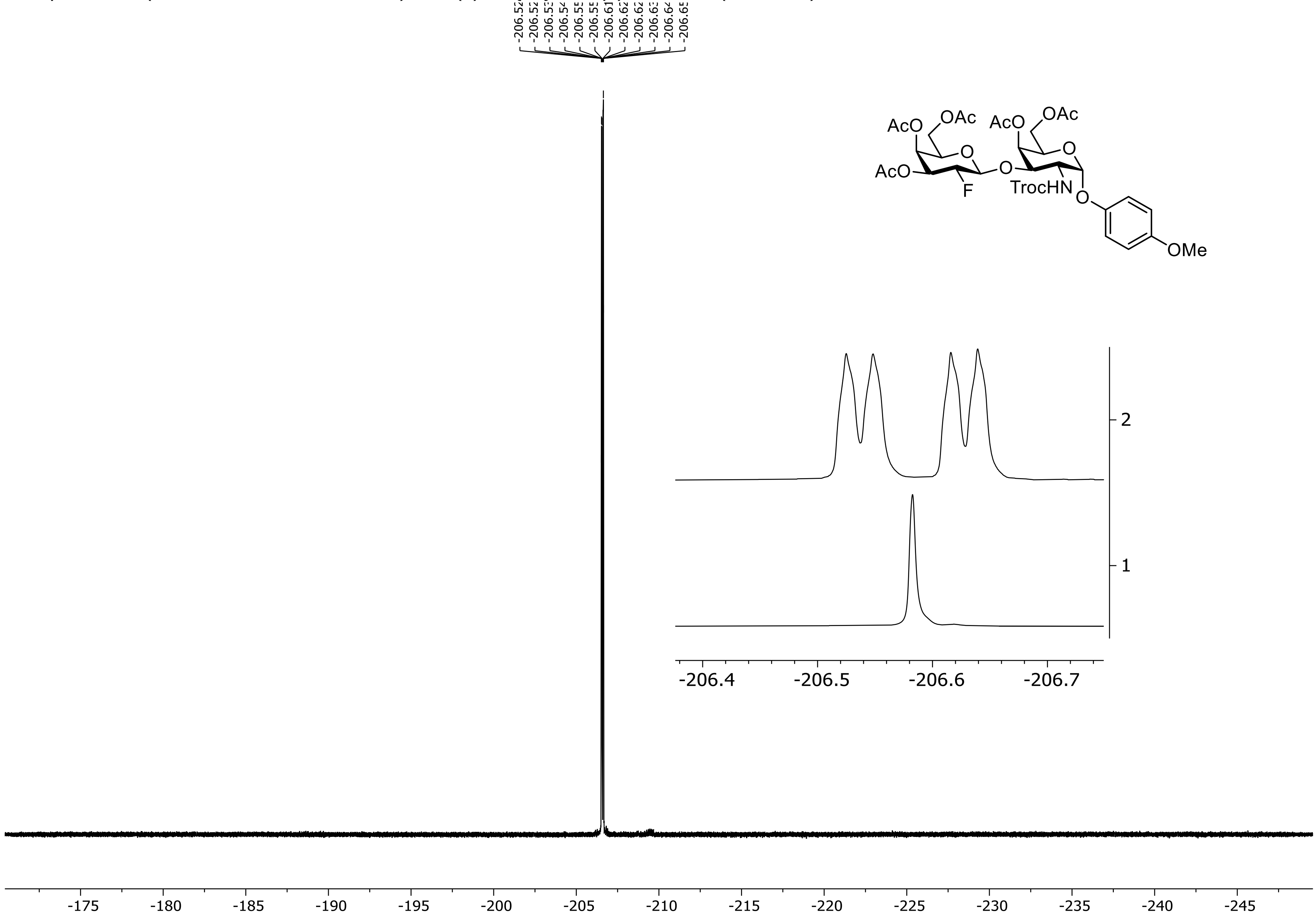

Compound **20** (^{1}H NMR, 599 MHz, $CDCl_3$)

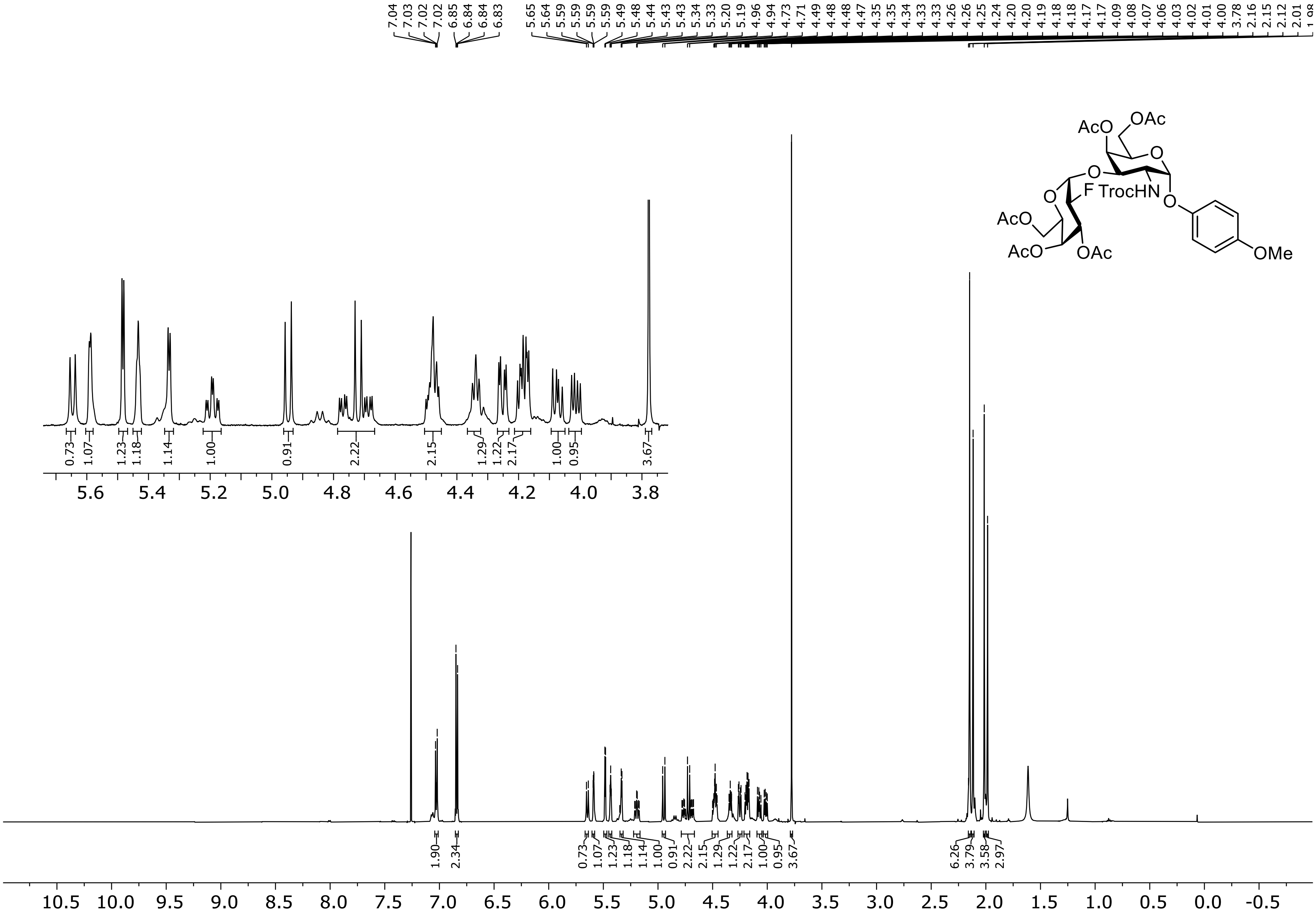

Compound **20** (^{13}C NMR, 151 MHz, $CDCl_3$)

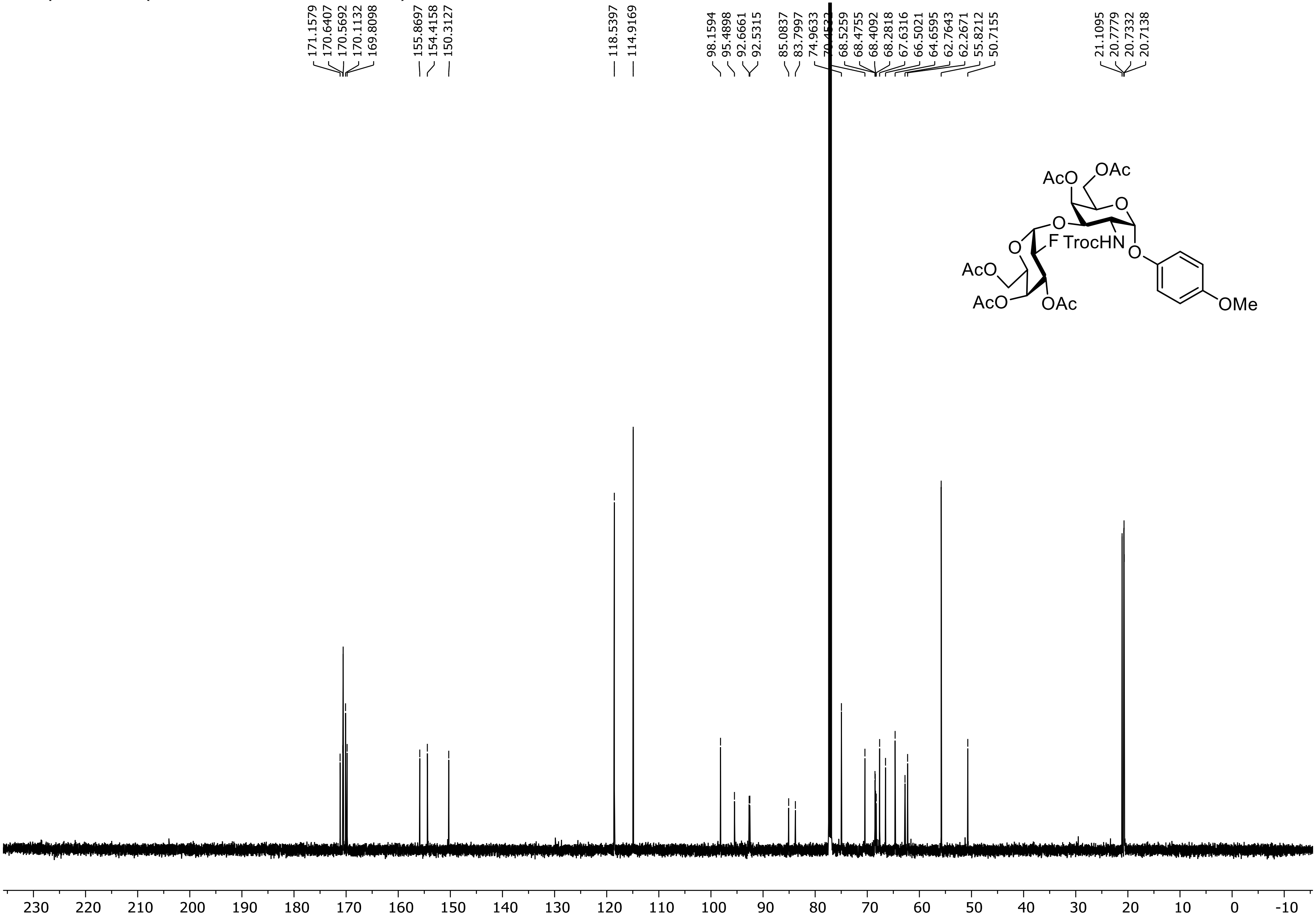

Compound **20** (^{19}F NMR, 564 MHz, $CDCl_3$) and (1) $^{19}F\{^1H\}$ and (2) ^{19}F NMR (564 MHz)

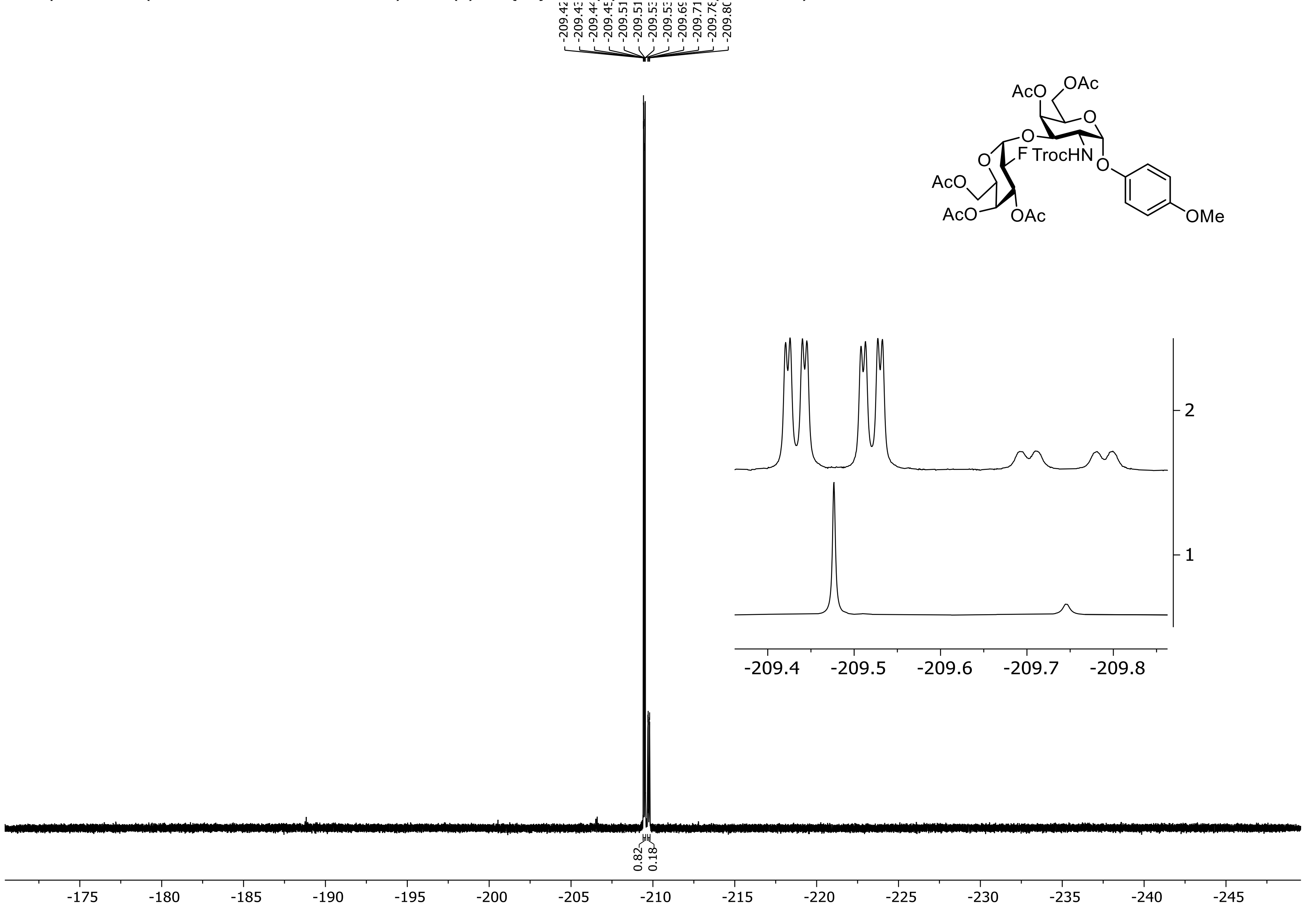

Compound **S3** (^{1}H NMR, 500 MHz, $CDCl_3$)

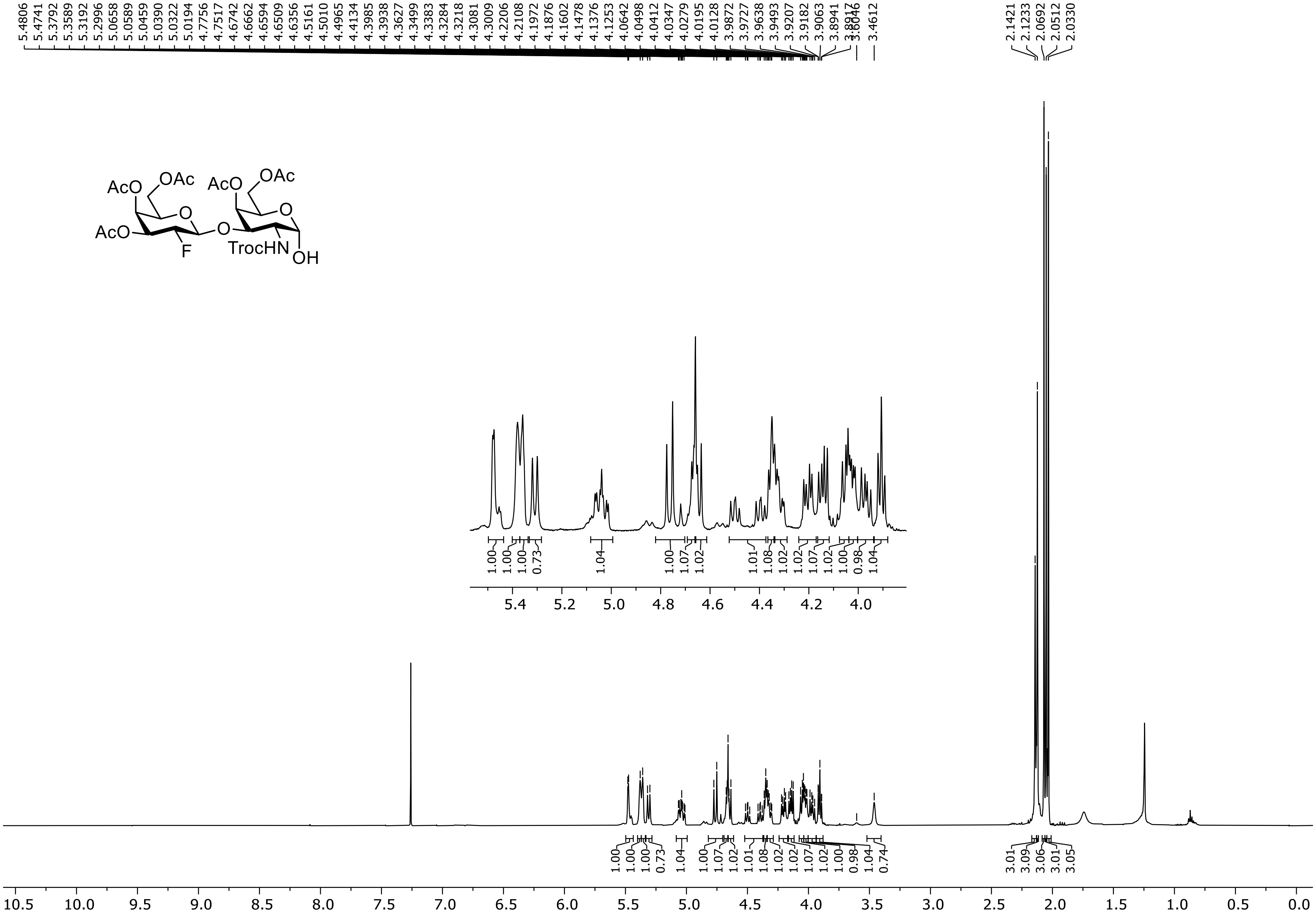

Compound **S3** (^{13}C NMR, 126 MHz, $CDCl_3$)

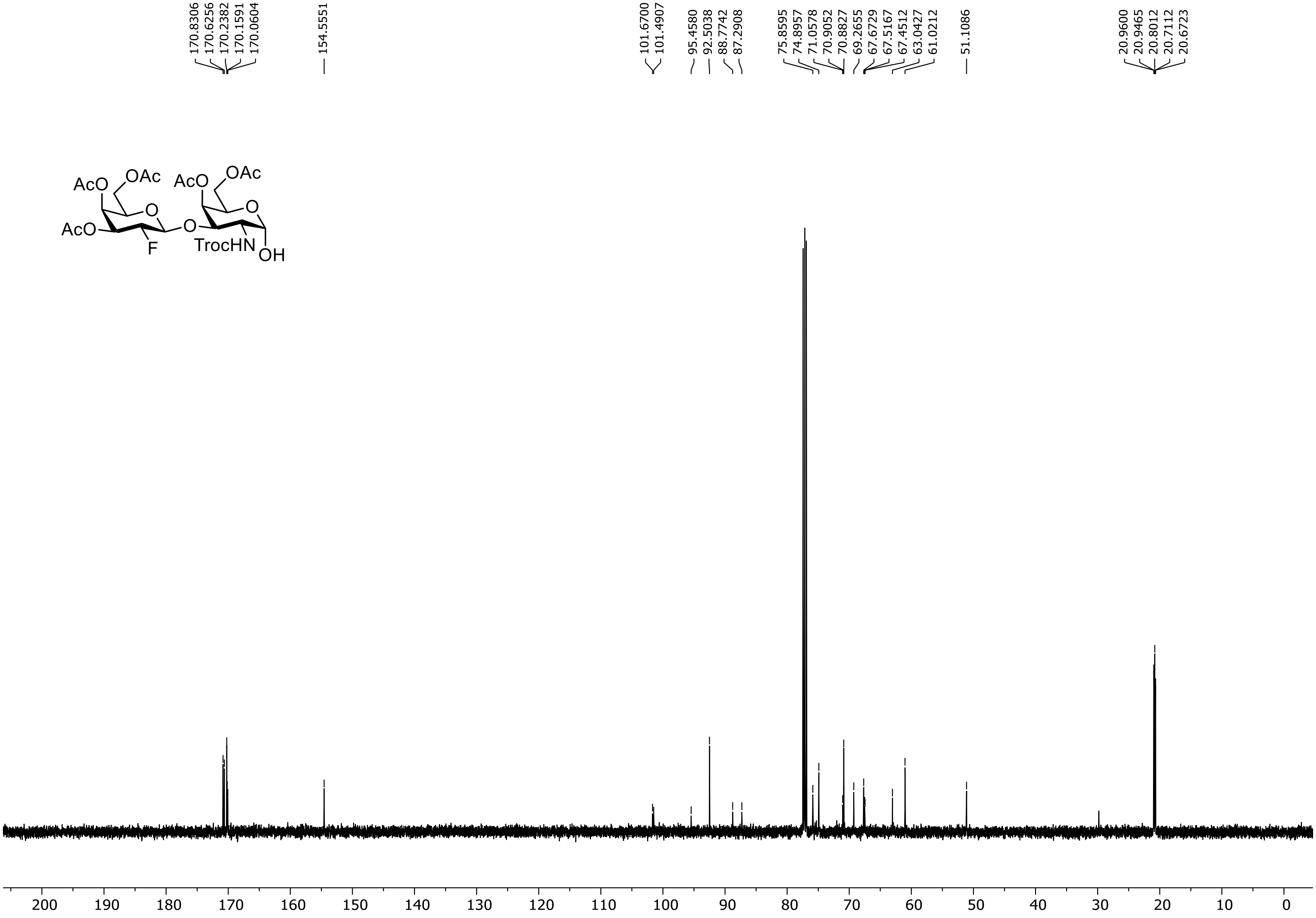

Compound **S3** (^{19}F NMR, 470 MHz, CDCl$_3$) and (1) ^{19}F{^{1}H} and (2) ^{19}F NMR (470 MHz)

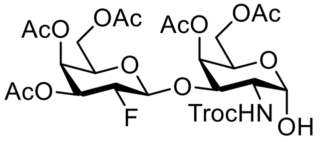

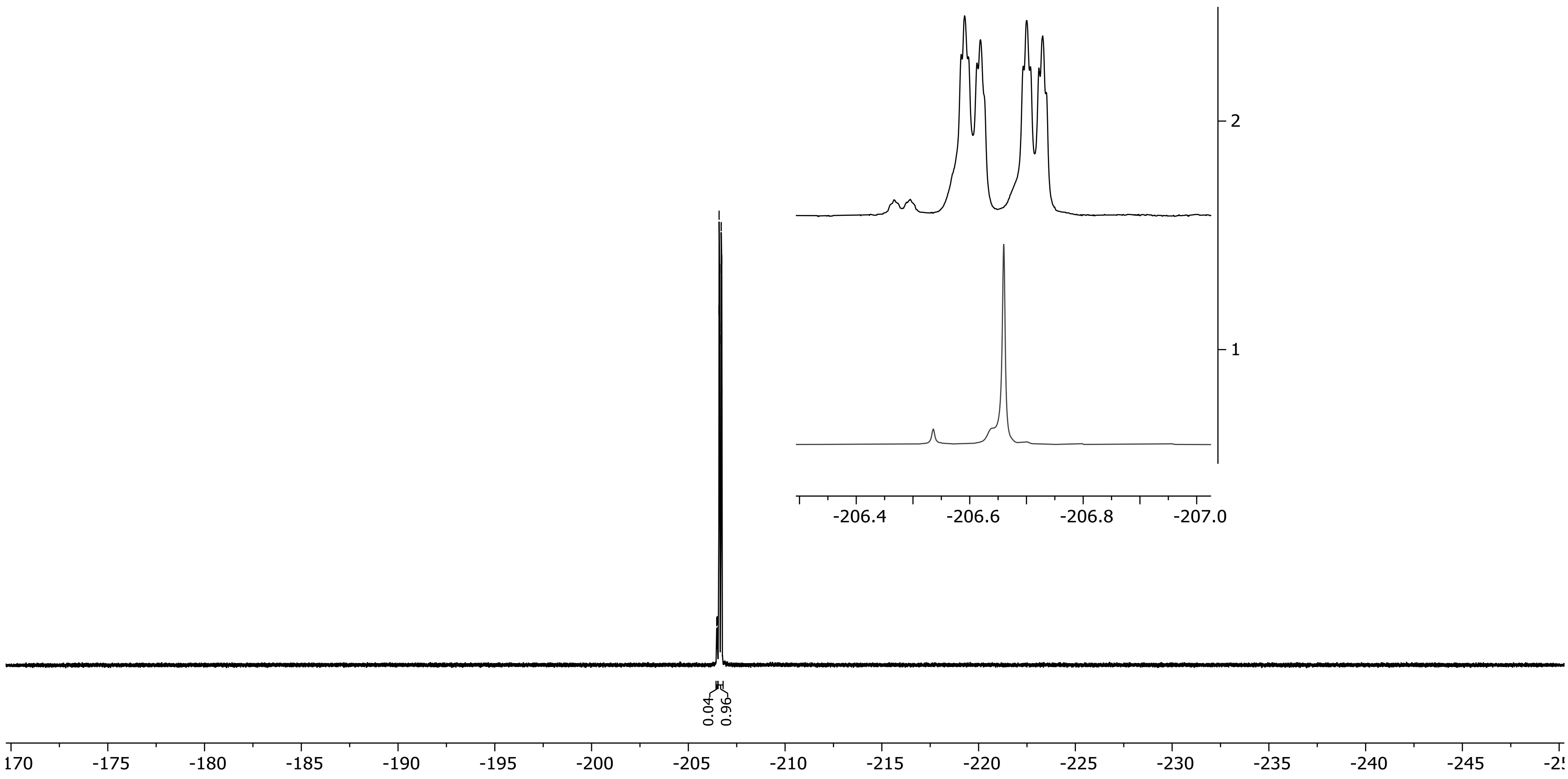

Compound **2** (^{1}H NMR, 500 MHz, CD_2Cl_2)

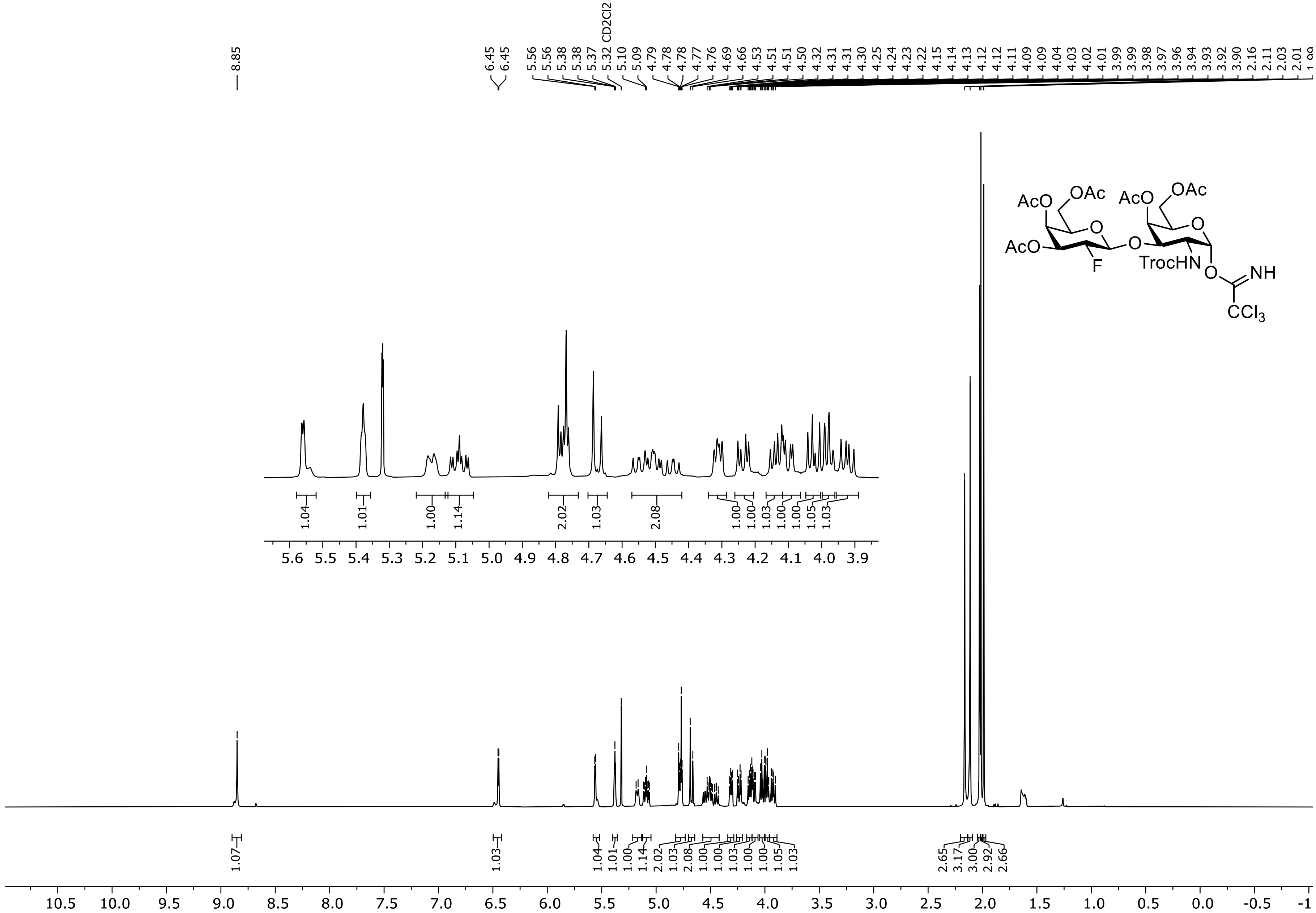

Compound **2** (^{13}C NMR, 126 MHz, CD_2Cl_2)

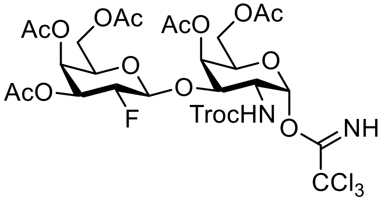

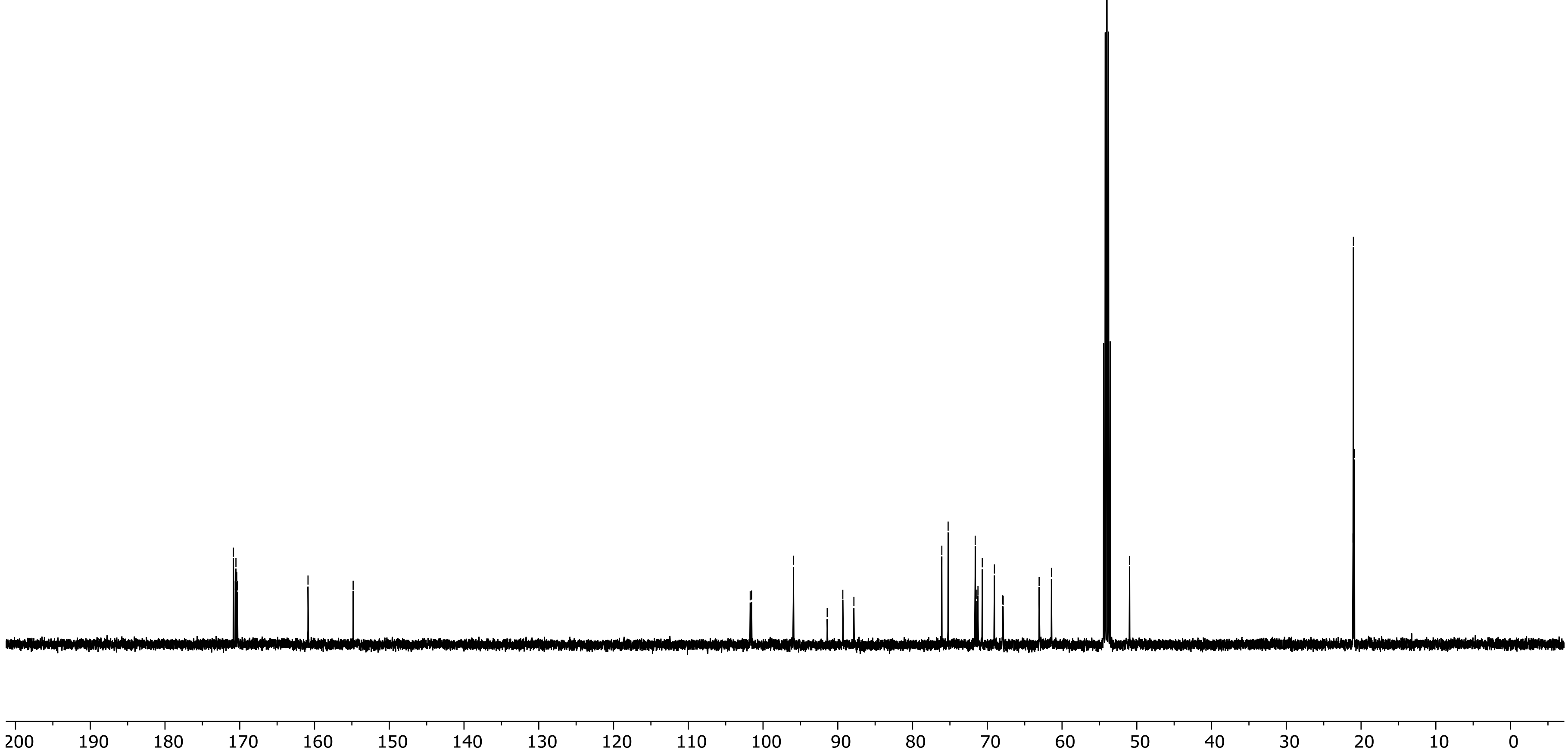

Compound **2** (^{19}F NMR (470 MHz, CD_2Cl_2) and (1) $^{19}F\{^1H\}$ and (2) ^{19}F NMR (470 MHz)

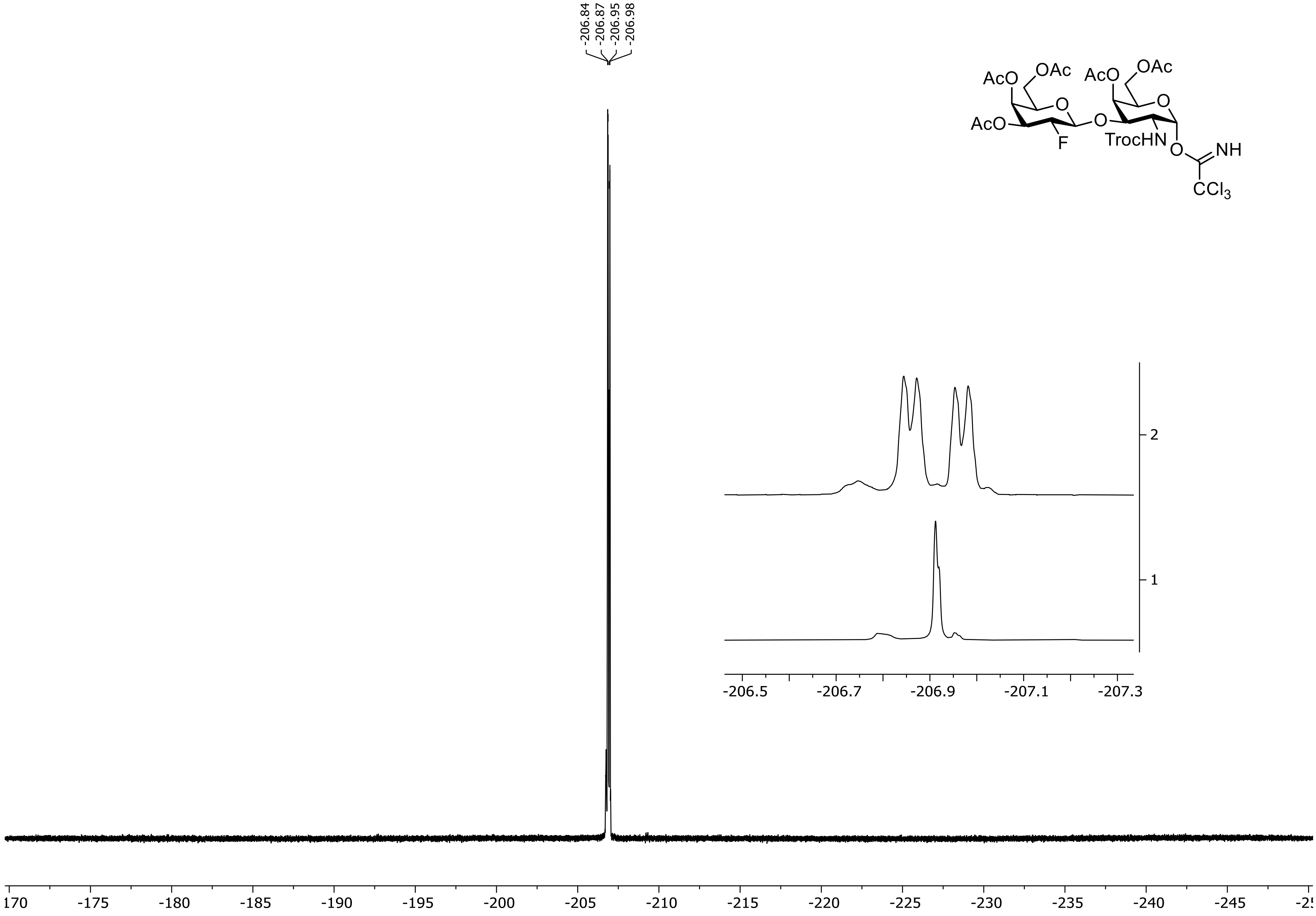

Compound **21** (^{1}H NMR, 599 MHz, $CDCl_3$)

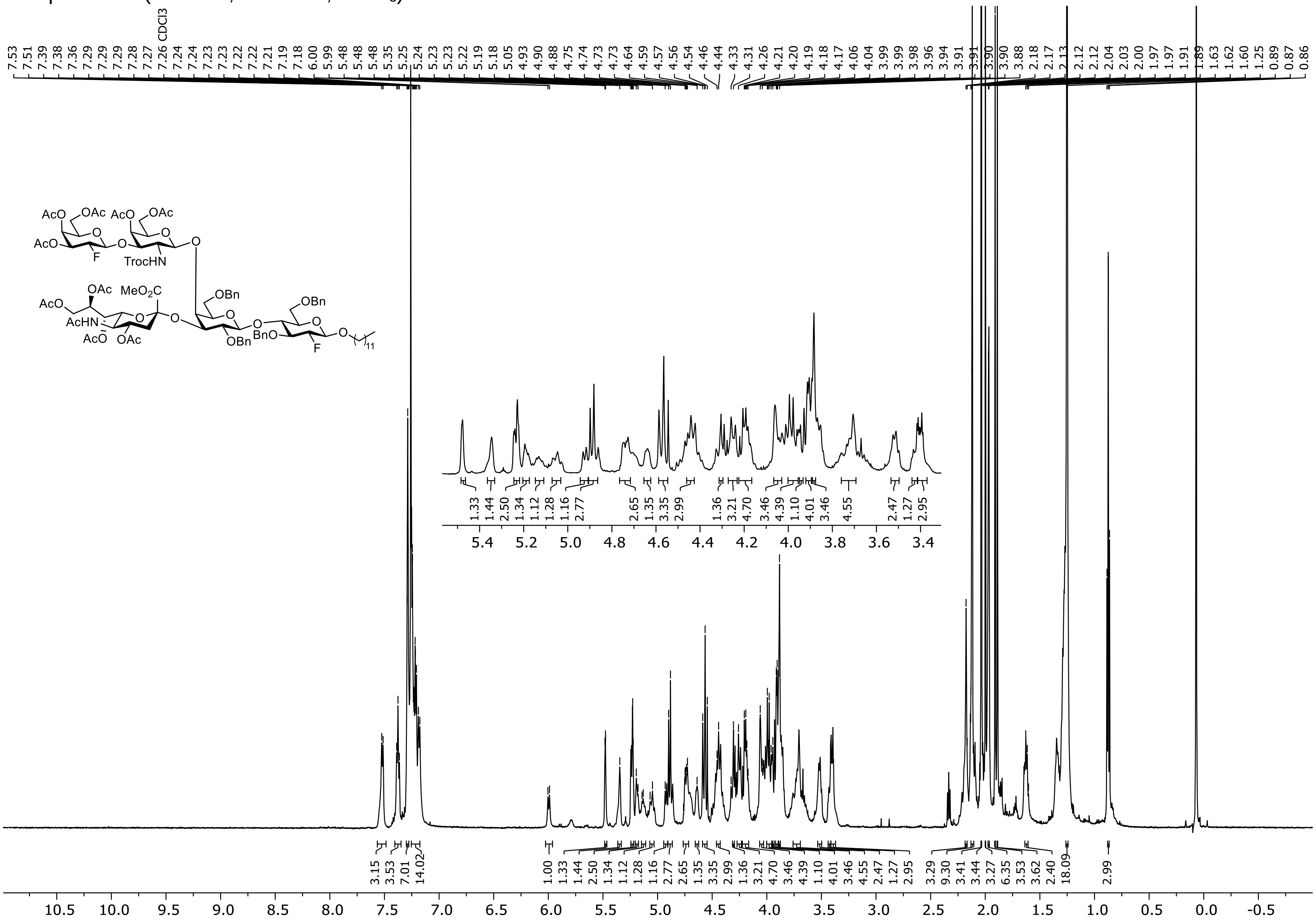

Compound **21** (^{13}C NMR, 151 MHz, $CDCl_3$)

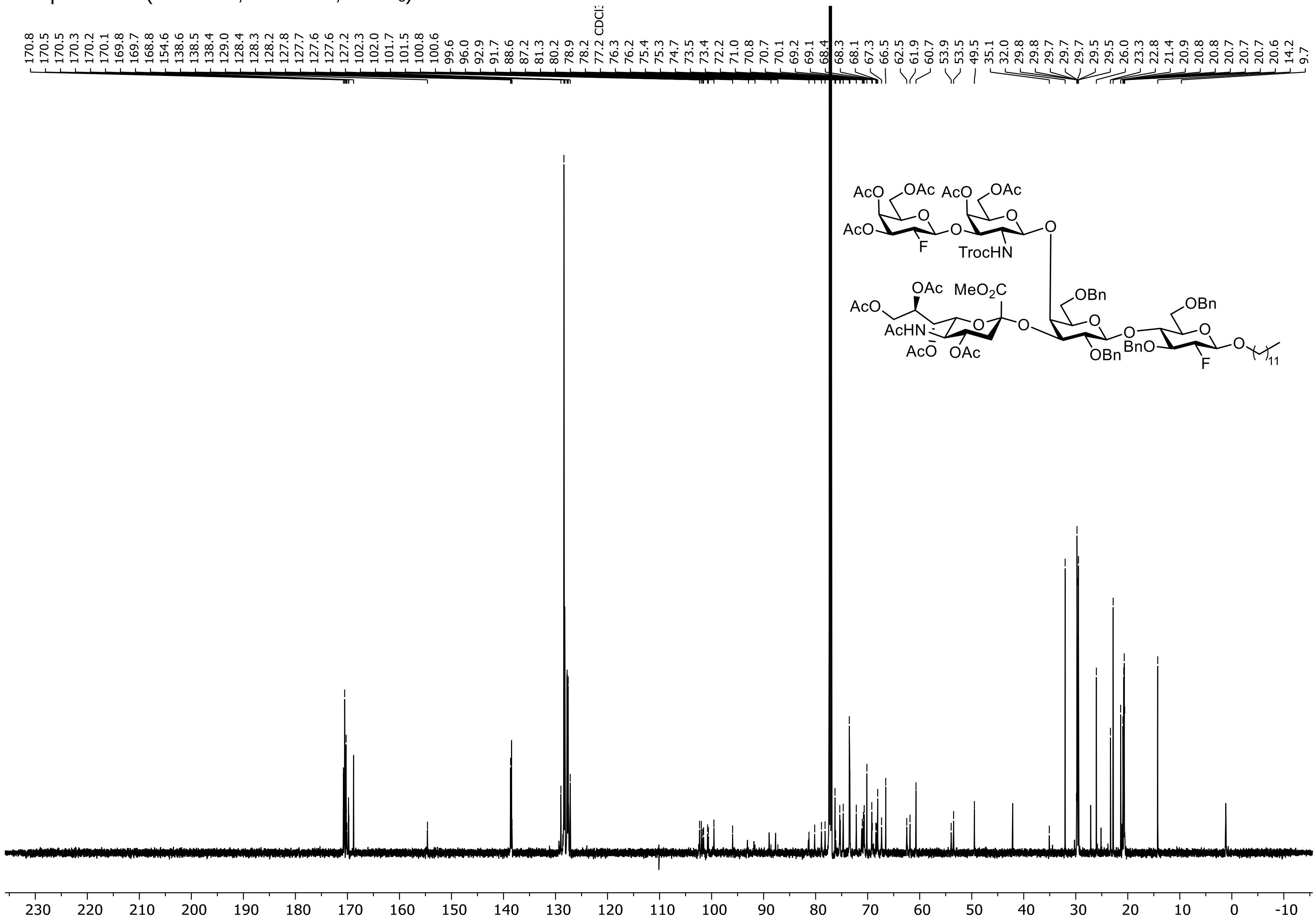

Compound **21** (^{19}F NMR, 564 MHz, $CDCl_3$) and (1) $^{19}F\{^1H\}$ and (2) ^{19}F NMR (564 MHz)

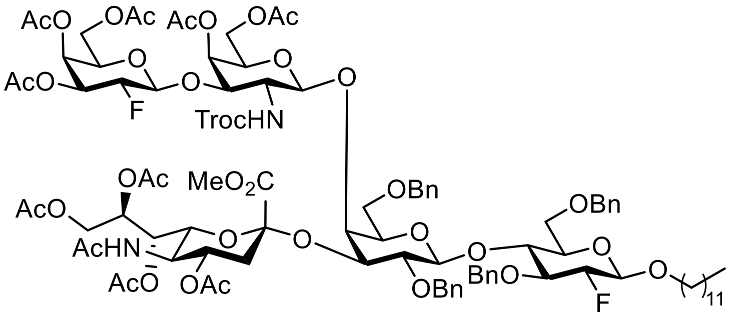

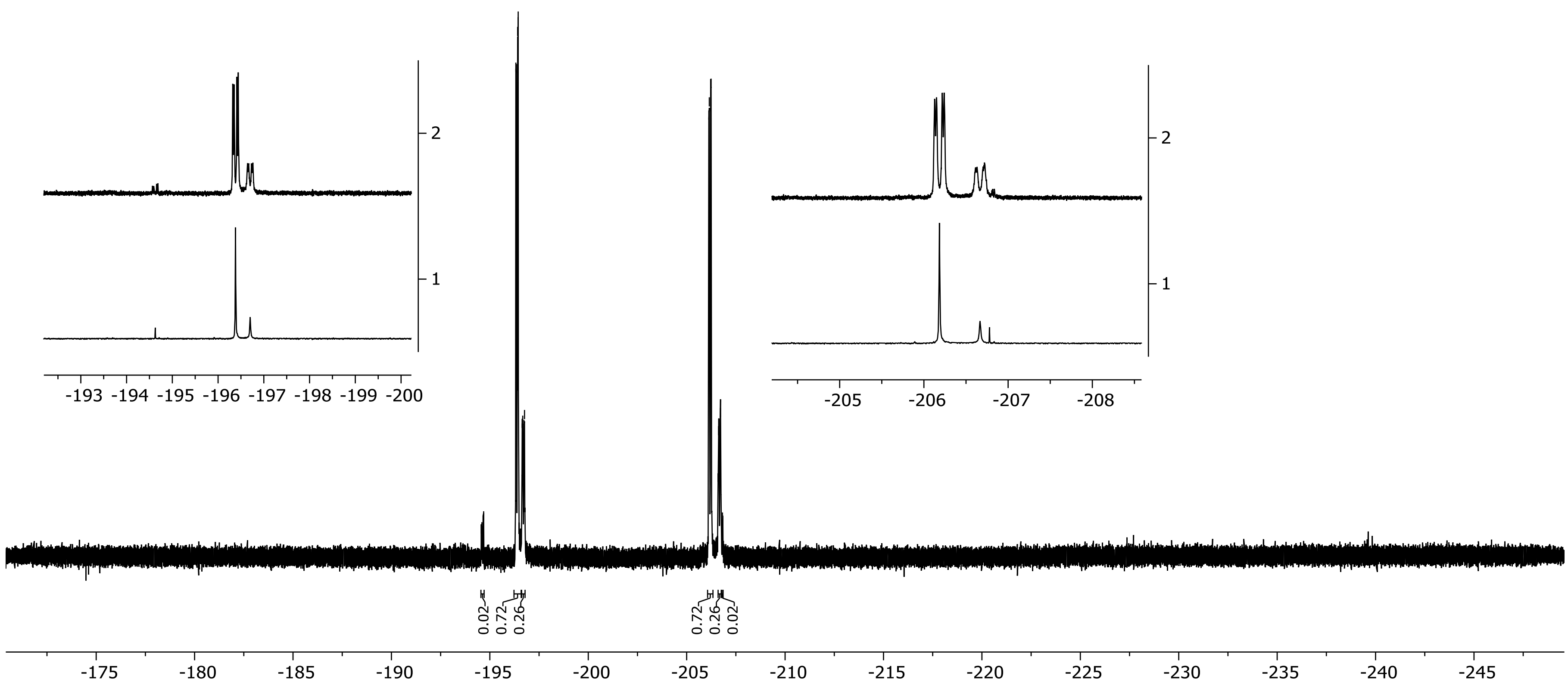

Compound **21** (^{1}H-^{1}H-COSY, $CDCl_3$)

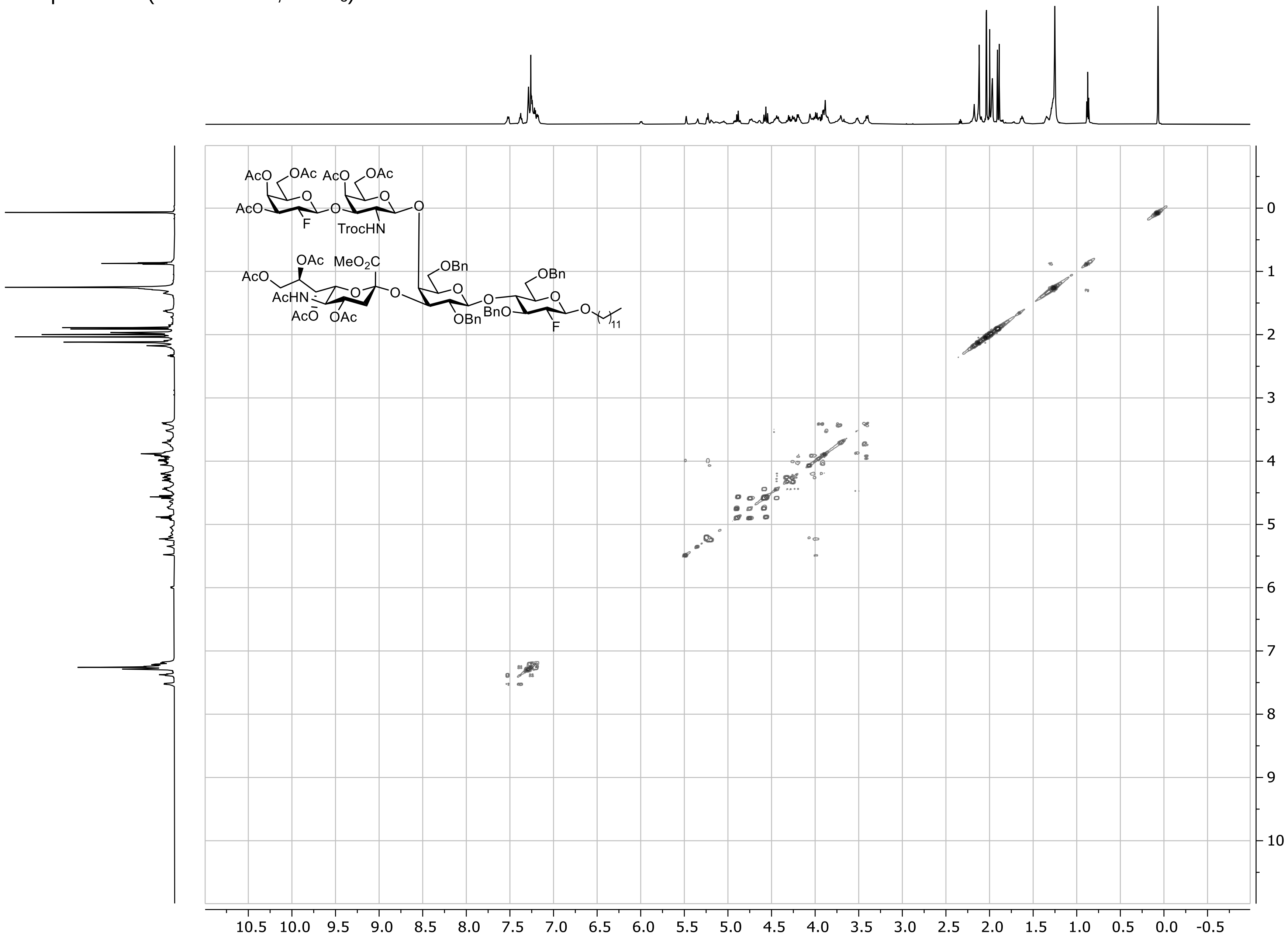

Compound **21** (^{1}H-^{13}H-gHSQC, $CDCl_3$)

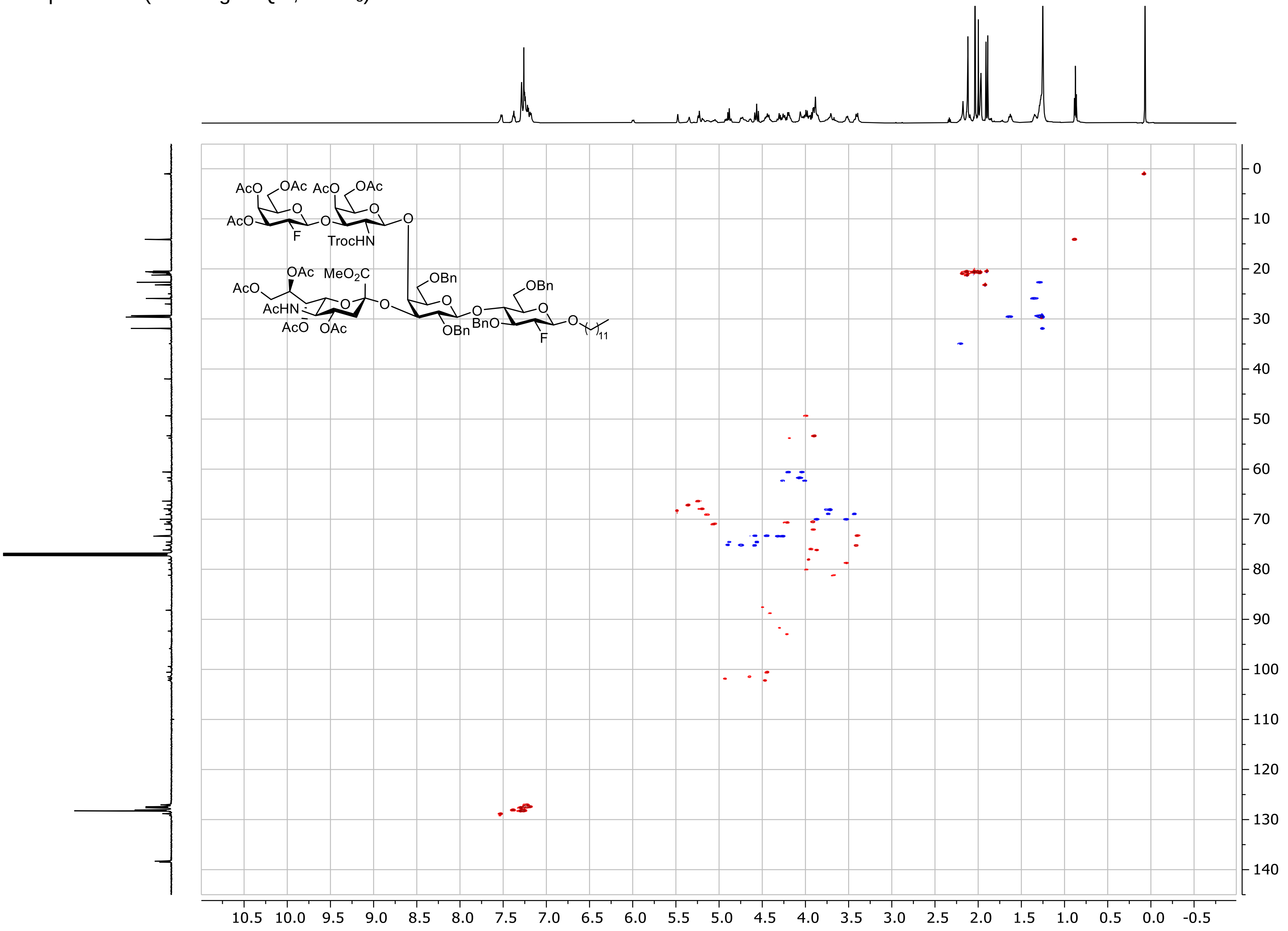

Compound **21** (^{1}H-^{13}C-gHMBC, $CDCl_3$)

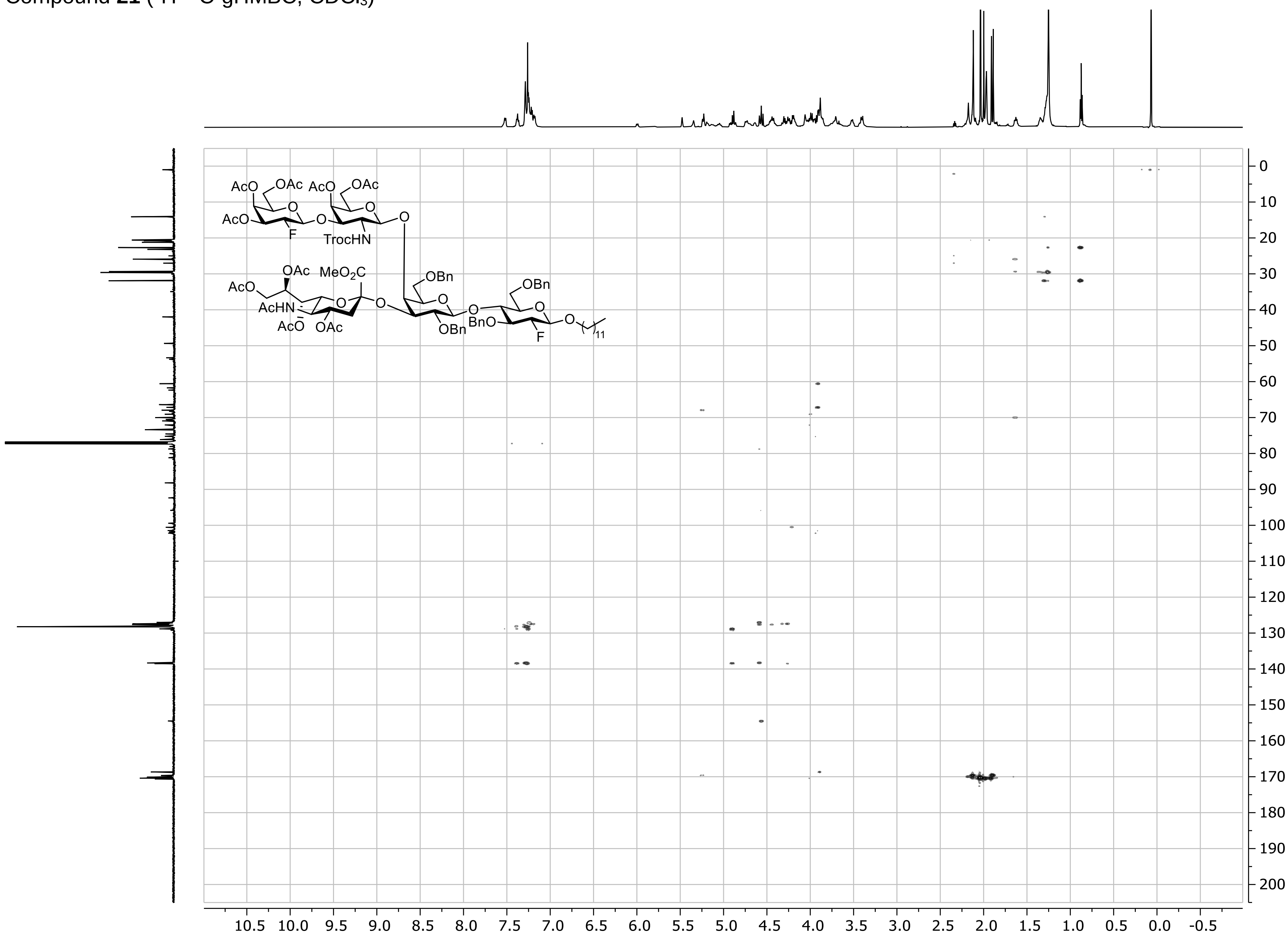

Compound **22** (^{1}H NMR, 599 MHz, $CDCl_3$)

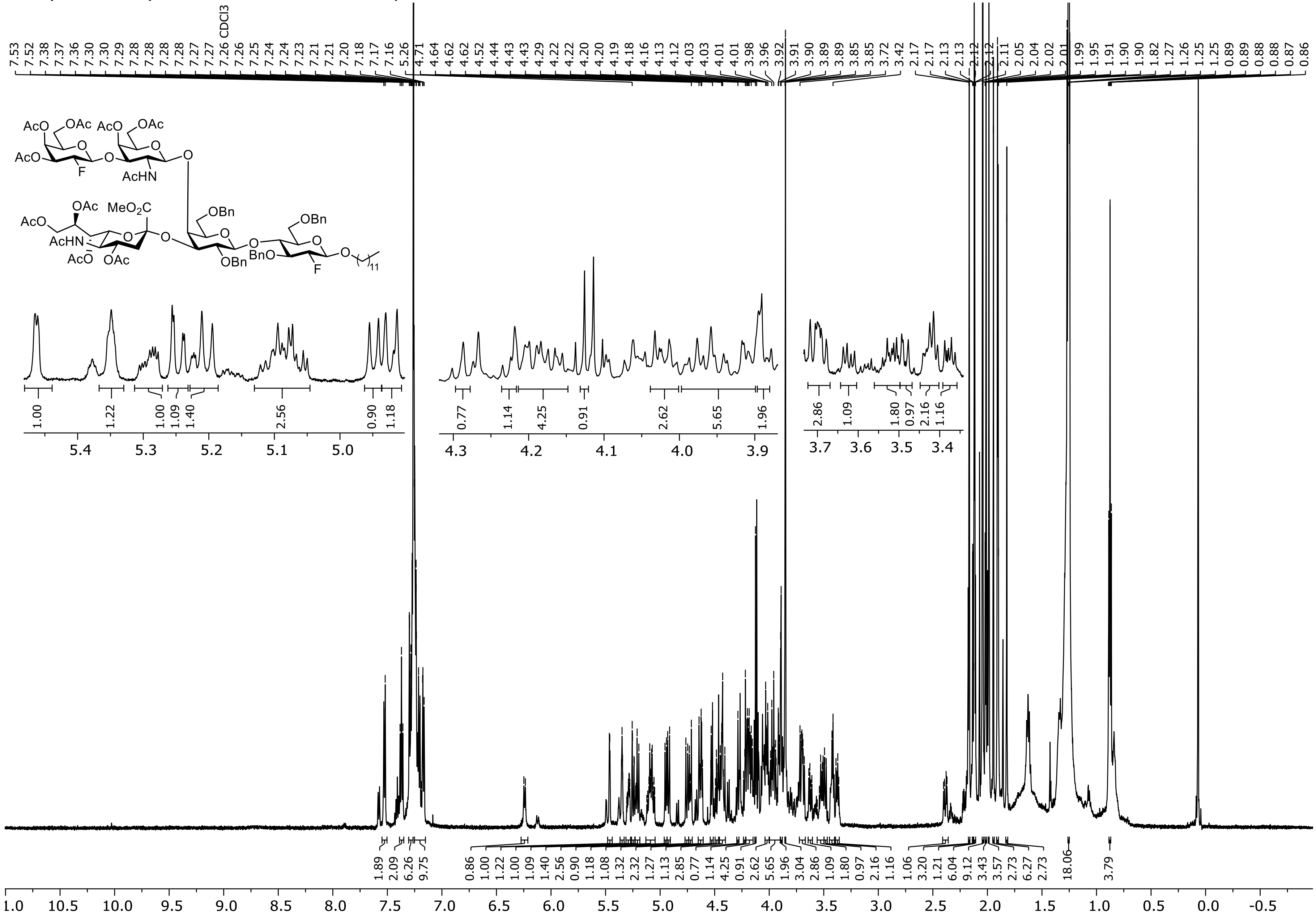

Compound **22** (^{13}C NMR, 151 MHz, $CDCl_3$)

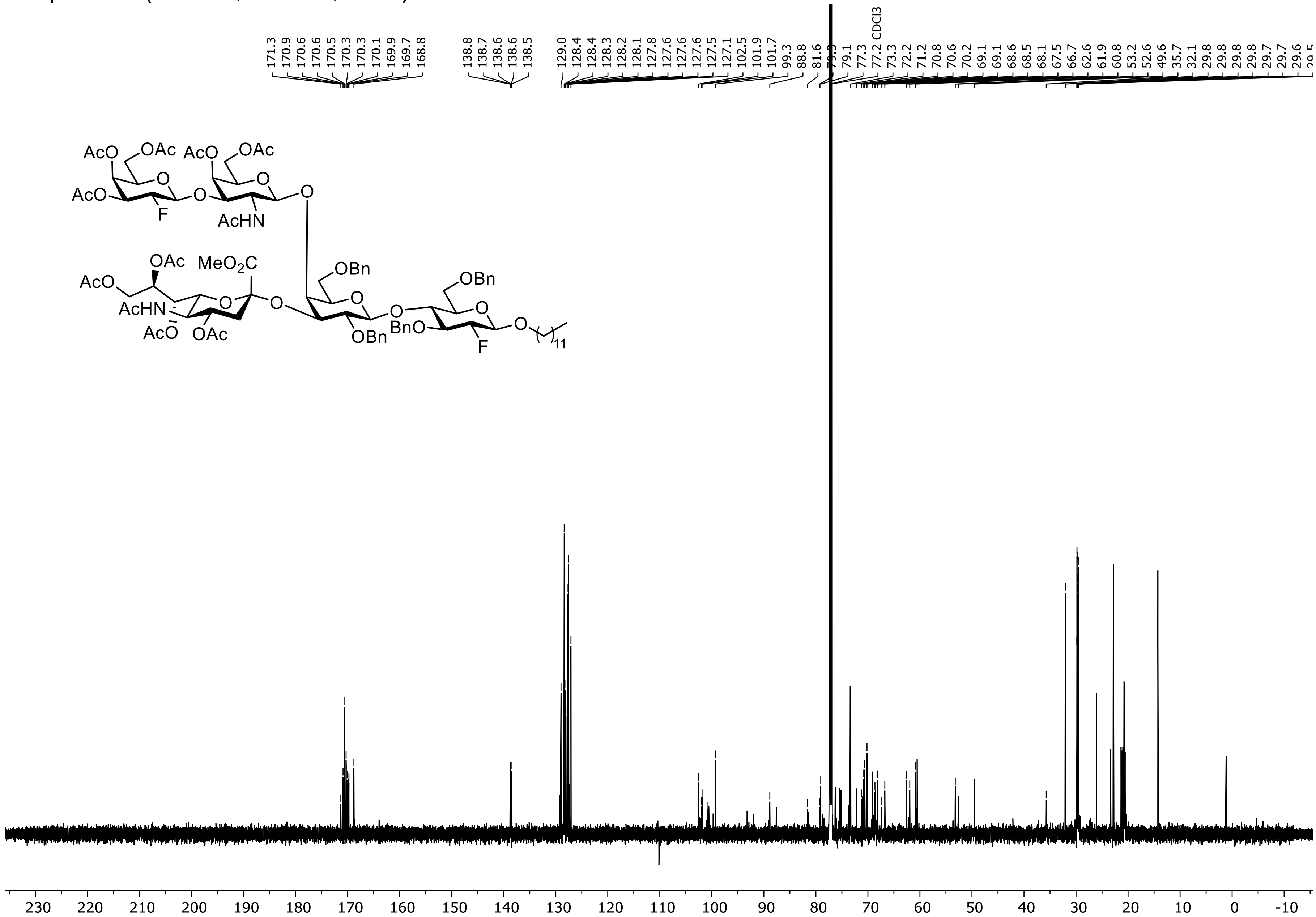

Compound **22** (^{19}F{^{1}H} NMR, 564 MHz, $CDCl_3$) and (1) ^{19}F{^{1}H} and (2) ^{19}F NMR (564 MHz)

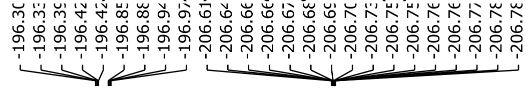

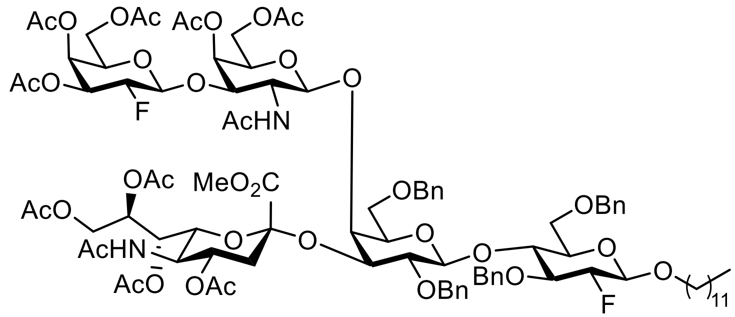

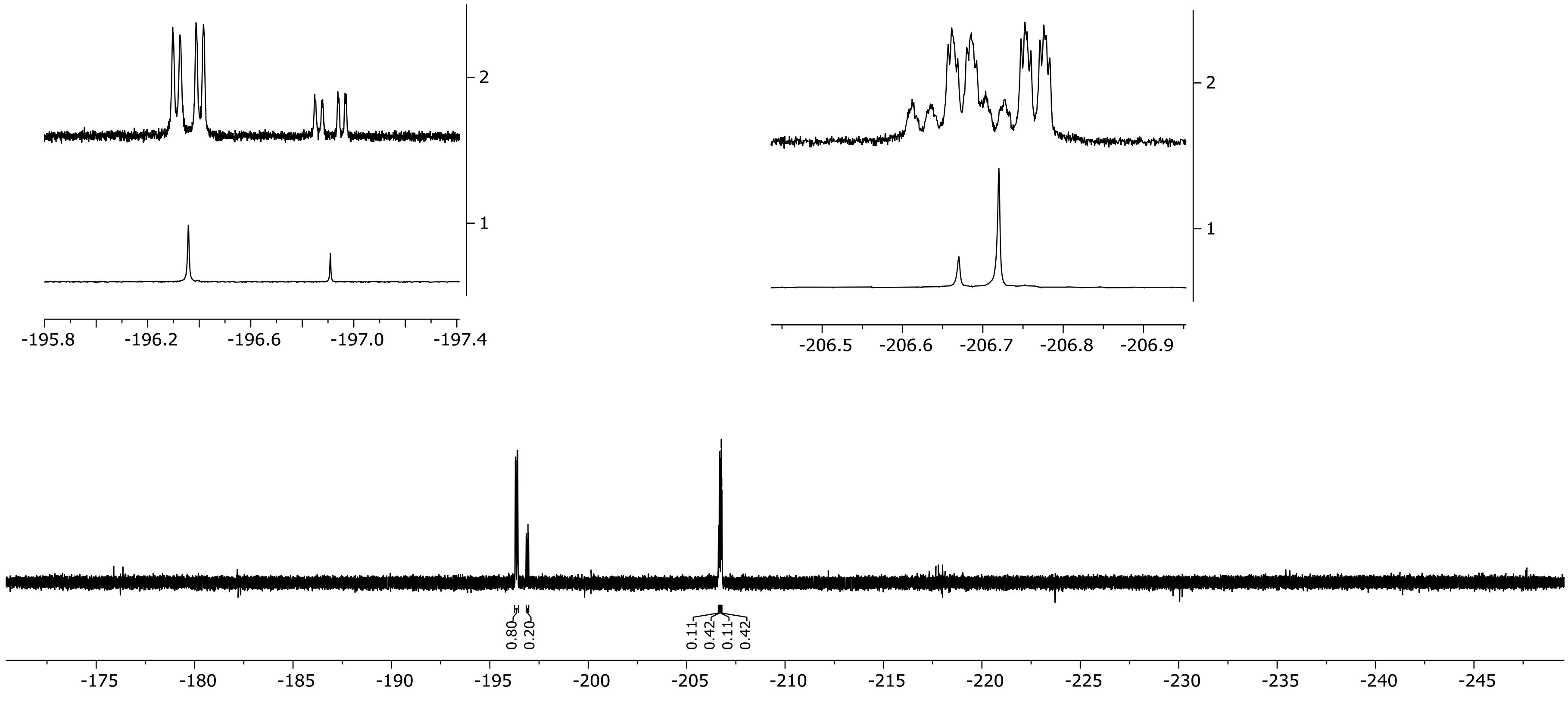

Compound **22** (^{1}H-^{1}H-COSY, $CDCl_3$)

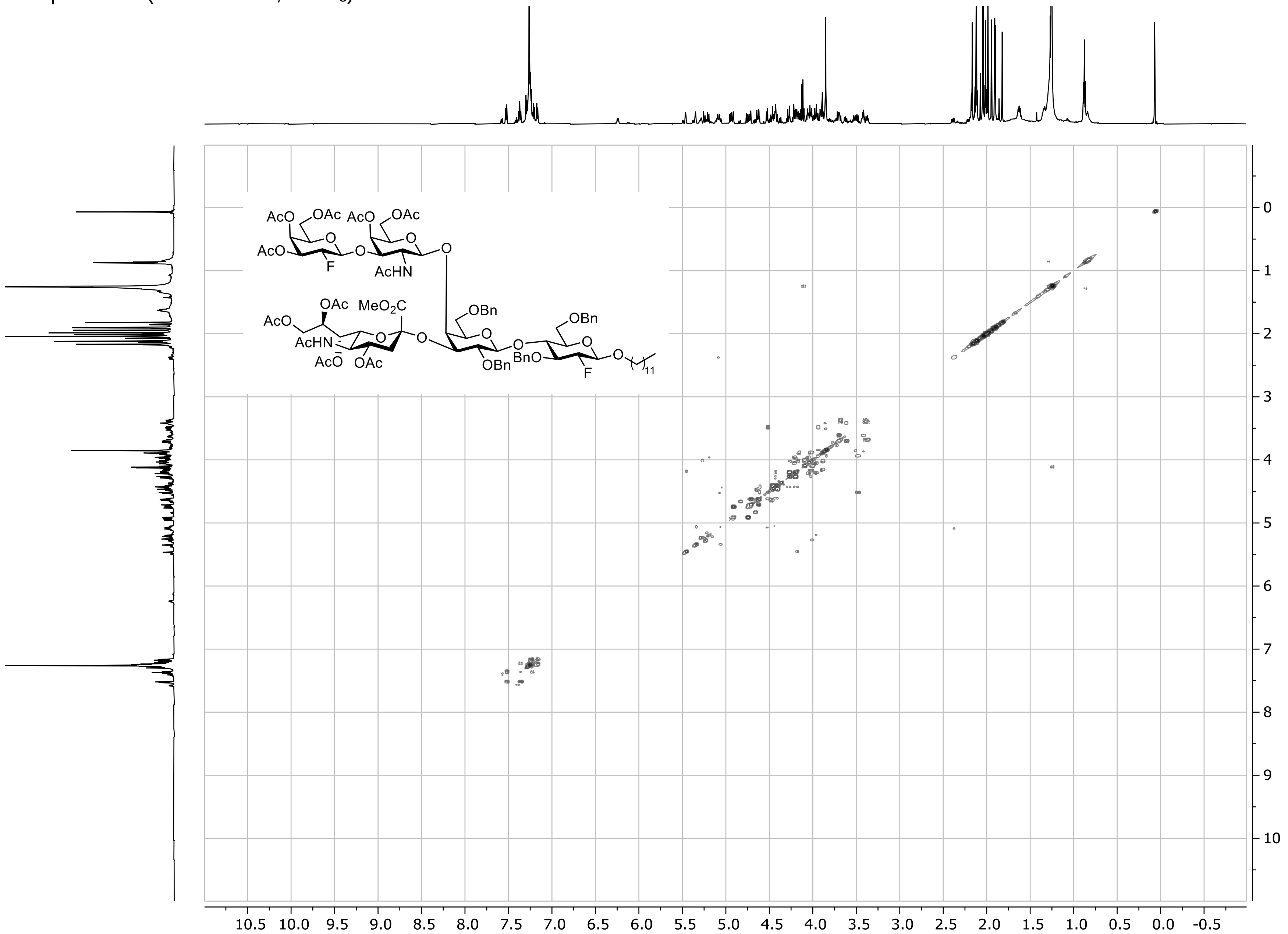

Compound **22** (^{1}H-^{13}C-gHSQC, $CDCl_3$)

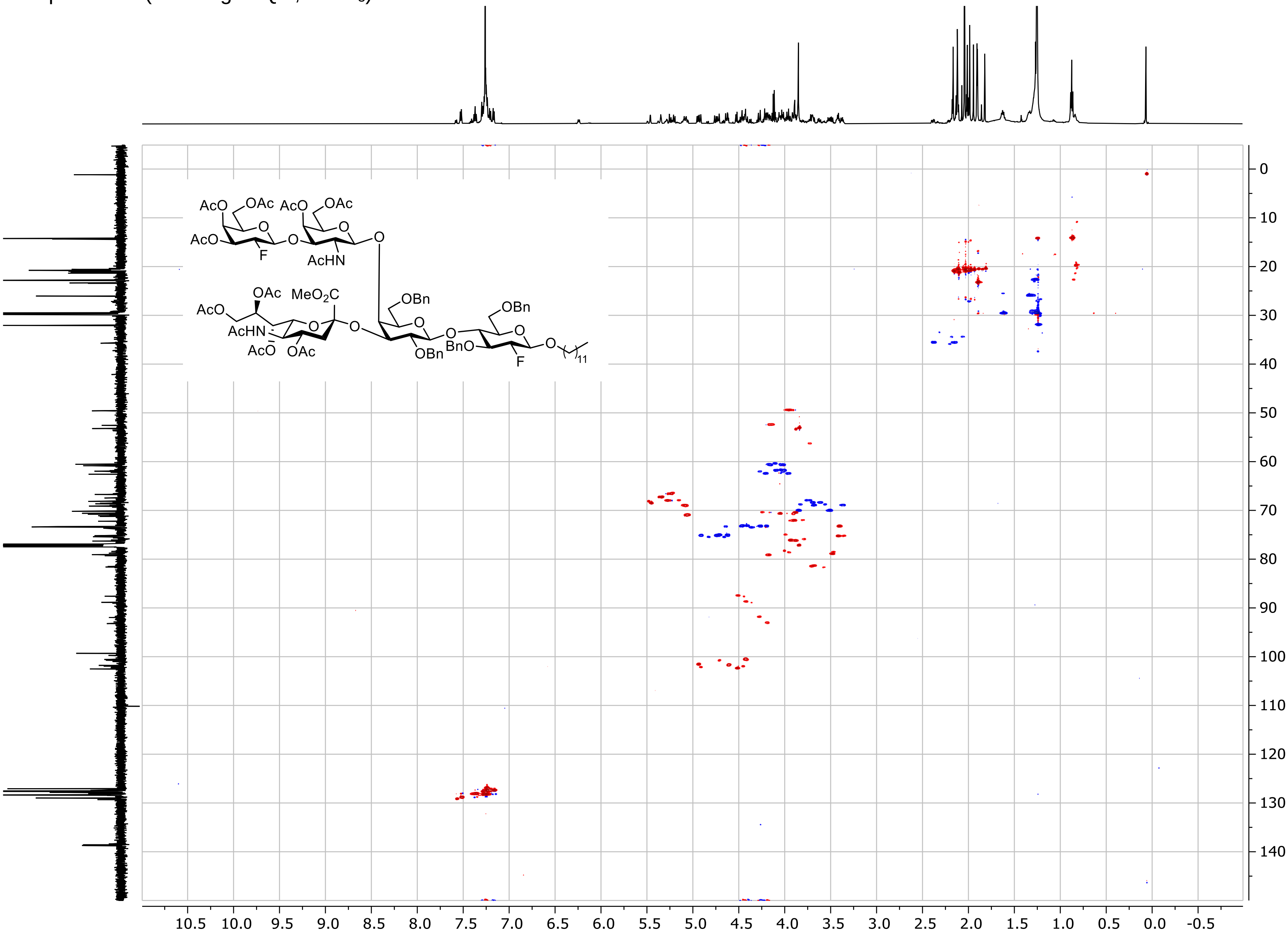

Compound **22** (^{1}H-^{13}C-gHMBC, $CDCl_3$)

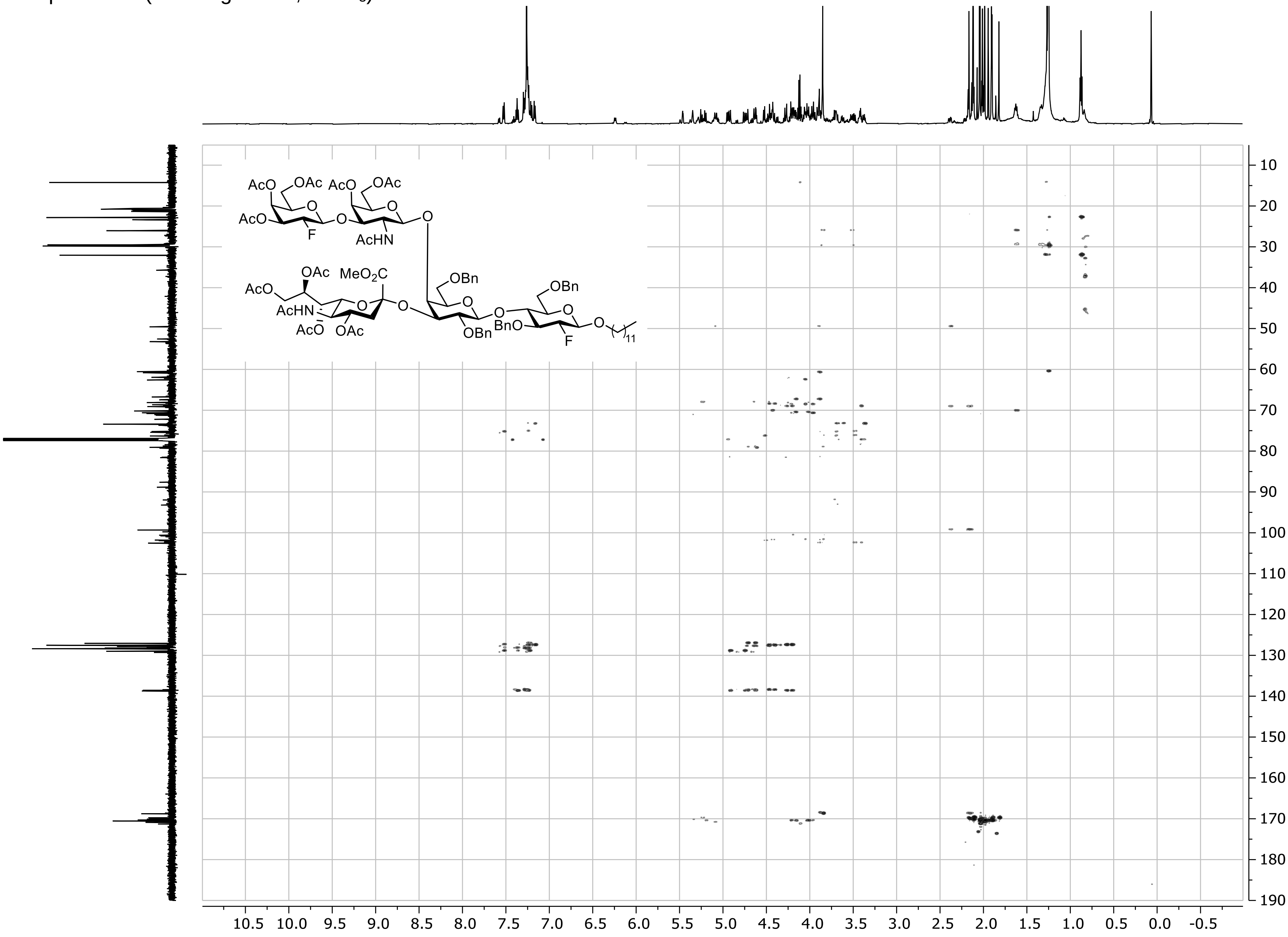

Compound **23** (^{1}H NMR, 599 MHz, $CDCl_3$)

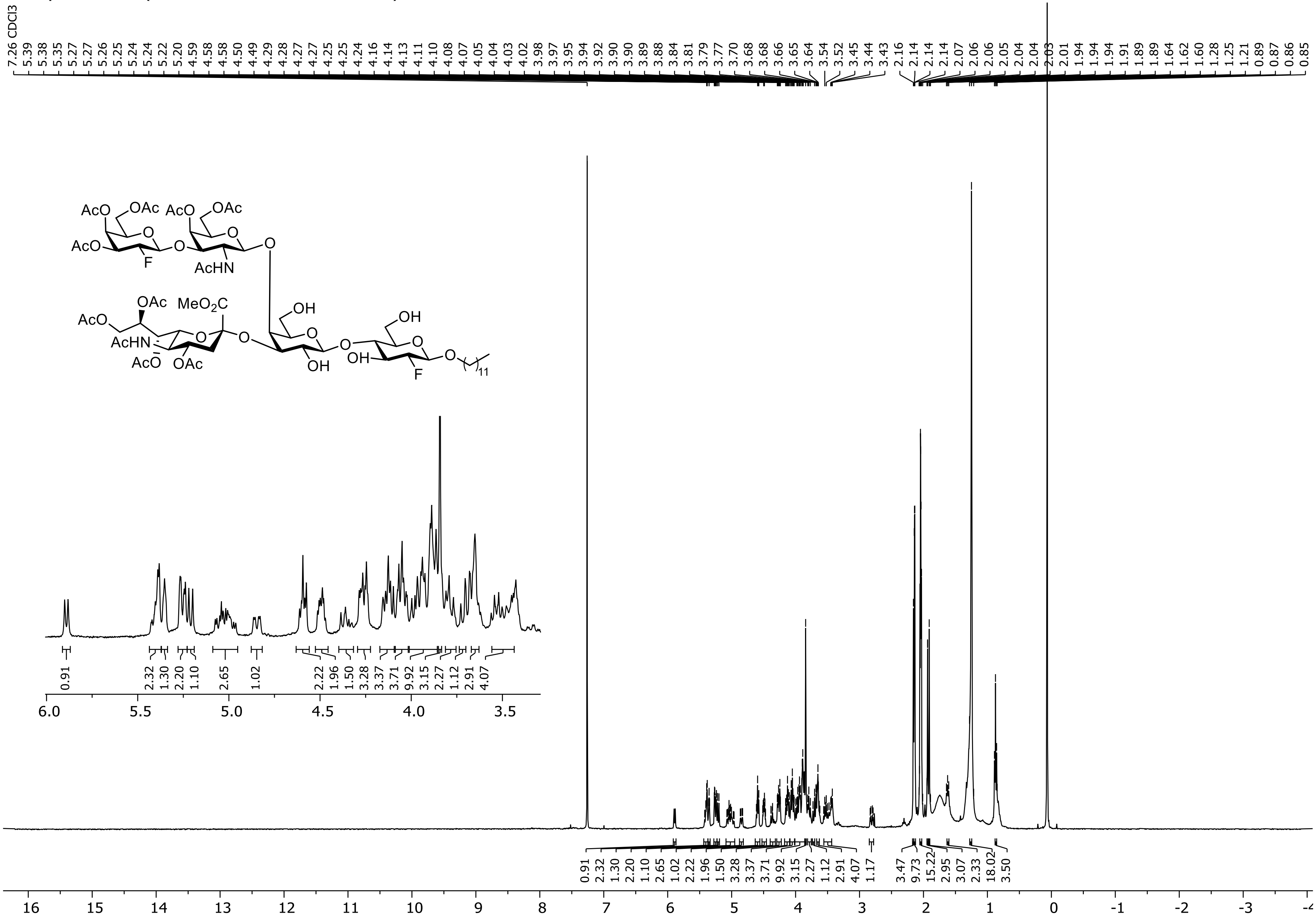

Compound **23** (^{13}C NMR, 151 MHz, $CDCl_3$)

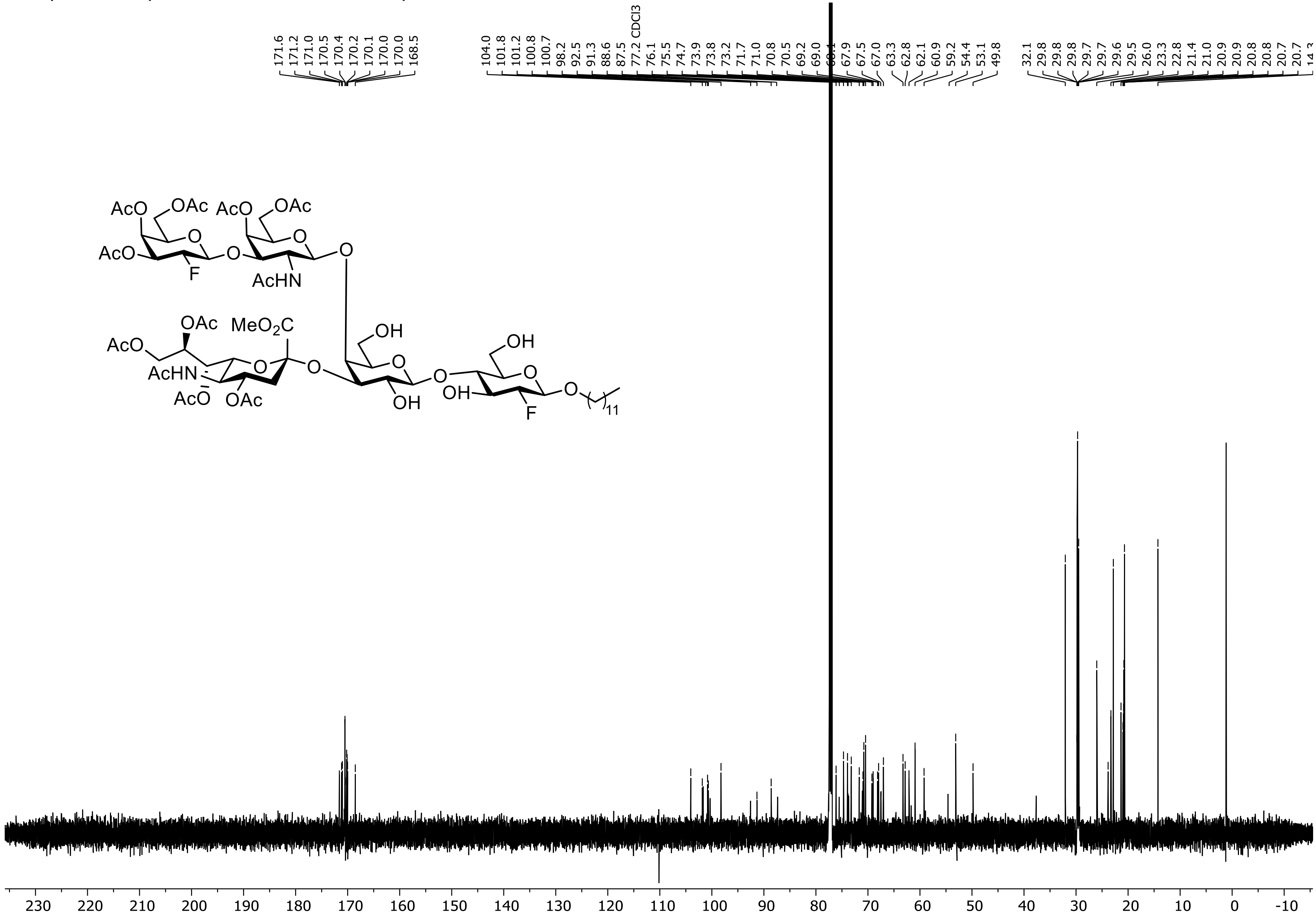

Compound **23** ($^{19}F\{^1H\}$ NMR, 564 MHz, $CDCl_3$) and (1) $^{19}F\{^1H\}$ and (2) ^{19}F NMR (564 MHz)

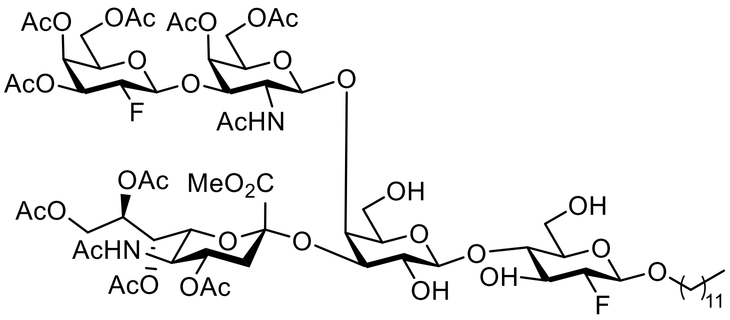

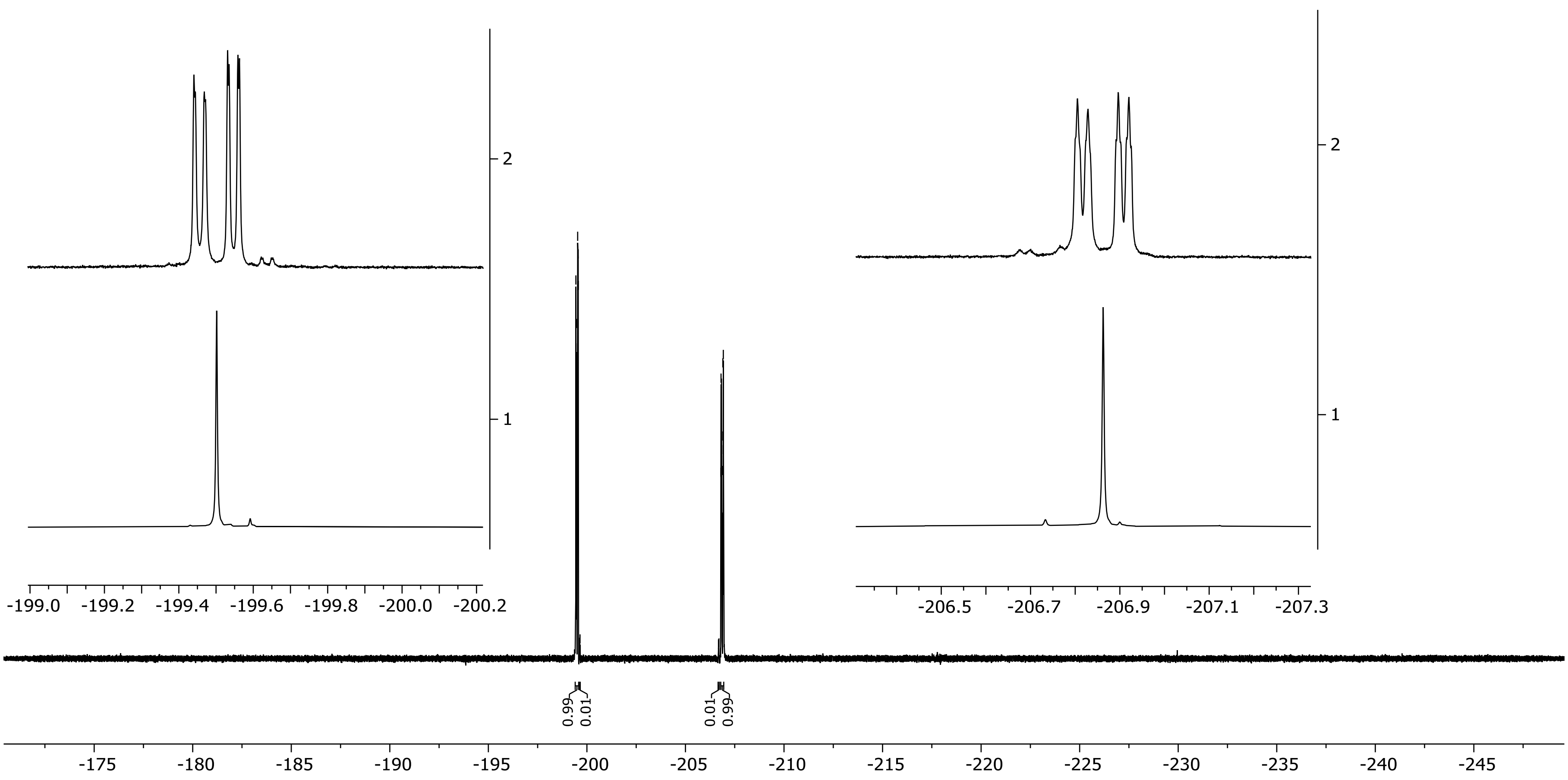

Compound **23** (^{1}H-^{1}H-COSY, $CDCl_3$)

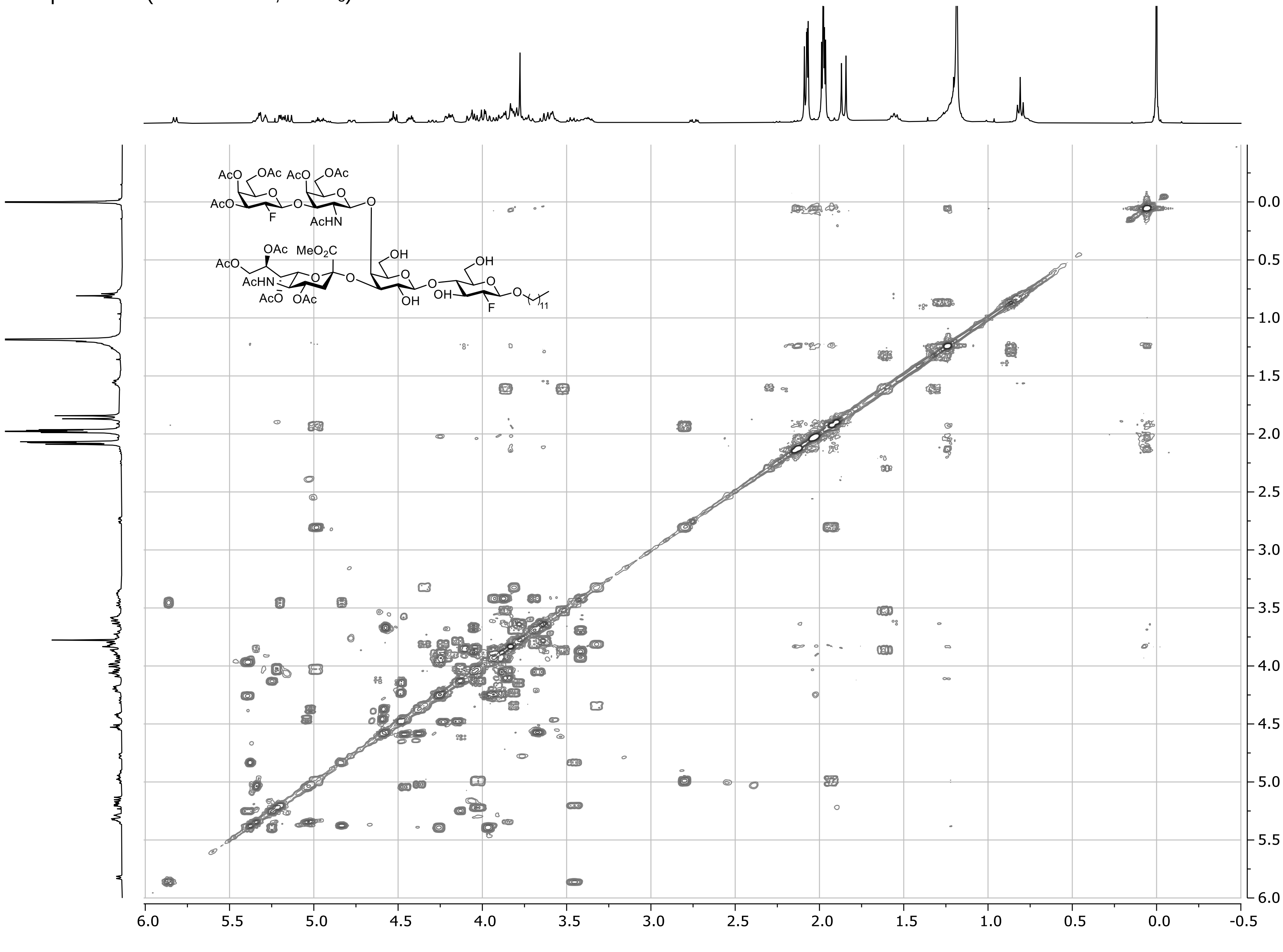

Compound **23** (^{1}H-^{13}C-gHSQC, $CDCl_3$)

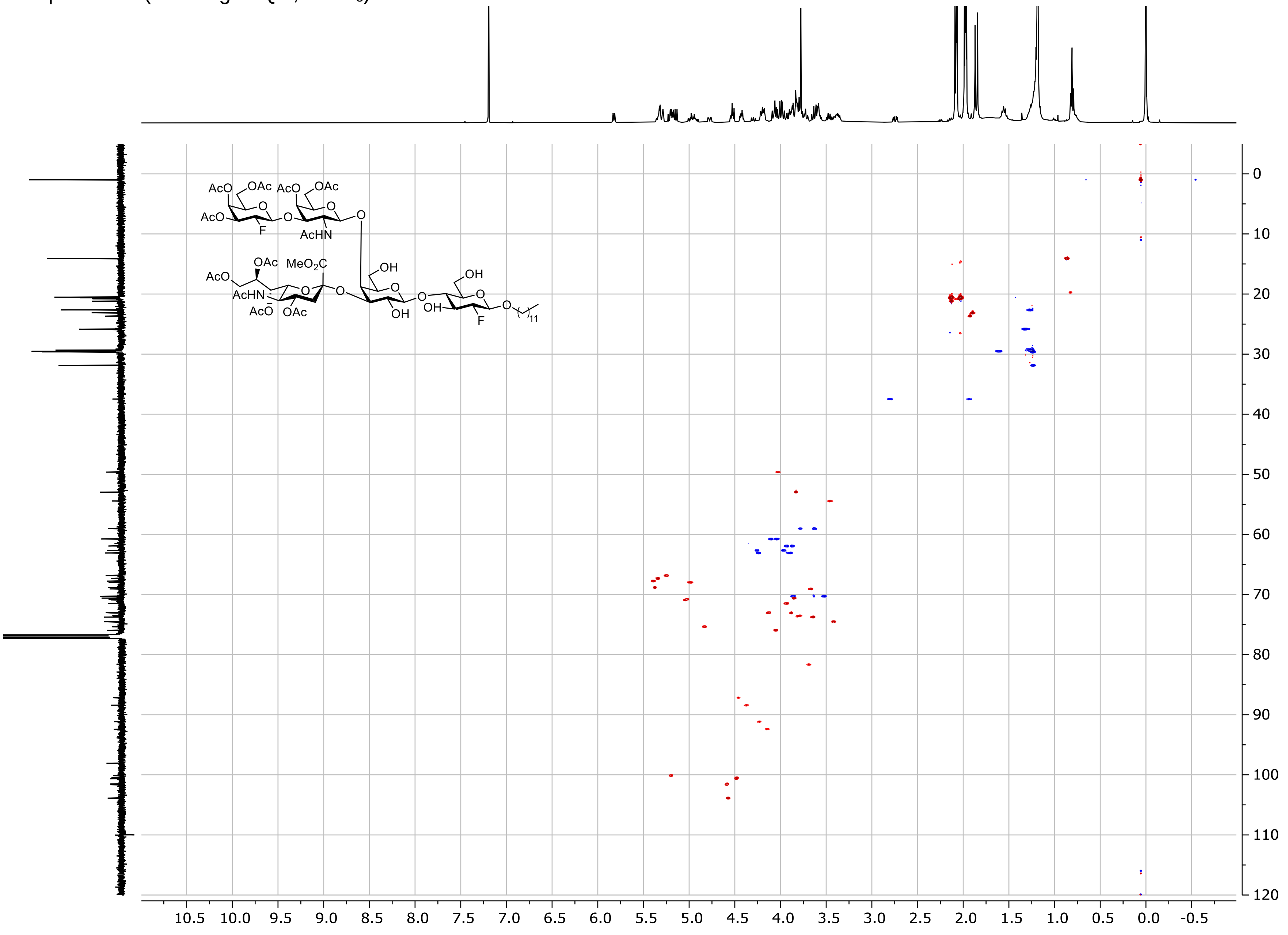

Compound **23** (^{1}H-^{13}C-gHMBC, $CDCl_3$)

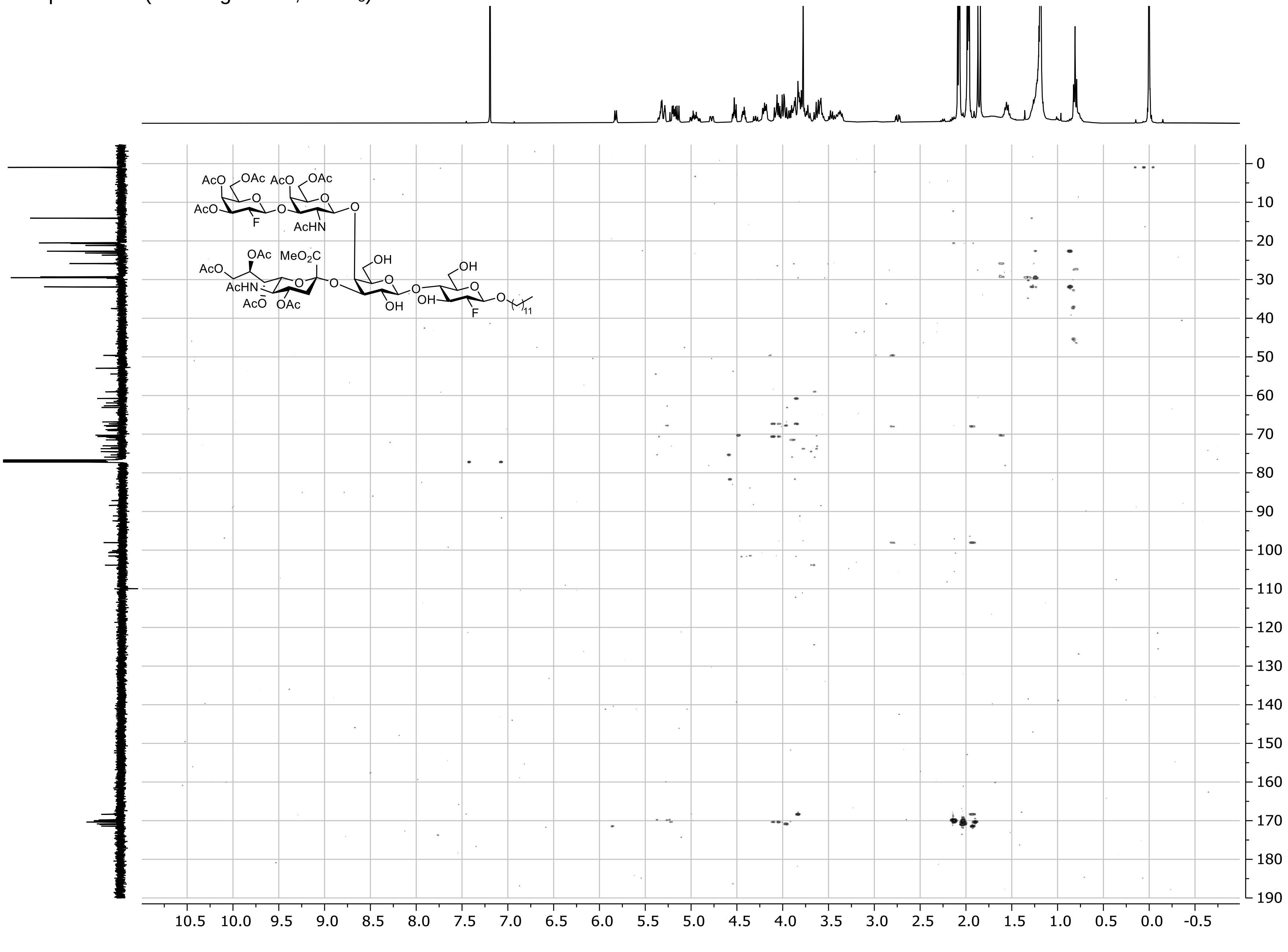

Compound **1** (^{1}H NMR, 599 MHz, CD_3OD)

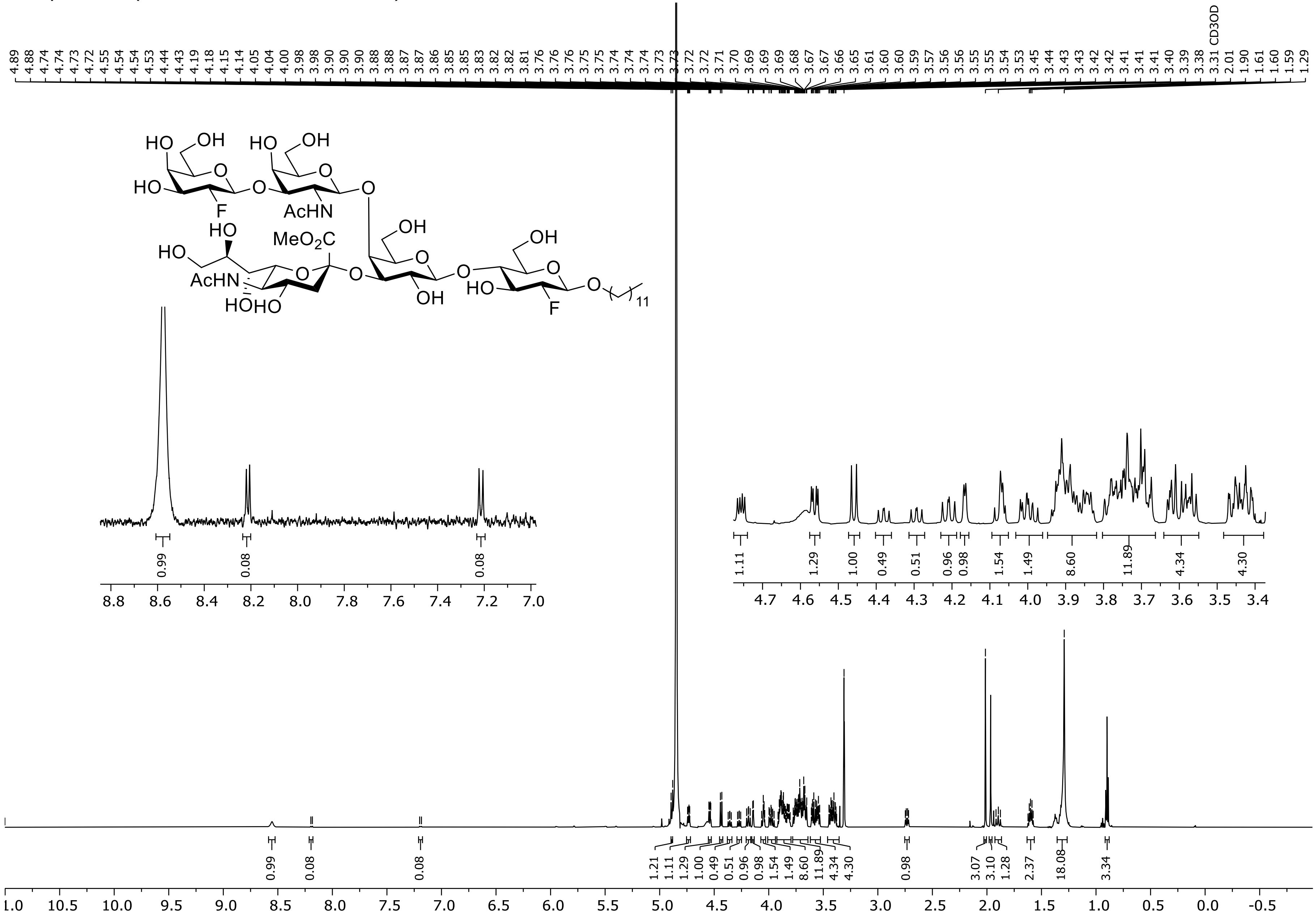

Compound **1** (^{13}C NMR, 151 MHz, CD_3OD)

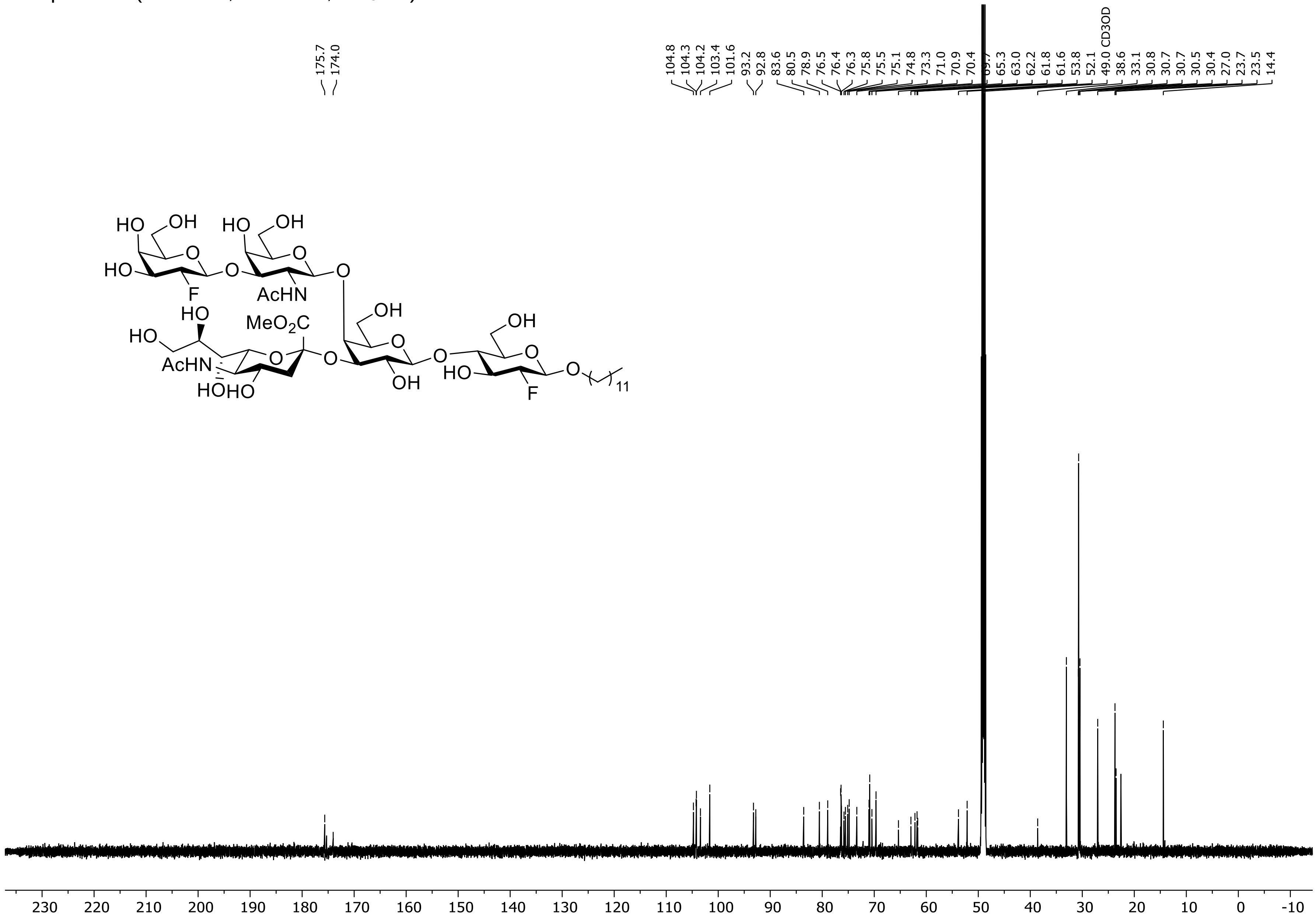

Compound **1** ($^{19}F\{^{1}H\}$ NMR, 564 MHz, CD_3OD) and (1) $^{19}F\{^{1}H\}$ and (2) ^{19}F NMR (564 MHz)

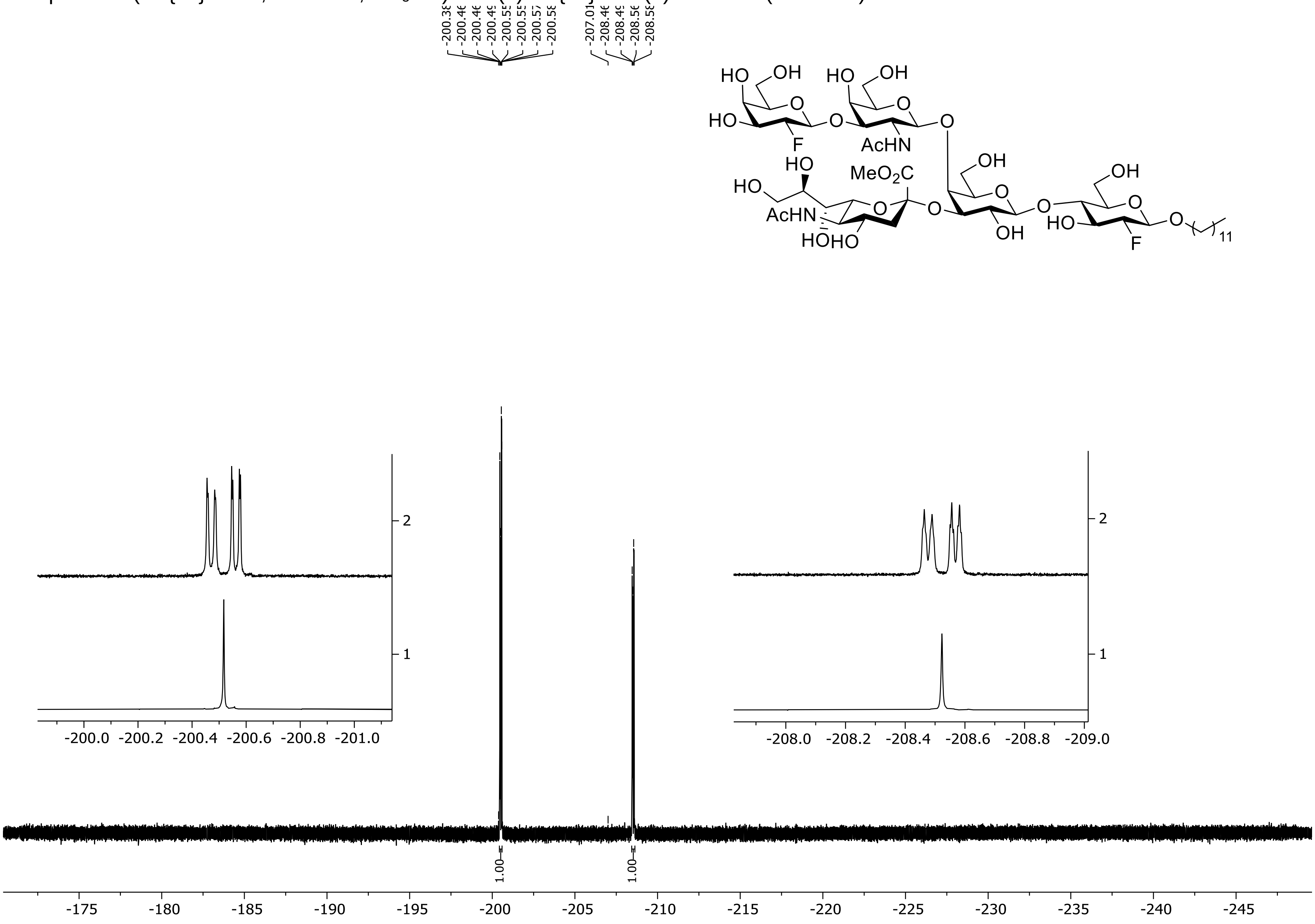

Compound **1** (^{1}H-^{1}H-COSY, CD_3OD)

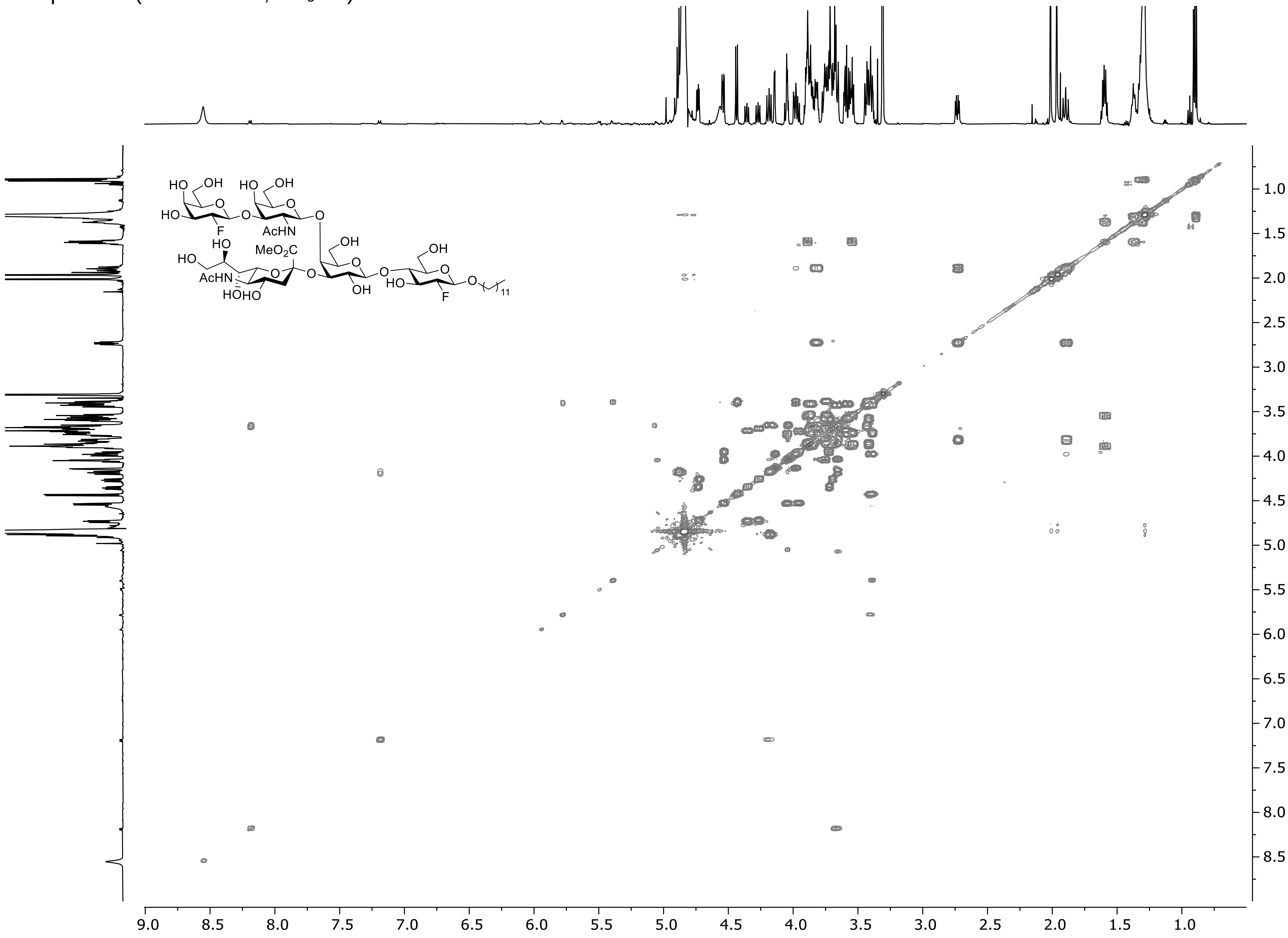

Compound **1** (^{1}H-^{13}C gHSQC, CD_3OD)

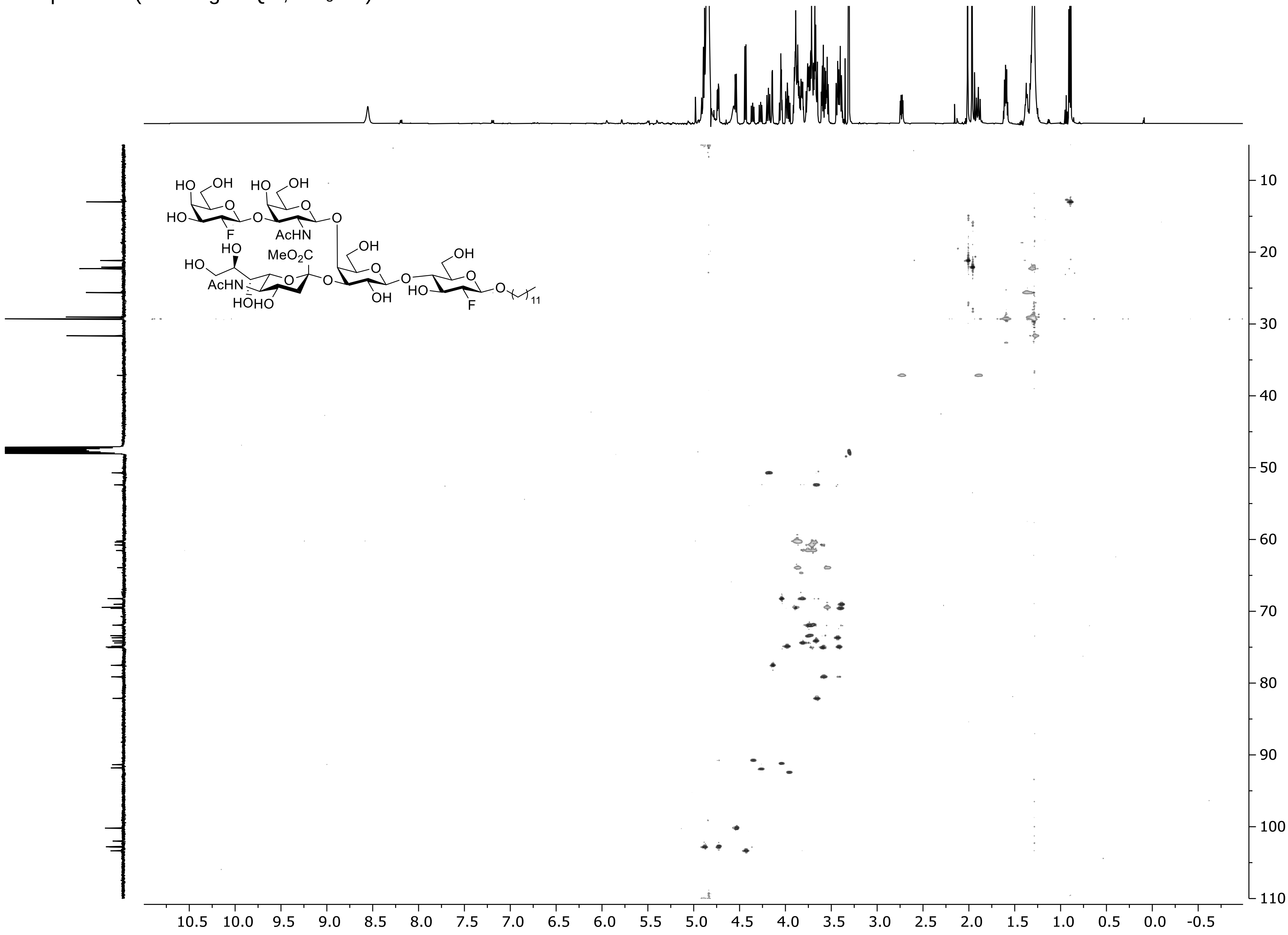

Compound **1** (^{1}H-^{13}C gHMBC, CD_3OD)

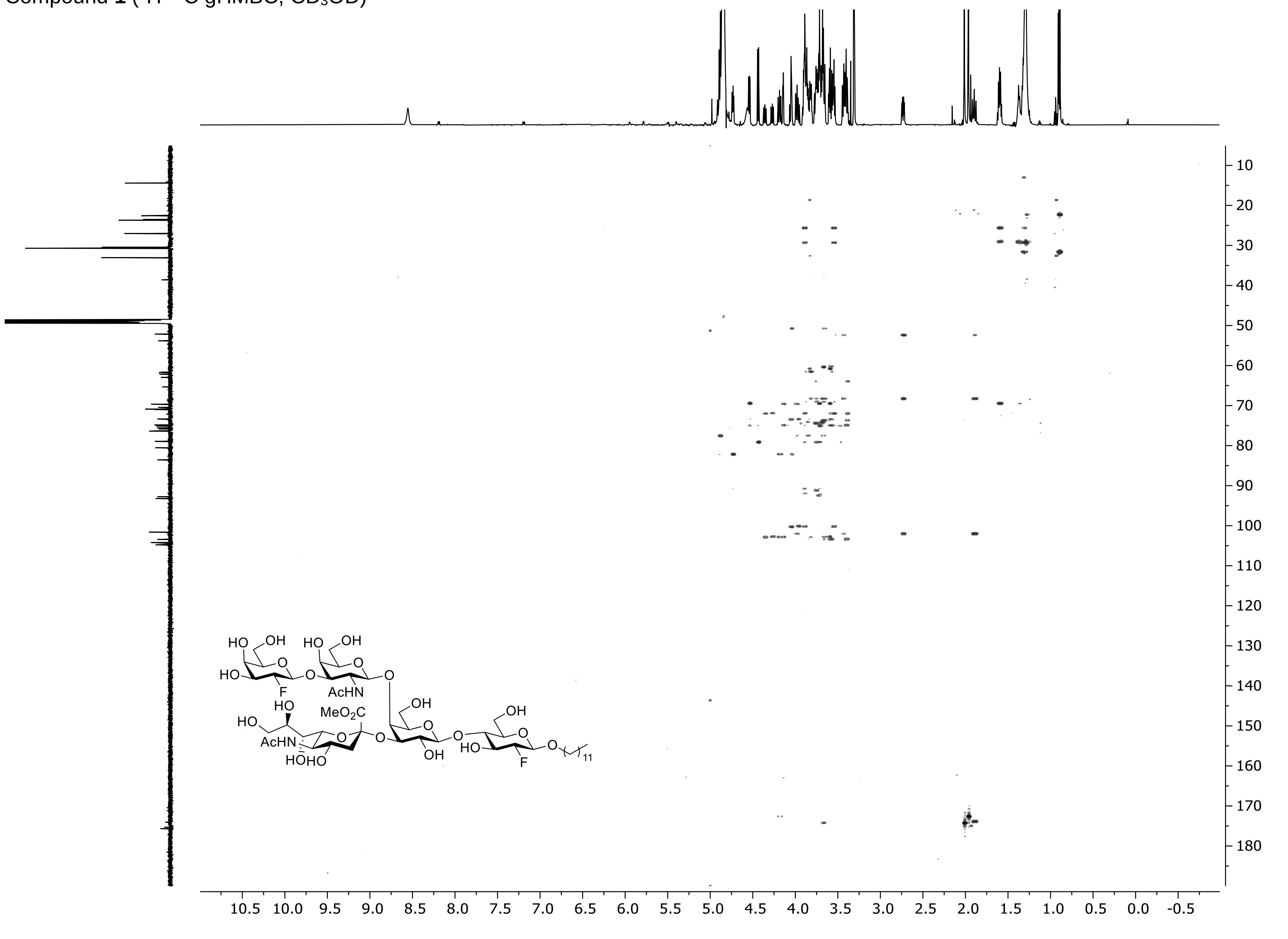

Supplement: Supplementary file 1 — oc4c00622_si_001.pdf [file oc4c00622_si_001.pdf]
