# Supplementary material for: Probing the Origin of Affinity in the GM1-Cholera Toxin Complex through Site-Selective Editing with Fluorine
Source: ACS Cent Sci. 2024 Jul 12;10(8):1481–9. doi: 10.1021/acscentsci.4c00622 (PMC11363330; doi:10.1021/acscentsci.4c00622)
Supplement: Supplementary file 2 — oc4c00622_si_002.pdf [file oc4c00622_si_002.pdf]

Full wwPDB X-ray Structure Validation Report (i)

Apr 16, 2024 – 01:18 pm BST

PDB ID	:	9EWF
Title	:	Cholera toxin B subunit in complex with fluorinated GM1
Deposited on	:	2024-04-03
Resolution	:	2.10 Å(reported)

This wwPDB validation report is for manuscript review

This is a Full wwPDB X-ray Structure Validation Report.

This report is produced by the wwPDB biocuration pipeline after annotation of the structure.

We welcome your comments at *validation@mail.wwpdb.org*
A user guide is available at
https://www.wwpdb.org/validation/2017/XrayValidationReportHelp
with specific help available everywhere you see the (i) symbol.

The types of validation reports are described at
http://www.wwpdb.org/validation/2017/FAQs#types.

For Manuscript Review

The following versions of software and data (see references (i)) were used in the production of this report:

MolProbity	:	4.02b-467
Mogul	:	1.8.4, CSD as541be (2020)
Xtriage (Phenix)	:	1.13
EDS	:	2.36
buster-report	:	1.1.7 (2018)
Percentile statistics	:	20191225.v01 (using entries in the PDB archive December 25th 2019)
Refmac	:	5.8.0158
CCP4	:	7.0.044 (Gargrove)
Ideal geometry (proteins)	:	Engh & Huber (2001)
Ideal geometry (DNA, RNA)	:	Parkinson et al. (1996)

1 Overall quality at a glance (i)

The following experimental techniques were used to determine the structure:
X-RAY DIFFRACTION

The reported resolution of this entry is 2.10 Å.

Percentile scores (ranging between 0-100) for global validation metrics of the entry are shown in the following graphic. The table shows the number of entries on which the scores are based.

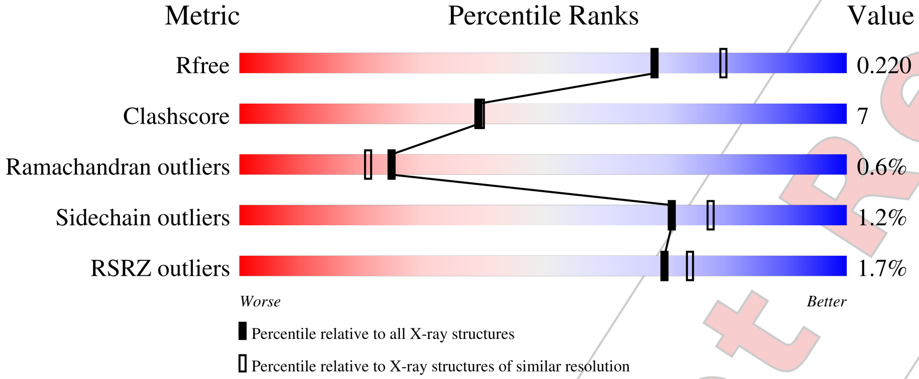

Metric	Whole archive (#Entries)	Similar resolution (#Entries, resolution range(Å))
R_{free}	130704	5197 (2.10-2.10)
Clashscore	141614	5710 (2.10-2.10)
Ramachandran outliers	138981	5647 (2.10-2.10)
Sidechain outliers	138945	5648 (2.10-2.10)
RSRZ outliers	127900	5083 (2.10-2.10)

The table below summarises the geometric issues observed across the polymeric chains and their fit to the electron density. The red, orange, yellow and green segments of the lower bar indicate the fraction of residues that contain outliers for >=3, 2, 1 and 0 types of geometric quality criteria respectively. A grey segment represents the fraction of residues that are not modelled. The numeric value for each fraction is indicated below the corresponding segment, with a dot representing fractions <=5% The upper red bar (where present) indicates the fraction of residues that have poor fit to the electron density. The numeric value is given above the bar.

Mol	Chain	Length	Quality of chain
1	A	103	4%<br>87% 11% •
1	B	103	%<br>91% 8% •
1	C	103	6%<br>90% 7% •
1	D	103	3%<br>81% 17% •

Continued on next page...

For Manuscript Review

Continued from previous page...

Mol	Chain	Length	Quality of chain
1	E	103	87% 12% •
1	F	103	83% 17% •
1	G	103	2% 88% 12%
1	H	103	% 90% 9% •
1	I	103	90% 9% •
1	J	103	% 84% 16%
2	K	4	75% 25%
2	L	4	50% 50%

The following table lists non-polymeric compounds, carbohydrate monomers and non-standard residues in protein, DNA, RNA chains that are outliers for geometric or electron-density-fit criteria:

Mol	Type	Chain	Res	Chirality	Geometry	Clashes	Electron density
2	NGA	K	2	-	-	-	X
2	2FG	K	3	-	-	-	X
2	NGA	L	2	-	-	-	X
2	2FG	L	3	-	-	-	X
3	A1H7V	G	201	-	-	-	X
3	A1H7V	I	201	-	-	-	X

For Manuscript Review

2 Entry composition (i)

There are 4 unique types of molecules in this entry. The entry contains 17405 atoms, of which 8296 are hydrogens and 0 are deuteriums.

In the tables below, the ZeroOcc column contains the number of atoms modelled with zero occupancy, the AltConf column contains the number of residues with at least one atom in alternate conformation and the Trace column contains the number of residues modelled with at most 2 atoms.

- Molecule 1 is a protein called Cholera enterotoxin subunit B.

Mol	Chain	Residues	Atoms						ZeroOcc	AltConf	Trace
1	A	103	Total 1631	C 511	H 817	N 142	O 156	S 5	0	0	0
1	B	103	Total 1631	C 511	H 817	N 142	O 156	S 5	0	0	0
1	C	103	Total 1631	C 511	H 817	N 142	O 156	S 5	0	0	0
1	I	103	Total 1631	C 511	H 817	N 142	O 156	S 5	0	0	0
1	J	103	Total 1631	C 511	H 817	N 142	O 156	S 5	0	0	0
1	D	103	Total 1631	C 511	H 817	N 142	O 156	S 5	2	0	0
1	E	103	Total 1631	C 511	H 817	N 142	O 156	S 5	0	0	0
1	F	103	Total 1631	C 511	H 817	N 142	O 156	S 5	0	0	0
1	G	103	Total 1631	C 511	H 817	N 142	O 156	S 5	0	0	0
1	H	103	Total 1631	C 511	H 817	N 142	O 156	S 5	0	0	0

There are 20 discrepancies between the modelled and reference sequences:

Chain	Residue	Modelled	Actual	Comment	Reference
A	18	HIS	TYR	conflict	UNP P01556
A	47	THR	ILE	conflict	UNP P01556
B	18	HIS	TYR	conflict	UNP P01556
B	47	THR	ILE	conflict	UNP P01556
C	18	HIS	TYR	conflict	UNP P01556
C	47	THR	ILE	conflict	UNP P01556
I	18	HIS	TYR	conflict	UNP P01556
I	47	THR	ILE	conflict	UNP P01556
J	18	HIS	TYR	conflict	UNP P01556

Continued on next page...

For Manuscript Review

Continued from previous page...

Chain	Residue	Modelled	Actual	Comment	Reference
J	47	THR	ILE	conflict	UNP P01556
D	18	HIS	TYR	conflict	UNP P01556
D	47	THR	ILE	conflict	UNP P01556
E	18	HIS	TYR	conflict	UNP P01556
E	47	THR	ILE	conflict	UNP P01556
F	18	HIS	TYR	conflict	UNP P01556
F	47	THR	ILE	conflict	UNP P01556
G	18	HIS	TYR	conflict	UNP P01556
G	47	THR	ILE	conflict	UNP P01556
H	18	HIS	TYR	conflict	UNP P01556
H	47	THR	ILE	conflict	UNP P01556

- Molecule 2 is an oligosaccharide called 2-deoxy-2-fluoro-beta-D-galactopyranose-(1-3)-2-ace tamido-2-deoxy-beta-D-galactopyranose-(1-4)-[N-acetyl-alpha-neuraminic acid-(2-3)]beta-D -galactopyranose.

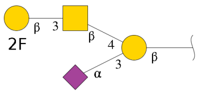

Mol	Chain	Residues	Atoms	ZeroOcc	AltConf	Trace
2	K	4	Total C F H N O<br>105 31 1 49 2 22	0	0	0
2	L	4	Total C F H N O<br>105 31 1 49 2 22	0	0	0

- Molecule 3 is (2R,3S,4S,5R,6R)-6-dodecoxy-5-fluoranyl-2-(hydroxymethyl)oxane-3,4-diol (three-letter code: A1H7V) (formula: $C_{18}H_{35}FO_5$) (labeled as "Ligand of Interest" by depositor).

For Manuscript Review

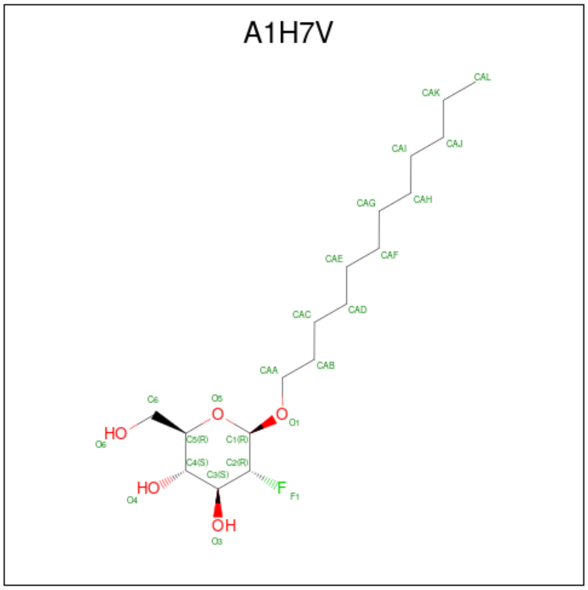

Mol	Chain	Residues	Atoms	ZeroOcc	AltConf
3	I	1	Total C F H O<br>28 8 1 14 5	0	0
3	G	1	Total C F H O<br>28 8 1 14 5	0	0

- Molecule 4 is water.

Mol	Chain	Residues	Atoms	ZeroOcc	AltConf
4	A	76	Total O<br>76 76	0	0
4	B	91	Total O<br>91 91	0	0
4	C	67	Total O<br>67 67	0	0
4	I	80	Total O<br>80 80	0	0
4	J	83	Total O<br>83 83	0	0
4	D	85	Total O<br>85 85	0	0
4	E	89	Total O<br>89 89	0	0
4	F	85	Total O<br>85 85	0	0
4	G	72	Total O<br>72 72	0	0
4	H	101	Total O<br>101 101	0	0

For Manuscript Review

3 Residue-property plots (i)

These plots are drawn for all protein, RNA, DNA and oligosaccharide chains in the entry. The first graphic for a chain summarises the proportions of the various outlier classes displayed in the second graphic. The second graphic shows the sequence view annotated by issues in geometry and electron density. Residues are color-coded according to the number of geometric quality criteria for which they contain at least one outlier: green = 0, yellow = 1, orange = 2 and red = 3 or more. A red dot above a residue indicates a poor fit to the electron density (RSRZ > 2). Stretches of 2 or more consecutive residues without any outlier are shown as a green connector. Residues present in the sample, but not in the model, are shown in grey.

• Molecule 1: Cholera enterotoxin subunit B

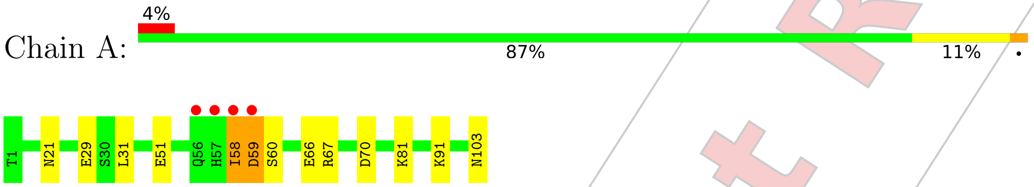

• Molecule 1: Cholera enterotoxin subunit B

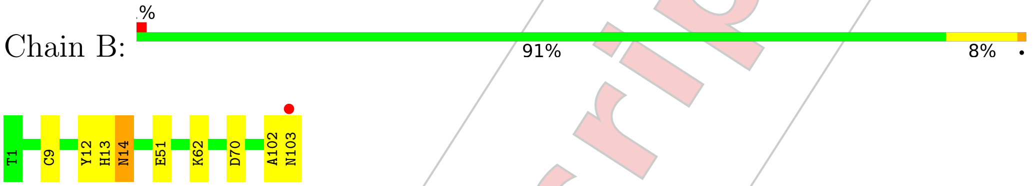

• Molecule 1: Cholera enterotoxin subunit B

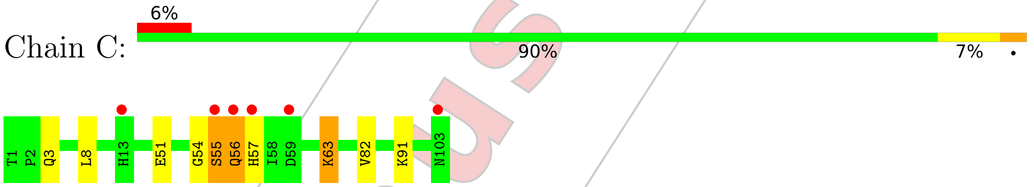

• Molecule 1: Cholera enterotoxin subunit B

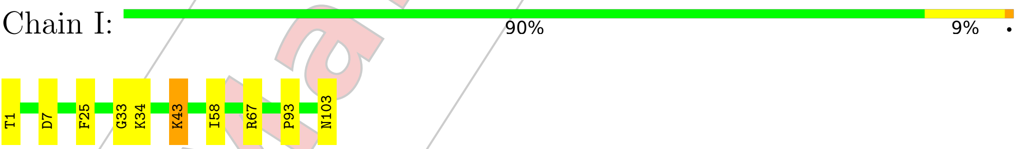

• Molecule 1: Cholera enterotoxin subunit B

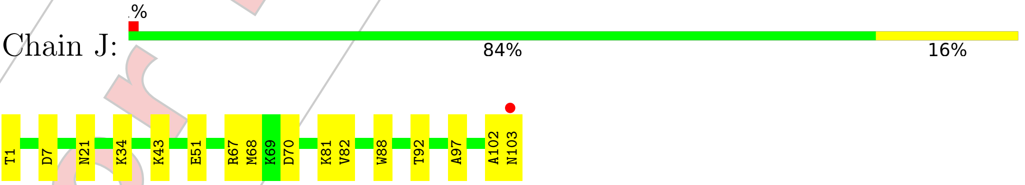

• Molecule 1: Cholera enterotoxin subunit B

For Manuscript Review

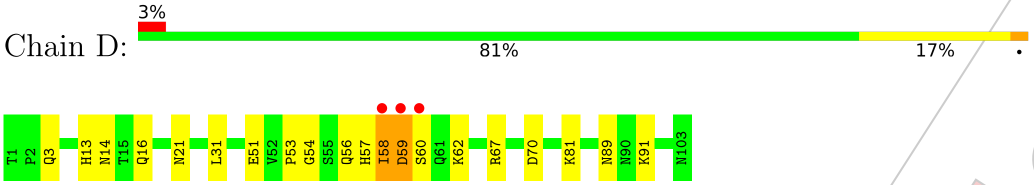

• Molecule 1: Cholera enterotoxin subunit B

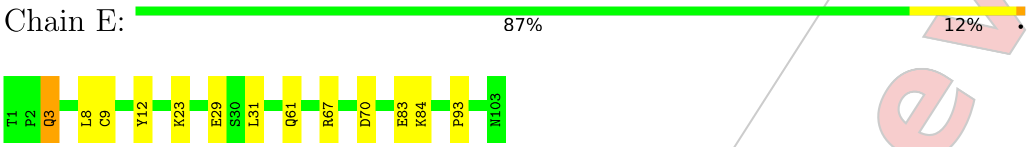

• Molecule 1: Cholera enterotoxin subunit B

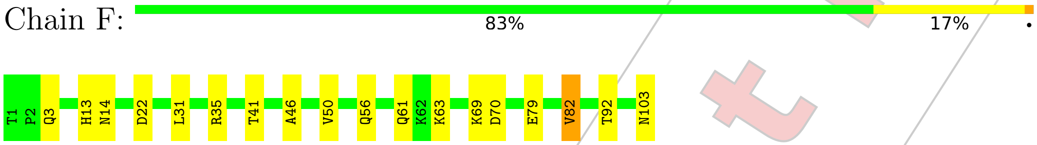

• Molecule 1: Cholera enterotoxin subunit B

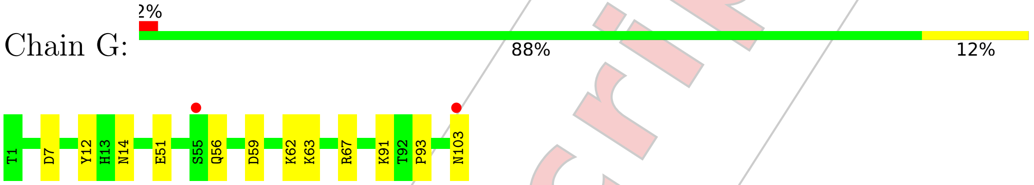

• Molecule 1: Cholera enterotoxin subunit B

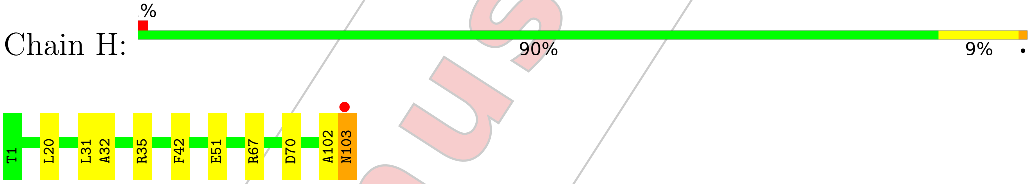

• Molecule 2: 2-deoxy-2-fluoro-beta-D-galactopyranose-(1-3)-2-acetamido-2-deoxy-beta-D-galacto pyranose-(1-4)-[N-acetyl-alpha-neuraminic acid-(2-3)]beta-D-galactopyranose

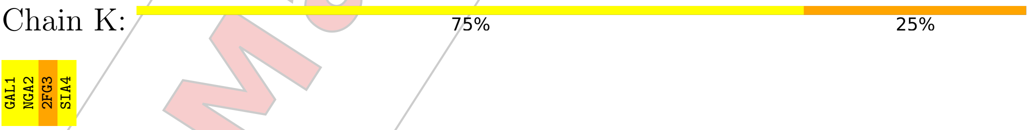

• Molecule 2: 2-deoxy-2-fluoro-beta-D-galactopyranose-(1-3)-2-acetamido-2-deoxy-beta-D-galacto pyranose-(1-4)-[N-acetyl-alpha-neuraminic acid-(2-3)]beta-D-galactopyranose

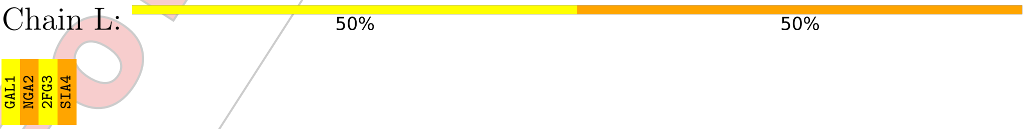

For Manuscript Review

4 Data and refinement statistics (i)

Property	Value	Source
Space group	P 4 21 2	Depositor
Cell constants<br>a, b, c, α, β, γ	132.52Å 132.52Å 113.85Å<br>90.00° 90.00° 90.00°	Depositor
Resolution (Å)	46.85 – 2.10<br>46.85 – 2.10	Depositor<br>EDS
% Data completeness<br>(in resolution range)	99.9 (46.85-2.10)<br>89.0 (46.85-2.10)	Depositor<br>EDS
R_{merge}	0.20	Depositor
R_{sym}	(Not available)	Depositor
$< I/\sigma(I) >$ [1]	1.29 (at 2.10Å)	Xtriage
Refinement program	PHENIX 1.21_5190	Depositor
R, R_{free}	0.183 , 0.221<br>0.184 , 0.220	Depositor<br>DCC
R_{free} test set	3000 reflections (5.04%)	wwPDB-VP
Wilson B-factor (Å^2)	24.0	Xtriage
Anisotropy	0.143	Xtriage
Bulk solvent k_{sol}(e/Å^3), B_{sol}(Å^2)	0.39 , 56.3	EDS
L-test for twinning[2]	$< \|L\| >$ = 0.49, $< L^2 >$ = 0.32	Xtriage
Estimated twinning fraction	No twinning to report.	Xtriage
F_o,F_c correlation	0.95	EDS
Total number of atoms	17405	wwPDB-VP
Average B, all atoms (Å^2)	38.0	wwPDB-VP

Xtriage's analysis on translational NCS is as follows: *The analyses of the Patterson function reveals a significant off-origin peak that is 41.08 % of the origin peak, indicating pseudo-translational symmetry. The chance of finding a peak of this or larger height randomly in a structure without pseudo-translational symmetry is equal to 2.4899e-04. The detected translational NCS is most likely also responsible for the elevated intensity ratio.*

[1]Intensities estimated from amplitudes.

[2]Theoretical values of $< |L| >$, $< L^2 >$ for acentric reflections are 0.5, 0.333 respectively for untwinned datasets, and 0.375, 0.2 for perfectly twinned datasets.

For Manuscript Review

5 Model quality (i)

5.1 Standard geometry (i)

Bond lengths and bond angles in the following residue types are not validated in this section: NGA, GAL, A1H7V, 2FG, SIA

The Z score for a bond length (or angle) is the number of standard deviations the observed value is removed from the expected value. A bond length (or angle) with $|Z| > 5$ is considered an outlier worth inspection. RMSZ is the root-mean-square of all Z scores of the bond lengths (or angles).

Mol	Chain	Bond lengths		Bond angles	
		RMSZ	#$|Z|$ >5	RMSZ	#$|Z|$ >5
1	A	0.31	0/828	0.50	0/1118
1	B	0.31	0/828	0.53	0/1118
1	C	0.32	0/828	0.50	0/1118
1	D	0.31	0/828	0.51	0/1118
1	E	0.29	0/828	0.49	0/1118
1	F	0.30	0/828	0.50	0/1118
1	G	0.30	0/828	0.50	0/1118
1	H	0.31	0/828	0.52	0/1118
1	I	0.29	0/828	0.50	0/1118
1	J	0.30	0/828	0.52	0/1118
All	All	0.31	0/8280	0.51	0/11180

There are no bond length outliers.

There are no bond angle outliers.

There are no chirality outliers.

There are no planarity outliers.

5.2 Too-close contacts (i)

In the following table, the Non-H and H(model) columns list the number of non-hydrogen atoms and hydrogen atoms in the chain respectively. The H(added) column lists the number of hydrogen atoms added and optimized by MolProbity. The Clashes column lists the number of clashes within the asymmetric unit, whereas Symm-Clashes lists symmetry-related clashes.

Mol	Chain	Non-H	H(model)	H(added)	Clashes	Symm-Clashes
1	A	814	817	817	15	0
1	B	814	817	817	12	0
1	C	814	817	817	7	0

Continued on next page...

For Manuscript Review

Continued from previous page...

Mol	Chain	Non-H	H(model)	H(added)	Clashes	Symm-Clashes
1	D	814	817	817	22	0
1	E	814	817	817	12	0
1	F	814	817	817	15	0
1	G	814	817	817	14	0
1	H	814	817	817	15	0
1	I	814	817	817	9	0
1	J	814	817	817	15	0
2	K	56	49	46	1	0
2	L	56	49	46	3	0
3	G	14	14	0	2	0
3	I	14	14	0	2	0
4	A	76	0	0	5	0
4	B	91	0	0	11	1
4	C	67	0	0	2	0
4	D	85	0	0	6	2
4	E	89	0	0	2	1
4	F	85	0	0	4	0
4	G	72	0	0	9	2
4	H	101	0	0	5	1
4	I	80	0	0	5	2
4	J	83	0	0	5	1
All	All	9109	8296	8262	122	5

The all-atom clashscore is defined as the number of clashes found per 1000 atoms (including hydrogen atoms). The all-atom clashscore for this structure is 7.

All (122) close contacts within the same asymmetric unit are listed below, sorted by their clash magnitude.

Atom-1	Atom-2	Interatomic distance (Å)	Clash overlap (Å)
1:H:51:GLU:OE1	4:H:201:HOH:O	1.58	1.17
1:J:7:ASP:OD1	4:J:201:HOH:O	1.77	1.02
1:D:51:GLU:OE1	4:D:201:HOH:O	1.77	1.01
3:I:201:A1H7V:O1	4:I:301:HOH:O	1.80	0.99
1:G:62:LYS:NZ	4:G:301:HOH:O	1.94	0.97
1:B:103:ASN:OXT	4:B:201:HOH:O	1.89	0.89
1:D:62:LYS:HD2	4:D:215:HOH:O	1.70	0.88
1:I:7:ASP:OD2	4:I:302:HOH:O	1.93	0.86
1:F:103:ASN:O	4:F:201:HOH:O	1.94	0.85
1:D:62:LYS:HE3	4:D:204:HOH:O	1.76	0.85
1:B:12:TYR:O	4:B:202:HOH:O	1.92	0.84
1:C:56:GLN:NE2	4:C:4301:HOH:O	1.84	0.81

Continued on next page...

For Manuscript Review

Continued from previous page...

Atom-1	Atom-2	Interatomic distance (Å)	Clash overlap (Å)
3:I:201:A1H7V:CAA	4:I:301:HOH:O	2.25	0.81
1:G:59:ASP:O	4:G:302:HOH:O	1.97	0.81
1:B:103:ASN:HA	4:B:209:HOH:O	1.81	0.80
1:B:102:ALA:O	4:B:204:HOH:O	2.00	0.79
1:B:51:GLU:OE1	4:B:203:HOH:O	2.00	0.78
1:J:51:GLU:OE2	4:J:202:HOH:O	2.03	0.77
1:G:63:LYS:NZ	4:G:305:HOH:O	2.16	0.76
1:J:21:ASN:HA	1:J:81:LYS:HE2	1.68	0.76
1:H:103:ASN:O	4:H:202:HOH:O	2.04	0.76
1:B:103:ASN:HB3	4:B:269:HOH:O	1.86	0.75
1:G:51:GLU:OE1	4:G:303:HOH:O	2.07	0.72
1:A:103:ASN:OXT	4:A:201:HOH:O	2.08	0.71
1:B:103:ASN:C	4:B:209:HOH:O	2.30	0.69
4:I:358:HOH:O	2:K:3:2FG:F2	2.00	0.69
1:A:58:ILE:HG23	1:A:59:ASP:N	2.11	0.66
1:A:66:GLU:OE1	4:A:202:HOH:O	2.14	0.65
1:H:103:ASN:O	4:H:203:HOH:O	2.13	0.65
1:F:3:GLN:HG3	1:G:93:PRO:HG3	1.79	0.65
1:G:56:GLN:OE1	1:G:56:GLN:N	2.25	0.65
1:B:103:ASN:CA	4:B:209:HOH:O	2.38	0.65
1:H:103:ASN:ND2	1:H:103:ASN:OXT	2.31	0.64
1:C:54:GLY:O	4:C:4302:HOH:O	2.15	0.64
1:F:14:ASN:OD1	4:F:202:HOH:O	2.14	0.63
1:J:43:LYS:NZ	1:F:79:GLU:OE1	2.34	0.61
1:G:67:ARG:NH1	4:G:304:HOH:O	2.13	0.60
3:G:201:A1H7V:C6	2:L:2:NGA:H82	2.33	0.59
1:A:21:ASN:HA	1:A:81:LYS:HE2	1.86	0.58
1:D:53:PRO:HB3	1:D:62:LYS:HE2	1.86	0.57
1:J:103:ASN:O	4:J:203:HOH:O	2.18	0.57
1:I:43:LYS:N	1:I:43:LYS:HD2	2.20	0.56
1:D:58:ILE:HG23	1:D:59:ASP:N	2.20	0.56
1:D:58:ILE:HG23	1:D:59:ASP:H	1.69	0.56
1:B:103:ASN:N	4:B:207:HOH:O	2.36	0.55
1:A:103:ASN:ND2	4:A:206:HOH:O	2.39	0.54
1:A:103:ASN:HB2	4:B:213:HOH:O	2.07	0.54
1:E:12:TYR:O	4:E:201:HOH:O	2.18	0.54
1:J:1:THR:N	4:J:209:HOH:O	2.41	0.54
1:E:67:ARG:NH1	1:F:70:ASP:OD1	2.37	0.53
1:E:3:GLN:OE1	1:F:92:THR:HG22	2.08	0.53
1:B:13:HIS:O	1:B:14:ASN:OD1	2.26	0.53
1:D:58:ILE:HG13	1:D:59:ASP:H	1.74	0.53

Continued on next page...

For Manuscript Review

Continued from previous page...

Atom-1	Atom-2	Interatomic distance (Å)	Clash overlap (Å)
1:C:51:GLU:OE2	1:C:91:LYS:HE3	2.09	0.52
1:A:58:ILE:HG23	1:A:59:ASP:H	1.73	0.52
4:D:259:HOH:O	1:E:23:LYS:HE2	2.09	0.52
1:J:102:ALA:O	1:J:103:ASN:CB	2.57	0.52
1:A:59:ASP:OD1	1:A:60:SER:N	2.40	0.51
1:A:51:GLU:OE2	1:A:91:LYS:HE2	2.11	0.50
1:D:70:ASP:OD1	1:H:67:ARG:NH1	2.39	0.50
1:H:102:ALA:O	1:H:103:ASN:HB2	2.11	0.50
1:G:12:TYR:CZ	1:H:32:ALA:HB1	2.47	0.50
1:I:25:PHE:HB2	1:I:43:LYS:HD2	1.94	0.49
1:H:102:ALA:O	1:H:103:ASN:CB	2.60	0.49
1:H:20:LEU:HD13	1:H:42:PHE:CZ	2.47	0.49
1:G:103:ASN:O	4:G:307:HOH:O	2.20	0.49
1:E:67:ARG:NH1	4:E:203:HOH:O	2.33	0.49
1:D:56:GLN:OE1	1:D:56:GLN:N	2.44	0.49
1:D:16:GLN:OE1	1:D:89:ASN:HB3	2.12	0.48
1:D:57:HIS:HE1	4:D:201:HOH:O	1.95	0.48
1:F:31:LEU:C	1:F:31:LEU:HD12	2.33	0.48
1:G:67:ARG:NH1	1:H:70:ASP:OD1	2.43	0.48
1:F:13:HIS:CG	4:F:207:HOH:O	2.66	0.48
1:G:7:ASP:OD2	4:G:306:HOH:O	2.19	0.48
1:D:51:GLU:OE2	1:D:91:LYS:HD3	2.13	0.48
1:C:63:LYS:HD2	1:C:63:LYS:C	2.33	0.48
1:A:67:ARG:NH1	1:B:70:ASP:OD1	2.41	0.48
1:D:59:ASP:OD1	1:D:60:SER:N	2.47	0.47
1:B:62:LYS:HE2	4:B:222:HOH:O	2.15	0.47
1:D:57:HIS:CE1	4:D:201:HOH:O	2.67	0.47
3:G:201:A1H7V:C6	2:L:2:NGA:C8	2.93	0.46
1:F:50:VAL:HG11	1:F:69:LYS:HG3	1.97	0.46
1:F:56:GLN:OE1	4:F:203:HOH:O	2.21	0.46
1:D:58:ILE:HG13	1:D:59:ASP:N	2.31	0.46
1:H:103:ASN:C	4:H:202:HOH:O	2.51	0.45
1:D:31:LEU:C	1:D:31:LEU:HD12	2.36	0.45
1:A:70:ASP:OD1	1:J:67:ARG:NH1	2.38	0.45
1:D:54:GLY:H	1:D:57:HIS:HD2	1.65	0.45
1:J:102:ALA:O	1:J:103:ASN:HB2	2.16	0.45
1:J:67:ARG:NH1	4:J:206:HOH:O	2.33	0.45
1:D:13:HIS:O	1:D:14:ASN:HB2	2.18	0.44
1:I:67:ARG:NH1	1:J:70:ASP:OD1	2.45	0.43
1:G:14:ASN:ND2	4:G:309:HOH:O	2.31	0.43
1:D:67:ARG:HG2	1:E:29:GLU:OE2	2.19	0.43

Continued on next page...

For Manuscript Review

Continued from previous page...

Atom-1	Atom-2	Interatomic distance (Å)	Clash overlap (Å)
1:D:21:ASN:OD1	1:D:81:LYS:NZ	2.46	0.43
1:H:31:LEU:C	1:H:31:LEU:HD12	2.39	0.43
1:H:51:GLU:OE2	4:H:204:HOH:O	2.21	0.43
1:I:58:ILE:CD1	1:J:34:LYS:HE2	2.49	0.43
1:A:51:GLU:OE2	4:A:203:HOH:O	2.22	0.42
1:I:43:LYS:NZ	4:I:309:HOH:O	2.42	0.42
1:E:8:LEU:HD13	1:F:35:ARG:HG2	2.01	0.42
1:D:3:GLN:HG2	1:E:93:PRO:HG3	2.02	0.42
1:A:59:ASP:HA	4:A:263:HOH:O	2.20	0.42
1:H:103:ASN:C	1:H:103:ASN:HD22	2.16	0.42
1:E:31:LEU:HD12	1:E:31:LEU:C	2.40	0.42
1:A:31:LEU:HD12	1:A:31:LEU:C	2.40	0.42
1:C:55:SER:C	1:C:57:HIS:H	2.23	0.42
1:C:3:GLN:CG	1:I:93:PRO:HG3	2.50	0.41
1:D:67:ARG:NH1	1:E:70:ASP:OD1	2.47	0.41
1:G:51:GLU:OE2	1:G:91:LYS:HE3	2.21	0.41
1:E:61:GLN:HG3	1:F:31:LEU:O	2.20	0.41
1:F:22:ASP:O	1:F:82:VAL:HG13	2.20	0.41
1:E:83:GLU:HG2	1:E:84:LYS:HG3	2.02	0.41
1:F:41:THR:HA	1:F:46:ALA:O	2.21	0.41
1:H:35:ARG:NH1	2:L:4:SIA:H111	2.36	0.41
1:A:29:GLU:HG3	1:J:68:MET:HE1	2.02	0.41
1:C:8:LEU:HD23	1:C:8:LEU:C	2.40	0.41
1:J:88:TRP:CZ3	1:J:97:ALA:HB2	2.56	0.41
1:F:61:GLN:OE1	1:F:63:LYS:HE3	2.20	0.41
1:G:62:LYS:CE	4:G:301:HOH:O	2.56	0.41
1:I:1:THR:HB	1:J:92:THR:O	2.20	0.40
1:I:33:GLY:O	1:I:34:LYS:HB2	2.22	0.40

All (5) symmetry-related close contacts are listed below. The label for Atom-2 includes the symmetry operator and encoded unit-cell translations to be applied.

Atom-1	Atom-2	Interatomic distance (Å)	Clash overlap (Å)
4:B:276:HOH:O	4:E:286:HOH:O[4_455]	1.64	0.56
4:I:333:HOH:O	4:G:309:HOH:O[7_555]	1.82	0.38
4:J:276:HOH:O	4:D:232:HOH:O[7_556]	1.90	0.30
4:I:315:HOH:O	4:G:327:HOH:O[7_555]	1.97	0.23
4:D:244:HOH:O	4:H:267:HOH:O[3_555]	2.18	0.02

For Manuscript Review

5.3 Torsion angles (i)

5.3.1 Protein backbone (i)

In the following table, the Percentiles column shows the percent Ramachandran outliers of the chain as a percentile score with respect to all X-ray entries followed by that with respect to entries of similar resolution.

The Analysed column shows the number of residues for which the backbone conformation was analysed, and the total number of residues.

Mol	Chain	Analysed	Favoured	Allowed	Outliers	Percentiles	
1	A	101/103 (98%)	98 (97%)	1 (1%)	2 (2%)	7	3
1	B	101/103 (98%)	97 (96%)	3 (3%)	1 (1%)	15	11
1	C	101/103 (98%)	97 (96%)	3 (3%)	1 (1%)	15	11
1	D	101/103 (98%)	97 (96%)	2 (2%)	2 (2%)	7	3
1	E	101/103 (98%)	98 (97%)	3 (3%)	0	100	100
1	F	101/103 (98%)	101 (100%)	0	0	100	100
1	G	101/103 (98%)	99 (98%)	2 (2%)	0	100	100
1	H	101/103 (98%)	99 (98%)	2 (2%)	0	100	100
1	I	101/103 (98%)	99 (98%)	2 (2%)	0	100	100
1	J	101/103 (98%)	100 (99%)	1 (1%)	0	100	100
All	All	1010/1030 (98%)	985 (98%)	19 (2%)	6 (1%)	25	21

All (6) Ramachandran outliers are listed below:

Mol	Chain	Res	Type
1	A	59	ASP
1	D	59	ASP
1	C	55	SER
1	D	58	ILE
1	A	58	ILE
1	B	14	ASN

5.3.2 Protein sidechains (i)

In the following table, the Percentiles column shows the percent sidechain outliers of the chain as a percentile score with respect to all X-ray entries followed by that with respect to entries of similar resolution.

The Analysed column shows the number of residues for which the sidechain conformation was analysed, and the total number of residues.

For Manuscript Review

Mol	Chain	Analysed	Rotameric	Outliers	Percentiles	
1	A	89/89 (100%)	89 (100%)	0	100	100
1	B	89/89 (100%)	88 (99%)	1 (1%)	73	79
1	C	89/89 (100%)	86 (97%)	3 (3%)	37	39
1	D	89/89 (100%)	89 (100%)	0	100	100
1	E	89/89 (100%)	87 (98%)	2 (2%)	52	57
1	F	89/89 (100%)	88 (99%)	1 (1%)	73	79
1	G	89/89 (100%)	89 (100%)	0	100	100
1	H	89/89 (100%)	88 (99%)	1 (1%)	73	79
1	I	89/89 (100%)	87 (98%)	2 (2%)	52	57
1	J	89/89 (100%)	88 (99%)	1 (1%)	73	79
All	All	890/890 (100%)	879 (99%)	11 (1%)	71	77

All (11) residues with a non-rotameric sidechain are listed below:

Mol	Chain	Res	Type
1	B	9	CYS
1	C	56	GLN
1	C	63	LYS
1	C	82	VAL
1	I	43	LYS
1	I	103	ASN
1	J	82	VAL
1	E	3	GLN
1	E	9	CYS
1	F	82	VAL
1	H	103	ASN

Sometimes sidechains can be flipped to improve hydrogen bonding and reduce clashes. All (4) such sidechains are listed below:

Mol	Chain	Res	Type
1	A	3	GLN
1	A	14	ASN
1	D	57	HIS
1	F	103	ASN

5.3.3 RNA (i)

There are no RNA molecules in this entry.

For Manuscript Review

5.4 Non-standard residues in protein, DNA, RNA chains (i)

There are no non-standard protein/DNA/RNA residues in this entry.

5.5 Carbohydrates (i)

8 monosaccharides are modelled in this entry.

In the following table, the Counts columns list the number of bonds (or angles) for which Mogul statistics could be retrieved, the number of bonds (or angles) that are observed in the model and the number of bonds (or angles) that are defined in the Chemical Component Dictionary. The Link column lists molecule types, if any, to which the group is linked. The Z score for a bond length (or angle) is the number of standard deviations the observed value is removed from the expected value. A bond length (or angle) with $|Z| > 2$ is considered an outlier worth inspection. RMSZ is the root-mean-square of all Z scores of the bond lengths (or angles).

Mol	Type	Chain	Res	Link	Bond lengths			Bond angles		
					Counts	RMSZ	$\#\|Z\| > 2$	Counts	RMSZ	$\#\|Z\| > 2$
2	GAL	K	1	3,2	11,11,12	2.07	3 (27%)	15,15,17	1.42	2 (13%)
2	NGA	K	2	2	14,14,15	2.45	6 (42%)	17,19,21	1.70	6 (35%)
2	2FG	K	3	2	11,11,12	1.51	2 (18%)	10,15,17	1.18	1 (10%)
2	SIA	K	4	2	20,20,21	2.51	10 (50%)	24,28,31	1.29	4 (16%)
2	GAL	L	1	3,2	11,11,12	2.01	3 (27%)	15,15,17	3.33	6 (40%)
2	NGA	L	2	2	14,14,15	2.48	6 (42%)	17,19,21	1.31	2 (11%)
2	2FG	L	3	2	11,11,12	1.59	1 (9%)	10,15,17	0.97	0
2	SIA	L	4	2	20,20,21	2.59	8 (40%)	24,28,31	1.56	4 (16%)

In the following table, the Chirals column lists the number of chiral outliers, the number of chiral centers analysed, the number of these observed in the model and the number defined in the Chemical Component Dictionary. Similar counts are reported in the Torsion and Rings columns. '-' means no outliers of that kind were identified.

Mol	Type	Chain	Res	Link	Chirals	Torsions	Rings
2	GAL	K	1	3,2	-	1/2/19/22	0/1/1/1
2	NGA	K	2	2	-	0/6/23/26	0/1/1/1
2	2FG	K	3	2	-	0/2/19/22	0/1/1/1
2	SIA	K	4	2	-	0/18/34/38	0/1/1/1
2	GAL	L	1	3,2	-	1/2/19/22	0/1/1/1
2	NGA	L	2	2	-	0/6/23/26	0/1/1/1
2	2FG	L	3	2	-	0/2/19/22	0/1/1/1
2	SIA	L	4	2	-	0/18/34/38	0/1/1/1

All (39) bond length outliers are listed below:

For Manuscript Review

Mol	Chain	Res	Type	Atoms	Z	Observed(Å)	Ideal(Å)
2	K	4	SIA	O6-C6	5.71	1.52	1.44
2	L	4	SIA	O6-C6	5.58	1.52	1.44
2	L	4	SIA	C4-C5	-5.46	1.48	1.53
2	L	4	SIA	C5-N5	4.96	1.53	1.45
2	K	1	GAL	O5-C5	4.73	1.53	1.43
2	K	4	SIA	C4-C5	-4.48	1.49	1.53
2	K	4	SIA	C5-N5	4.39	1.52	1.45
2	L	1	GAL	O5-C5	4.27	1.52	1.43
2	L	2	NGA	C7-N2	3.95	1.47	1.34
2	L	2	NGA	C1-C2	3.95	1.58	1.52
2	L	2	NGA	O5-C5	3.90	1.51	1.43
2	K	2	NGA	C1-C2	3.84	1.58	1.52
2	K	2	NGA	O3-C3	3.82	1.52	1.43
2	K	2	NGA	C7-N2	3.80	1.47	1.34
2	L	3	2FG	O5-C5	3.80	1.51	1.43
2	L	4	SIA	C10-N5	3.75	1.47	1.34
2	K	4	SIA	C10-N5	3.57	1.46	1.34
2	K	2	NGA	O5-C5	3.54	1.50	1.43
2	L	2	NGA	O3-C3	3.51	1.51	1.43
2	K	3	2FG	O5-C5	3.49	1.50	1.43
2	L	4	SIA	C11-C10	2.79	1.56	1.50
2	L	1	GAL	O3-C3	2.77	1.49	1.43
2	K	4	SIA	C11-C10	2.70	1.56	1.50
2	L	2	NGA	C8-C7	2.58	1.55	1.50
2	K	2	NGA	C8-C7	2.57	1.55	1.50
2	K	2	NGA	C4-C3	-2.55	1.45	1.52
2	K	1	GAL	O5-C1	2.51	1.47	1.43
2	K	4	SIA	C7-C6	-2.50	1.49	1.53
2	K	1	GAL	O3-C3	2.44	1.48	1.43
2	L	4	SIA	O10-C10	-2.36	1.17	1.23
2	L	2	NGA	C4-C3	-2.33	1.46	1.52
2	K	4	SIA	O10-C10	-2.21	1.18	1.23
2	L	1	GAL	O5-C1	2.20	1.47	1.43
2	L	4	SIA	C7-C6	-2.20	1.50	1.53
2	K	4	SIA	C6-C5	-2.18	1.49	1.53
2	L	4	SIA	C6-C5	-2.06	1.49	1.53
2	K	3	2FG	C4-C3	-2.02	1.47	1.52
2	K	4	SIA	O1A-C1	2.02	1.28	1.22
2	K	4	SIA	O6-C2	2.02	1.46	1.43

All (25) bond angle outliers are listed below:

For Manuscript Review

Mol	Chain	Res	Type	Atoms	Z	Observed(°)	Ideal(°)
2	L	1	GAL	C1-C2-C3	9.83	121.75	109.67
2	L	1	GAL	O3-C3-C2	-4.06	102.21	109.99
2	K	1	GAL	C1-C2-C3	3.82	114.36	109.67
2	L	1	GAL	O5-C1-C2	3.14	115.61	110.77
2	L	4	SIA	C3-C4-C5	3.13	115.24	111.46
2	K	2	NGA	C3-C4-C5	3.11	115.79	110.24
2	L	1	GAL	C1-O5-C5	3.09	116.37	112.19
2	L	4	SIA	C8-C7-C6	-3.07	107.22	113.03
2	L	4	SIA	C11-C10-N5	3.04	121.25	116.10
2	L	1	GAL	C2-C3-C4	3.03	116.14	110.89
2	K	2	NGA	C1-C2-N2	-2.90	105.53	110.49
2	L	2	NGA	O5-C5-C6	2.72	111.47	107.20
2	K	2	NGA	C4-C3-C2	2.69	114.97	111.02
2	K	4	SIA	C4-C5-N5	-2.65	105.13	110.38
2	L	2	NGA	C1-C2-N2	-2.65	105.96	110.49
2	K	4	SIA	C3-C4-C5	2.46	114.43	111.46
2	K	4	SIA	C8-C7-C6	-2.46	108.37	113.03
2	L	4	SIA	O1B-C1-C2	2.43	119.97	113.03
2	K	3	2FG	C3-C4-C5	2.35	114.44	110.24
2	K	2	NGA	O5-C1-C2	-2.26	107.72	111.29
2	K	4	SIA	O1B-C1-C2	2.26	119.48	113.03
2	K	2	NGA	C6-C5-C4	-2.25	107.73	113.00
2	L	1	GAL	C3-C4-C5	-2.21	106.30	110.24
2	K	2	NGA	O5-C5-C6	2.15	110.57	107.20
2	K	1	GAL	O3-C3-C2	-2.03	106.10	109.99

There are no chirality outliers.

All (2) torsion outliers are listed below:

Mol	Chain	Res	Type	Atoms
2	L	1	GAL	O5-C5-C6-O6
2	K	1	GAL	O5-C5-C6-O6

There are no ring outliers.

3 monomers are involved in 4 short contacts:

Mol	Chain	Res	Type	Clashes	Symm-Clashes
2	L	2	NGA	2	0
2	K	3	2FG	1	0
2	L	4	SIA	1	0

The following is a two-dimensional graphical depiction of Mogul quality analysis of bond lengths,

For Manuscript Review

bond angles, torsion angles, and ring geometry for oligosaccharide.

Oligosaccharide Chain K

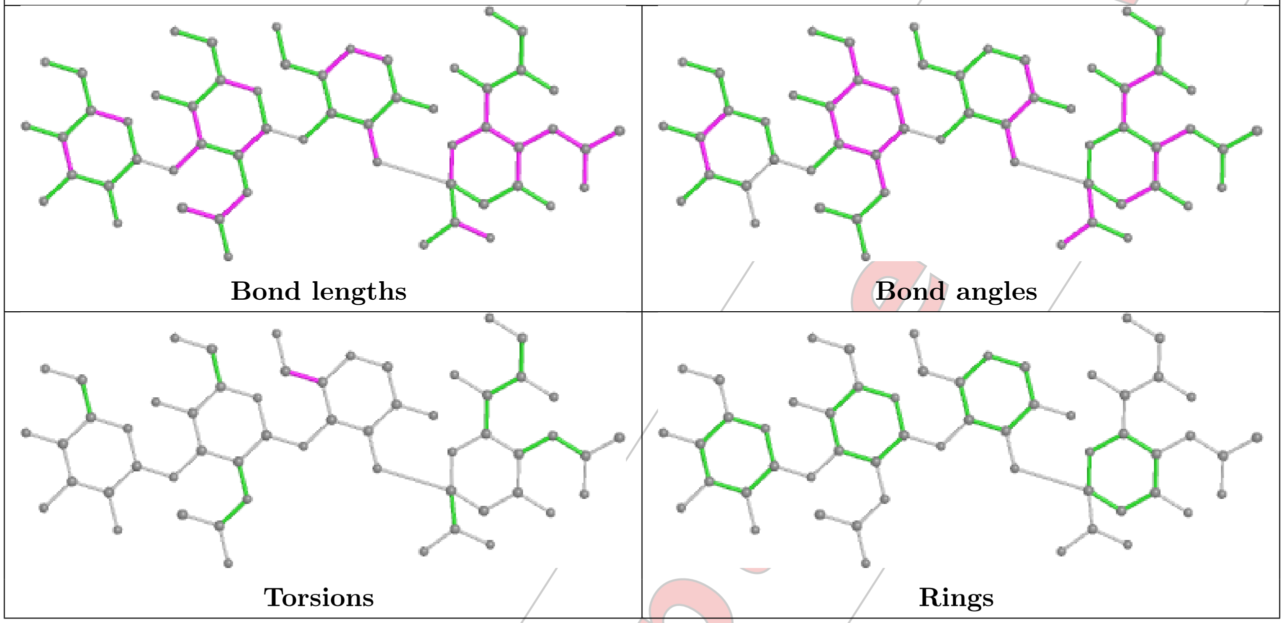

Oligosaccharide Chain L

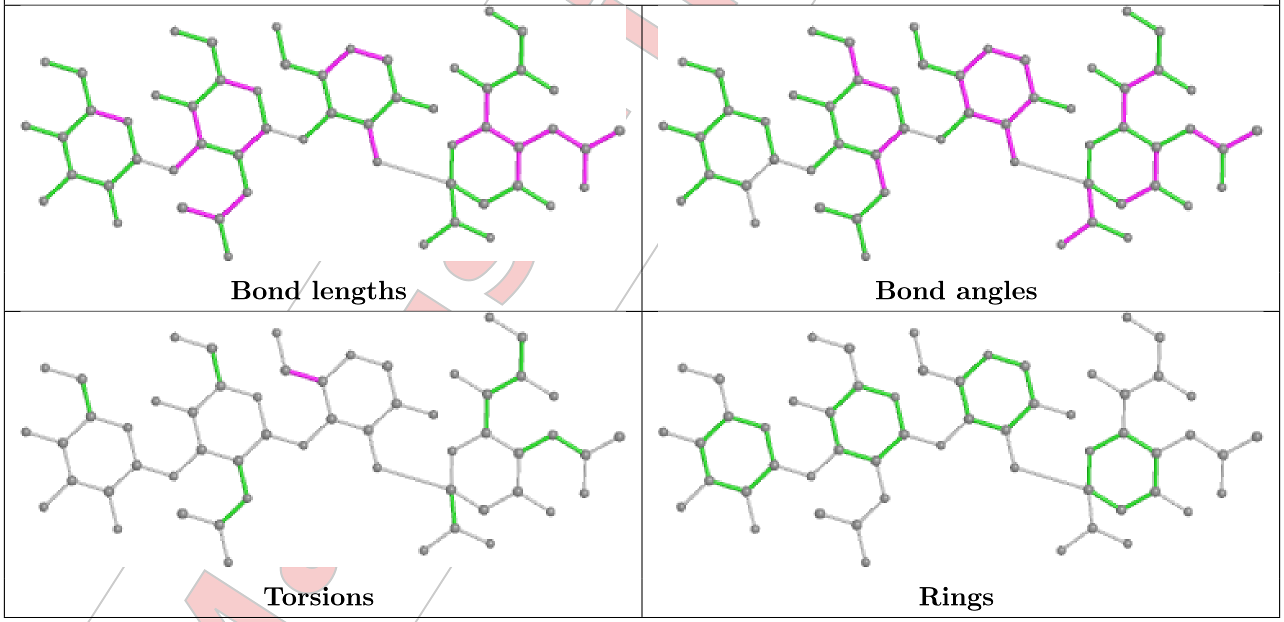

5.6 Ligand geometry (i)

2 ligands are modelled in this entry.

In the following table, the Counts columns list the number of bonds (or angles) for which Mogul statistics could be retrieved, the number of bonds (or angles) that are observed in the model and the number of bonds (or angles) that are defined in the Chemical Component Dictionary. The Link column lists molecule types, if any, to which the group is linked. The Z score for a bond length (or angle) is the number of standard deviations the observed value is removed from the

For Manuscript Review

expected value. A bond length (or angle) with $|Z| > 2$ is considered an outlier worth inspection. RMSZ is the root-mean-square of all Z scores of the bond lengths (or angles).

Mol	Type	Chain	Res	Link	Bond lengths			Bond angles		
					Counts	RMSZ	#$|Z| > 2$	Counts	RMSZ	#$|Z| > 2$
3	A1H7V	I	201	2	14,14,24	1.89	3 (21%)	17,19,29	2.24	5 (29%)
3	A1H7V	G	201	2	14,14,24	1.82	5 (35%)	17,19,29	2.00	4 (23%)

In the following table, the Chirals column lists the number of chiral outliers, the number of chiral centers analysed, the number of these observed in the model and the number defined in the Chemical Component Dictionary. Similar counts are reported in the Torsion and Rings columns. '-' means no outliers of that kind were identified.

Mol	Type	Chain	Res	Link	Chirals	Torsions	Rings
3	A1H7V	I	201	2	-	5/5/25/35	0/1/1/1
3	A1H7V	G	201	2	-	2/5/25/35	0/1/1/1

All (8) bond length outliers are listed below:

Mol	Chain	Res	Type	Atoms	Z	Observed(Å)	Ideal(Å)
3	I	201	A1H7V	C2-C3	-3.64	1.49	1.52
3	G	201	A1H7V	C2-C3	-3.40	1.49	1.52
3	I	201	A1H7V	O4-C4	3.29	1.50	1.43
3	G	201	A1H7V	O4-C4	3.20	1.50	1.43
3	I	201	A1H7V	O5-C1	3.13	1.49	1.41
3	G	201	A1H7V	O5-C1	2.94	1.49	1.41
3	G	201	A1H7V	O3-C3	2.28	1.48	1.43
3	G	201	A1H7V	C3-C4	-2.01	1.47	1.52

All (9) bond angle outliers are listed below:

Mol	Chain	Res	Type	Atoms	Z	Observed(°)	Ideal(°)
3	I	201	A1H7V	O5-C5-C4	6.19	120.94	109.69
3	G	201	A1H7V	O5-C5-C4	4.72	118.27	109.69
3	G	201	A1H7V	O1-C1-C2	4.17	116.13	108.25
3	I	201	A1H7V	C6-C5-C4	-3.62	104.53	113.00
3	I	201	A1H7V	CAA-O1-C1	2.89	120.44	113.92
3	G	201	A1H7V	C1-O5-C5	-2.49	108.81	113.69
3	I	201	A1H7V	F1-C2-C1	2.39	110.25	107.57
3	G	201	A1H7V	O4-C4-C5	-2.27	103.65	109.30
3	I	201	A1H7V	C1-O5-C5	2.12	117.86	113.69

There are no chirality outliers.

For Manuscript Review

All (7) torsion outliers are listed below:

Mol	Chain	Res	Type	Atoms
3	I	201	A1H7V	C2-C1-O1-CAA
3	I	201	A1H7V	O5-C1-O1-CAA
3	I	201	A1H7V	CAB-CAA-O1-C1
3	I	201	A1H7V	O5-C5-C6-O6
3	I	201	A1H7V	C4-C5-C6-O6
3	G	201	A1H7V	CAB-CAA-O1-C1
3	G	201	A1H7V	C2-C1-O1-CAA

There are no ring outliers.

2 monomers are involved in 4 short contacts:

Mol	Chain	Res	Type	Clashes	Symm-Clashes
3	I	201	A1H7V	2	0
3	G	201	A1H7V	2	0

The following is a two-dimensional graphical depiction of Mogul quality analysis of bond lengths, bond angles, torsion angles, and ring geometry for all instances of the Ligand of Interest. In addition, ligands with molecular weight > 250 and outliers as shown on the validation Tables will also be included. For torsion angles, if less then 5% of the Mogul distribution of torsion angles is within 10 degrees of the torsion angle in question, then that torsion angle is considered an outlier. Any bond that is central to one or more torsion angles identified as an outlier by Mogul will be highlighted in the graph. For rings, the root-mean-square deviation (RMSD) between the ring in question and similar rings identified by Mogul is calculated over all ring torsion angles. If the average RMSD is greater than 60 degrees and the minimal RMSD between the ring in question and any Mogul-identified rings is also greater than 60 degrees, then that ring is considered an outlier. The outliers are highlighted in purple. The color gray indicates Mogul did not find sufficient equivalents in the CSD to analyse the geometry.

For Manuscript Review

Ligand A1H7V I 201

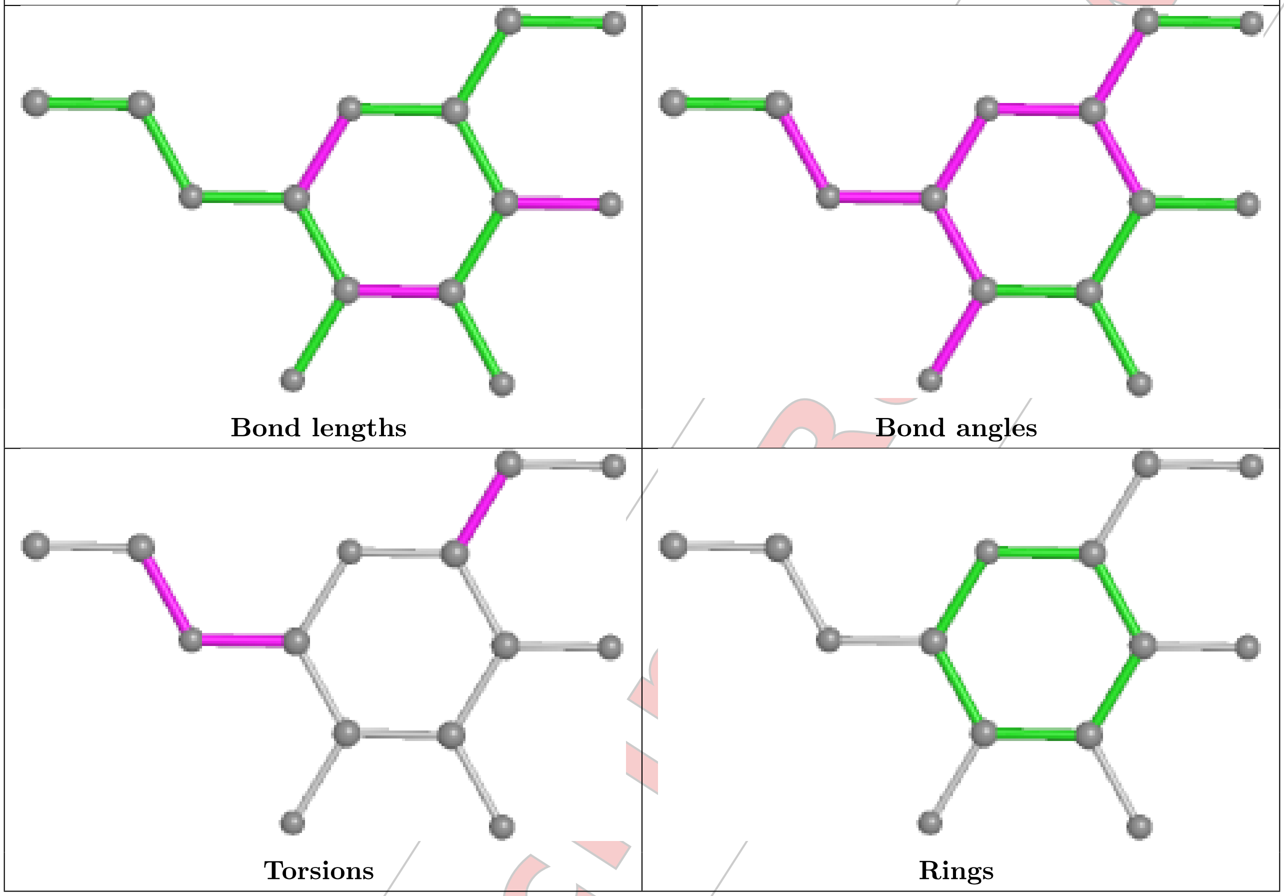

For Manuscript Review

Ligand A1H7V G 201

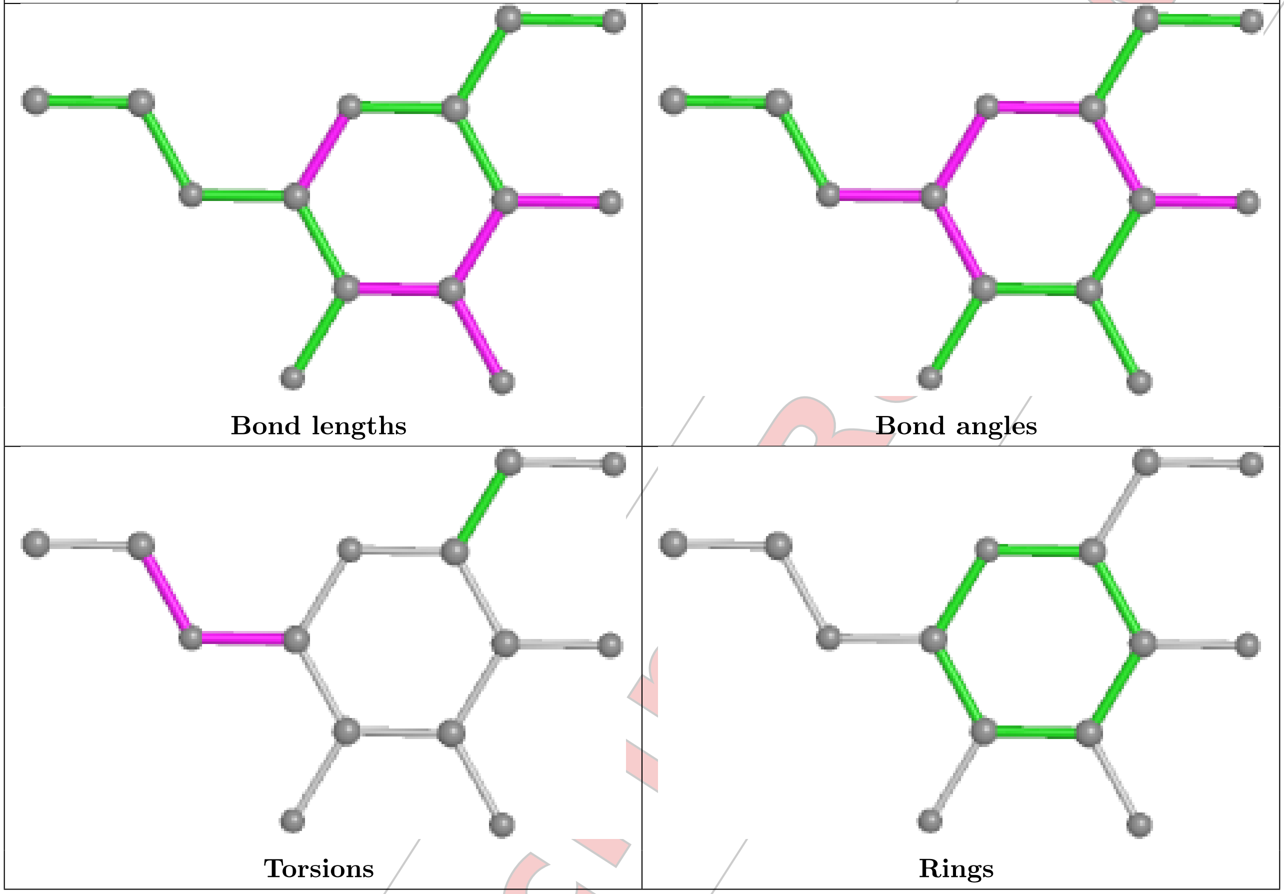

5.7 Other polymers (i)

There are no such residues in this entry.

5.8 Polymer linkage issues (i)

There are no chain breaks in this entry.

For Manuscript Review

6 Fit of model and data (i)

6.1 Protein, DNA and RNA chains (i)

In the following table, the column labelled '#RSRZ> 2' contains the number (and percentage) of RSRZ outliers, followed by percent RSRZ outliers for the chain as percentile scores relative to all X-ray entries and entries of similar resolution. The OWAB column contains the minimum, median, 95th percentile and maximum values of the occupancy-weighted average B-factor per residue. The column labelled 'Q< 0.9' lists the number of (and percentage) of residues with an average occupancy less than 0.9.

Mol	Chain	Analysed	<RSRZ>	#RSRZ>2			OWAB(Å^2)	Q<0.9
1	A	103/103 (100%)	-0.07	4 (3%)	39	45	21, 28, 66, 89	1 (0%)
1	B	103/103 (100%)	-0.20	1 (0%)	82	85	22, 29, 48, 80	0
1	C	103/103 (100%)	0.05	6 (5%)	23	28	24, 35, 72, 97	0
1	D	103/103 (100%)	-0.08	3 (2%)	51	57	20, 27, 68, 89	1 (0%)
1	E	103/103 (100%)	-0.37	0	100	100	22, 28, 37, 51	0
1	F	103/103 (100%)	-0.16	0	100	100	24, 32, 45, 64	0
1	G	103/103 (100%)	-0.29	2 (1%)	66	71	24, 31, 50, 77	0
1	H	103/103 (100%)	-0.25	1 (0%)	82	85	21, 30, 46, 66	0
1	I	103/103 (100%)	-0.23	0	100	100	25, 33, 52, 74	0
1	J	103/103 (100%)	-0.27	1 (0%)	82	85	22, 30, 47, 78	0
All	All	1030/1030 (100%)	-0.19	18 (1%)	70	74	20, 31, 52, 97	2 (0%)

All (18) RSRZ outliers are listed below:

Mol	Chain	Res	Type	RSRZ
1	C	56	GLN	5.4
1	A	58	ILE	4.6
1	A	59	ASP	4.6
1	B	103	ASN	4.4
1	G	103	ASN	3.8
1	D	58	ILE	3.7
1	D	59	ASP	3.6
1	D	60	SER	3.5
1	C	59	ASP	3.0
1	J	103	ASN	2.8
1	A	56	GLN	2.7
1	C	103	ASN	2.7
1	H	103	ASN	2.6

Continued on next page...

For Manuscript Review

Continued from previous page...

Mol	Chain	Res	Type	RSRZ
1	C	55	SER	2.4
1	C	57	HIS	2.4
1	C	13	HIS	2.3
1	G	55	SER	2.2
1	A	57	HIS	2.1

6.2 Non-standard residues in protein, DNA, RNA chains (i)

There are no non-standard protein/DNA/RNA residues in this entry.

6.3 Carbohydrates (i)

In the following table, the Atoms column lists the number of modelled atoms in the group and the number defined in the chemical component dictionary. The B-factors column lists the minimum, median, 95th percentile and maximum values of B factors of atoms in the group. The column labelled 'Q< 0.9' lists the number of atoms with occupancy less than 0.9.

Mol	Type	Chain	Res	Atoms	RSCC	RSR	B-factors(Å^2)	Q<0.9
2	NGA	K	2	14/15	0.40	0.73	83,100,114,125	0
2	NGA	L	2	14/15	0.42	0.85	80,106,131,132	0
2	2FG	L	3	11/12	0.56	0.55	62,81,110,119	0
2	2FG	K	3	11/12	0.62	0.55	55,77,103,124	0
2	GAL	L	1	11/12	0.70	0.19	82,100,119,123	0
2	GAL	K	1	11/12	0.81	0.20	72,95,106,119	0
2	SIA	K	4	20/21	0.87	0.15	42,55,67,68	0
2	SIA	L	4	20/21	0.88	0.13	43,61,80,89	0

The following is a graphical depiction of the model fit to experimental electron density for oligosaccharide. Each fit is shown from different orientation to approximate a three-dimensional view.

For Manuscript Review

Electron density around Chain K:

2mF$_o$-DF$_c$ (at 0.7 rmsd) in gray
mF$_o$-DF$_c$ (at 3 rmsd) in purple (negative)
and green (positive)

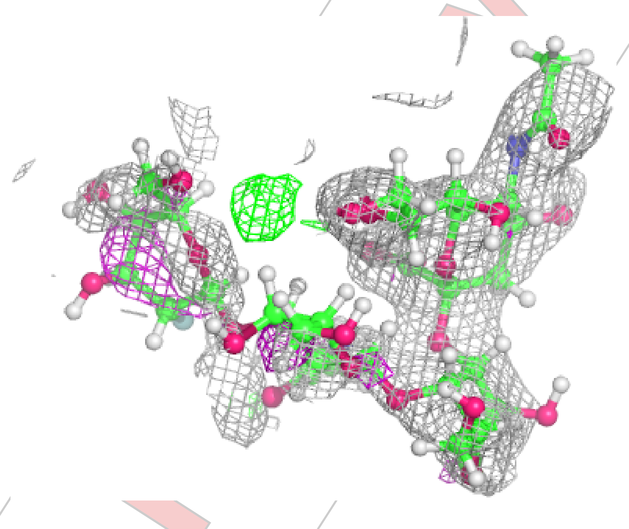

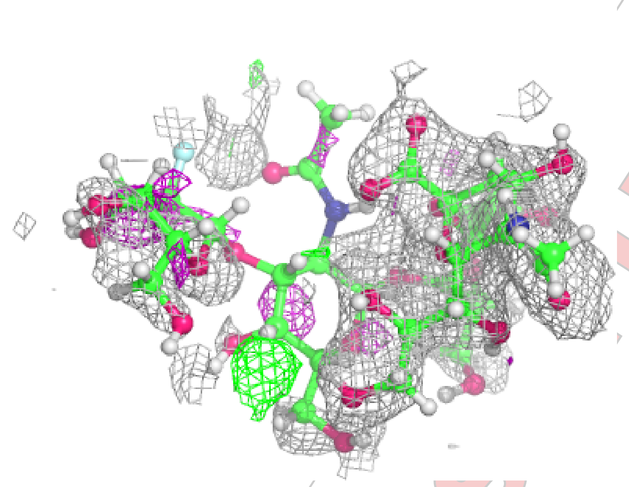

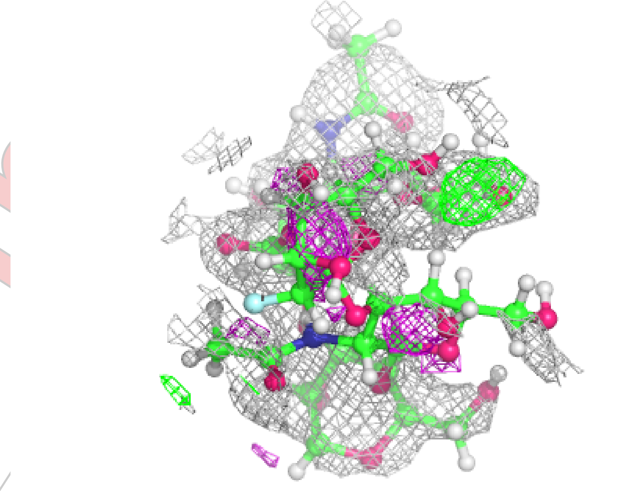

For Manuscript Review

Electron density around Chain L:

2mF$_o$-DF$_c$ (at 0.7 rmsd) in gray
mF$_o$-DF$_c$ (at 3 rmsd) in purple (negative)
and green (positive)

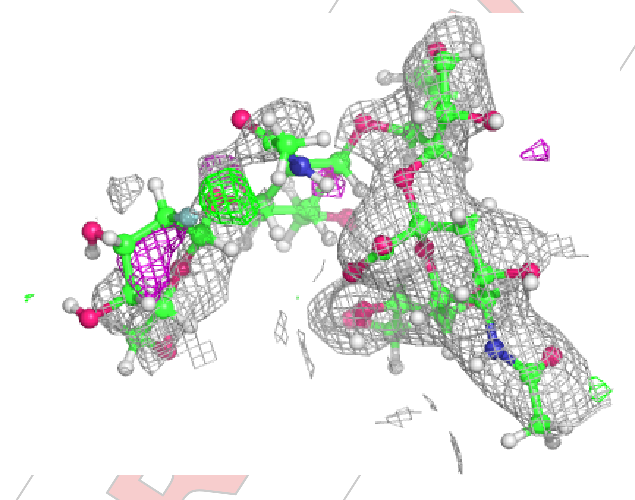

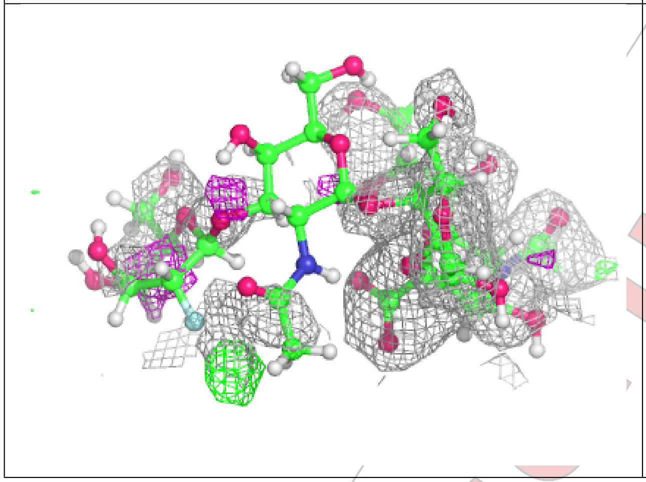

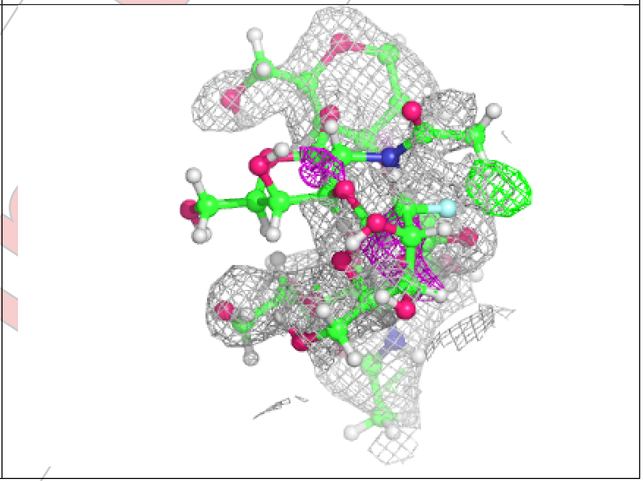

6.4 Ligands ⓘ

In the following table, the Atoms column lists the number of modelled atoms in the group and the number defined in the chemical component dictionary. The B-factors column lists the minimum, median, 95th percentile and maximum values of B factors of atoms in the group. The column labelled 'Q< 0.9' lists the number of atoms with occupancy less than 0.9.

Mol	Type	Chain	Res	Atoms	RSCC	RSR	B-factors(Å^2)	Q<0.9
3	A1H7V	I	201	14/24	0.48	0.65	91,109,126,131	0
3	A1H7V	G	201	14/24	0.51	0.52	86,118,141,143	0

The following is a graphical depiction of the model fit to experimental electron density of all instances of the Ligand of Interest. In addition, ligands with molecular weight > 250 and outliers as shown on the geometry validation Tables will also be included. Each fit is shown from different orientation to approximate a three-dimensional view.

For Manuscript Review

Electron density around A1H7V I 201:

$2mF_o$-DF_c (at 0.7 rmsd) in gray
mF_o-DF_c (at 3 rmsd) in purple (negative)
and green (positive)

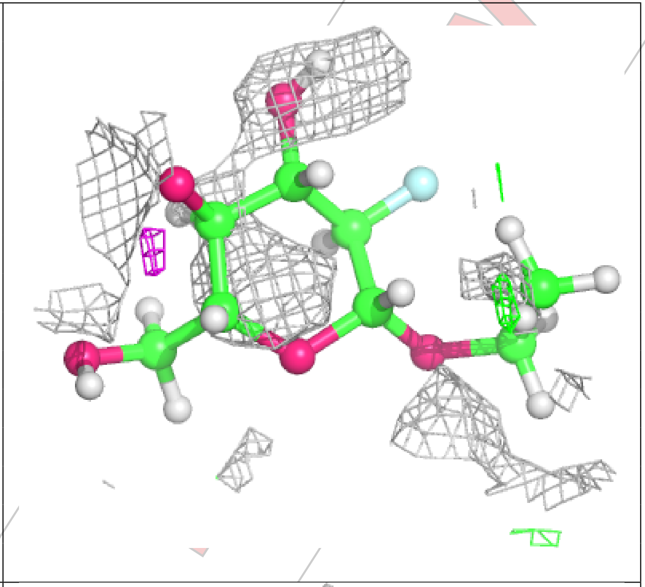

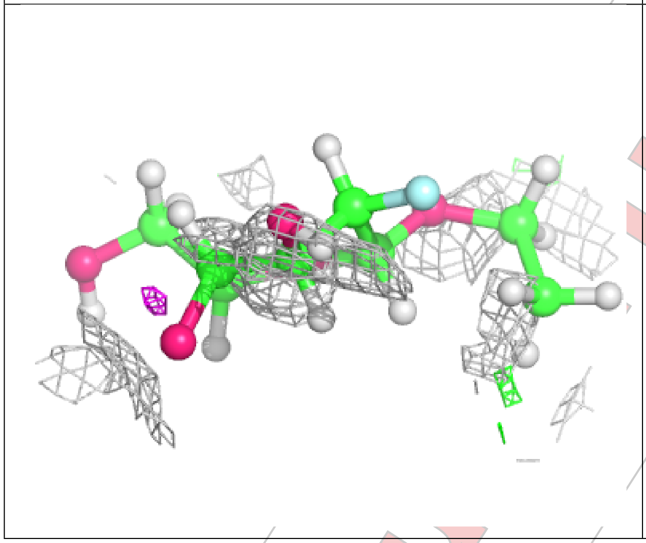

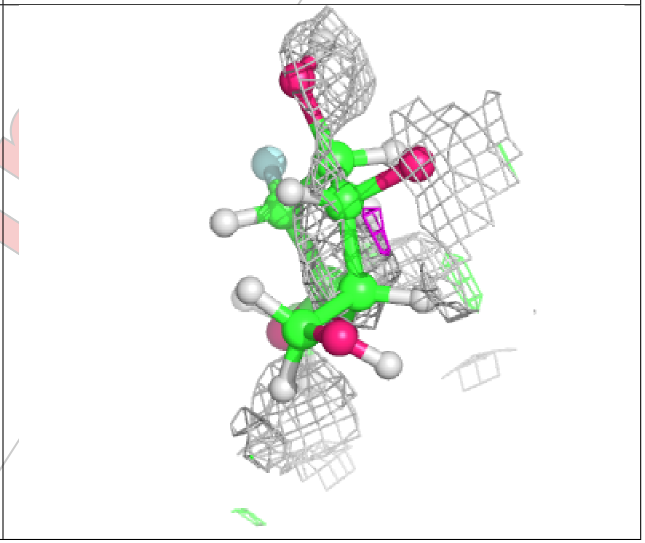

For Manuscript Review

Electron density around A1H7V G 201:

$2mF_o$-DF_c (at 0.7 rmsd) in gray
mF_o-DF_c (at 3 rmsd) in purple (negative)
and green (positive)

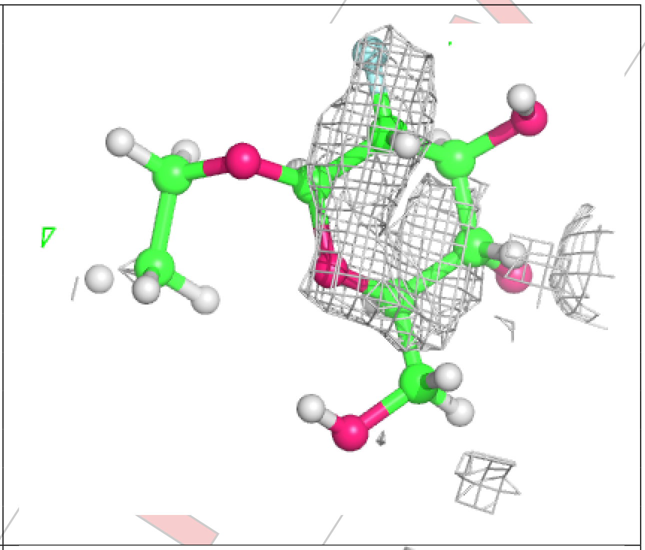

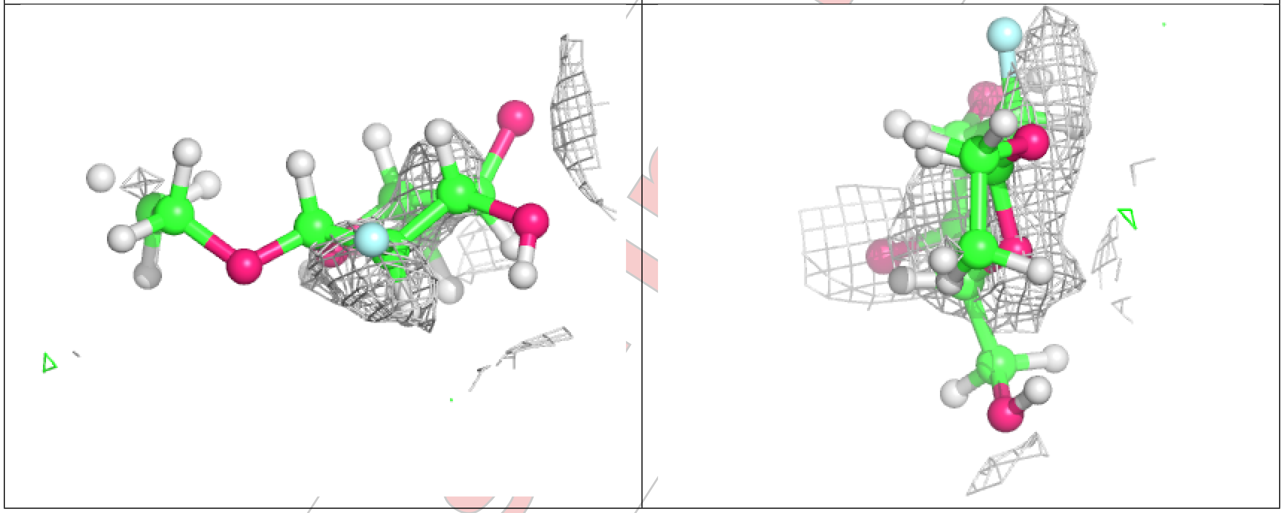

6.5 Other polymers (i)

There are no such residues in this entry.

For Manuscript Review
